# Evolution-Guided Engineering of *Trans*-Acyltransferase Polyketide Synthases

**DOI:** 10.1126/science.adj7621

**Published:** 2024-03-21

**Authors:** Mathijs F.J. Mabesoone, Stefan Leopold-Messer, Hannah A. Minas, Clara Chepkirui, Pornsuda Chawengrum, Silke Reiter, Roy A. Meoded, Sarah Wolf, Ferdinand Genz, Nancy Magnus, Birgit Piechulla, Allison S. Walker, Jörn Piel

**Affiliations:** 1 Institute of Microbiology, Eidgenössische Technische Hochschule (ETH) Zürich, Vladimir-Prelog-Weg 4, 8093 Zürich, Switzerland.; 2 Chemical Biology Program, Chulabhorn Graduate Institute, Chulabhorn Royal Academy, Bangkok 10210, Thailand.; 3 Institute for Biological Sciences, University of Rostock, Albert-Einstein-Straße 3, 18059 Rostock, Germany.; 4 Department of Biological Chemistry and Molecular Pharmacology, Harvard Medical School, 240 Longwood Avenue, Boston, Massachusetts 02115, United States.; 5 Department of Chemistry, Vanderbilt University, 1234 Stevenson Center Lane, Nashville, Tennessee 37240, United States.; 6 Department of Biological Sciences, Vanderbilt University, 465 21st Avenue S, Nashville, Tennesee 37232, United States.; 7 Lead contact

## Abstract

Bacterial multimodular polyketide synthases (PKSs) are giant enzymes that generate a wide range of therapeutically important but synthetically challenging natural products. Diversification of polyketide structures can be achieved by engineering these enzymes. However, notwithstanding successes made with textbook, *cis*-acyltransferase (*cis*-AT) PKSs, tailoring such large assembly lines remains challenging. Unlike textbook PKSs, *trans*-AT PKSs feature an extraordinary diversity of PKS modules and commonly evolve to form hybrid PKSs. Here, we analyze amino acid coevolution to identify a common module site that yields functional PKSs. We use this site to insert and delete diverse PKS parts and create 22 engineered *trans*-AT PKSs from various pathways and in two bacterial producers. The high success rates of our engineering approach highlight the broader applicability to generate complex designer polyketides.

## One-sentence summary

Evolutionary insights enable the engineered biosynthesis of designer polyketides, an important class of bioactive natural products.

Bacteria are a rich source of bioactive natural products, many of which have found pharmacological applications (1). Among the most therapeutically useful compounds are complex polyketides produced by large megaenzymes termed polyketide synthases (PKSs) (2–4). These assembly line-like proteins are composed of multiple modules, each introducing a specific part of the final structure. Biosynthesis is achieved by stepwise incorporation and modification of small acyl-CoA-derived building blocks. Textbook multimodular PKSs, coined *cis*-AT PKSs, largely contain modules employing fatty acid synthase-type biochemistry. A minimal module consists of an acyl carrier protein (ACP) domain tethering the polyketide intermediate, a ketosynthase (KS) domain catalyzing the chain elongation, and an acyl transferase (AT) domain selecting the building blocks. Optional additional modifications at the β-carbon, such as reductions by ketoreductases (KR) or dehydrations by dehydratases (DH), diversify the polyketide scaffold to generate chemical complexity. The sequence of modules is represented in the chemical structure of the final product – a phenomenon termed the collinearity principle. This correspondence at the protein and chemical level has inspired the vision to create designer PKSs from module parts that produce synthetically challenging polyketides in a predictable fashion (5). Efforts towards engineering have been successful for the erythromycin PKS and other model systems (6–8), yet design rules that enable combinatorial biosynthesis with high success rates remain elusive (9). The observation that KSs from *cis*-AT systems typically form distinct clades with other KSs from the same cluster (10) suggests the formation of natural hybrids by recombination is rare in *cis*-AT PKSs, which might represent an intrinsic challenge to engineering of this family of PKSs.

A second large family of bacterial multimodular PKSs, termed *trans*-AT PKSs, differ from *cis*-AT assembly lines (11). They commonly contain modules catalyzing non-fatty acid synthase-type reactions, such as halogenation (12, 13), formation of diverse heterocycles (14–16), oxygen insertion (17, 18), α-hydroxylation (13), and β-branching (15, 19). This unparalleled biochemical diversity provides a vast combinatorial space to diversify polyketide structures in a modular fashion. *Trans*-AT PKSs evolve via widespread recombination between biosynthetic gene clusters (BGCs) to form mosaic-like natural hybrids (20). A common recombination site is located at a region corresponding to the C-terminus of KS domains (21, 22), i.e., KSs coevolve with modifying domains located directly upstream (20, 23). Together, the apparent natural combinatorial evolution of *trans*-AT PKSs and their expanded chemical repertoire make this class of enzymes highly attractive for engineering efforts. Guidelines to engineer these megaenzymes remain, however, unclear from the few reported modified *trans*-AT PKSs (24, 25). Here, we leverage insights obtained from natural *trans*-AT PKS evolution to uncover a fusion site that allows construction of diverse engineered, large PKS assembly lines in several organisms.

## Fusions at a conserved NAHVILEE motif result in stalled intermediates

In a first attempt to identify the natural recombination sites in *trans*-AT PKSs, we extracted 821 KS sequences with their adjacent up- and downstream regions from our in-house database of annotated *trans*-AT PKS biosynthetic gene clusters (20, 26). A multiple sequence alignment showed a conserved NAHVILEE motif near the C-terminus of KSs (Fig. S1). In many natural *trans*-AT PKS hybrids with shared module series (21, 22), we observed that pairwise sequence similarity dropped off behind the NAHVILEE motif of the terminal, shared module. However, a putative recombination site could not be precisely localized within a ca. 100 amino acid region due to high sequence divergences even among architecturally closely related PKS hybrids. Since the NAHVILEE motif was also reported as a functional fusion site to yield functional *cis*-AT PKS chimeras (27, 28), we explored its utility for *trans*-AT engineering using the bacillaene (*pks*) biosynthetic gene cluster from *Bacillus subtilis* (29, 30). Four chimeric PKSs were generated by genomic integration, resulting in PKSs with non-native terminal modules (Figs. S1–6, see Supplementary Information for additional details). However, instead of full-length polyketides, we detected products that resulted from hydrolytic release of stalled intermediates just before the fusion point. The data suggested that none of the chimeras was functional and that the NAHVILEE site is not suitable for engineering in this PKS.

## Statistical coupling analysis suggests the LPTYPFx_5_W motif as potential recombination site

**Figure 1.**
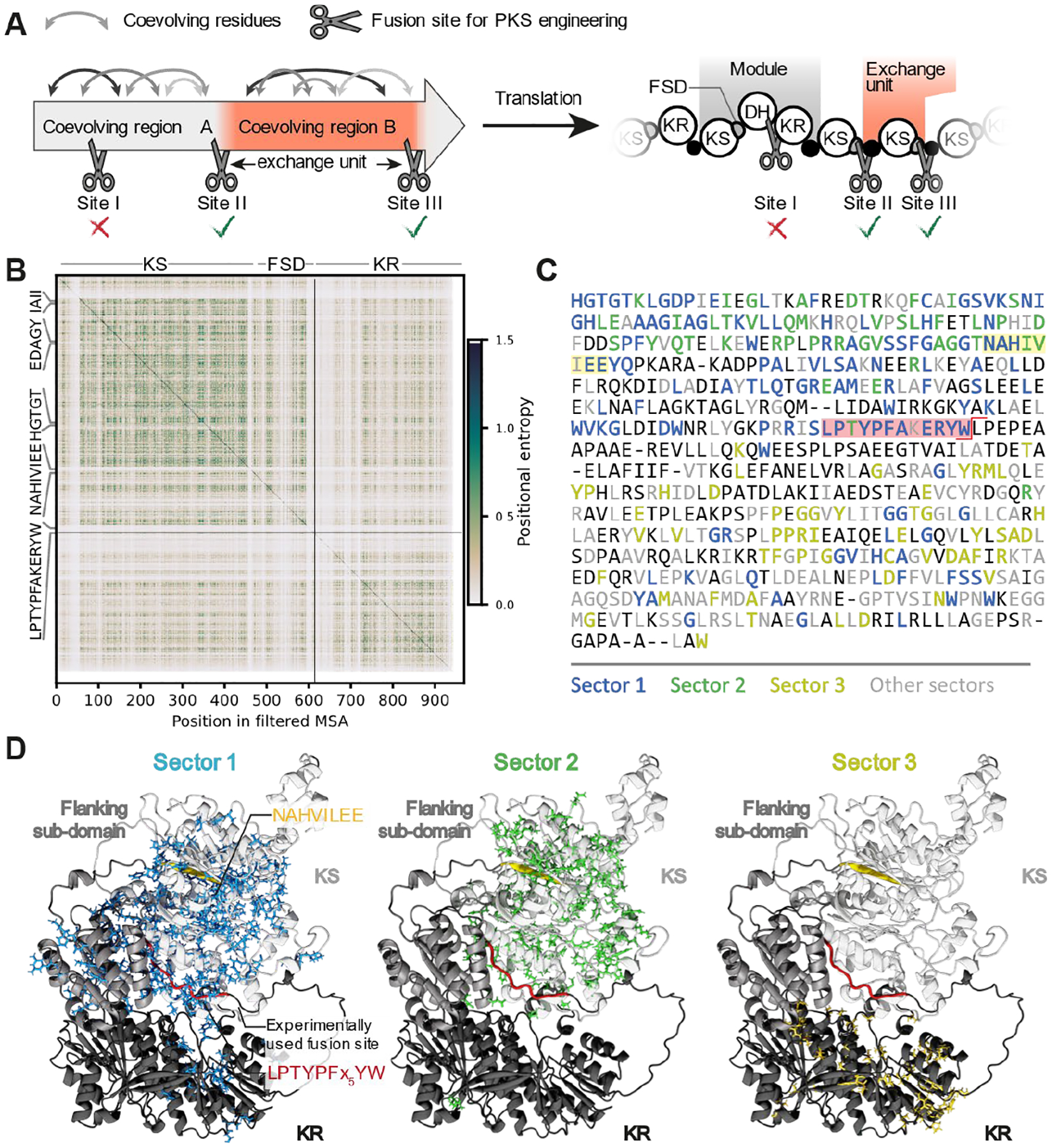
SCA identifies the LPTYPFx_5_W motif as a candidate fusion point for chimeric *trans*-AT PKSs. (**A**) Engineering within regions of coevolving residues is hypothesized to perturb important amino acid interactions, yielding non-functional chimeras (site I). In contrast, engineering at boundaries between regions of coevolving residues minimizes such perturbations and might lead to functional chimeras (sites II and III). At the protein level, *trans*-AT PKS exchange units span between the C-terminal boundaries of the FSDs, which slightly contrasts with the commonly used PKS module boundaries, spanning from KS to ACP. (**B**) Covariance matrix of Clustal-aligned (53) multiple sequence alignments of the KS-FSD-KR tridomain, showing the positional entropy obtained from SCA for amino acid residue pairs of the tridomain. The position of conserved IAII, HGTGT, NAHVILEE, and LPTYPFx_5_W motifs are indicated on the side. (**C**) Consensus sequence of the C-terminal part of the KS-FSD-KR tridomain obtained from the conservation-filtered sequence alignment used for SCA. Residues are color-coded according to SCA sectors. Residues not assigned to sectors are black. The NAHVILEE motif and the experimentally used engineering site downstream of the LPTYPFx_5_W motif is indicated with the yellow and red shades, respectively. (**D**) 3D visualizations of sector 1 (left), sector 2 (middle), and sector 3 (right) on an alphaFold2-generated (54) model of the OocR KS-FSD-KR tridomain. Colors correspond to residue colors in panel B.

To infer the site at which *trans*-AT PKSs potentially recombine, we analyzed amino acid coevolution with statistical coupling analysis (SCA) (31, 32). This method analyzes covariance of amino acids in multiple sequence alignments and identifies global networks of coevolving residues. We reasoned that this method might reveal structurally or functionally interconnected networks of amino acid residues that might be sensitive to disruption by engineering (Fig. 1A). Due to dynamic processes during polyketide elongation as well as lateral interactions between assembly lines that create a multitude of domain contacts (33, 34), such residue interactions remain obscure when using the available structural snapshots of *trans*-AT PKS components (35, 36). As such, we hypothesized that SCA might uncover engineering sites at boundaries between independent networks of coevolving residues that minimally disrupt evolutionary conserved interactions in *trans*-AT PKSs and thereby enable engineering of productive chimeric *trans*-AT PKSs. For analysis, we extracted protein sequences that encompass the KS domain and commonly occurring neighboring regions from manually collected *trans*-AT PKSs and *trans*-AT PKSs deposited in the antiSMASH database (Fig. S9) (37) and analyzed sequence alignments with SCA (see Supplementary Materials). The amino acid covariance in the alignments of the extracted motifs reveals coevolution within each modifying domain (e.g., KS and KR, Figs. 1B, S10–12). In addition, covariance between KS domains and upstream modifying domains is also apparent, which is in line with the previous observation that KSs clade according to the polyketide modifications introduced by these upstream modifying domains (Figs. S10–12) (20, 23). We additionally observed coevolution of residues within the KS and a C-terminal region termed flanking subdomain (FSD) (Figs. 1B, S10–12). Although the precise function of the FSD is unclear, it has been found to mediate lateral interactions between PKSs to form higher-order, supramolecular PKS assemblies (33, 36, 38–40), suggesting this subdomain plays an important role in the organizational dynamics of polyketide biosynthesis.

To test whether this coevolution between KS and FSD is significant, we deconvoluted the amino acid covariance and extracted networks of statistically significantly coevolving positions. The most significant of the amino acid networks, coined sectors, has been associated with conserved residues and general enzyme stability (41), whereas other significant sectors that contain less-conserved residues are associated with more specialized enzyme functionality (41, 42). We consistently found that the LPTYPFx_5_W motif at the FSD C-terminus acts as boundary between sectors containing lesser-conserved residues that are presumably involved in enzyme functionality (Figs. 1C, D, S13). This suggested that the LPTYPFx_5_W motif, which also occurs in *cis*-AT PKSs downstream of the AT domain and has been successfully used in AT swapping experiments in *cis*-AT PKSs (43, 44), separates evolutionarily autonomous parts in *trans*-AT PKSs. In line with terminology from NRPS engineering (45), we use the term “exchange units” for these evolutionarily autonomous parts that contain various domains (25, 34, 46), while “module” refers to biochemically functional KS-to-ACP sections (Fig. 1A).

## A *Serratia plymuthica* platform for PKS engineering

**Figure 2:**
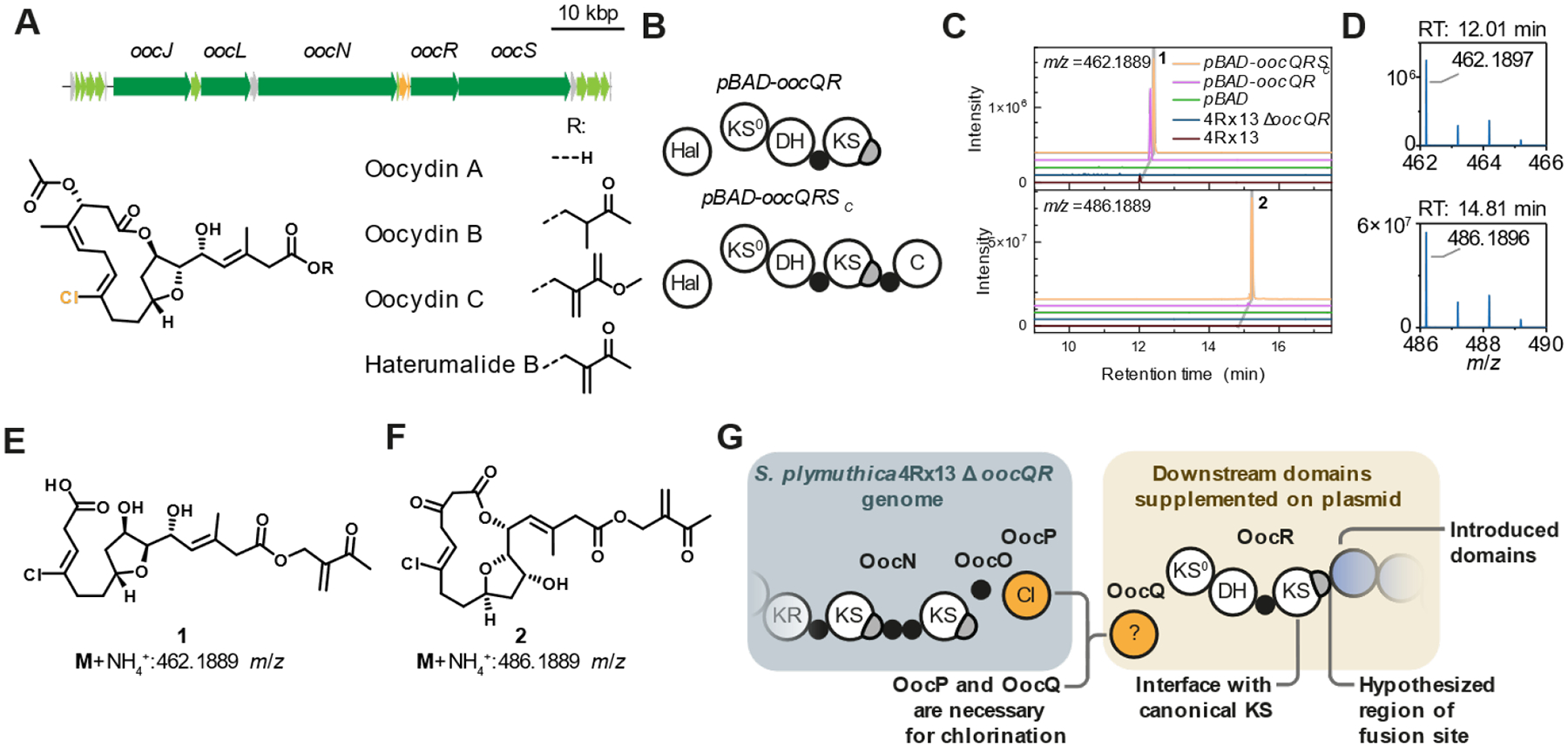
The oocydin BGC as platform for PKS engineering. (**A**) The oocydin BGC from *S. plymuthica* 4Rx13 and chemical structures of natural oocydin congeners. Core PKS genes are shaded dark green, accessory biosynthetic genes light green, *oocP* and *oocQ* are shaded yellow. (**B**) Domain motifs of truncated OocR PKSs and OocQ as supplemented with the *pBAD-oocQR* and *pBAD-oocQRS_C_* plasmids. (**C**) EICs for **1**+NH_4_^+^ (top) and **2**+NH_4_^+^ (bottom) for *S. plymuthica*, *S. plymuthica* Δ*oocQR* and *S. plymuthica* Δ*oocQR* supplemented empty *pBAD*, *pBAD*-*oocQR* and *pBAD*-*oocQRSC* plasmids. (**D**) Isotope patterns confirm chlorination of **1** (top) and **2** (bottom). (**E**) Putative chemical structures of **1** and (**F**) NMR-confirmed structure of **2**. (**G**) The engineering strategy relies on supplementing *oocQ* and engineered variants of *oocR* on a *pBAD* plasmid.

To experimentally assess whether the computationally suggested LPTYPFx_5_W motif can serve as an artificial fusion site, we developed a screening platform using the oocydin BGC in the genetically tractable bacterium *Serratia plymuthica* 4Rx13 (henceforth termed *S. plymuthica*) (13, 17). The oocydin *trans*-AT PKS contains biochemically diverse modules including a halogenation module that catalyzes chlorination during polyketide chain elongation (13, 47). This module comprises a heterodimer of the *trans*-acting Fe(II)/α-ketoglutarate-dependent halogenase OocP and the auxiliary protein OocQ (Figs. 2A, S14). By using the chlorination module at the engineering interface, we hoped to introduce a characteristic chlorine isotope tag into hybrid polyketides that would facilitate their mass spectrometric detection in bacterial extracts (47).

High-performance liquid chromatography coupled to mass spectrometry (HPLC-MS) analysis of culture extracts of *S. plymuthica* Δ*oocQR*, a mutant lacking *oocQ* and the downstream PKS gene *oocR*, showed that chlorination of polyketide intermediates was indeed abolished (Fig. 2C). We next supplemented *oocQ* to *S. plymuthica* Δ*oocQR* on a plasmid encoding *oocQ* and *oocR* up to the LPTYPFx_5_W motif of the second KS of OocR (*pBAD-oocQR*) (Fig. 2B, top). Besides restoring oocydin production (Fig. S30), supplementation of *oocQR* also resulted in the production of a compound with an *m*/*z* value corresponding to compound **1** (Figs. 2C–E), the free acid of the putative polyketide intermediate at the OocR ACP domain (13), thereby confirming restored chlorination by supplementation of *oocQ*. With the aim to facilitate processivity of the truncated assembly line, we next appended the terminal ACP-C didomain of OocS to the truncated OocR. This didomain natively offloads the polyketide intermediate by catalyzing macrolactonization (13). Extracts resulted in larger amounts of a product with an additional C2 extension. NMR-based structural elucidation after HPLC-MS-guided fractionation confirmed the macrolactone structure of **2** (Figs. 2C, D, F, S17–21). *S. plymuthica* Δ*oocQR* thus provides a platform for introducing engineered *oocR* mutants to assess guidelines for *trans*-AT PKS engineering (Fig. 2G).

## Introducing foreign domains after the LPTYPFx_5_W motif yields functional chimeric PKSs

**Figure 3:**
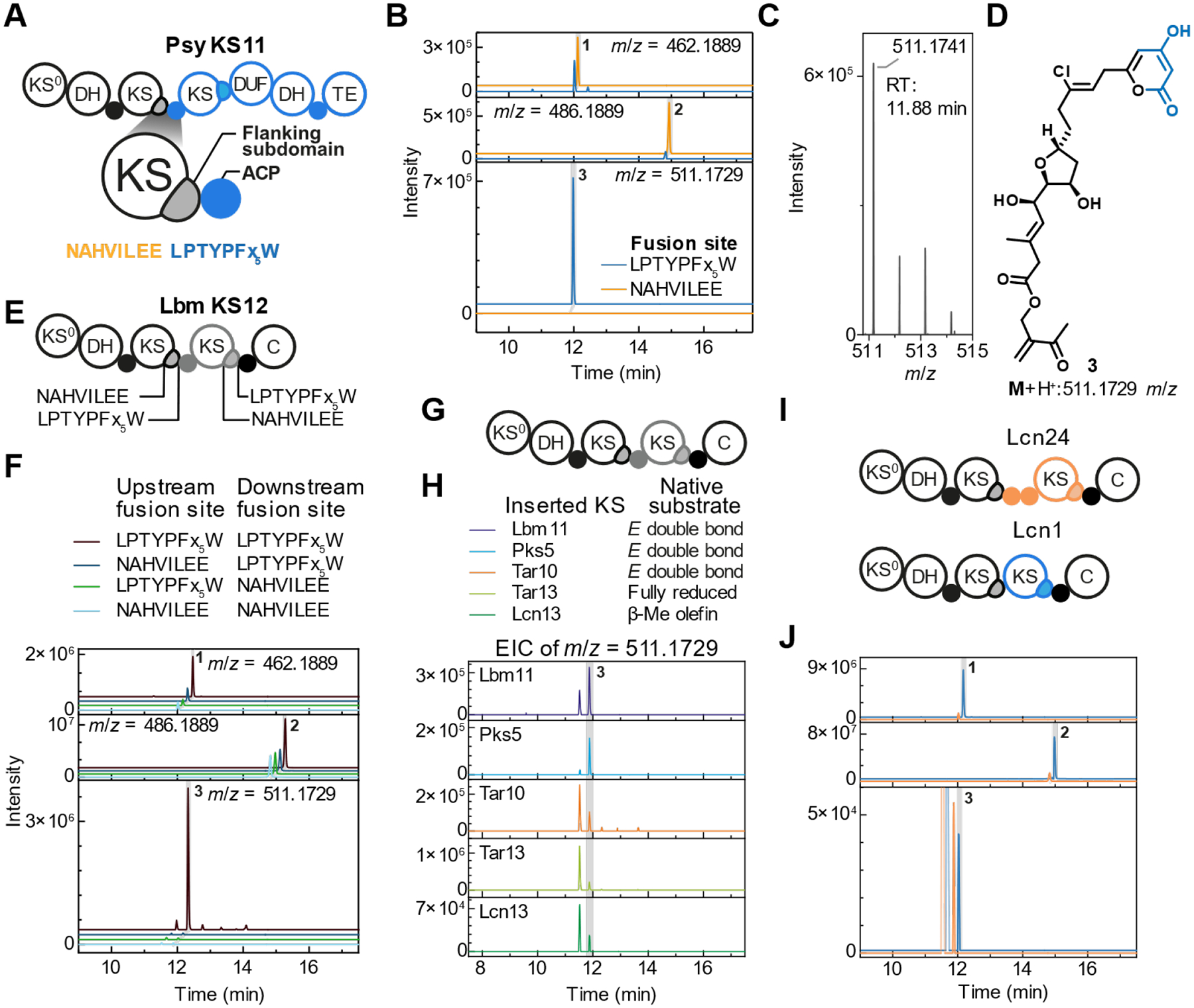
Insertion of KS domains yields various functional chimeric PKSs. (**A**) Domain architecture of chimeric PKSs incorporating the terminal domains of the psymberin (*psy*) PKS. The two fusion sites are indicated in the enlarged region. (**B**) Extracted ion chromatograms (EIC)s for **1**, **2**, and **3** from mutant cultures harboring the *ooc-psy* chimeras. (**C**) Mass spectrum of **3** at a retention time of 11.88 min showing the characteristic peak pattern indicative of chlorination. (**D**) NMR-characterized structure of **3**. (**E**) Domain architecture of the various chimeric PKSs incorporating lobatamide (*lbm*) KS12 with its native upstream ACP. The two fusion sites upstream and downstream of the inserted ACP-KS domain series are indicated. (**F**) EICs for **1**, **2**, and **3** from mutant cultures harboring the *ooc-lbm-ooc* chimeras. (**G**) Domain architecture of additional chimeric PKSs incorporating ACP-KS domain series with various foreign non-matching KSs, i.e., which naturally accept substrates other than β-ketoacyl thioesters. (**H**) EICs for **3** of mutant cultures harboring these chimeric PKSs. (**I**) Domain architecture of the two chimeric PKSs incorporating ACP-KS domain series from N-termini of PKS proteins. (**J**) EICs for **1**, **2**, and **3** from mutant cultures harboring the chimeric PKSs. The peak eluting at 11.51 minutes originating from a prematurely offloaded intermediate has been blurred. See Fig. S16 for additional details.

Having generated a functional engineered PKS with native *ooc* parts, we next explored the compatibility of the LPTYPFx_5_W site with a foreign component. To minimize the number of non-native interactions, we introduced a single fusion site by joining the truncated *oocR* to the terminus of the psymberin (*psy*) BGC from an uncultivated bacterium (21). The *psy* region encodes the domain series ACP-KS-DUF-DH-ACP (DUF: domain of unknown function), and an offloading thioesterase (TE) domain that jointly catalyze β-keto extension and δ-lactone formation (Fig. 3A). The *psy* KS natively accepts a β-ketoacyl intermediate (21), as would be produced by the upstream minimal ACP-KS domain series that is the result of the PKS fusion (13). As a control experiment, we constructed a PKS with the same domains, but the fusion point located at the NAHVILEE motif of the OocR KS. HPLC-MS analysis of medium extracts showed that only the LPTYPFx_5_W-fused chimera suggested by SCA produces a chlorinated compound with an *m*/*z* value of 511.1729, corresponding to the expected, doubly extended product **3** (Figs. 3B, C), whereas the NAHVILEE-fused chimera yielded offloaded intermediates **1** and **2**. HPLC-guided purification and structural elucidation confirmed **3** as a pyrone resulting from two keto extensions and δ-lactone formation (Figs. 3D, S22–26).

Having established the utility of the LPTYPx_5_W site for new PKS termini, we next aimed to insert foreign domains between domains of the *ooc* assembly line. For this, we placed an exchange unit containing a β-ketoacyl-accepting minimal ACP-KS12 domain series from the lobatamide (*lbm*) PKS of *Gynuella sunshinyii* (18) between the truncated OocR and the OocS ACP C terminus (Fig. 3E). If functional, this PKS would catalyze two terminal keto extensions as for the *psy* construct. In addition to the SCA-conform LPTYPx_5_W double fusion, we prepared three control constructs with one or both *lbm* fusion sites exchanged to the NAHVILEE motif (Figs. 3E, F). HPLC-MS analysis showed that fusion the LPTYPx_5_W motif at both sites led to notable production of **3**, whereas fusion at the NAHVILEE motif at any of the two sites primarily showed stalled biosynthesis, indicated by only trace amounts of **3**. Mutants employing a fusion site upstream of the OocR KS12, which was shown to yield productive truncated disorazol PKSs (24), were not productive (Fig. S15). We thus concluded that the LPTYPx_5_W motif provides an engineering site for chimeric *trans*-AT PKSs and used this fusion site in our further experiments.

## A wide range of exchange units with minimal ACP-KS domain series is tolerated

Excised KS domains can accept and elongate substrates *in vitro* that differ from the natively encountered polyketide intermediate, albeit at lower rates (14). This promiscuity suggests that the β-ketoacyl thioesters presented by the truncated OocR PKS can be extended by foreign KS domains that natively elongate different substrates. To test this hypothesis, we constructed five chimeric PKSs with ACP-KS inserts that naturally process reduced intermediates (Figs. 3G, H). As above, foreign domains were located between the truncated OocR and OocSC. Two of these chimeric PKSs, containing exchange units harboring *lbm* KS11 and *pks* KS5 (48), were excised from larger, dehydrating domain series, whereas the remaining three, harboring KS10 and KS13 from the tartrolon BGC (*tar*) (20) and KS13 from the lacunalide (*lcn*) BGC, both from *G. sunshinyii* (26), occur naturally in a minimal KS-ACP architecture. We observed production of **3** for all mutants (Fig. 3H). However, 3 to 50-fold lower HPLC-MS intensities suggested impaired processivity of the chimeric assembly lines, likely due to decreased rates of elongation. This hypothesis is additionally supported by the increased intensity of signals attributable to hydrolysis products of stalled intermediates with masses near-identical to the mass of **3**, but slightly shorter retention time (Figs. 3H, S16). While these lower intensities suggest that matching KSs increase titers in PKS engineering, the notion that KS specificity might be important for engineering *trans*-AT PKSs (49, 50) seems not to be stringent. Encouraged by the six functional chimeras containing exchange units of foreign domain series from interior protein regions, we also tested ACP-KS domain series from the N-termini of PKS proteins at the same engineering site. A chimeric PKS containing *lcn* KS24 with its upstream tandem ACP also produced **3**, albeit at considerably lower titers than those observed for constructs incorporating domain series excised from internal modules (Fig. 3J). Unexpectedly, **3** was also produced by a chimera harboring the ACP-less KS1 from the start of the entire *lcn* assembly line, i.e., containing a tandem KS (Fig. 3I). Collectively, these data show that *trans*-AT PKS engineering at the LPTYPFx_5_W motif enables incorporation of minimalACP-KS domain series from diverse biosynthetic context with variable processivity.

## Chimeric assembly lines with foreign β-keto-modifying domain series

**Figure 4:**
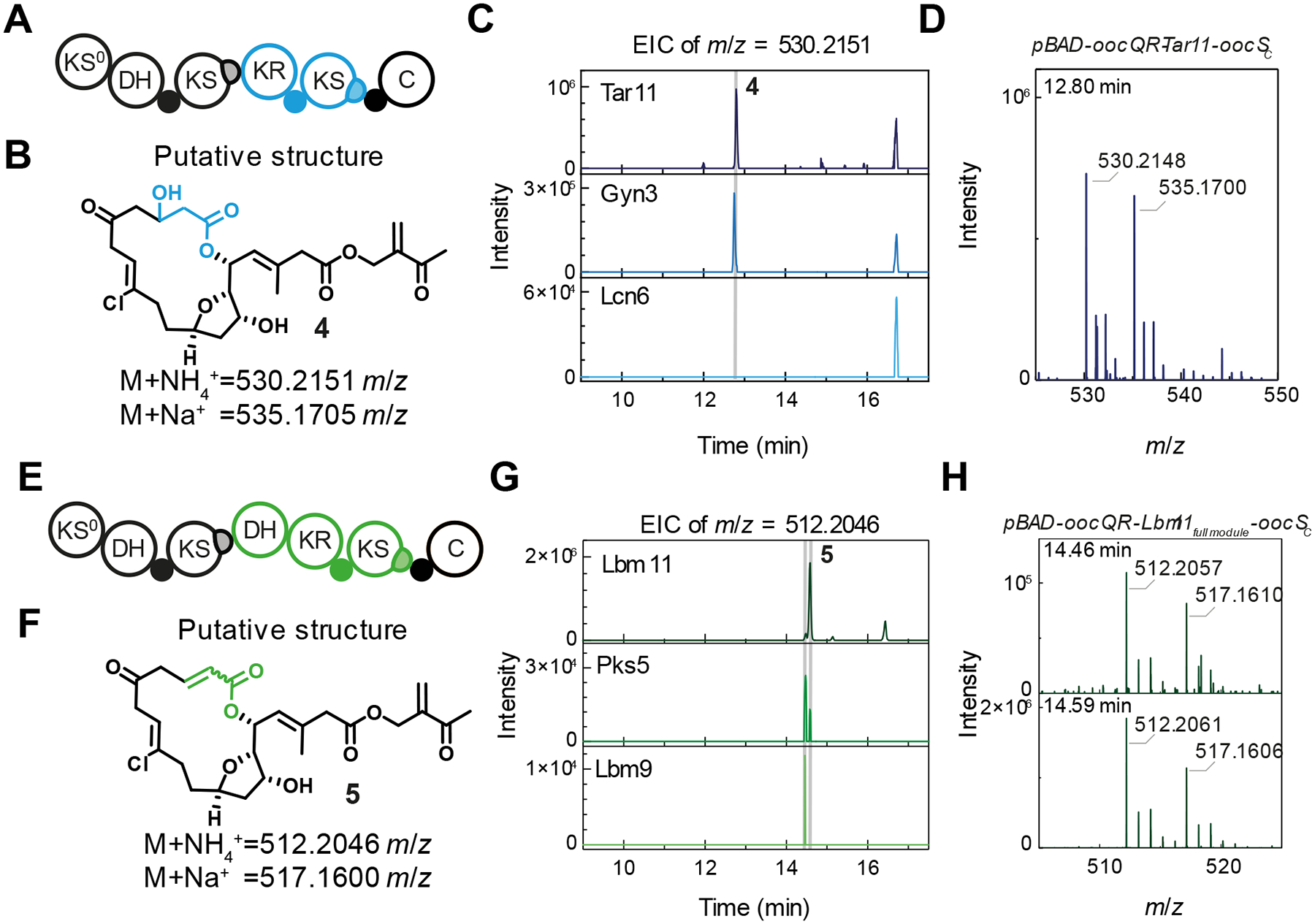
Domain series with additional modifying domains can be engineered into chimeric PKSs. (**A**) Domain architecture of the chimeric PKS incorporating reducing domain series. (**B**) Putative structure of hydroxylated polyketide **4**. (**C**) EICs for *m*/*z*=530.2151 of extracts of cultures of mutants expressing chimeric, reducing PKSs. The peak assigned to **4** is indicated by the shaded area. (**D**) Mass spectrum of the peak eluting at 12.80 minutes from culture extracts of *S. plymuthica pBAD-oocQR-tar11-oocS_C_*, showing masses and chlorination isotopes patterns corresponding to NH_4_^+^ and Na^+^ adducts of **4**. (**E**) Domain architecture of the chimeric PKS incorporating dehydrating domain series. (**F**) Putative structure of dehydrated polyketide **5**. (**G**) EICs for *m*/*z*=512.2046 of extracts of cultures of mutants expressing chimeric, dehydrating PKSs. (**H**) Mass spectra of the peaks eluting at 14.46 and 14.59 minutes from culture extracts of *S. plymuthica pBAD-oocQR-lbm11*_DH-KR-ACP-KS_*-oocS_C_*, showing masses and chlorination isotopes patterns corresponding to NH_4_^+^ and Na^+^ adducts of **5**.

Having shown that our engineering strategy allows for insertion of exchange units of diverse minimal ACP-KS domain series into a PKS, we interrogated the engineering scope for exchange units of domain series that contain additional modifying domains. First, we inserted reducing exchange units comprising KR, ACP, and KS domains into the test site (Fig. 4A). The selected exchange units contain *tar* KS11, *gyn* KS3, and *lcn* KS6 from the lacunalide, gynuellalide, and tartrolon pathways, respectively. For the *gyn* and *tar* chimeras, HPLC-MS analysis showed a chlorinated compound with *m*/*z* values corresponding to the singly extended and reduced product **4** (Figs. 4A–D). Lastly, we inserted exchange units of dehydrating domain series incorporating *lbm* KS9, *lbm* KS11, and *pks* KS5 with the architecture DH-KR-ACP-KS into OocR (Fig. 4E). The resulting mutants produced two chlorinated, isobaric compounds eluting around 14.5 minutes, suggesting the presence of *E*- and *Z*-isomers of the singly extended and dehydrated polyketide **5** (Figs. 4F–H). Although the low titers precluded isolation of **4** and **5**, the presence of both ammonium and sodium adducts of these compounds and their absence in *S. plymuthica* Δ*oocQR* extracts (Figs. 4D, H, S28) suggest that the engineering strategy also allows for the incorporation of exchange units that not only elongate but also modify the polyketide intermediate.

## Engineering at LPTYPFx_5_W motifs enables biosynthesis of truncated lacunalides

**Figure 5:**
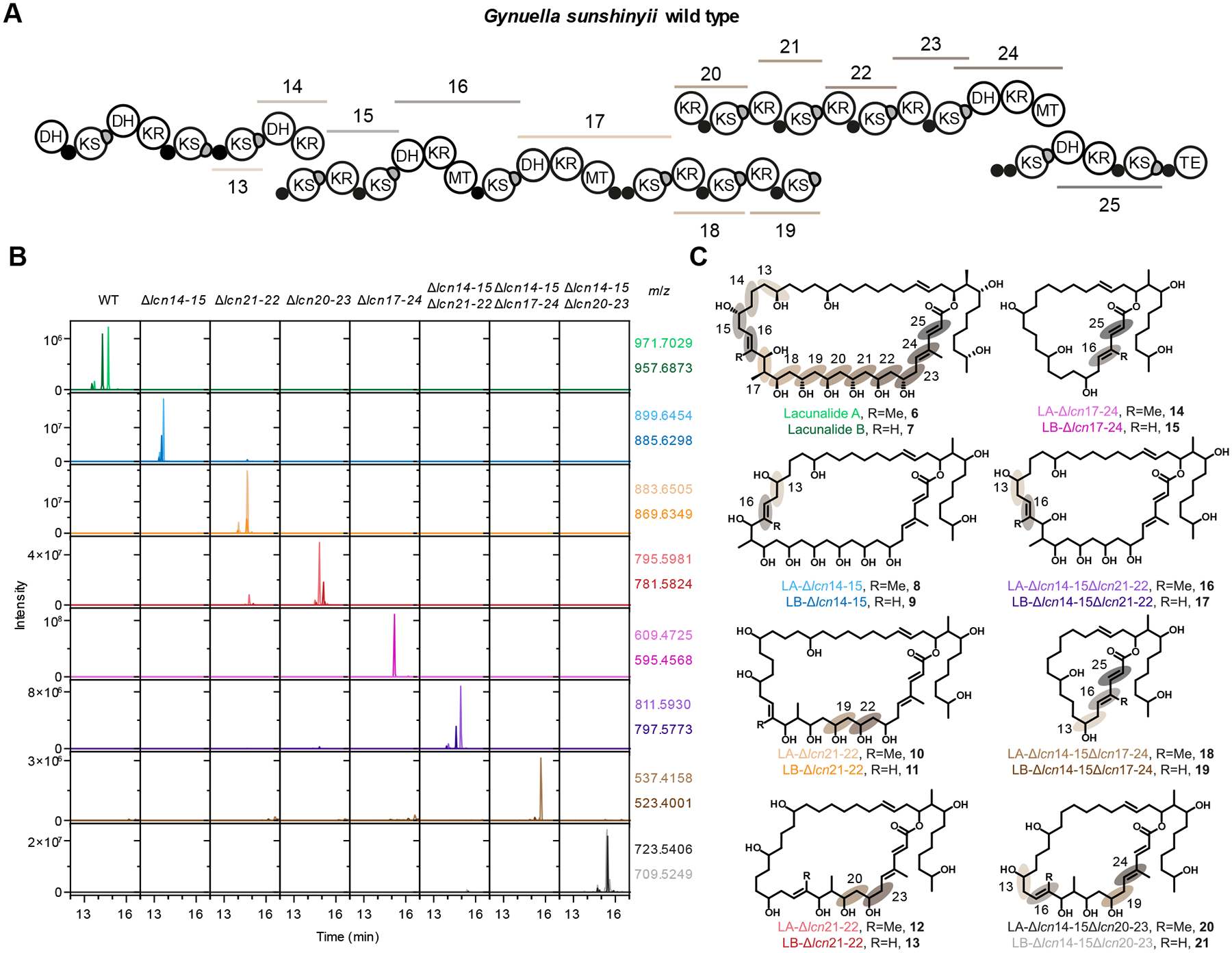
The engineering strategy enables biosynthesis of truncated lacunalides. (**A**) The domain architecture of the last PKSs in the lacunalide (*lcn*) PKS in wild type (WT) *G*. *sunshinyii* YC6258. (**B**) EICs for *m*/*z* values corresponding to proton adducts of compounds **6**–**21** for the various *G. sunshinyii* mutants. Each column shows the EICs obtained for the mutant mentioned above the column. The rows show the EICs obtained for *m*/*z* values indicated at the right of the row. Masses corresponding to **15** and **19** could not be observed. (**C**) Chemical structures of wild-type lacunalide A (6), lacunalide B (7), the NMR-confirmed truncated lacunalides **8**,**9**, **10**, **11**, **12**, **14**, **16**, **18**, **20**, and **21** and putative structures of truncated lacunalides **13** and **17**. The shaded numbered circles indicate the moieties installed by the corresponding *lcn* domain series.

We further tested the generality of the engineering strategy by applying it to a different bacterial host and assembly line, i.e., the lacunalide PKS of *G. sunshinyii* YC6258 (Fig. 5A). Using the statistically identified LPTYPFx_5_W motif, we deleted exchange units of *lcn* modules 14 and 15 containing a DH-KR-ACP-KS-KR-ACP-KS domain series, to produce the mutant *G. sunshinyii* YC6258 Δ*lcn14–15* (Figs. 5A, B). In this design, we specifically aimed to match the α-δ regions of the putative polyketide intermediate with the moiety that is naturally accepted by the downstream KS (Fig. S54). Based on biosynthetic logic, a functional engineered PKS would not generate lacunalides A, **6**, and B, **7**, but instead the spliced lacunalides **8** and **9** (Figs. 5B, C). In line with this hypothesis, MS features corresponding to **8** and **9** were only observed in culture extracts of *G. sunshinyii* YC6258 Δ*lcn14–15*, whereas production of **6** and **7** was completely abolished in this mutant. HPLC purification and combined NMR and MS analysis confirmed the structure of truncated lacunalides **8** and **9** (Figs. S62–79). The stable genomic integration of the *lcn* PKSs truncation allows combination of multiple such modifications. While taking the above-mentioned design principles into account, we then deleted three further exchange unit series in the *lcn* PKS, covering modules 21–22; 20–23 and 17–24 (Fig. 5A) from the wild-type producer as well as from the mutant *G. sunshinyii* YC6258 Δ*lcn14–15*. HPLC-MS analysis of culture extracts of the respective mutants revealed distinct MS features corresponding to truncated lacunalides **10**–**21** (Fig. 5B). To verify the processivity of all members of this engineered PKS library, we isolated compounds from each *G*. *sunshinyii* mutant and determined the structure by NMR (Figs. 5B, S80–121). Isolated yields of lacunalide A analogues **8**, **10**, **12** and **20** (0.4–0.7 mg/mL) and lacunalide B analogues **9**, **11** and **21** (0.04–0.21 mg/L) were comparable to yields of the parent lacunalide A (0.7 mg/L) and lacunalide B (0.15 mg/L) (51), while the other metabolites were isolated with reduced yields (0.03–0.26 mg/L) (Table S17). Cytotoxicity assays against Henrietta Lacks (HeLa) cervical cancer cells furthermore showcase the utility of our engineering strategy in elucidating structure-activity relationships in synthetically challenging polyketides (Fig. S122). In summary, the seven constructed mutants produced at least 12 different new compounds. As such, these results show that the engineering strategy not only enables introduction of foreign domains into *trans*-AT PKS, but also enables the reductive combinatorial biosynthesis of lacunalide congeners.

## Conclusion

We demonstrate that evolution-guided engineering of *trans*-AT PKSs at the FSD LPTYPFx_5_W motif provides a useful strategy to construct functional chimeric and truncated complex assembly lines. We show its broad applicability by successfully engineering 22 large, chimeric assembly lines comprising parts from diverse PKSs and using two host organisms and study the bioactivity of several engineered metabolites. The discovery of this engineering principle, in combination with the exceptional biochemical diversity of *trans*-AT PKS modules, offers potential for combinatorial biosynthesis of *de novo* designed, synthetically challenging polyketides including structure-activity relationship studies and drug improvement, pharmacophore discovery and introduction, and the biotechnological production of stereochemically complex fine chemicals.

## Supplementary Material

supplementary material

MDAR reproducibility report

## Data Availability

All data, scripts and plasmid maps that support the claims in this manuscript are available on the Zenodo repository (https://doi.org/10.5281/zenodo.8146702) (52). Scripts for SCA and HPLC-MS analysis are also available at www.github.com/mathijs-m/PKS_engineering_SCA and www.github.com/mathijs-m/Engineered_PKS_MS_analysis, respectively.
