# Supplementary material for: Evolution-Guided Engineering of Trans-Acyltransferase Polyketide Synthases
Source: Science. Author manuscript; Available in PMC 2024 Jul 20. (PMC11260071; doi:10.1126/science.adj7621)
Supplement: supplementary material [file NIHMS2008389-supplement-supplementary_material.pdf]

Science
AAAS

Supplementary Materials for

Evolution-guided engineering of *trans*-acyltransferase polyketide synthases

Mathijs F. J. Mabesoone *et al*.

Corresponding author: Jörn Piel, jpiel@ethz.ch

Science **383**, 1312 (2024)
DOI: 10.1126/science.adj7621

The PDF file includes:

Materials and Methods
Tables S1 to S19
Figs. S1 to S122
References

Other Supplementary Material for this manuscript includes the following:

MDAR Reproducibility Checklist

Materials and Methods

Experimental Procedures

General

Restriction endonucleases, T4 polymerase and NEBuffer 2.1 were obtained from New England BioLabs (Ipswich, MA, USA). For Gibson assembly, the Gibson assembly kit from New England BioLabs (Ipswich, MA, USA) was used. T4 ligase and buffer for Golden Gate cloning were obtained from Promega (Fitchburg, WI, USA). PCR was performed with Phusion polymerase (Thermo Fisher Scientific, Waltham, MA, USA) or Q5 polymerase (New England Biolabs, Ipswich, MA, USA) according to manufacturer's instructions. DNA sequencing was conducted by GATC Biotech (Konstanz, Germany), Microsynth (Balgach, Switzerland) or Plasmidsaurus (Eugene, OR, USA). The pJET1.2/blunt and pCR-Blunt II-TOPO cloning kits were purchased from Thermo Fisher Scientific (Waltham, MA, USA) and the pGEM-T Easy subcloning kit was obtained from Promega (Fitchburg, WI, USA). For sonication, a Sonicator Q700 from QSonica, Newton was used and Ni-NTA agarose for protein purification was purchased from Macherey-Nagel. Gel purification and plasmid extraction kits were purchased from Macherey-Nagel. High-pressure liquid chromatography high resolution mass spectrometry (HPLC-HRMS) was performed on a Thermo Scientific Q Exactive or LTQ Orbitrap XL mass spectrometer coupled to a Dionex Ultimate 3000 UPLC system operated by Xcalibur 4.1 and Chromeleon Xpress 7.2 (Thermo Scientific), respectively.

Strains and culture conditions

B. subtilis, *E. coli*, and *S. plymuthica* strains used in this study are listed in Table S1. We used *B. subtilis* DK1042 which is a derivative of the *B. subtilis* 3610 wild-type strain with increased competence due to a single point mutation in the *comI* gene negatively regulating natural competence (*55*). Strains were grown in LB liquid medium and on LB agar plates at 37 °C. To select plasmids in *E. coli* and *S. plymuthica*, antibiotics were used at final concentrations as follows: Ampicillin 100 µg/mL, chloramphenicol 12.5 µg/mL, gentamycin 20 µg/mL, kanamycin 50 µg/mL, and spectinomycin 50 µg/mL. To select for positive transformants in *B. subtilis*, antibiotics were used at final concentrations of 60 µg/mL spectinomycin and 5 µg/mL chloramphenicol. *Gynuella sunshinyii* YC6258^T was obtained from NITE Biological Resource Center (NBRC).

Plasmid construction for *Bacillus* engineering

As an acceptor vector, pFusA was used. The plasmid was a gift from Adam Bogdanove & Daniel Voytas (Addgene plasmid #31028). All gene fragments and resistance genes used in Golden Gate cloning were amplified with primers containing the BsaI restriction sites (Table S3). All PCR fragments were subcloned into pJET1.2/blunt, pCRBlunt II-TOPO or pGEM-T Easy according to manufacturer's instructions. Golden Gate cloning was performed as published by Engler and Marillonnet using pFusA/Spc as an acceptor vector (*56*, *57*). Additionally, the

resistance cassette was replaced by the gentamycin resistance cassette from pIC20H-RL to get a second pFusA/Gm acceptor plasmid (*58*). See Table S5 for a list of plasmids and information on donor and acceptor vectors for Golden Gate assemblies. The chloramphenicol (Cm) resistance gene was amplified from pACYC184 (New England BioLabs, Ipswich, MA, USA) and subcloned into pCR-Blunt II-TOPO. The HyperSpac promoter was amplified from pMF37/pDGICZ and fused to the Cm resistance gene using Gibson assembly. As a common acceptor plasmid, pFusA with a placeholder kanamycin resistance cassette, the chloramphenicol resistance cassette and a common downstream homology was assembled. Subcloned Golden Gate pieces and the final assemblies were introduced into *E. coli* DH5α.

Bacillus transformation and screening

The transformation protocol was obtained from http://2012.igem.org. 3 mL of freshly prepared medium A (25% glucose 1 mL, 10x medium A (yeast extract 5 g, casamino acids 1g, ddH$_2$O add 450 mL) 4 mL, 10× salt solution $(NH_4)_2SO_4$ 10 g, K_2HPO_4 69.8 g, KH_2PO_4 30g, Na^+ citrate 5 g, $MgSO_4$·7 H_2O 1 g, ddH$_2$O add 500 mL) 4.5 mL, H_2O added to 45 mL) were inoculated with a single colony from a fresh plate and incubated at 37 °C and 180 rpm overnight. 20 mL of freshly prepared and pre-warmed (37 °C) medium A was inoculated to an OD_{600} of 0.1 with the overnight culture. Cells were grown to an OD_{600} of 1.0 at 37 °C and 180 rpm. 45 mL of prewarmed freshly prepared medium B (25% glucose 1 mL, 10x medium A, 4 mL, 10x salt solution 4.5 mL, H_2O added to 45 mL, 1 M $CaCl_2$ 22.5 µl, 1 µM $MgCl_2$ 112.5 µl, ddH$_2$O added to 45 mL) were inoculated with 5 mL of the culture and incubated for 1.5 h at 37 °C and 180 rpm. Cells were harvested by centrifugation for 5 min at 1800 × g at 25 °C and resuspended in 3 mL of supernatant. 1 µg of DNA was added to 100 µl of cell suspension. After 2 h of incubation at 37 °C and 900 rpm cells were plated on LB containing 5 mg/mL of Cm or 60 mg/mL Spc, depending on the transformed construct. Plates were incubated overnight at 37 °C. Single colonies were picked and transferred to a fresh LB plate containing appropriate antibiotics and incubated overnight at 37 °C. If a mutant already containing one of the selection markers was transformed, colonies were plated on both antibiotics separately for counter selection screening. Positive colonies were chosen for colony PCR. *Bacillus* colony material was incubated at 100 °C for 10 min in 20 µl of DMSO and used as a template. An overview of screening primers is provided in Table S8.

Expression and analysis of chimeric *Bacillus* PKSs

To test for polyketide production, 5 mL YEME7 medium (4 g/L yeast extract, 10 g/L malt extract, 4 g/L glucose, 0.5 mL of 1 M NaOH per liter, add MOPS after autoclaving)(*30*) with the appropriate antibiotics was inoculated with a single colony of *B. subtilis* strains. The culture was incubated in the dark at 37 °C and 250 rpm for 16-18 h. To check for bacillaene production or intermediates, the culture was mixed in the dark with methanol and shaken vigorously. The samples were spun down for 20 min at 10 °C and max speed and the supernatant subjected to UHPLC-HRMS analysis. Kinetex 2.6 µm XB-C18 100 A column (4.6 × 150 mm; Phenomenex) for 5 min at 10% acetonitrile (0.1% FA) in water (0.1% FA) followed by a gradient (10 to 100%) of

acetonitrile (0.1% FA) in water (0.1% FA) over 11.5 min followed by 2 min 10% acetonitrile (0.1% FA) in water (0.1% FA) (1 mL/min).

Generation of *S. plymuthica* 4Rx13 Δ*oocQR* knockouts

The *S. plymuthica* 4Rx13 Δ*oocR* and *S. plymuthica* 4Rx13 Δ*oocQR* knockout strains were generated as described in Domik et al. (*59*), using the primer pairs KO_SOD_b01030_fwd/ KO_SOD_c22970_rev and KO_SOD_b01030_fwd/ KO_SOD_b01030_rev for *S. plymuthica* 4Rx13Δ*oocQR* and *S. plymuthica* 4Rx13Δ*oocR* respectively (Table S8).

Cloning of plasmids encoding chimeric *ooc* PKSs

The sequences encoding $OocQR_{KS0\text{-}11\_to\_KS12}$ and $OocS_{ACP\_C}$ were amplified from *S. plymuthica* 4Rx13 liquid culture and the sequences encoding grafted PKS fragments were amplified from *Gynuella sunshinyii* YC6258 liquid culture (lacunalide (*51*), lobatamide (*18*), gynuellalide (*26*) and tartrolon (*20*)), previously isolated metagenomic DNA of *Mycale hentscheli* (peloruside (*61*)), *Bacillus subtilis* DK1042 liquid culture (bacillaene(*30*, *48*)), or a previously isolated pCC1Fos fosmid (psymberin(*21*)). The amplicon of the *psy* terminus from the NAHVILEE motif onward was synthesized by GenScript (Leiden, The Netherlands). An overview of the primers used is provided in Table S9. pBAD was used as backbone and the individual constructs were assembled using extension PCRs, Gibson assembly or ExoCET (*61*). Assembled constructs were introduced into *E. coli* DH5α, verified by sequencing, and then introduced into *S. plymuthica* 4Rx13 Δ*oocQR*. As controls, empty *pBAD* was introduced into *S. plymuthica* 4Rx13 Δ*oocQR*.

Expression and analysis of *S. plymuthica* 4Rx13 strains

For MS-based analysis of *S. plymuthica* 4Rx13 chimeras, the strains were inoculated from overnight cultures and grown in 20 mL enriched potato dextrose broth (EPB: 24 g/L potato dextrose broth (Difco), 6 g/L bactopeptone, 4 g/L yeast extract, 100 mg/L NaCl). The volumes of overnight culture used for inoculation were adjusted to reflect a 200 µL culture with a 5x-diluted OD_{600}=0.5. Cultures were supplemented with appropriate antibiotics. For wild type *S. plymuthica*, no antibiotics were used, for *S. plymuthica* Δ*oocQR* 25 µg/mL kanamycin was used and for *S. plymuthica* Δ*oocQR* strains supplemented with plasmids, 100 µg/mL ampicillin and 25 µg/mL kanamycin was used. Expression of the introduced PKS parts was induced by the addition of 0.2% L-arabinose (2 µL/mL 1 g/mL). Then, cultures were incubated for 20 hours at 26 °C and 180 rpm. We note expression at higher or lower temperatures leads to a significant drop and near-abolishment in production. After 20 hours, 7 mL of the cultures were extracted with 7 mL ethyl acetate. 4 mL of the organic phase was evaporated over a nitrogen flow while being heated to 40 °C, after which the solid residue was resuspended in 1 mL methanol and centrifuged. The supernatant was subjected to UHPLC-HRMS analysis using a Dionex Ultimate 3000 UPLC system connected to a Thermo QExactive mass spectrometer. A solvent gradient (A = H_2O + 0.1% formic acid and B = acetonitrile + 0.1% formic acid) with B at 1% for 0–3 min, 5–95% for 3–19 min and 95% for 19–23 min at a flow rate of 1.0 mL/min) was used on a

Phenomenex Kinetex 2.6 µM C18 100A (150 × 4.6 mm) column at 27 °C. The MS was operated in positive ionization mode at a scan range of 150–1500 *m/z*. The spray voltage was set to 3.7 kV and the capillary temperature to 320 °C. Analysis of MS data was done with 5 ppm accuracy. Downstream data analysis was performed with custom Python scripts to obtain the EICs reported for the *S. plymuthica* mutants in which all peaks reported have a chlorinated isotope pattern. Peaks at *m/z* values corresponding to the target mass for the EICs but that did not show chlorination isotope patterns were excluded.

Isolation of compounds 2 and 3

Expression for purification of **2** and **3** was done under the same conditions as expressions for analysis of all *S. plymuthica* mutants, as described directly above. For the expression of **2**, 5 liter liquid cultures of were grown in batches of 200 mL in 1 L baffled Erlenmeyer flasks. For the expression and purification of **3**, 10 liter liquid cultures of *S. plymuthica* 4Rx13 Δ*oocQR* + *pBAD-oocQR*$_{\mathrm{LPTYPFx5W}}$-*Psy*$_{\mathrm{KS11}}$ were grown in batches of 100 mL in 250 mL baffled Erlenmeyer flasks. The pellet and supernatant from the total culture volumes were separated via centrifugation. The supernatant was extracted twice with equal volumes of ethyl acetate. The resulting ethyl acetate extracts were evaporated to dryness, leaving a brown solid. The supernatant was extracted two times with equal volumes of ethyl acetate and evaporated to dryness.

The crude extracts were fractionated using preparative reverse phase HPLC (Agilent 1260 Infinity system, equipped with a Phenomenex Luna 5 µm C18 21.2 x 250 mm column). Deionized water (Milli-Q, Millipore) +0.05% TFA (solvent A) and acetonitrile +0.05% TFA (solvent B) were used as the mobile phase. An elution gradient of 5–100% solvent B in 50 min followed by isocratic conditions at 100% solvent B for 10 min was applied. UV detection was carried out at 210, 254, and 280 nm. The collected fractions were analyzed by HR-MS.

For the isolation of compound **2**, the crude extracts were fractionated by semi-preparative reverse phase HPLC (Agilent 1260 Infinity system, equipped with a Phenomenex Kinetex 5 µm C18 10 x 250 mm column) with solvent A/solvent B above as the mobile phase. An elution gradient of 5–50% solvent B in 15 minutes followed by followed by a steady increase of solvent B from 50-100% in 25 minutes and finally isocratic conditions at 100% solvent B for 10 min was applied. UV detection was carried out at 210, 254, and 280 nm and two fractions collected every minute. The fractions containing **2** were combined to afford 3 mg of the compound.

Fractions containing **3** were further purified by semi-preparative reverse phase HPLC (Agilent 1260 Infinity system, equipped with a Phenomenex Kinetex 5 µm C18 10 x 250 mm column) with solvent A/solvent B above as the mobile phase. Compound **3** was obtained by purification of fraction 12 and 13 by applying an elution gradient 25–65% solvent B for 30 min followed by gradient shift from 65 to 100% in 5 min, and finally isocratic condition at 100% solvent B for 5 min.

Construction of module deletion plasmids (pEB17_Δ*lcn*14-15, pEB17_Δ*lcn*17-24, pEB17_Δ*lcn*20-23, pEB17_Δ*lcn*21-22)

Primers for Gibson assemblies were designed using NEBuilder (https://nebuilder.neb.com/#!/) to generate products with 20 bp overhang to one another. For each deletion plasmid, the two 500 bp homology arms were chosen in a way that the first homology arm ended at the protein level downstream of the FSD after the respective KS (e.g., KS13 in pEB17_Δ*lcn*14-15), and the second homology arm started after the FSD of the last deleted KS (e.g., KS15 in pEB17_Δ*lcn*14-15; hence enclosing the 8168 bp of modules 14 and 15).

To amplify target regions, a small *G. sunshinyii* culture was transferred to a 1.5 mL Eppendorf tube, sonicated for 10 min and then boiled at 100 °C for 10 min. The supernatant was used as a template in the PCR (for primer pairs see Table S10, eg., GS03&GS04 to amplify the Δlcn1415 homology arm 1). Purified plasmids were used as a template for the plasmid backbone (pEB17, eg. GS01&GS02 to amplify the Δ*lcn*14-15 backbone). PCR products were separated on an agarose gel and purified with a NucleoSpin Gel and PCR Clean-up kit (Macherey-Nagel AG). The homology arms and respective inserts were fused by an overlap extension PCR (outer primers of the fused product were only added after 15 PCR steps). After Gibson assembly of the fragments using in-house prepared master mixes (*62*), plasmids were electroporated into *E. coli* DH5α pir recovered in Luria-Bertani (LB) broth for one hour and plated onto LB containing kanamycin (50 µg/mL). A culture of a single colony was prepared, the plasmid was isolated with a NucleoSpin Plasmid kit (Macherey-Nagel AG) and sequenced (primers GS25, GS26, Table S10).

Transformation of *E. coli* ST18 donor strains

The plasmids (pEB17_Δ*lcn*14-15, pEB17_Δ*lcn*17-24, pEB17_Δ*lcn*20-23, pEB17_Δ*lcn*21-22) were electroporated into the auxotrophic donor strain *E. coli* ST18 (*63*). Bacteria were recovered in LB supplemented with 50 µg/mL 5-aminolevulinic acid (ALA). After 1-hour, cultures were plated onto LB (50 µg/mL ALA, 50 µg/mL kanamycin). For the conjugation, a culture of a single colony was prepared.

Construction of *G. sunshinyii* mutants by conjugation and homologous recombination

The following *G. sunshinyii* mutants were constructed using the same protocol: pEB17_Δ*lcn*14-15, pEB17_Δ*lcn*17-24, pEB17_Δ*lcn*20-23, pEB17_Δ*lcn*21-22, pEB17_Δ*lcn*14-15_17-24, pEB17_Δ*lcn*14-15_20-23, pEB17_Δ*lcn*14-15_21-22. Table S12 lists the donor and acceptor strains used for the construction of each mutant. Cultures of donor strain (*E. coli* ST18 carrying one of the plasmids in Table S11) were prepared in LB (50 µg/mL ALA, 50 µg/mL kanamycin). Cultures of acceptor strain (wild type or *G. sunshinyii* YC6258 Δ*lcn*14-15; Table S12) were prepared in marine broth (½ MB). The next day, 10 mL of *E. coli* cultures and 50 mL of *G. sunshinyii* cultures were harvested in falcon tubes by centrifugation. The cells were resuspended in 10 mL of the respective medium (LB or ½ MB), the optical density at 600 nm (OD_{600}) was measured and, after another centrifugation step, the cells were resuspended in the

respective medium to yield a suspension of OD_{600} = 4. Donor and acceptor strain were mixed in three different ratios (1:9, 3:7, 1:1) to a total volume of 1 mL. After centrifugation, 800 µL of the supernatant was discarded, the remaining volume was used to resuspend the cell mixtures. A plate (½ MB, 50 µg/mL ALA) was separated into three segments and each of the three different ratios were placed in a single spot in one of the segments. After one day of growth at 37 °C, half of each of the three spots was combined and the combined culture was resuspended in 500 µL 0.9% (w/v) NaCl solution, of which 200 µL was plated onto ½ marine broth agar (MB (5 g bacteriological peptone, 1 g yeast extract, 16.5 g instant ocean sea salt, 15 g agar), 50 µg/mL kanamycin) and distributed using a cell spreader. Single colonies were isolated and propagated to a liquid culture (½ MB, 50 µg/mL kanamycin), and the genomic integration of the suicide plasmid was confirmed by PCR with two primer pairs (one primer binds in the plasmid after the homology arms, facing upstream and one in the genome upstream the integration site, facing downstream, or the other way around; eg. primers GS25&GS28 and GS26&GS27 for *G. sunshinyii* YC6258 Δ*lcn*14-15, Table S10). Then, 5 mL of ½ MB without any additives was inoculated with 100 µL of the *G. sunshinyii* culture and cultivated for one day at 30 °C. The next day, 200 µL of this culture was plated onto ½ MB containing 10% (w/v) sucrose. After two days of growth at 30 °C, about 30 colonies were picked using a toothpick and first a plate of ½ MB (50 µg/mL kanamycin) then a plate of ½ MB was inoculated for each colony. Colonies that grew on antibiotics were considered false positives and 10 colonies that only grew on ½ MB were propagated into liquid culture (½ MB). Genetic modification was confirmed using primers that bind up- and downstream of the modification site (Fig. S53, Table S10, eg. primers GS27&GS28 for *G. sunshinyii* YC6258 Δ*lcn*14-15). PCR products were separated by agarose gel electrophoresis, purified with a NucleoSpin Gel and PCR Clean-up kit (Macherey-Nagel AG) and sequenced.

HPLC-HRMS analysis of *G. sunshinyii* mutants

The following *G. sunshinyii* mutants were analyzed using the same protocol: wild type, pEB17_Δ*lcn*14-15, pEB17_Δ*lcn*17-24, pEB17_Δ*lcn*20-23, pEB17_Δ*lcn*2-122, pEB17_Δ*lcn*14-15_17-24, pEB17_Δ*lcn*14-15_20-23, pEB17_Δ*lcn*14-15_21-22. Bacteria were cultivated in 20 mL ½ MB supplemented with 0.25% (w/v) arabinose in 100 mL Erlenmeyer flasks. After four days of growth at 30 °C and 180 rpm, 10 mL of the supernatant were extracted with 10 mL ethyl acetate and the organic phase was dried. The residue was dissolved in 300 µL acetonitrile and analyzed by HPLC-HRMS (Figure 5, Figure S55-62).

Isolation of engineered lacunalides 8, 9, 10, 11, 12, 14, 16, 18, 20, and 21

For each mutant of *G. sunshinyii* YC6258, a total of 6 L liquid culture (see Table S17) was grown in batches of 300 mL MB broth with 0.25% (w/v) arabinose in 1 L Erlenmeyer flasks at 30 °C for 3 days on an orbital shaker (150 rpm). The cultures were centrifuged, and each supernatant was extracted three times with ethyl acetate. The cell pellets were resuspended in H_2O and extracted with acetone. The ethyl acetate and acetone extract were dried and combined. The combined crude extract was separated by RP-HPLC (Phenomenex Luna 5µ C18, φ 20 x 250 mm,

15.0 mL/min, 260 nm) with MeCN in H_2O + 0.1% formic acid as mobile phase, starting from isocratic 5% MeCN for 5 min, gradient from 5% to 95% MeCN for 32 min, and isocratic elution 95% MeCN for 10 min to afford 40 fractions. The fractions at a retention time (t_R) of 26 to 30 minutes were combined and further purified by semi preparative HPLC (Phenomenex Luna 5µ Phenyl-Hexyl, φ 10 x 250 mm, 2.0 mL/min, 260 nm) with an isocratic 37% MeCN + 0.1% formic acid elution to afford 4.2 mg of **8**, at t_R 25 min. Peaks at a t_R of 21 to 23 minutes were combined and purified by semi preparative HPLC (Phenomenex Synergi 4µ Hydro-RP, φ 10 x 250 mm, 2.0 mL/min, 260 nm) with an isocratic 40% MeCN + 0.1% formic acid elution to obtain **9**, t_R 33.4 min. Compounds **10**, **11**, **12**, **14**, **16**, **18**, **20**, and **21** were isolated using a similar workflow, for details see Table S17.

Structure elucidation of compound 8

Compound **8** was assigned the molecular formula $C_{50}H_{90}O_{13}$ with six degrees of unsaturation based on the deduction from the HR-MS data (*m/z* 899.6520 $[M+H]^+$, Δ 6.55 mmu; Figure S65). ^1H NMR in conjunction with HSQC and ^{13}C data (Figure S66-72) suggested three aliphatic doublet methyls, two vinylic singlet methyls, 22 methylene groups, six protons connected to sp^2 carbons, 12 methanetriyl groups connected to oxygen, and two aliphatic methanetriyl groups. The HMBC spectrum in conjunction with ^{13}C data revealed three quaternary carbons accounting for all 50 carbons suggested by the molecular formula.

HMBC correlations from each of the vinylic singlet methyls to a quaternary carbon in combination with six protons connected to sp^2 carbons suggested four double bonds, which were all assigned as *trans* based on the high coupling constants ($^3J_{C2\text{-}C3}$ = 15.7 Hz, $^3J_{C34\text{-}C35}$ = 15.3 Hz) and the upfield carbon shifts of C-47 and C-49 (δ 12.6 and 11.5 ppm). HMBC correlations from two directions to a downfield quaternary carbon at δ 168.9 ppm suggest a macrolactone, accounting for all six degrees of unsaturation.

From the COSY spectrum, six spin systems were identified (Figure S68 and S72), of which systems **a**/**b**/**e** and **c/d** were connected by HMBC correlations. The remaining three methanetriyl groups connected to oxygen and the 12 methylene groups could not be assigned due to overlapping signals. A high similarity of chemical shifts (Figure S71 and S79, Table S18) suggests a very similar structure to lacunalide A (**6**)(*51*).

Structure elucidation of compound 9

Compound **9** was assigned the molecular formula $C_{49}H_{88}O_{13}$ with six degrees of unsaturation based on the deduction from the HR-MS data (*m/z* 885.6359 $[M+H]^+$, Δ 6.13 mmu; Figure S73). ^1H NMR in conjunction with HSQC and ^{13}C data (Figure S74-78) suggested three aliphatic doublet methyls, one vinylic singlet methyl, >20 methylene groups, seven protons connected to sp^2 carbons, 12 methanetriyl groups connected to oxygen, and two aliphatic methanetriyl groups. The HMBC spectrum in conjunction with ^{13}C data revealed two quaternary carbons accounting for more than 45 carbons of the suggested molecular formula.

HMBC correlations from each of the vinylic singlet methyls to a quaternary carbon in combination with six protons connected to sp^2 carbons suggested four double bonds, which were all assigned as *trans* based on the high coupling constants ($^3J_{C2\text{-}C3}$ = 15.7 Hz, $^3J_{C20\text{-}C21}$ = 15.5 Hz, $^3J_{C34\text{-}C35}$ = 15.2 Hz) and the upfield carbon shift of C-47 (δ 12.6 ppm).HMBC correlations from two directions to a downfield quaternary carbon at δ 168.9 ppm suggest a macrolactone, accounting for all six degrees of unsaturation.

From the COSY spectrum, five spin systems were identified (Figure S76 and S72), of which systems **a**/**b**/**d** were connected by HMBC correlations. The remaining three methanetriyl groups connected to oxygen and the >10 methylene groups could not be assigned due to overlapping signals. A high similarity of chemical shifts (Figure S79, Table S18) suggests a very similar structure to lacunalide A (**6**).

Structure elucidation of compound 10 and 11

Compounds **10** and **11** could not be separated by several rounds of HPLC purification (see Table S17, and Figure 5). The structures were elucidated using the mixture. **10** was assigned the molecular formula $C_{50}H_{90}O_{12}$ with six degrees of unsaturation based on the deduction from the HR-MS data (*m/z* 883.6490 $[M+H]^+$, Δ -1.51 mmu; Figure S81). **11** was assigned the molecular formular $C_{49}H_{88}O_{12}$ with six degrees of unsaturation based on the deduction from the HR-MS data (*m/z* 869.6346 $[M+H]^+$, Δ -0.25 mmu; Figure S81). ^{1}H NMR in conjunction with HSQC and ^{13}C data of the **10** and **11** mixture (Figure S82, S83, S86) suggested for **10** three aliphatic doublet methyls, two vinylic singlet methyls, more than 23 methylene groups, six protons connected to sp^2 carbons, ten methanetriyl groups connected to oxygens (with a normalized integral of 100% or ~80%), and two aliphatic methanetriyl group. The HMBC spectrum in conjunction with ^{13}C data revealed three quaternary carbons accounting for all 50 carbons suggested by the molecular formula. For **11** two additional protons connected to sp^2 carbons (integrals of ~20% compared to other signals), two methanetriyl groups connected to oxygens (integrals of ~20% compared to other signals), and one aliphatic methanetriyl group were detected.

HMBC correlations (Figure S85) from the vinylic singlet methyls to quaternary carbons in combination with six protons connected to sp^2 carbons suggested four double bonds in **10** and an additional in **11**, which were all assigned as *trans* based on the high coupling constants ($^3J_{C2\text{-}C3}$ = 15.7 Hz, $^3J_{C34\text{-}C35}$ = 15.2 Hz, $^3J_{C16\text{-}C17}$ = 15.5 Hz for **11**) and the upfield carbon shift of C-47 (δ 12.7 ppm) and C-49 (δ 11.8 ppm for **10**). HMBC correlations from two directions to a downfield quaternary carbon at δ 168.9 ppm suggest a macrolactone, accounting for all six degrees of unsaturation.

From the COSY spectrum, ten spin systems were identified (Figure S80 and S84), of which systems **a**/**b**/**c**/**d**/**g** were connected by HMBC correlations. The remaining two methanetriyl groups connected to oxygen and the ten methylene groups could not be assigned due to overlapping signals. Despite obtaining **10** and **11** as a mixture, the C-13 to C-20 region of the

compounds could be differentiated clearly. A high similarity of chemical shifts (Figure S79, Table S18) suggests a very similar structure to lacunalide A (**6**).

Structure elucidation of compound 12

Compound **12** was assigned the molecular formula $C_{46}H_{82}O_{10}$ with six degrees of unsaturation based on the deduction from the HR-MS data (*m/z* 795.5974 $[M+H]^+$, Δ -0.68 mmu; Figure S88). ^1H NMR in conjunction with HSQC and ^{13}C data (Figure S89, S90. S93) suggested three aliphatic doublet methyls, two vinylic singlet methyl, 21 methylene groups, six protons connected to sp^2 carbons, nine methanetriyl groups connected to oxygen, and two aliphatic methanetriyl group. The HMBC spectrum in conjunction with ^{13}C data revealed three quaternary carbons accounting for all 46 carbons suggested by the molecular formula.

HMBC correlations from the vinylic singlet methyls to a quaternary carbons in combination with six protons connected to sp^2 carbons suggested four double bonds, which were all assigned as *trans* based on the high coupling constants ($^3J_{C2\text{-}C3}$ = 15.7 Hz, $^3J_{C30\text{-}C31}$ = 15.3 Hz) and the upfield carbon shift of C-43 (δ 12.7 ppm) and C-45 (δ 12.0 ppm). HMBC correlations from two directions to a downfield quaternary carbon at δ 168.9 ppm suggest a macrolactone, accounting for all six degrees of unsaturation.

From the COSY spectrum, seven spin systems were identified (Figure S 87 and S91), of which systems **a**/**b**/**c**/**f** were connected by HMBC correlations. The remaining two methanetriyl groups connected to oxygen and the 11 methylene groups could not be assigned due to overlapping signals. A high similarity of chemical shifts (Figure S79, Table S18) suggests a very similar structure to lacunalide A (**6**)

Structure elucidation of compound 14

Compound **14** was assigned the molecular formula $C_{36}H_{65}O_7$ with five degrees of unsaturation based on the deduction from the HR-MS data (*m/z* 609.4716 $[M+H]^+$, Δ -0.88 mmu; Figure S95). ^1H NMR in conjunction with HSQC and ^{13}C data (Figure S96, S97, S100) suggested two aliphatic doublet methyls, one vinylic singlet methyl, 19 methylene groups, five protons connected to sp^2 carbons, six methanetriyl groups connected to oxygen, and one aliphatic methanetriyl group. The HMBC spectrum in conjunction with ^{13}C data revealed two quaternary carbons accounting for all 36 carbons suggested by the molecular formula.

HMBC correlations from the vinylic singlet methyl to a quaternary carbon in combination with five protons connected to sp^2 carbons suggested three double bonds, which were all assigned as *trans* based on the high coupling constants ($^3J_{C2\text{-}C3}$ = 15.7 Hz, $^3J_{C22\text{-}C23}$ = 15.3 Hz) and the upfield carbon shift of C-35 (δ 12.5 ppm). HMBC correlations from two directions to a downfield quaternary carbon at δ 168.7 ppm suggest a macrolactone, accounting for all five degrees of unsaturation.

From the COSY spectrum, six spin systems were identified (Figure S94 and 98), of which systems **a**/**b**/**e** were connected by HMBC correlations. The remaining two methanetriyl groups

connected to oxygen and the ten methylene groups could not be assigned due to overlapping signals. A high similarity of chemical shifts (Figure S79, Table S18) suggests a very similar structure to lacunalide A (**6**).

Structure elucidation of compound 16

Compound **16** was assigned the molecular formula $C_{46}H_{82}O_{11}$ with six degrees of unsaturation based on the deduction from the HR-MS data (*m/z* 811.5905 $[M+H]^+$, Δ -2.49 mmu; Figure S102). ^{1}H NMR in conjunction with HSQC and ^{13}C data (Figure S103, S104. S107) suggested three aliphatic doublet methyls, two vinylic singlet methyl, 21 methylene groups, six protons connected to sp^2 carbons, nine methanetriyl groups connected to oxygen, and two aliphatic methanetriyl group. The HMBC spectrum in conjunction with ^{13}C data revealed three quaternary carbons accounting for all 46 carbons suggested by the molecular formula.

HMBC correlations from the vinylic singlet methyls to a quaternary carbons in combination with six protons connected to sp^2 carbons suggested four double bonds, which were all assigned as *trans* based on the high coupling constants ($^3J_{C2\text{-}C3}$ = 15.7 Hz, $^3J_{C30\text{-}C31}$ = 15.3 Hz) and the upfield carbon shift of C-43 (δ 12.7 ppm) and C-45 (δ 11.5 ppm). HMBC correlations from two directions to a downfield quaternary carbon at δ 168.9 ppm suggest a macrolactone, accounting for all six degrees of unsaturation.

From the COSY spectrum, seven spin systems were identified (Figure S 101 and S105), of which systems **a**/**b**/**c**/**d**/**f** were connected by HMBC correlations. The remaining methanetriyl group connected to oxygen and the nine methylene groups could not be assigned due to overlapping signals. A high similarity of chemical shifts (Figure S79, Table S18) suggests a very similar structure to lacunalide A (**6**)

Structure elucidation of compound 18

Compound **18** was assigned the molecular formula $C_{32}H_{56}O_6$ with five degrees of unsaturation based on the deduction from the HR-MS data (*m/z* 537.4141 $[M+H]^+$, Δ -1.41 mmu; Figure S109). ^{1}H NMR in conjunction with HSQC and ^{13}C data (Figure S110, S111, S114) suggested two aliphatic doublet methyls, one vinylic singlet methyl, more than 15 methylene groups, five protons connected to sp^2 carbons, six methanetriyl groups connected to oxygen, and one aliphatic methanetriyl group. The HMBC spectrum in conjunction with ^{13}C data revealed two quaternary carbons accounting for 33 carbons, one more than suggested by the molecular formula. In particular, the NMR signals of six methanetriyl groups connected to oxygens and one carboxylate ester account for a total of seven oxygens. For the observed mass, the closest molecular formular containing seven oxygens and a similar degree of unsaturation (which is based on the partially assigned structure a conclusive assumption; see below) is $C_{31}H_{52}O_7$ (*m/z* 537.4141 $[M+H]^+$, Δ 34.97 mmu), and hence highly improbable. The six NMR-detectable methanetriyl groups connected to oxygens might arise from isomers that were not resolvable or from an impurity.

HMBC correlations from the vinylic singlet methyl to a quaternary carbon in combination with five protons connected to sp^2 carbons suggested three double bonds, which were all assigned as *trans* based on the high coupling constants ($^3J_{C2-C3}$ = 15.7 Hz, $^3J_{C18-C19}$ = 15.2 Hz) and the upfield carbon shift of C-31 (δ 12.3 ppm). HMBC correlations from two directions to a downfield quaternary carbon at δ 168.7 ppm suggest a macrolactone, accounting for all five degrees of unsaturation.

From the COSY spectrum, six spin systems were identified (Figure S108 and S112), of which systems **a**/**b**/**e** were connected by HMBC correlations. The remaining methanetriyl group connected to oxygen and the eight methylene groups could not be assigned due to overlapping signals. A high similarity of chemical shifts (Figure S79, Table S18) suggests a very similar structure to lacunalide A (**6**).

Structure elucidation of compound 20 and 21

Compounds **20** and **21** could not be separated by several rounds of HPLC purification (see Table S17, and Figure 5). The structures were elucidated using the mixture. **20** was assigned the molecular formula $C_{42}H_{74}O_9$ with six degrees of unsaturation based on the deduction from the HR-MS data (*m/z* 723.5389 $[M+H]^+$, Δ -1.66 mmu; Figure S116). **21** was assigned the molecular formular $C_{41}H_{72}O_9$ with six degrees of unsaturation based on the deduction from the HR-MS data (*m/z* 709.5236 $[M+H]^+$, Δ -1.31 mmu; Figure S116). ^{1}H NMR in conjunction with HSQC and ^{13}C data of the **20** and **21** mixture (Figure S117, S118, S121) suggested for **20** three aliphatic doublet methyls, two vinylic singlet methyls, more than 18 methylene groups, six protons connected to sp^2 carbons, eight methanetriyl groups connected to oxygens (with a normalized integral of 100% or ~70%), and two aliphatic methanetriyl group. The HMBC spectrum in conjunction with ^{13}C data revealed three quaternary carbons accounting for all 42 carbons suggested by the molecular formula. For **21** two additional protons connected to sp^2 carbons (integrals of ~30% compared to other signals), two methanetriyl groups connected to oxygens (integrals of ~30% compared to other signals), and one aliphatic methanetriyl group were detected.

HMBC correlations (Figure S120) from the vinylic singlet methyls to quaternary carbons in combination with six protons connected to sp^2 carbons suggested four double bonds in **20** and an additional in **21**, which were all assigned as *trans* based on the high coupling constants ($^3J_{C2-C3}$ = 15.7 Hz, $^3J_{C26-C27}$ = 15.2 Hz, $^3J_{C12-C13}$ = 15.5 Hz for **21**) and the upfield carbon shift of C-39 (δ 12.6 ppm) and C-41 (δ 11.6 ppm for **20**). HMBC correlations from two directions to a downfield quaternary carbon at δ 169.0 ppm suggest a macrolactone, accounting for all six degrees of unsaturation.

From the COSY spectrum, seven spin systems were identified (Figure S115 and S119), of which systems **a**/**b**/**c**/**e** were connected by HMBC correlations. The remaining methanetriyl group connected to oxygen and the nine methylene groups could not be assigned due to overlapping signals. Despite obtaining **20** and **21** as a mixture, the C-9 to C-15 region of the compounds

could be differentiated clearly. A high similarity of chemical shifts (Figure S79, Table S18) suggests a very similar structure to lacunalide A (**6**).

Bioactivity tests of isolated compounds (8, 9, 10+11, 12, 14, 16, 18, 20+21) against HeLa cells

HeLa cells purchased from ATCC were cultivated at 37 °C, 5% (v/v) CO_2 for 3-4 days. Cells were washed with PBS buffer (Sigma D8537), 0.05 % trypsin-EDTA solution (Thermo 25300-054) was added and the plate was incubated for 5 min at 37 °C. The cells were resuspended in 5 mL medium (DMEM-GlutaMAX) supplemented with 10% FCS (Eurobio CVFSVF00-01), and 50 µg/mL penicillin-streptomycin (Corning). After counting the cells under a ZEISS Axiovert 25 microscope using a Neubauer hemocytometer a 10,000 cells/mL suspension was prepared and 200 µL were transferred into each well of three 96-well plates. After one day of cultivation, 2 µL of compounds (**8**, **9**, **10**+**11**, **12**, **14**, **16**, **18**, **20**+**21** at a concentration of 10 mM), DMSO as a negative control and doxorubicin (1 mg/mL) were added to row B of the plate. 50 µL media of row A was added and a 5-fold serial dilution was performed to row G. After three days of cultivation, 50 µL of 3-(4,5-dimethylthiazol-2-yl)-2,5-diphenyltetrazolium bromide (MTT, 1 mg/mL in sterile H_2O) were added and the cells incubated for 3 h at 37 °C. The supernatant was discarded and then 150 µL of DMSO were added to the wells. The absorbance at 570 nm was measured on a spectraMAXplus spectrometer (Molecular Devices LLC). Results are shown in Figure S122.

Bioinformatic procedures

Sequence alignments of KS sequences

Amino acid sequences of 821 KS with and without downstream adapter regions were extracted from an in-house database of 88 annotated *trans*-AT PKS clusters. As an outgroup, KS3 and KS5 from the erythromycin *cis*-AT PKS were used. The sequences were aligned using the MUSCLE algorithm with default settings (*53*) and a phylogenetic tree was computed with FastTree (version 2.1.10 +SSE3 +OpenMP, 16 threads, default settings) (*64*). A sequence logo was created using WebLogo (*65*).

Computational details on the statistical coupling analysis

The sequences of BGCs annotated as *trans*-AT PKS in the antiSMASH database(*37*) and an in-house database of *trans*-AT PKS assembly lines (Table S1) were used in the statistical analysis. 2239 *trans*-AT PKS BGC deposited in the antiSMASH database and an in-house database were extracted. A list of accession numbers of the genomes containing the BGCs obtaining from the antiSMASH database are enclosed in the data repository. An overview of the number of domain motifs is provided in Figure S10. Extraction of the tridomain sequences yielded 1194 amino acid sequences extracted from 516 clusters. From these sequences, the amino acid sequences indicated by the antiSMASH annotation of the domains, including 100 leading and trailing amino acids in multidomain sequences, while taking gene termini into account. To ensure that

sequences annotated with any of antiSMASH's various carrier protein annotations are included in the sequence extractions., With this, any of the PP-binding, ACP, ACP_beta AMP_binding, PCP and PKS_PP annotations were accepted as PP-binding target domain and these sequences were extracted in the presence of appropriate neighboring domains. To prevent large, highly gapped regions in MSAs of multidomain sequences, 15 amino acids following and leading consecutive domain annotations were extracted, instead of the full sequence linking the domains. The sequences obtained were then used to construct a multiple sequence alignment (MSA). MSAs with Clustal-Omega (*66*, *67*) were constructed using the '-threads=5 -seqtype=Protein' options. The MSAs were then used for statistical coupling analysis (SCA) using Python scripts published by Rivoire et al (*32*). First, positions that contained more than 80% gaps and subsequently sequences containing more than 20% gaps were removed from the MSA, and positions that contained more than 20% gaps were filtered from the alignment. Then, the sequences were weighed by the inverse of the total number of sequences in the MSA with which the sequence shares more than 80% sequence identity. The obtained filtered MSA and sequence weights were used to perform the statistical coupling analysis. Full details of SCA algorithm can be found elsewhere (*32*). The final, filtered MSA contained 1284 sequences and 970 amino acid positions. Taking sequence weights into account (*32*), 428 effective sequences were used. The SCA matrices for the various domain motifs are given below. The EPIAII, HGTGT, NAHxVxE and TYPFx$_5$W motifs are indicated on the axes, indicating the *N*-terminus, active site, and *C*-terminus of the KS domain and the *C*-terminus of the FSD, respectively. Since the NAHxVxE motif is not as highly conserved as many of the other motifs, the exact sequence of the motif in the consensus sequence varies slightly between the alignments (Figures S10-13).

Table S1 Sources for the *trans*-AT BGC sequences not included in the antiSMASH database.

PKS	Reference	MiBIG accession	Genbank accession
9-Methylstreptimidone	(*68*)		
Albicidin		BGC0001088	
Alpiniamide	(*69*, *70*)		
Anthracimycin	(*71*, *72*)		
Apicularen			ASRX01000032.1
Aquimarina sp. RZ0 PKS			GCF_008370685.1
Aurantinin			NZ_LYMC00000000.1
Bacillaene (*B. amyloliquefaciens*)		BGC0001089	AJ634060.2
Bacillaene (*B. subtilis*)	(*73*)		
Basiliskamide		BGC0000172	
Bongkrekic acid		BGC0000173	
Bryostatin		BGC0000174	
Calyculin		BGC0000967	
Catenulispora acidiphila DSM 44928 PKS			NC_013131.1
Chivosazol		BGC0001069	
Chlorotonil	(*74*)		
Corallopyronin		BGC0001091	
Cuniculene	(*20*)		
Cycloheximide		BGC0000175	
Diaphorin	(*75*)		
Difficidin		BGC0000176	
Disorazole		BGC0001093	
Dorrigocin/Migrastatin		BGC0000177	
Elansolid		BGC0000178	
Enacyloxin		BGC0001094	
Etnangien		BGC0000179	
Geotalea uraniireducens Rf4 PKS			NC_009483.1
Gladiofungin		BGC0002083	
Griseoviridin		BGC0000459	
Gynuellalide		BGC0001835	
Inthomycin		BGC0002451	
Janustatin		BGC0002136	
Kalimantacin		BGC0001099	
Kirromycin		BGC0001070	
Labrenzin		BGC0002068	
Lactimidomycin		BGC0000083	
Lacunalide		BGC0001644	
Lagriamide		BGC0001646	
Lankacidin		BGC0001100	
Legioliulin		BGC0000180	
Leinamycin		BGC0001101	
Leptolyngbyalide		BGC0001837	

Table S1 continued.

Lobatamide		BGC0002046	
Luminaolide		BGC0001656	
Macrobrevin		BGC0001470	
Macrolactin		BGC0000181	
Malleilactone		BGC0001102	
Misakinolide		BGC0001186	
Mupirocin		BGC0000182	
Mycalamide		BGC0002055	
Mycale hentscheli PKS4	(*60*)		
Myxopyronin		BGC0001091	
Myxovirescin		BGC0001025	
Necroxime		BGC0002050	MN734804.1
NOCAP	(*76*)		
Nosperin		BGC0001071	
Onnamide		BGC0001032	
Oocydin		BGC0001032	
Oxazolomycin		BGC0001106	
Pateamide	(*60*, *77*)		
Patellazole		BGC0001107	CP006745.1
Azospirillum sp. B4 PKS			NZ_BACU01000416.1
Pederin		BGC0001108	
Peluroside		BGC0002056	
Phormidolide		BGC0001350	
Phthoxazolin		BGC0001740	
Pristinamycin		BGC0000952	
Psymberin		BGC0001110	
Pulvomycin		BGC0000186	
Pyxipyrrolone		BGC0001751	KY765914.1
Rhizopodin		BGC0001111	
Rhizoxins		BGC0001112	
Ripostatin		BGC0001761	
SIA7248	(*78*)		
Scytophycin		BGC0001772	KY767986.1
Sorangicin		BGC0000184	
Spliceostatin		BGC0001113	
Swinholide		BGC0001795	KY767987.1
Tartrolon		BGC0001836	
Thailandamide		BGC0000186	
Thailanstatin		BGC0001114	
Thiomarinol		BGC0001115	
Bacillus thuringiensis IEBC_T61001 PKS			NZ_FMBI01000020.1
Toblerol		BGC0001991	
Tolytoxin	(*22*)		
Virginiamycin		BGC0001116	
Serratia sp. 3ACOL1 PKS			NZ_CP033055.1

Supplementary tables

Table S2 Strains used in this study.

Strain	Characteristics
E. coli DH5α (Invitrogen)	F– Φ80*lacZ*ΔM15 Δ(*lacZYA-argF*) U169 *recA1 endA1 hsdR17* (rK–, mK+) *phoA supE44* λ– *thi*-1 *gyrA96 relA1*
E. coli BL21 Tuner™ (DE3, Novagen)	
S. plymuthica 4Rx13	NCBI accession number CP006250.1, NC_021591.1
S. plymuthica 4Rx13 Δ*oocR*	Δ^- *oocR*, Δ^+ Kan^R
S. plymuthica 4Rx13 Δ*oocQR*	Δ^- *oocQ, oocR*, Δ^+ Kan^R
G. sunshinyii YC6258	
G. sunshinyii YC6258 Δ*lcnBC*	Δ*lcnBC* modules 14 to 15
G. sunshinyii YC6258 Δ*lcnCDE*	Δ*lcnCDE* modules 17 to 24
G. sunshinyii YC6258 Δ*lcnCD*	Δ*lcnCD* modules 20 to 23
G. sunshinyii YC6258 Δ*lcnD*	Δ*lcnD* modules 21 to 22
G. sunshinyii YC6258 Δ*lcnBCDE*	Δ*lcnBC* modules 14 to 15 and 17 to 24
G. sunshinyii YC6258 Δ*lcnBCD*	Δ*lcnBC* modules 14 to 15 and 20 to 23
G. sunshinyii YC6258 Δ*lcnBCD*	Δ*lcnBC* modules 14 to 15 and 21 to 22
B. subtilis DK1042 (*55*)	B. subtilis 3610 $comI^{Q12L}$
B. subtilis DK1042 8PD10 fusion site NAHVILEE	Δ^- *pksM* (DH8)-*ymzB*, Δ^+ *psyD* (dKS9-KS11), *SpcR*, $comI^{Q12L}$
B. subtilis DK1042 8PD10 fusion site LPTYPFx_5W	Δ^- *pksM* (DH8)-*ymzB*, Δ^+ *psyD* (dKS9-KS11), *SpcR*, $comI^{Q12L}$
B. subtilis DK1042 4PD10 fusion site NAHVILEE	Δ^- *pksM* (DH4)-*ymzB*, Δ^+ *psyD* (dKS9-KS11), *SpcR*, $comI^{Q12L}$
B. subtilis DK1042 4PD10 fusion site LPTYPFx_5W	Δ^- *pksM* (DH4)-*ymzB*, Δ^+ *psyD* (dKS9-KS11), *SpcR*, $comI^{Q12L}$
B. subtilis DK1042 4PD11 fusion site LPTYPFx_5W	Δ^- *pksM* (DH4)-*ymzB*, Δ^+ *psyD* (dKS10-KS11), *SpcR*, $comI^{Q12L}$
B. subtilis DK1042 4OnnJ fusion site NAHVILEE	Δ^- *pksM* (DH4)-*ymzB*, Δ^+ *onnJ* (dKS9), *SpcR*, $comI^{Q12L}$

Table S3 Primers used for construction of Golden Gate donor and acceptor plasmids for the *Bacillus* mutants. Lowercase letters indicate overhangs resulting from restriction enzyme digestion.

**Primer name**	**Primer sequence**	**Template**	**Resulting construct**
Gent_*Dra*III Fw	ATCACACCGTGTTAGGTGGCGGTAC	TREX vector pIC20H-RL (*58*)	pFus_A/*Gm*
Gent_*Dra*III Rv	ATCACGTAGTGTAGGGATAACAGGGTAA		
GG_*Spe*^{R}2 Fw	ttACTAGTTAACCATCGTGACGCGGC	pIC333 (*79*)	pGEM_GG_*Spc*^{R}
GG_*Spe*^{R}2 Rv	atCCTGCAGGCTAATTGAGAGAAGTTTC		
Cm^{R}2.2Fus Fw	CTTACATAAGGAGGAACTACTATGGAGAAAAAAATCACTGGA	pACYC184 (New England BioLabs)	pTopo_*Cm*^{R}
GG_*Cm*^{R}2.2 Rv	TGGTCTCATACGTTACGCCCCGCCC		
Phspac Fw	AGTTTAAACTACACAGCCCAGTCCAGACT	pMF37/pDGICZ (*80*)	pTopo_GG_pH*Cm*^{R}
pHspacFus Rv	TCCAGTGATTTTTTTCTCCATAGTAGTTCCTCCTTATGTAAG		
Kan^{R}7 Fw	AGGTCTCACTATACTAGTCTGCGCTAGCATG	pCOLADuet-1 (Novagen)	pGEM_*Kan*^{R}
Kan^{R}7 Rv	AGGTCTCAGAGT TTAGAAAAACTCATCGAGCATCAAATG		
GG_*ymzB* Fw	AGGTCTCACGTATGCACGATCTGTTACGA	*B. subtilis* 3610 gDNA (DSMZ)	pJET_GG_*ymzB*
GG_*ymzB*E Rv	TGGTCTCTCGCCTTAATTAACATCAAAACTGAACC		
PsyD10 (1) Fw	atatatACTAGTGCGCCTCCGGA	pPSCG2 (*80*)	pGEM_*PsyD*10
PsyD10 (2) Rv	tGTTTAAACTAACAACAGATGTTGACGC		
PsyD11 (1) Fw	atatatACTAGTCACCGGTCGAGCA	pPSCG2 (*80*)	pTopo_*PsyD*11
PsyD11 (2) Rv	tGTTTAAACGCTCTATCTTTGGCCA		
pTOPO_*pksL*_KS4_up Fw	AGGTCTCACTATGAAGAAGCATCAGCAG	*B. subtilis* 3610 gDNA (DSMZ)	pTOPO_*pksL*_KS4_up
pTOPO_*pksL*_KS4_up Rv	TGGTCTCTagcTTCCTCAAGGATAATATGTG		
pTOPO_*pksL*_KS5_down Fw	AGGTCTCAAgctTATGCTCCGGAACC	*B. subtilis* 3610 gDNA (DSMZ)	pTOPO_*pksL*_KS5_down
pTOPO_*pksL*_KS5_down Rv	TGGTCTCTGAGTACTCGTGTGCTAAGTA		
pTOPO_*pksM*_up Fw	AGGTCTCACGTAAAGAGAGGAGTGGGA	*B. subtilis* 3610 gDNA (DSMZ)	pTOPO_*pksM*_up
pTOPO_*pksM*_up Rv	TGGTCTCTCGCCTTAATTAACGCAAATACATT		
*pksL*KS5down_d Fw	AGGTCTCACGTATATGCTCCGGAACCTGTGG	*B. subtilis* 3610 gDNA (DSMZ)	pTOPO_*pksL*_KS5_down2
*pksL*KS5down_d Rv	TGGTCTCTCGCCTTAATTAAACTCGTGTGCTAAGTA		
pTOPO_*pksL*_down1 Fw	atatatACTAGTTATGCTCCGGAACCTGTGGA	*B. subtilis* 3610 gDNA (DSMZ)	pTOPO_*pksL*_down1
pTOPO_*pksL*_down1 Rv	tGTTTAAACTGATGCTTGCTTGCACGTCC		
pTOPO_*pksL*_down2 Fw	atatatACTAGTCATGTGTGAGCTCCTTCGG	*B. subtilis* 3610 gDNA (DSMZ)	pTOPO_*pksL*_down2

Table S3 continued.

pTOPO_*pksL*_down2 Rv	tGTTTAAACCCCACTCCTCTCTTATTTGAAAGT		
pksL Fw	AGGTCTCACTATATATAGGAGGCCG	*B. subtilis* 3610 gDNA (DSMZ)	pTOPO_GG_*pksL*_hom
pksL Rv	TGGTCTCTGAGTTTATTTGAAAGTTTTCC		
pksM Fw	AGGTCTCACGTAAGAGAGGAGTGG	*B. subtilis* 3610 gDNA (DSMZ)	pTOPO_GG_*pksM*_hom
pksM Rv	TGGTCTCTCGCCCGCAAATACATT		

Table S4 Golden Gate assemblies in *Bacillus* hybrids.

**Construct**	**Part**	**Template plasmid**
PRB8OJ11	backbone	pFus_A/Spc
	upstream genome homology	pGEM_GG_pksMKS8
	first gene fragment	pGEM_GG_onnJKS11
	resistance cassette	pTopo_GG_pHCm^R
	downstream genome homology	pJET_GG_*ymzB*

**Construct**	**Part**	**Template plasmid**
pFusA/S/C_Kan^R	backbone	pFus_A/Spc
	spacer	pGEM_GG_Kan^R
	resistance cassette	pTopo_GG_pHCm^R
	downstream genome homology	pJET_GG_*ymzB*

**Construct**	**Part**	**Template plasmid**
pFusA/G/S_Kan^R	backbone	pFus_A/Gm
	spacer	pGEM_GG_Kan^R
	resistance cassette	pGEM_GG_Spc^R
	downstream genome homology	pJET_GG_*ymzB*

Table S5 Overview of restriction digests performed to obtain *pks-psy* and *pks-onn* chimeric constructs from subcloned plasmids**.**

**Construct**	**Acceptor plasmid**	**Donor plasmid**
pFusA_PD9(1)	pFusA/G/S_Kan^R	pGEM_*psyD*9(1)
pFusA_PD9(2)	pFusA/S/C_Kan^R	pGEM_*psyD*9(1)
pFusA_PD10	pFusA/G/S_Kan^R	pGEM_*psyD*10
pFusA_PD11	pFusA/S/C_Kan^R	*pGEM_psyD11*
Fus_A/G/C_psyD11	pFus_A/S/C_Kan^R	pTopo_psyD11

pFus_A/G/S_onnJI	pFusA/G/S_Kan^R	pTopo_onnJI
pFus_A/S/C_onnJII	pFus_A/S/C_Kan^R	pTopo_onnJII
pFus_A/G/S_onnJIII	pFusA/G/S_Kan^R	pTopo_onnJIII
pFus_A/S/C_onnJIV	pFus_A/S/C_Kan^R	pTopo_onnJIV

Table S6 Overview of primers used in Gibson assemblies of *pks-psy* chimeric PKSs.

Construct	Part	Template	Primer sequence
pBAD-4PD11 Gibson cloning	pBAD	pBAD/Myc-His	GTAAATGCTTGATGAACAAGTGATCTCAATAGCGCCGTCGACCATCATC
			CTTTGGCGTATAGCCCGCATCCATGGTTAATTCCTCCTGTTAGCC
	upstream genome homology	*B. subtilis* DK1042	GGCTAACAGGAGGAATTAACCATGGATGCGGGCTATACGCCAAAG
			TTCCAAAACGGGAATCCAATAACGCACCCTCTCAAATGGG
	psyD$_{DH-ACP-TE}$	pPSCG2 (*21*)	AGGGTGCGTTATTGGATTCCCGTTTTGGAAGAGAAGGCGG
			GCGTCACGATGGTTAGCTCTATCTTTGGCCAGGCCATAAC
	spcR resistance cassette	pIC333	GGCCAAAGATAGAGCTAACCATCGTGACGCGGCATTCTAG
			ATCCAGCACAGCTGACTAATTGAGAGAAGTTTCTATAGAATTTTTCATATACTTAACGAG
	downstream genome homology	*B. subtilis* DK1042	ACTTCTCTCAATTAGTCAGCTGTGCTGGATATCAATTGTATATAC
			GATGATGGTCGACGGCGCTATTGAGATCACTTGTTCATCAAGCATTTAC

Construct	Part	Template	Primer sequence
pBAD-4PD10-NAHVILEE Gibson cloning	backbone	pBAD-4PD11	AGCACCGGTCGAGCAATTCCCGTTTTGGAAGAGAAGGCGG
			GCCTTCCGGAGGCGCTTCCTCAAGGATAATATGTGCGTTTGAAC
	psyD$_{ACP-KS11}$	pPSCG2 (*21*)	ATTATCCTTGAGGAAGCGCCTCCGGAAGGCGTCAAGGCAC
			TTCCAAAACGGGAATTGCTCGACCGGTGCTTCCCCCAGAATG

Construct	Part	Template	Primer sequence
pBAD-4PD10-LPTYPFx_5W Gibson cloning	backbone	pBAD-4PD11	AGCACCGGTCGAGCAATTCCCGTTTTGGAAGAGAAGGCGG
			AATGAGCTCGGTAATCCAATAACGCACCCTCTCAAATGGG
	psyD$_{ACP-KS11}$	pPSCG2 (*21*)	AGGGTGCGTTATTGGATTACCGAGCTCATTCCCTCGGGCATG
			TTCCAAAACGGGAATTGCTCGACCGGTGCTTCCCCCAGAA

Construct	Part	Template	Primer sequence
pBAD-8PD10-NAHVILEE Gibson cloning	backbone	pBAD-4PD11	CTTATTTTGGAAGAAGCGCCTCCGGAAGGCGTCAAGGCAC
			AGCAATTGTCTCTGGCATGGTTAATTCCTCCTGTTAGCCCAAAAAACGGG
	psyD$_{ACP-KS11}$	pPSCG2 (*21*)	GAGGAATTAACCATGCCAGAGACAATTGCTTCACCGCAGG
			GCCTTCCGGAGGCGCTTCTTCCAAAATAAGATGCGCATTTGATC

Construct	Part	Template	Primer sequence
pBAD-8PD10-LPTYPFx_5W Gibson cloning	backbone	pBAD-4PD11	AGGGAGCGCTACTGGATTACCGAGCTCATTCCCTCGGGCATG
			AGCAATTGTCTCTGGCATGGTTAATTCCTCCTGTTAGCCCAAAAAAC
	psyD$_{ACP-KS11}$	pPSCG2 (*21*)	GAGGAATTAACCATGCCAGAGACAATTGCTTCACCGCAGG
			AATGAGCTCGGTAATCCAGTAGCGCTCCCTTGCAAACGGATAG

Table S7 Screening primers for verification of genomic integration in *Bacillus subtilis* mutants encoding chimeric *pks* PKSs.

**Screening primer name**	**Primer sequence**	**Primer binding**
CP *Cat*2.2 Fw	CGTGGCCAATATGGACAACTTC	Inside Cm^R marker
CP2_*Spc* Fw	AAGTGGGAAGGACTATATTCAAAGG	Inside SpcR marker
CP all Bs Rv	CATCCCGATGGACAAACTTGG	Downstream of „*ymzB*"-DGH
PKS_L/M Check Rv	AACAGCAGTGTCGGAGCAAG	Downstream of „*pksM*"-DGH

Table S8 Primers used for the construction of the S. plymuthica 4Rx13 deletion strains.

**Name**	**Sequence**
KO_SOD_c22970_rev	AACTCGTAAGCTCAGCCTTGGCGAGAAACGGCGTATAAGCGGCGCCTTCCTAATACGACTCACTATAGGGCTC
KO_SOD_c22970_fw	GAGGACGCCTGTTATTTGCCACAACAGCTAAGCGAAGCCAGCGTCGATGTAATTAACCCTCACTAAAGGGCGG
KO_SOD_b01030_fwd	CCGGCAGACAAAGCCTATTACATCCTTAATGATGATCGGGCTTCGACGGAAATTAACCCTCACTAAAGGGCGG

Table S9 Overview of primers used to construct *S. plymuthica* 4Rx13 mutants

**Construct**	**Part**	**Template**	**Primer sequence**
pBAD-oocQRC	pBAD	pBAD	TAGCGGCTTTGGATGTTTAGAATAGCGCCGTCGACCATCATCATCATCATC
			TTTATCAGGTACTCGCTCATCATGGTTAATTCCTCCTGTTAGCCCAAAAAAC
	oocQ and *oocR*$_{KS011\text{-}DH\text{-}ACP\text{-}KS12}$	*S. plymuthica* 4Rx13	AACAGGAGGAATTAACCATGATGAGCGAGTACCTGATAAATTCCGGCGAG
			GCAGGTTCGGCGTTGCCGGCGATCCAGTATTGATCGGTCGCGAACGGATAAG
	oocS$_{ACP\text{-}C}$	*S. plymuthica* 4Rx13	CGACCGATCAATACTGGATCGCCGGCAACGCCGAACCTGCGCCTGTCGGC
			TGATGGTCGACGGCGCTATTCTAAACATCCAAAGCCGCTACCTCCTCCGGCGATAAACGATCGATC

**Construct**	**Part**	**Template**	**Primer sequence**
pBAD-oocQR	pBAD-*oocQR*$_{to\text{-}KS12}$	pBAD-oocQRC	CGATCAATACTGGATCAATAGCGCCGTCGACCATCATCATCATCATC
			GACGGCGCTATTGATCCAGTATTGATCGGTCGCGAACGGATAAG

**Construct**	**Part**	**Template**	**Primer sequence**
pBAD-oocQR^{LPTYPFx5W}-PsyDend	pBAD-*oocQR*$_{to\text{-}KS12}$	pBAD-oocQRC	TATGGCCTGGCCAAAGATAGAATAGCGCCGTCGACCATCATCATCATCATC
			GAGGGAATGAGCTCGGTAATGATCCAGTATTGATCGGTCGCGAACGGATAAG
	psyD$_{end}$ part 1	pPSCG2 (*21*)	CGACCGATCAATACTGGATCATTACCGAGCTCATTCCCTCGGGCATGCGC
			TCTCGTAAATCGGGGCGGACCAGCGGCTTCGCACGACCAGGCCACAGGAC
	psyD$_{end}$ part 2	pPSCG2 (*21*)	CTGGTCGTGCGAAGCCGCTGGTCCGCCCCGATTTACGAGAGCCGCGTTTG
			TGATGGTCGACGGCGCTATTCTATCTTTGGCCAGGCCATAACAAACGAATGATCTCATG

Table S9 continued.

Construct	Part	Template	Primer sequence
pBAD-oocQR^{NAHVILEE}-PsyDend	*psyD*-pBAD-*oocQR*$_{to\text{-}KS12}$	pBAD-oocQR^{LPTYPFx5W}-PsyDend	TTTTGAAGCAACGGTTTTGGATTACCGAGCTCATTCCCTCGGGCATGCGCGATGAGCCCG
			TTGACGCCTTCCGGAGGCGCTTCCTCCAGCACGATATGTGCATTGGCGCCGCCAAAGCCG
	psyD$_{KS10}$ FSD	pPSCG2 (*21*)	CACATATCGTGCTGGAGGAAGCGCCTCCGGAAGGCGTCAAGGCACCCGGGGATGAGATGG
			GAGGGAATGAGCTCGGTAATCCAAAACCGTTGCTTCAAAAATGGGTAGGTAGGAAGTGTCAACGTTCGCCTGACTTG

Construct	Part	Template	Primer sequence
pBAD-oocQR-Lbm12-oocS$_{ACP\text{-}C}$	*oocS*$_{ACP\text{-}C}$-pBAD-*oocQR*$_{to\text{-}KS12}$	pBAD-oocQRC	TTGCCAAAAAACGTTGCTGGGCCGGCAACGCCGAACCTGCGCCTGTCGGCAAATCATCGG
			GTATGGACCGCATCATTGAGGATCCAGTATTGATCGGTCGCGAACGGATAAGTCGGCAGGCTGATGC
	lbmD$_{ACP\text{-}KS12}$	*G. sunshinyii* YC6258	CGACCGATCAATACTGGATCCTCAATGATGCGGTCCATACCGATGCGTCTGTGGTGCCGG
			GCAGGTTCGGCGTTGCCGGCCCAGCAACGTTTTTTGGCAAACGCATAGGTCGGCAGTGGC

Construct	Part	Template	Primer sequence
pBAD-oocQR^{NAHVILEE}-Lbm12-oocS$_{ACP\text{-}C}$	*lbmD*$_{ACP\text{-}KS12}$-*oocS*$_{ACP\text{-}C}$-pBAD-*oocQR*$_{to\text{-}KS12}$	pBAD-oocQR-Lbm12-C	TCCGTTTGCACCAGAGCGGCATTGGCTCAATGATGCGGTCCATACCGATG
			GTCCGGCCCGAACGCATCTTCAACATACTCCAGCACGATATGTGCATTGGCG
	lbmD$_{KS11}$ FSD	*G. sunshinyii* YC6258	CGCCAATGCACATATCGTGCTGGAGTATGTTGAAGATGCGTTCGGGCCGGAC
			CATCGGTATGGACCGCATCATTGAGCCAATGCCGCTCTGGTGCAAACGGA

Construct	Part	Template	Primer sequence
pBAD-oocQR-Lbm12^{NAHVILEE}--oocS$_{ACP\text{-}C}$	*lbmD*$_{ACP\text{-}KS12}$-*oocS*$_{ACP\text{-}C}$-pBAD-*oocQR*$_{to\text{-}KS12}$	pBAD-oocQR-Lbm12-C	TCCGTTTGACGCGCGTTTATGCTGGGCCGGCAACGCCGAACCTGCG
			CCCCGTTTGAATCAGGCTCGGCGACCTCGGCCACGATCACATGTACATTC
	lbmD$_{KS12}$ FSD	*G. sunshinyii* YC6258	GAATGTACATGTGATCGTGGCCGAGGTCGCCGAGCCTGATTCAAACGGGG
			CGCAGGTTCGGCGTTGCCGGCCCAGCATAAACGCGCGTCAAACGGA

Construct	Part	Template	Primer sequence
pBAD-oocQR^{NAHVILEE}-Lbm12^{NAHVILEE}-oocS$_{ACP\text{-}C}$	*lbmD*$_{ACP\text{-}KS12}$-*oocS*$_{ACP\text{-}C}$-pBAD-*oocQR*$_{to\text{-}KS12}$	pBAD-oocQR^{NAHVILEE}-Lbm12-C	TCCGTTTGACGCGCGTTTATGCTGGGCCGGCAACGCCGAACCTGCG
			CCCCGTTTGAATCAGGCTCGGCGACCTCGGCCACGATCACATGTACATTC
	lbmD$_{KS12}$ FSD	*G. sunshinyii* YC6258	GAATGTACATGTGATCGTGGCCGAGGTCGCCGAGCCTGATTCAAACGGGG
			CGCAGGTTCGGCGTTGCCGGCCCAGCATAAACGCGCGTCAAACGGA

Table S9 continued.

Construct	Part	Template	Primer sequence
pBAD-oocQR-Lbm11-oocS$_{ACP-C}$	*oocS*$_{ACP-C}$-pBAD-*oocQR*$_{to-KS12}$	pBAD-oocQRC	TTGCACCAGAGCGGCATTGGGCCGGCAACGCCGAACCTGCGCCTGTCGGCAAATC
			TCCAGCGTCATCCCGCCCTGGATCCAGTATTGATCGGTCGCGAACGGATAAGTCGGCAGGC
	lbmD$_{ACP-KS11}$	*G. sunshinyii* YC6258	CGACCGATCAATACTGGATCCAGGGCGGGATGACGCTGGATGATACCACCAGGAC
			GCAGGTTCGGCGTTGCCGGCCCAATGCCGCTCTGGTGCAAACGGATACGTGGGTAAAC

Construct	Part	Template	Primer sequence
pBAD-oocQR-Pks5-oocS$_{ACP-C}$	*oocS*$_{ACP-C}$-pBAD-*oocQR*$_{to-KS12}$	pBAD-oocQRC	TCGCAAGAGATCGCTATTGGGCCGGCAACGCCGAACCTGCGCCTGTCGGCAAATC
			GCATCAATTTGCATGCCCCCGATCCAGTATTGATCGGTCGCGAACGGATAAGTCGGCAGGC
	pksL$_{ACP-KS5}$	*B. subtilis* DK1042	CGACCGATCAATACTGGATCGGGGGCATGCAAATTGATGCGGAAACTGCAAGGAT
			GCAGGTTCGGCGTTGCCGGCCCAATAGCGATCTCTTGCGAAAGGATAGGCAGGTAAAC

Construct	Part	Template	Primer sequence
pBAD-oocQR-Tar10-oocS$_{ACP-C}$	*oocS*$_{ACP-C}$-pBAD-*oocQR*$_{to-KS12}$	pBAD-oocQRC	GCCGGCAACGCCGAACCT
			GATCCAGTATTGATCGGTCGCGAACG
	tarE$_{ACP-KS8}$	*G. sunshinyii* YC6258	TATCCGTTCGCGACCGATCAATACTGGATCCCCGATGACGGTGCGGCG
			GCCGACAGGCGCAGGTTCGGCGTTGCCGGCCCAGTAACGTTCACCACCAAACGGC

Construct	Part	Template	Primer sequence
pBAD-oocQR-Tar13-oocS$_{ACP-C}$	*oocS*$_{ACP-C}$-pBAD-*oocQR*$_{to-KS12}$	pBAD-oocQRC	GCCGGCAACGCCGAACCT
			GATCCAGTATTGATCGGTCGCGAACG
	tarF$_{ACP-KS13}$	*G. sunshinyii* YC6258	TATCCGTTCGCGACCGATCAATACTGGATCTTAACGCCGGAAACGTCTG
			GCCGACAGGCGCAGGTTCGGCGTTGCCGGCCCAATAGGATTTCTCCTCAAACG

Construct	Part	Template	Primer sequence
pBAD-oocQR-Gyn13-oocS$_{ACP-C}$	*oocS*$_{ACP-C}$-pBAD-*oocQR*$_{to-KS12}$	pBAD-oocQRC	CCCATGAACGTTACTGGATCGCCGGCAACGCCGAACCTGCGCCTGTCGGC
			GCCGGCGACGACTGCATGCCGATCCAGTATTGATCGGTCGCGAACGGATAAG
	lcnB$_{ACP-KS13}$	*G. sunshinyii* YC6258	CGACCGATCAATACTGGATCGGCATGCAGTCGTCGCCGGCAGCTACCCGG
			GCAGGTTCGGCGTTGCCGGCGATCCAGTAACGTTCATGGGAAAACGGATATACCGGCAGTTCCAG

Table S9 continued.

Construct	Part	Template	Primer sequence
pBAD-oocQR-Lcn24-oocS$_{ACP-C}$	*oocS*$_{ACP-C}$-pBAD-*oocQR*$_{to-KS12}$	pBAD-oocQRC	GCCGGCAACGCCGAACCT
			GATCCAGTATTGATCGGTCGCGAACG
	lcnE$_{ACP-ACP-KS24}$	*G. sunshinyii* YC6258	TATCCGTTCGCGACCGATCAATACTGGATCATGACTCACAACACTATGTTATTC
			GCCGACAGGCGCAGGTTCGGCGTTGCCGGCCCAGTAACGCTCATGAGC

Construct	Part	Template	Primer sequence
pBAD-oocQR-Lcn1-oocS$_{ACP-C}$	*oocS*$_{ACP-C}$-pBAD-*oocQR*$_{to-KS12}$	pBAD-oocQRC	GCCGGCAACGCCGAACCT
			GATCCAGTATTGATCGGTCGCGAACG
	lcnA$_{KS1}$	*G. sunshinyii* YC6258	TATCCGTTCGCGACCGATCAATACTGGATCATGACTCACAACACTATGTTATTC
			GCCGACAGGCGCAGGTTCGGCGTTGCCGGCCCAGTAACGCTCATGAGC

Construct	Part	Template	Primer sequence
pBAD-oocQR-Tar11-oocS$_{ACP-C}$	*oocS*$_{ACP-C}$-pBAD-*oocQR*$_{to-KS12}$	pBAD-oocQRC	GCCGGCAACGCCGAACCT
			GATCCAGTATTGATCGGTCGCGAACG
	tarF$_{ACP-KS11}$	*G. sunshinyii* YC6258	TATCCGTTCGCGACCGATCAATACTGGATCATGAACCGCTCAGAACAC
			GCCGACAGGCGCAGGTTCGGCGTTGCCGGCCCACAGTTTAAGCCGGGC

Construct	Part	Template	Primer sequence
pBAD-oocQR-Gyn3-oocS$_{ACP-C}$	*oocS*$_{ACP-C}$-pBAD-*oocQR*$_{to-KS12}$	pBAD-oocQRC	GCCGGCAACGCCGAACCT
			GATCCAGTATTGATCGGTCGCGAACG
	gynD$_{KR-ACP-KS3}$	*G. sunshinyii* YC6258	TATCCGTTCGCGACCGATCAATACTGGATCCTGGATGGGAAACTGGCGTC
			GCCGACAGGCGCAGGTTCGGCGTTGCCGGCCCAGAAACGCTGCCGGGC

Construct	Part	Template	Primer sequence
pBAD-oocQR-Lcn6-oocS$_{ACP-C}$	*oocS*$_{ACP-C}$-pBAD	pBAD-oocQRC	CGTGATCCGTACTGGGCCGGCAACGCCGAACCTGCGCCTG
			CAGGTACTCGCTCATCATGGTTAATTCCTCCTGTTAGCCCAAAAAACGGGTATGGAGAAAC
	oocQ and *oocR*$_{KS011-DH-ACP-KS12}$	*S. plymuthica* 4Rx13	GAGGAATTAACCATGATGAGCGAGTACCTGATAAATTCCG
			GGTGGTATCCGGGGCGATCCAGTATTGATCGGTCGCGAAC

Construct	Part	Template	Primer sequence
pBAD-oocQR-Lbm11$^{DH-KR-ACP-KS}$-oocS$_{ACP-C}$	*oocS*$_{ACP-C}$-pBAD-*oocQR*$_{to-KS12}$	pBAD-oocQRC	TTGCACCAGAGCGGCATTGGGCCGGCAACGCCGAACCTGCGCCTGTCGGCAAATC
			TCAACACTGACGGCATCGCGGATCCAGTATTGATCGGTCGCGAACGGATAAGTCGGCAGGC
	lbmD$_{DH-KR-ACP-KS11}$	*G. sunshinyii* YC6258	CGACCGATCAATACTGGATCCGCGATGCCGTCAGTGTTGATCAGACATCTTCTCC
			GCAGGTTCGGCGTTGCCGGCCCAATGCCGCTCTGGTGCAAACGGATACGTGGGTAAAC

Table S9 continued.

Construct	Part	Template	Primer sequence
pBAD-oocQR-Pks5$^{DH-KR-ACP-KS}$-oocS$_{ACP-C}$	*oocS*$_{ACP-C}$-pBAD-*oocQR*$_{to-KS12}$	pBAD-oocQRC	TCGCAAGAGATCGCTATTGGGCCGGCAACGCCGAACCTGCGCCTGTCGGCAAATC
			TTTTTCTCCGCTTTCGGCACGATCCAGTATTGATCGGTCGCGAACGGATAAGTCGGCAGGC
	pksL$_{DH-KR-ACP-KS5}$	*B. subtilis* DK1042	CGACCGATCAATACTGGATCGTGCCGAAAGCGGAGAAAAAGACTGATCGTTCAAA
			GCAGGTTCGGCGTTGCCGGCCCAATAGCGATCTCTTGCGAAAGGATAGGCAGGTAAAC

Construct	Part	Template	Primer sequence
pBAD-oocQR-Lbm9-oocS$_{ACP-C}$	*oocS*$_{ACP-C}$-pBAD-*oocQR*$_{to-KS12}$	pBAD-oocQRC	CGTGATCCGTACTGGGCCGGCAACGCCGAACCTGCGCCTG
			CAGGTACTCGCTCATCATGGTTAATTCCTCCTGTTAGCCCAAAAAACGGGTATGGAGAAAC
	lbmD$_{ACP-KS10}$	*G. sunshinyii* YC6258	TATCCGTTCGCGACCGATCAATACTGGATCATGAAAAAAAACAATACCAGCCTTC
			GCCGACAGGCGCAGGTTCGGCGTTGCCGGCCCAATAATGCTCCCGGGC

Construct	Part	Template	Primer sequence
pBAD-oocQ-oocR$_{KS0-DH-ACP}$-Lbm$_{KS12-FSD}$-oocS$_{ACP-C}$	Lbm$_{KS12FSD}$-oocS$_{ACP-C}$-pBAD$_{ori}$	pBAD-oocQR-Lbm12-C	TGACGAGACAAAACCCGACATCCCGCGTTC
			ACAGGATTAGCAGAGCGAGGTATGTAGGCG
	PBAD$_{to\ ori}$ oocQ-oocR$_{KS0-DH-ACP}$	pBAD-oocQR-Lbm12-C	CCTCGCTCTGCTAATCCTGTTACCAGTGGCTGC
			TGTCGGGTTTTGTCTCGTCATGGCGCCTC

Construct	Part	Template	Primer sequence
pBAD-oocQ-oocR$_{KS0-DH-ACP}$-Lbm$_{KS11-FSD-ACP-KS12-FSD}$-oocS$_{ACP-C}$	Lbm$_{KS12FSD}$-oocS$_{ACP-C}$-pBAD$_{ori}$	pBAD-oocQR-Lbm12-oocS$_{ACP-C}$	TGATCGTGGCCGAGGCACCTGAACGTGTCGGTTCGAATCA
			TATCCGGTAACTATCGTCTTGAGTCCAACCCGGTAAGACA
	PBAD$_{to\ ori}$ oocQ-oocR$_{KS0-DH-ACP}$	pBAD-oocQR-Lbm12-oocS$_{ACP-C}$	GGTTGGACTCAAGACGATAGTTACCGGATAAGG
			AGACTGGCACTATCCGCTGTCTCGTCATGG
	lbmD$_{KS11-ACP-KS12}$	G. sunshinyii YC6258	ACAGCGGATAGTGCCAGTCTGGTGGATAC
			TTCAGGTGCCTCGGCCACGATCA

Table S10 Primers used in to create *G. sunshinyii* mutants producing truncated lacunalides.

Construct	Template	Part	ID	Sequence
pEB17_ Δ*lcn*14-15	pEB17	Δ*lcn*14-15 backbone forward	GS01	AAGGTGAACTGAATTCCCATGTCAGCCG
		Δ*lcn*14-15 backbone reverse	GS02	TCGGGTGCCTAGGTCGACTCTAGAGGATC
	G. sunshinyii	Δ*lcn*14-15 homology arm 1 forward	GS03	GAGTCGACCTAGGCACCCGAAACCCATAC
		Δ*lcn*14-15 homology arm 1 reverse	GS04	GATGATCGGTGATCCAGTAACGTTCATGGGAAAAC
	G. sunshinyii	Δ*lcn*14-15 homology arm 2 forward	GS05	TTACTGGATCACCGATCATCTGCCGACG
		Δ*lcn*14-15 homology arm 2 reverse	GS06	ATGGGAATTCAGTTCACCTTCACCGTGAG

Table S11 continued.

Construct	Template	Part	ID	Sequence
pEB17_ Δ*lcn*17-24	pEB17	Δ*lcn*17-24 backbone forward	GS07	TTTATCAGGCGAATTCCCATGTCAGCCG
		Δ*lcn*17-24 backbone reverse	GS08	TGCGGACGATAGGTCGACTCTAGAGGATC
	G. sunshinyii	Δ*lcn*17-24 homology arm 1 forward	GS09	GAGTCGACCTATCGTCCGCAGGCACTATTC
		Δ*lcn*17-24 homology arm 1 reverse	GS10	AATGATCCGGCCAGAAGCTGTCACGGGC
	G. sunshinyii	Δ*lcn*17-24 homology arm 2 forward	GS11	CAGCTTCTGGCCGGATCATTCAGAGACG
		Δ*lcn*17-24 homology arm 2 reverse	GS12	ATGGGAATTCGCCTGATAAACGTCATAAC

Table S10 continued.

Construct	Template	Part	ID	Sequence
pEB17_ Δ*lcn*20-23	pEB17	Δ*lcn*20-23 backbone forward	GS13	CCGATCTGTAGAATTCCCATGTCAGCCG
		Δ*lcn*20-23 backbone reverse	GS14	TGCAACACGAAGGTCGACTCTAGAGGATC
	G. sunshinyii	Δ*lcn*20-23 homology arm 1 forward	GS15	GAGTCGACCTTCGTGTTGCAGGAATACC
		Δ*lcn*20-23 homology arm 1 reverse	GS16	TGAGGGGCAACCAGTAAGTTTCCCGCGC
	G. sunshinyii	Δ*lcn*20-23 homology arm 2 forward	GS17	AACTTACTGGTTGCCCCTCAAGGCATCG
		Δ*lcn*20-23 homology arm 2 reverse	GS18	ATGGGAATTCTACAGATCGGTACTGCTGACC

Construct	Template	Part	ID	Sequence
pEB17_ Δ*lcn*21-22	pEB17	Δ*lcn*21-22 backbone forward	GS19	GCACGCTGCAGAATTCCCATGTCAGCCG
		Δ*lcn*20-23 backbone reverse	GS20	CTTGCCGCCGAGGTCGACTCTAGAGGATC
	G. sunshinyii	Δ*lcn*21-22 homology arm 1 forward	GS21	GAGTCGACCTCGGCGGCAAGGCCGGTAT
		Δ*lcn*21-22 homology arm 1 reverse	GS22	TGCCGGCGCTCCAGTATTTTTCCCGCGCAAA CGG
	G. sunshinyii	Δ*lcn*21-22 homology arm 2 forward	GS23	AAAATACTGGAGCGCCGGCAATATCGGC
		Δ*lcn*21-22 homology arm 2 reverse	GS24	ATGGGAATTCTGCAGCGTGCGGCCGTTG

Table S10 continued.

Template	Plasmid verification	ID	Sequence
pEB17 plasmids	Upstream	GS25	CTAAATAATAGTGAACGGCAGGTATATG
	Downstream	GS26	AGGGATGTAACGCACTGAGAAGC
G. sunshinyii mutants (Δ*lcn*14-15, Δlcn14-15_17-24, Δlcn14-15_Δlcn20-23, Δlcn14-15_Δlcn21-22)	Δ*lcn*14-15 genomic upstream	GS27	GCGGCAACTGACTGCATGGC
G. sunshinyii mutants (Δ*lcn*14-15, Δlcn14-15_17-24, Δlcn14-15_Δlcn20-23, Δlcn14-15_Δlcn21-22)	Δ*lcn*14-15 genomic downstream	GS28	GATTCCGGCAACTGTAGTCGTG
G. sunshinyii mutants (Δlcn17-24, Δlcn14-15_Δlcn17-24)	Δ*lcn*17-24 genomic upstream	GS29	ATCAATGGCGTCATGCAGGAACG
G. sunshinyii mutants (Δlcn17-24, Δlcn14-15_Δlcn17-24)	Δ*lcn*17-24 genomic downstream	GS30	CTGCCGGCAACGTCAGTTTCG
G. sunshinyii mutants (Δlcn20-23, Δlcn14-15_Δlcn20-23)	Δ*lcn*20-23 genomic upstream	GS31	GCGACCACAGCTGTCGCAGG
G. sunshinyii mutants (Δlcn20-23, Δlcn14-15_Δlcn20-23)	Δ*lcn*20-23 genomic downstream	GS32	CAATCGCAAGGCGGCAACCG
G. sunshinyii mutants (Δlcn21-22, Δlcn14-15_Δlcn21-22)	Δ*lcn*21-22 genomic upstream	GS33	CAGCAGCGGCTCGGAGCC
G. sunshinyii mutants (Δlcn21-22, Δlcn14-15_Δlcn21-22)	Δ*lcn*21-22 genomic downstream	GS34	GACACCTTCATCCTGCCATTGC

Table S11 *G. sunshinyii* mutants generated in this study. A new mutant was generated by conjugative transfer of a suicide plasmid from the donor strain to the respective acceptor strain.

***G. sunshinyii* mutant construct**	**donor *E. coli* ST18 carrying plasmid**	**acceptor strain (*G. sunshinyii*)**
Δ*lcn*14-15	pEB17_Δ*lcn*14-15	wild type
Δ*lcn*17-24	pEB17_Δ*lcn*17-24	wild type
Δ*lcn*20-23	pEB17_Δ*lcn*20-23	wild type
Δ*lcn*21-22	pEB17_Δ*lcn*21-22	wild type
Δ*lcn*14-15_17-24	pEB17_Δ*lcn*17-24	Δ*lcn*14-15
Δ*lcn*14-15_20-23	pEB17_Δ*lcn*20-23	Δ*lcn*14-15
Δ*lcn*14-15_21-22	pEB17_Δ*lcn*21-22	Δ*lcn*14-15

Table S12 Overview of consensus retention times of the detected engineered and natural oocydins, as extracted from the combined UHPLC-MS traces; n.d. indicates not determined. The oocydin PKS produces various product congeners due to various degrees of hydrolysis and reduction of the alkoxy moiety installed in the initial phases of biosynthesis (*13*, *17*), leading to the production of various products with R groups **A**-**D**. The putative structure of scaffold **I** is based on the biosynthetic logic of oocydin biosynthesis (*13*, *17*) and the putative structure of scaffolds **IV** and **V** are based on the NMR-confirmed structure of **2** (**II**-**D**).

(Putative) compound scaffold		R group			
		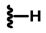 **A**	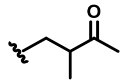 **B**	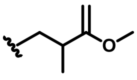 **C**	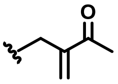 **D**
Putative 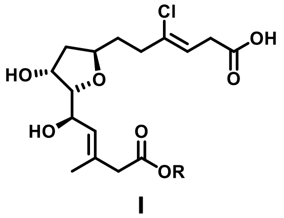 **I**	Parent *m/z*	362.1126	470.1701	458.1701	444.1545
	**M**+H$^+$	363.1205	471.1780	459.1780	445.1624
	**M**+NH_4^+	380.1470	488.2045	476.2045	462.1889
	Retention time (min)	10.28	n.d.	12.02	12.02
	Compound number				**1**
Confirmed by NMR 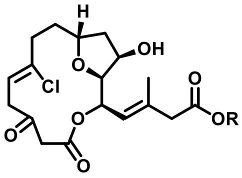 **II**	Parent *m/z*	386.1126	494.1701	482.1701	468.1545
	**M**+H$^+$	387.1205	495.1780	483.1780	469.1624
	**M**+NH_4^+	404.1470	512.2045	500.2045	486.1889
	Retention time (min)	12.55	n.d.	16.72	14.82
	Compound number				**2**
Confirmed by NMR 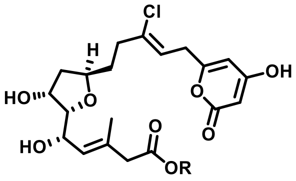 **III**	Parent *m/z*	428.1232	536.1807	524.1807	510.1651
	**M**+H$^+$	429.1311	537.1886	525.1886	511.1730
	**M**+NH_4^+	446.1576	554.2151	542.2151	528.1995
	Retention time (min)	12.81/9.97	n.d.	11.77/14.89	11.88
	Compound number				**3**

Table S12 continued.

Putative 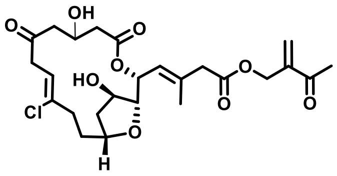	Parent *m/z*	430.1388	538.1963	526.1963	512.1807
	**M**+H$^+$	431.1467	539.2042	527.2042	513.1886
	**M**+NH$_4^+$	448.1732	556.2308	544.2308	530.2151
	Retention time (min)	10.76	n.d.	n.d.	12.8
**IV**	Compound number				**4**
Putative 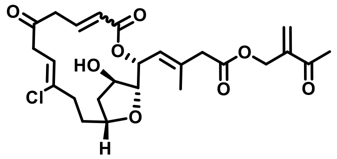	Parent *m/z*	412.1553	520.1858	508.1858	494.1701
	**M**+H$^+$	413.1362	521.1937	509.1937	495.1780
	**M**+NH$_4^+$	430.1627	538.2202	526.2202	512.2046
	Retention time (min)	12.55	n.d.	14.59	14.59
**V**	Compound number				**5**
Confirmed by NMR (*17*) 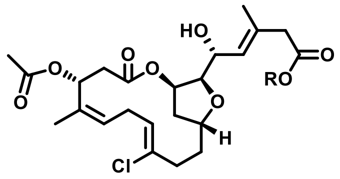	Parent *m/z*	470.1707	554.2283	566.2283	552.2126
	**M**+H$^+$	471.1780	555.2355	567.2355	553.2199
	**M**+NH$_4^+$	488.2045	572.2621	584.2621	570.2464
	Retention time (min)	14.1	17.25	16.01	16.01
Oocydin	Compound name	Oocydin A	Oocydin B	Oocydin C	Haterumalide B

Design and analysis of chimeric bacillaene *trans*-AT PKSs in *B. subtilis*

In our initial attempts to engineer *trans*-AT PKSs, we focused on engineering the well-studied bacillaene *pks* biosynthetic pathway in *B. subtilis* (Fig. SXA). Manual alignment of KS sequences showed the presence of the conserved NAHVILEE motif at the C-terminus of the KS domains (Fig. S1B). We selected the terminal domain series of the *psy* pathway to be introduced into the *pks* assembly line. As fusion sites, we selected either the C-terminus of the 4th or 8th KS domain of the *pks* pathway. This design resulted in a first set of chimeric PKSs, i. e., PksL4$_{NAHVILEE}$-PsyD10 and PksM8$_{NAHVILEE}$-PsyD10, which both contain the C-terminus of PsyD from the 10th KS onward fused directly downstream of either the PksL KS4 or the PksM KS8 KS domain. In a second design, PksL4$_{NAHVILEE}$-PsyD11, the terminal domains downstream of the *psy* KS10 pathway are fused to PksL KS4 at the NAHVILEE site. For primers used to construct these mutants, see Tables S3-6.

HPLC-MS analysis showed only the presence of stalled intermediates up to the fusion points in culture extracts of these mutants, e.g., the intermediate from ACP 11 for PksM8$_{NAHVILEE}$-PsyD10 (Figs. S1 and S3). To compare levels of bacillaene derivatives and intermediates, bacillibactin was used as an internal standard as described previously (*48*, *81*). After elucidation of the LPTYPFx$_5$W motif as useful site for *trans*-AT PKS engineering, we additionally constructed PksL4$_{LPTYPFX5W}$-PsyD10 and PksM8$_{LPTYPFX5W}$-PsyD10. These chimeric assembly lines have similar architectures as the chimeras fused at the NAHVILEE site, but are fused directly downstream of the Pks KS LPTYPFx$_5$W site. Although we were not able to detect ions at *m/z* values corresponding to anticipated products originating from these chimeric PKSs, we note that the intensities of the intermediates released from the ACP upstream of the engineering site is decreased in the PksM8 $_{LPTYPFX5W}$-PsyD10 mutant. This decrease in intensity suggests that this assembly line might be processive and converts these intermediates into downstream products. However, as for wild-type bacillaene, the large number of conjugated double bonds, together with potential cyclization of the product, is likely to induce rearrangements and/or other decomposition processes that preclude us from identifying ions at *m/z* values corresponding to the product of these chimeric assembly lines.

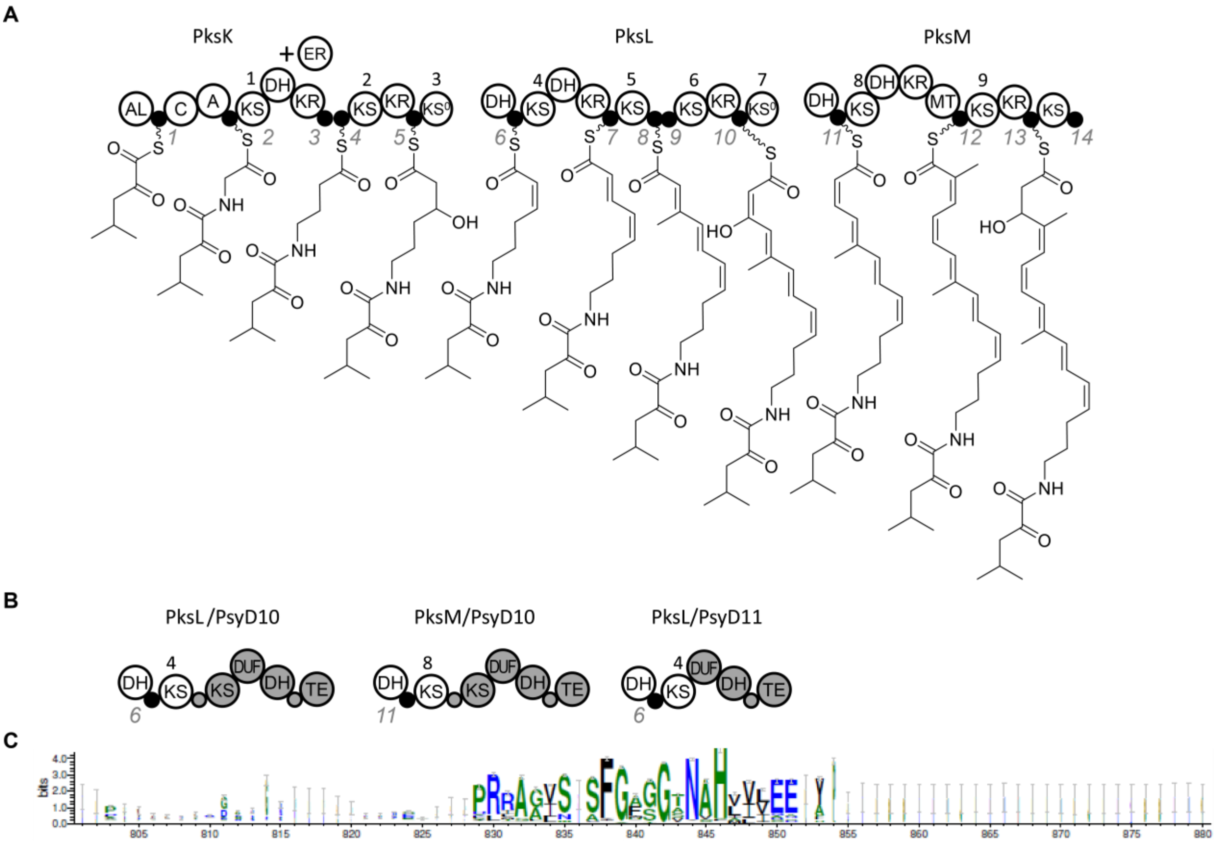

Figure S1 Overview of chimeric *trans*-AT PKSs produced by exchange of terminal domains. (**A**) Relevant section of the bacillaene *trans*-AT PKS from *B. subtilis*. KS numbers are indicated in black above the KS domains. ACP numbers are indicated in italic and gray below the assembly line. (**B**) Chimeric PKSs created by fusion of either KS4 (located in PksL) or KS8 (PksM) with terminal domains of the psymberin (*psy*), onnamide (*onn*) *trans*-AT PKSs. AL: acyl ligase, C: condensation domain, A: adenylation domain, KS: ketosynthase, DH: dehydratase, ER: enoylreductase, KR: ketoreductase, KS^0: non-elongating ketosynthase, MT: methyltransferase, filled circles: carrier protein, DUF: domain of unknown function, TE: thioesterase. (**C**) WebLogo representation of the conserved NAHVILEE motif downstream of the KSs.

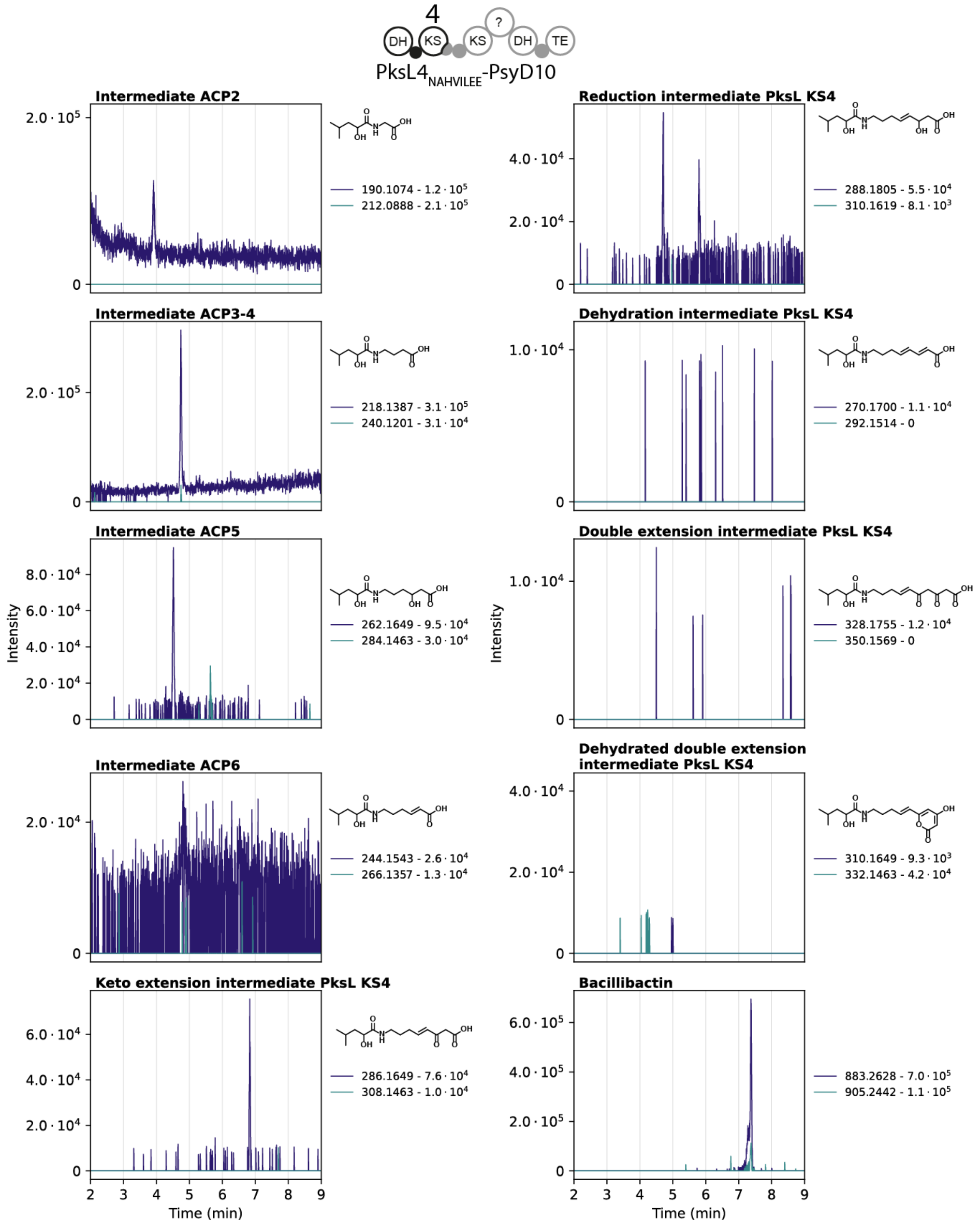

Figure S2 EICs for ions of various intermediates and potential chimeric *trans*-AT PKS products were obtained from HPLC-MS traces of extracts of *B. subtilis* encoding the PksL4$_{NAHVILEE}$-PsyD10 chimeric PKS. The dark blue traces indicate the proton adduct and the light blue traces indicate the sodium adduct, for which the masses are indicated in the legend of each subplot. The maximum intensity in the respective EICs is also indicated in each legend. Putative structures of intermediates and products corresponding to the *m/z* values of interest, as obtained from the biosynthetic scheme in Fig. S1 are depicted next to each EIC.

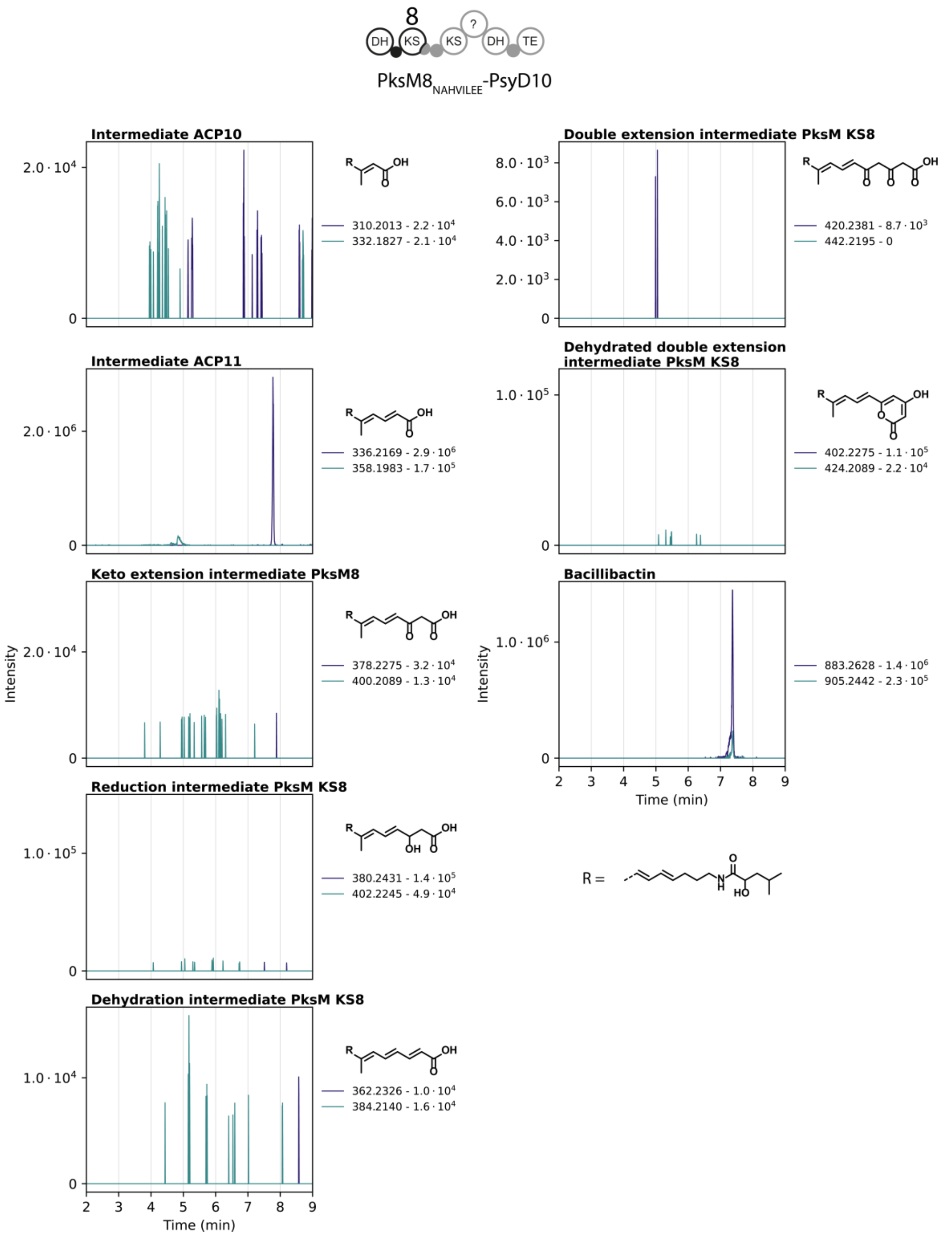

Figure S3 EICs for ions of various intermediates and potential chimeric *trans*-AT PKS products were obtained from HPLC-MS traces of extracts of *B. subtilis* encoding the PksM8$_{NAHVILEE}$-PsyD10 chimeric PKS. The dark blue traces indicate the proton adduct and the light blue traces indicate the sodium adduct, for which the masses are indicated in the legend of each subplot. The maximum intensity in the respective EICs is also indicated in each legend. Putative structures of intermediates and products corresponding to the *m/z* values of interest, as obtained from the biosynthetic scheme in Fig. S1 are depicted next to each EIC.

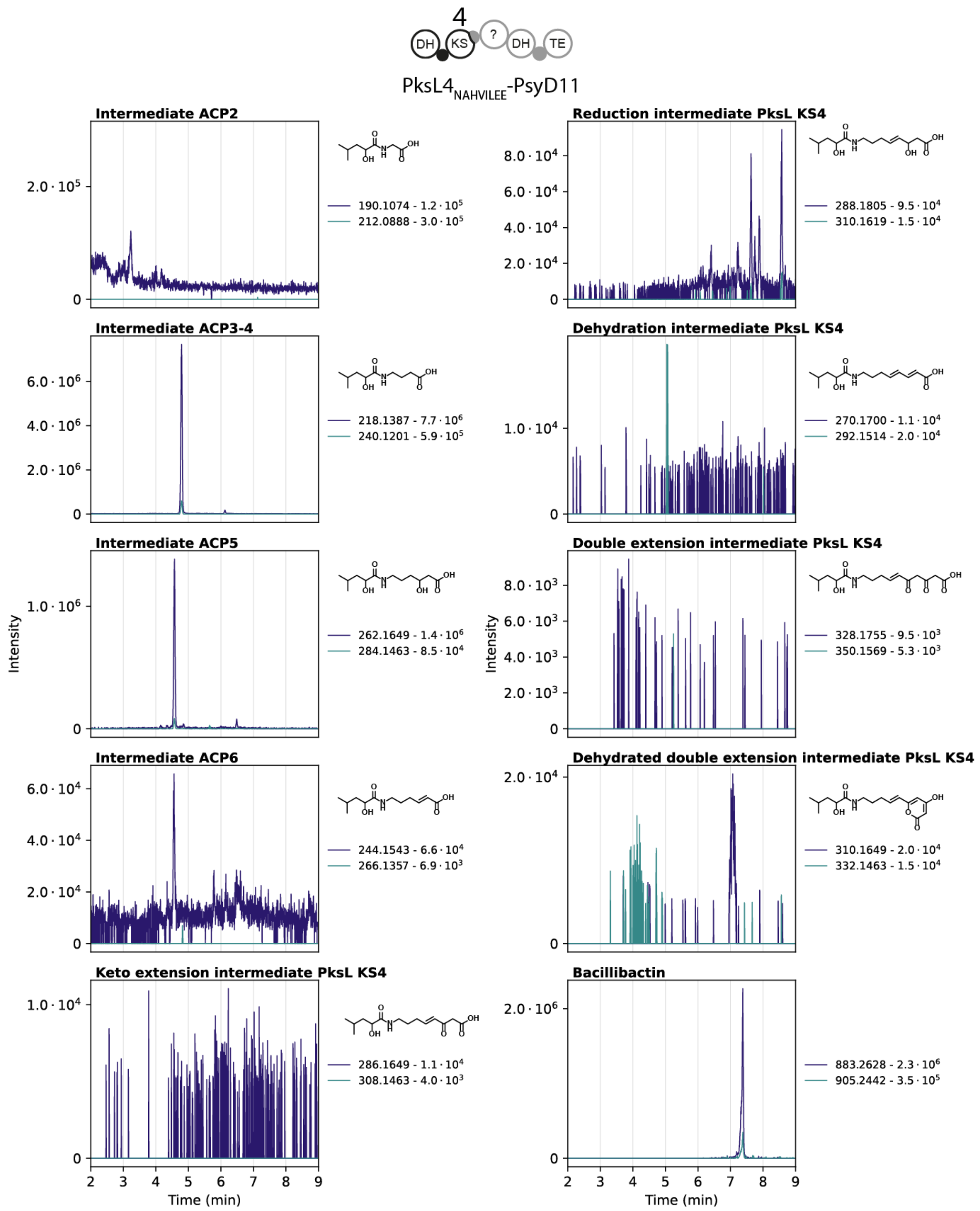

Figure S4 EICs for ions of various intermediates and potential chimeric *trans*-AT PKS products were obtained from HPLC-MS traces of extracts of *B. subtilis* encoding the PksL4$_{\text{NAHVILEE}}$-PsyD11 chimeric PKS. The dark blue traces indicate the proton adduct and the light blue traces indicate the sodium adduct, for which the masses are indicated in the legend of each subplot. The maximum intensity in the respective EICs is also indicated in each legend. Putative structures of intermediates and products corresponding to the *m/z* values of interest, as obtained from the biosynthetic scheme in Fig. S1 are depicted next to each EIC.

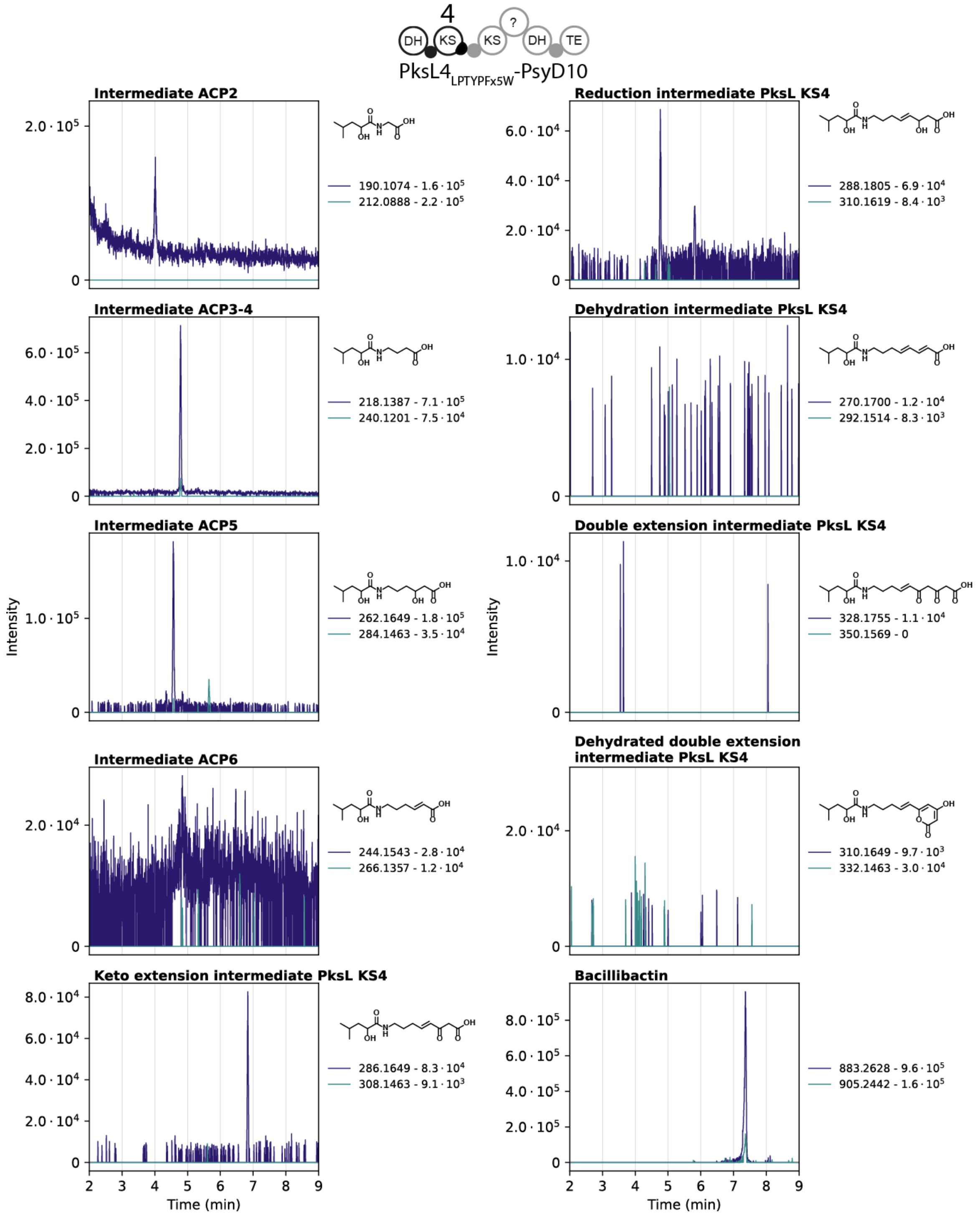

Figure S5 EICs for ions of various intermediates and potential chimeric trans-AT PKS products were obtained from HPLC-MS traces of extracts of B. subtilis encoding the $\text{PksM8}_{\text{NAHVILEE}}$-PsyD10 chimeric PKS. The dark blue traces indicate the proton adduct and the light blue traces indicate the sodium adduct, for which the masses are indicated in the legend of each subplot. The maximum intensity in the respective EICs is also indicated in each legend. Putative structures of intermediates and products corresponding to the *m/z* values of interest, as obtained from the biosynthetic scheme in Fig. S1 depicted next to each EIC.

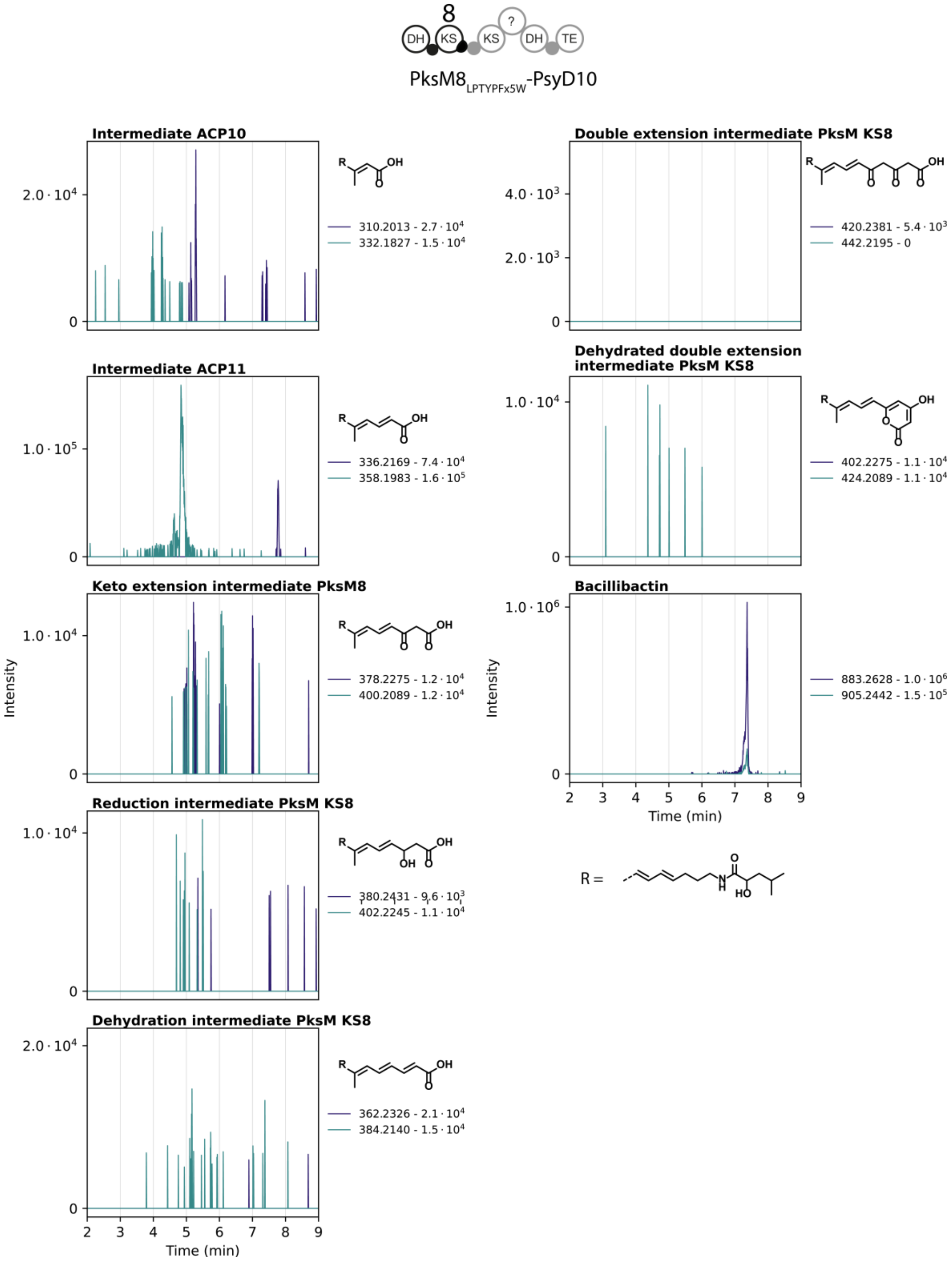

Figure S6 EICs for ions of various intermediates and potential chimeric *trans*-AT PKS products were obtained from HPLC-MS traces of extracts of *B. subtilis* encoding the PksM8$_{LPTYPFx5W}$-PsyD10 chimeric PKS. The dark blue traces indicate the proton adduct and the light blue traces indicate the sodium adduct, for which the masses are indicated in the legend of each subplot. The maximum intensity in the respective EICs is also indicated in each legend. Putative structures of intermediates and products corresponding to the *m/z* values of interest, as obtained from the biosynthetic scheme in Fig. S1 are depicted next to each EIC.

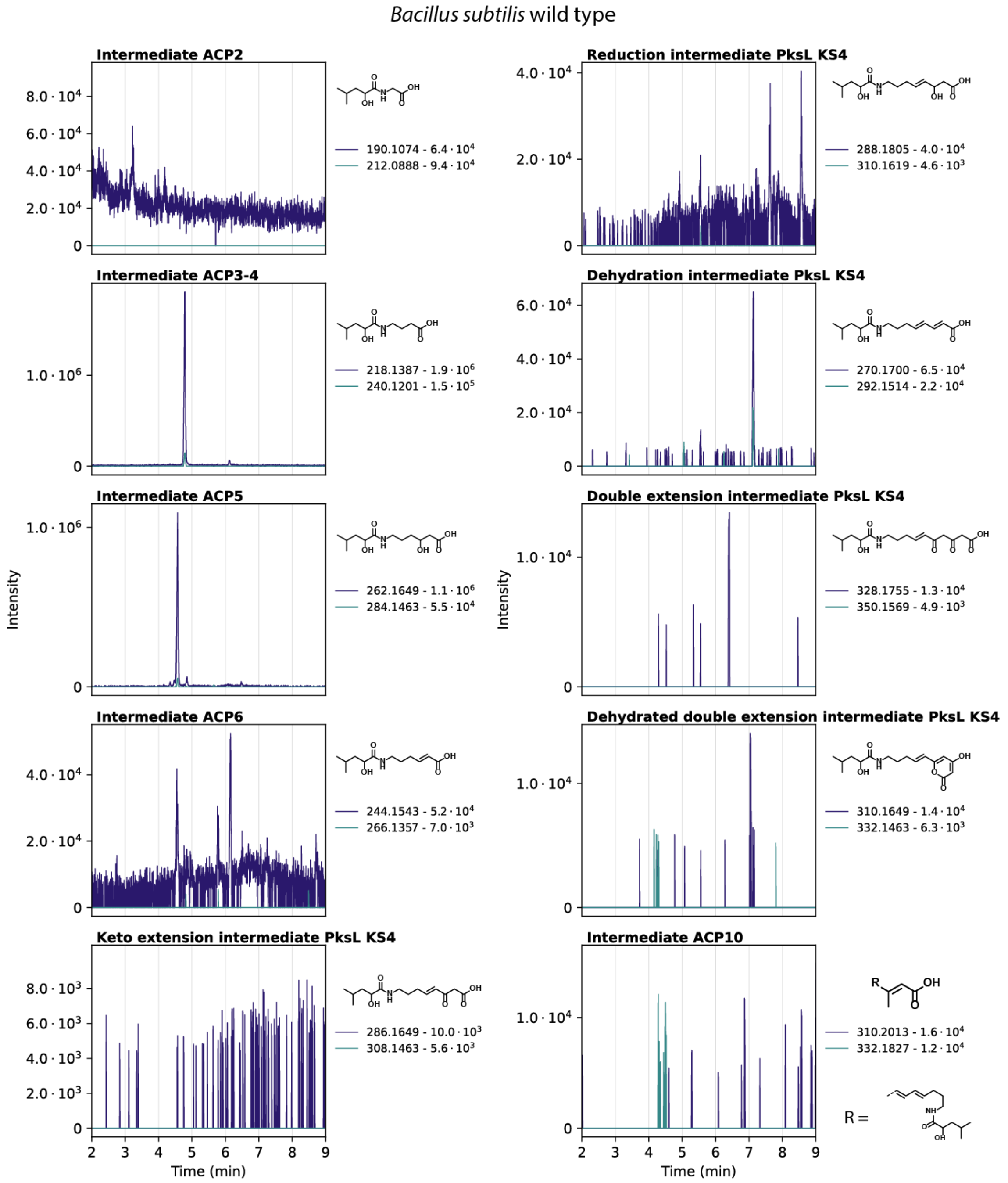

Figure S7 EICs for ions of various intermediates and potential chimeric *trans*-AT PKS products were obtained from HPLC-MS traces of extracts of wild type *B. subtilis*. The dark blue traces indicate the proton adduct and the light blue traces indicate the sodium adduct, for which the masses are indicated in the legend of each subplot. The maximum intensity in the respective EICs is also indicated in each legend.

Figure S7 continued

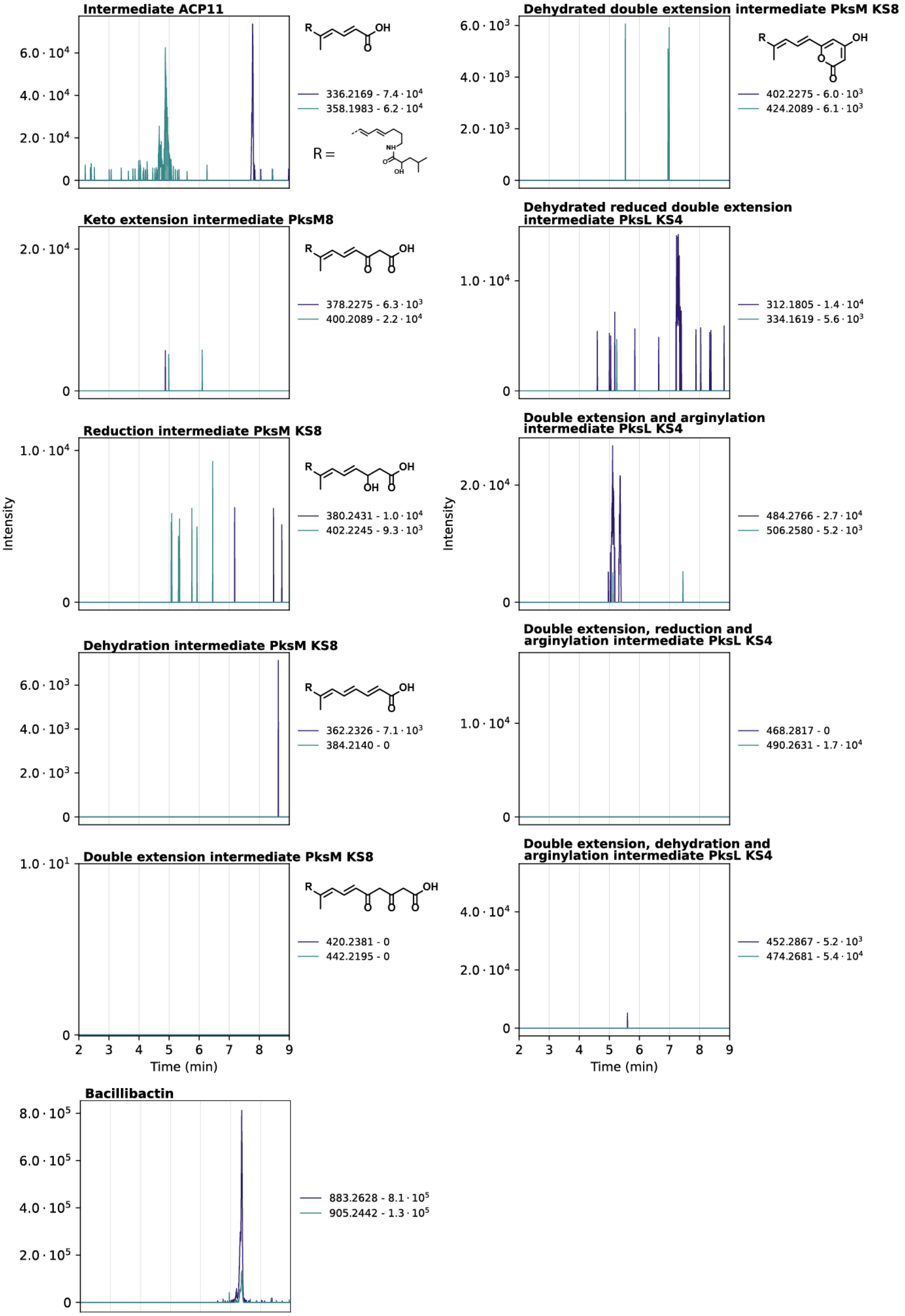

Additional supplementary figures

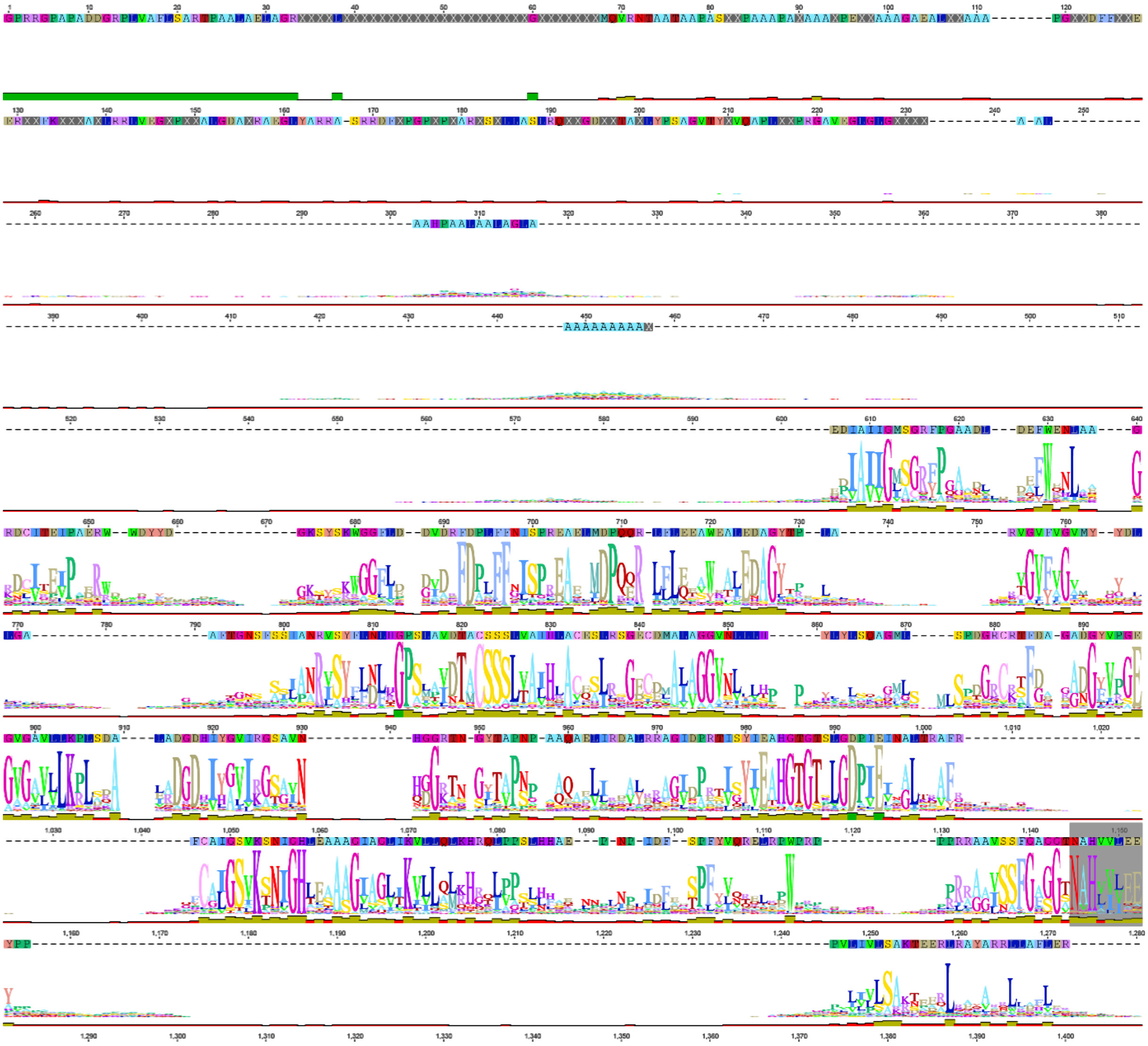

Figure S8 WebLogo representation of manual alignment of extended KS sequences. The NAHVILEE and LPTYPFx$_5$YW motif are indicated as shaded areas.

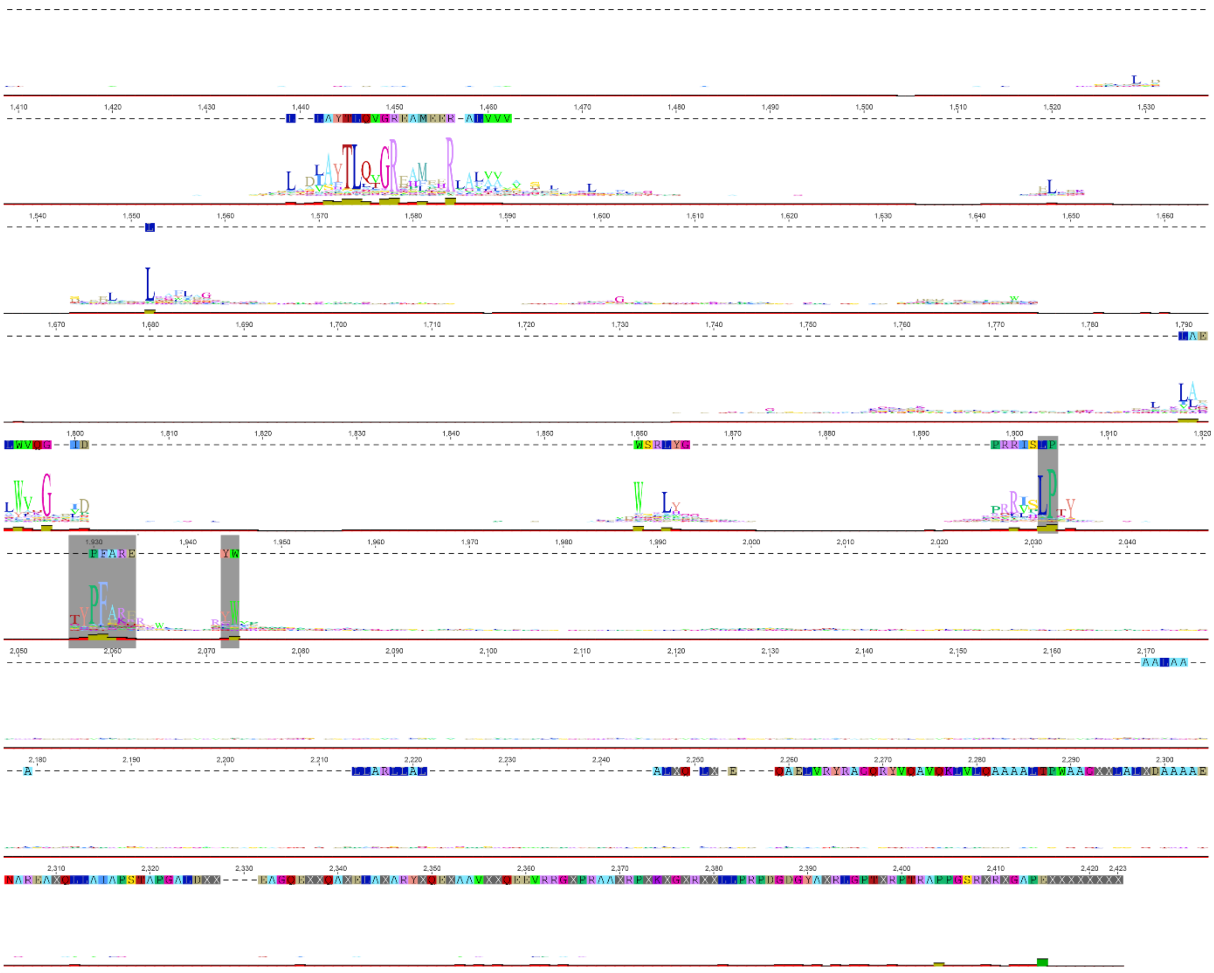

Figure S8 continued.

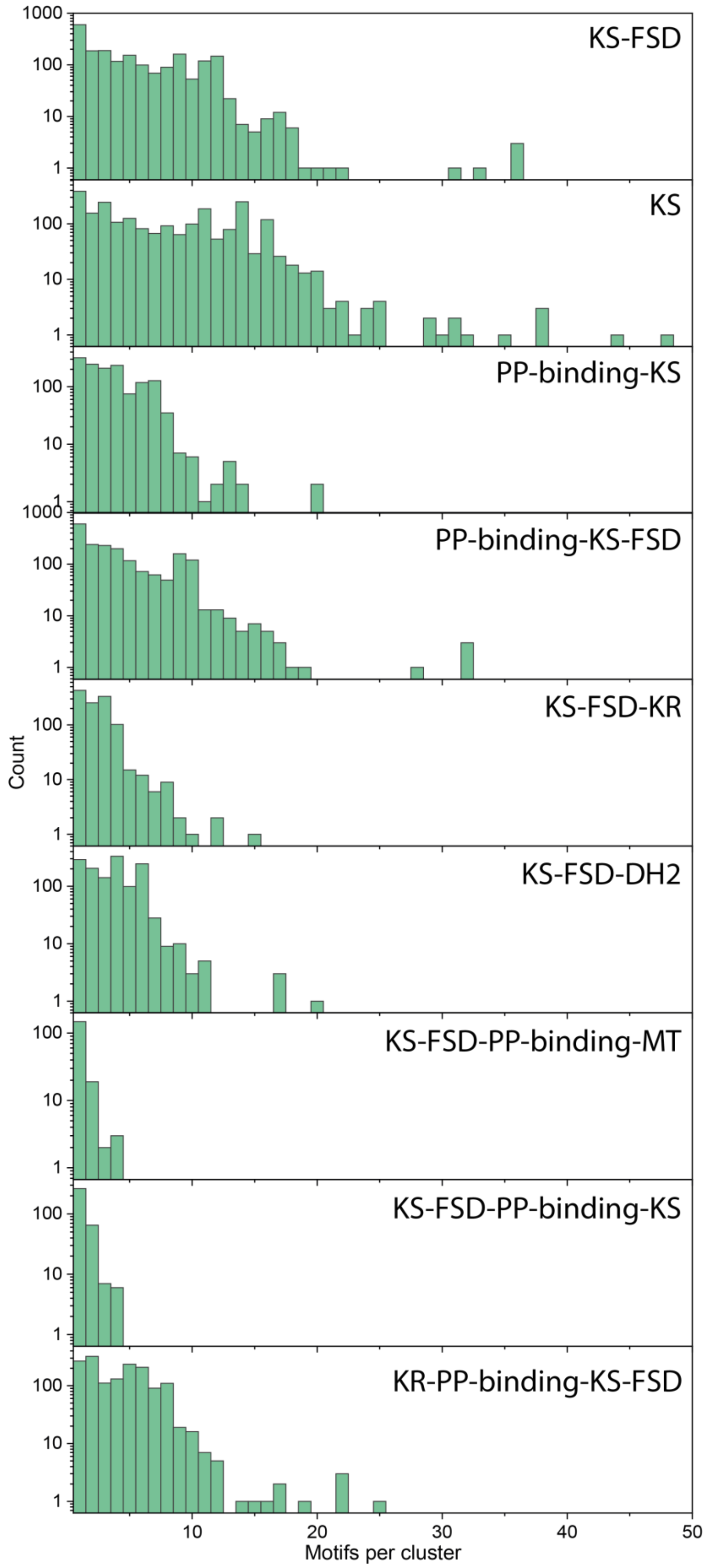

Figure S9 Histograms of various domain motifs in the BGCs used for the SCA analysis.

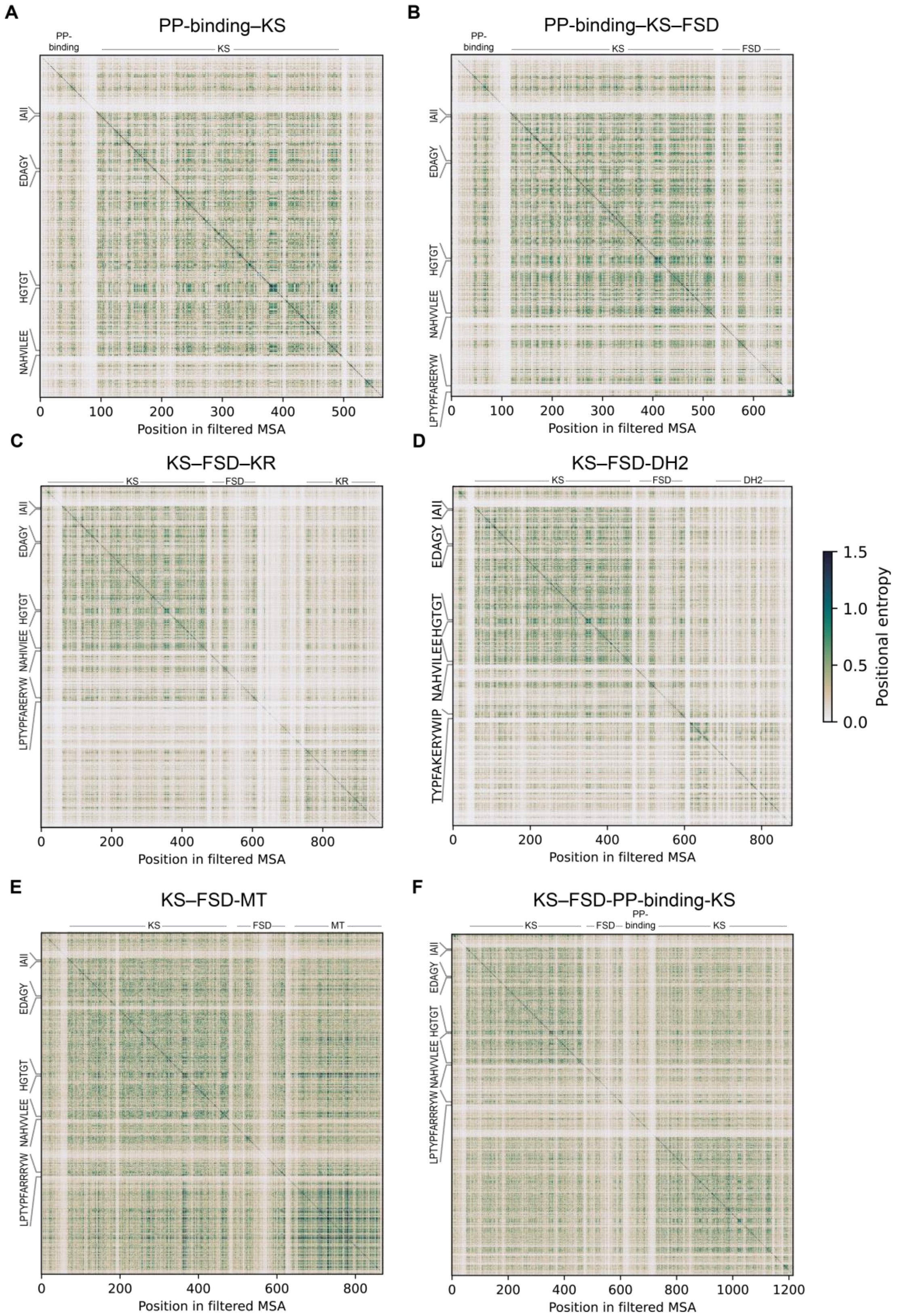

Figure S10 SCA matrices of Clustal alignments obtained of (**A**) PP-binding–KS, (B) PP-binding–KS–FSD, (**C**) KS–FSD–KR, (**D**) KS–FSD–DH2, (**E**) KS–FSD–MT and (**F**) KS–FSD–PP-binding–KS domain motifs. The IAII, EDAGY, HGTGT, and the consensus sequence of the NAHVILEE and LPTYPFx_5W motifs obtained from each MSA are indicated on the axes, indicating the N-terminus, active site and C-terminus of the KS domain and the C-terminus of the FSD, respectively.

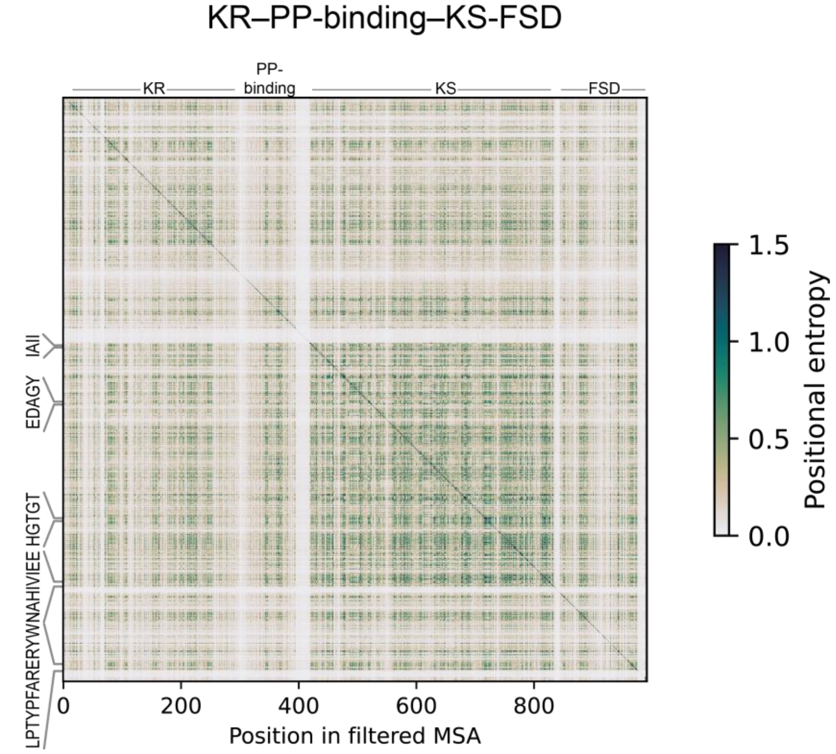

Figure S11 SCA matrices of a Clustal alignment obtained of a KR–PP-binding–KS–FSD domain motif. The IAII, EDAGY, HGTGT, and the consensus sequence of the NAHVILEE and LPTYPFx_5W motifs obtained from each MSA are indicated on the axes, indicating the N-terminus, active site and C-terminus of the KS domain and the C-terminus of the FSD, respectively.

PP-binding–KS

```
-DAASLREKVEAYLKELLAVLKIDADAPFEDYGIDSILLVELTNRLEKFLSPTLFFEYPTIAELAAYL
LEEHREALAALLG--PAAAAAAAADEDIAIIGMSGRYPGAENLDEFWENLKEGKDCITEIPADWDWRY
YDPGKTYSKWGGFIDDVDEFDPLFFGISPREAELMDPQERLFLETAWKAIEDAGYTPKSLSRVGVFVG
VMYGDYQLLGAATGSSPSSIANRVSYFLNLHGPSMAVDTACSSSLVAIHLACESLRRGECEMALAGGV
NLSLHPNKYIALSQAGMLSSDGRCKSFGAGADGYVPGEGVGAVLLKPLSDAEADGDHIYGVIKGSAVN
HGGKTNGYTAPNPKAQAELIREALDKAGIDPRTISYIEAHGTGTSLGDPIEINGLTKAFRQ-DKQFCA
IGSVKSNIGHLEAAAGIAGLIKVLLQMKHRTLVPSHSELNPNIDFEGSPFYVQQELREWKRP-ELPRR
AGVSSFGAGGTNAHVILEEYIPPAAA-AAGPALIVLSAKNEERLREYAERLLAFLEE-DIAQVGREAM
EERLAASSLEL-KLRAFLAG-
```

Sector 1	Sector 2	Sector 3	Sector 4	Sector 5	Sector 6
Sector 7	Sector 8	Sector 9	Sector 10	Sector 11	Sector 12

PP-binding–KS–FSD

```
AGLQ--A-AADEDALREKVEAYLKQLIAEVKIPAEKIDADAPLEEYGIDSIMLTELTNELEKTFLSPT
LFFEYQTIAELAAYLVEEHREALAALLGLEAKEAAA--AAPAAAAAPADEDIAIIGMSGRYPQAEDLE
EFWENLKEGKDCITEIPADRWDWREYDGKTYSKWGGFIDDVDKFDPLFFGISPREAELMDPQERLFLE
TAWEAIEDAGYTPESLA-KVGVFVGVMYTGYQLLGEALGSSPSSIANRVSYFLNLHGPSMAVDTACSS
SLTAIHLACESLRRGECEMAIAGGVNLSLHPNKYISLSQAGMLSSDGRCKSFGEGGDGYVPGEGVGAV
LLKPLSKAEADGDHIYGVIKGSAVNHGGKTNGYTVPNPNAQADLIKEALEKAGIDPRTVSYIEAHGTG
TSLGDPIEIAGLTKAFRE-TDKQFCAIGSVKSNIGHLESAAGIAGLTKVLLQMKHKTLVPSLHEELNP
YIDFEDSPFYVQQELEEWKRPVLPRRAGISSFGAGGANAHVVIEEYIPEAAE--AAEGPALIVLSAKN
EERLREYAEQLLAFLEEDADLADIAYTLQVGREAMEERLAFVASSLEELEEKLNAFLAGK-LGGEIEA
WIEKGKLAKLAELWVKDIDWSKYGKPRRISLPTYPFAKERYWIPDAAKAAAA-RFGFLPEMKNVWRP
```

Sector 1	Sector 2	Sector 3	Sector 4	Sector 5	Sector 6
Sector 7	Sector 8	Sector 9	Sector 10	Sector 11	Sector 12
Sector 13	Sector 14	Sector 15	Sector 16		

Figure S12 Color-coded consensus sequences of various domain motifs. SCA sectors are indicated with color coding of the residues, with sector mapping indicated below the consensus sequence. Residues that are not in a protein sector are colored black.

KS–FSD–KR

TVVELTNALNKAFGELSPTLFFEYPTIRALAEYLIKEHPDALIKLTGA-AAAAAADRAAEDIAIIGMS
GRYPGAKDLDEFWDNLAEGKDCITEIPKERWDWRAYYDPEKEGKTYCKWGGFLDDIDEFDPLFFNISP
REAELMDPQERLFLQTAWKAIEDAGYTPESLSKKTGVFAGVMNNEYGLLL-ATGNSFSIIANRVSYFL
NLKGPSIPVDTACSSSLVAIHLACEALRNGECDMAIAGGVNLYLTPESYISLSKAGMLSPDGRCKTFD
DGADGFVPGEGVGAVVLKPLSDAEADGDHIYGVIRGSAINHGGKTNGITAPNPKAQADLIREAYERAG
IDPRTISYIEAHGTGTKLGDPIEIEGLTKAFREDTRKQFCAIGSVKSNIGHLEAAAGIAGLTKVLLQM
KHRQLVPSLHFETLNPHIDFDDSPFYVQTELKEWERPLPRRAGVSSFGAGGTNAHIVIEEYQPKARA-
KADPPALIVLSAKNEERLKEYAEQLLDFLRQKDIDLADIAYTLQTGREAMEERLAFVAGSLEELEEKL
NAFLAGKTAGLYRGQM--LIDAWIRKGKYAKLAELWVKGLDIDWNRLYGKPRRISLPTYPFAKERYWL
PEPEAAPAAE-REVLLLQKQWEESPLPSAEEGTVAILATDETA-ELAFIIF-VTKGLEFANELVRLAG
ASRAGLYRMLQLEYPHLRSRHIDLDPATDLAKIIAEDSTEAEVCYRDGQRYRAVLEETPLEAKPSPFP
EGGVYLITGGTGGLGLLCARHLAERYVKLVLTGRSPLPPRIEAIQELELGQVLYLSADLSDPAAVRQA
LKRIKRTFGPIGGVIHCAGVVDAFIRKTAEDFQRVLEPKVAGLQTLDEALNEPLDFFVLFSSVSAIGA
GQSDYAMANAFMDAFAAYRNE-GPTVSINWPNWKEGGMGEVTLKSSGLRSLTNAEGLALLDRILRLLL
AGEPSR-GAPA-A--LAW

Sector 1	Sector 2	Sector 3	Sector 4	Sector 5	Sector 6
Sector 7	Sector 8	Sector 9	Sector 10	Sector 11	Sector 12
Sector 13	Sector 14	Sector 15	Sector 16	Sector 17	

KS–FSD–DH

DSILLVELANRNKEFGLTLLFEYPTIALAYLAEYAEAFA--E--APAAAEPKYADEDIAIIGISGRFP
GANIDEFWENLKEGKDCITEIPKDRWDWREYGEGKTYSKWGGFIDGVDCFDPLFFGISPREAELMDPQ
ERLFLETAWKAIEDAGYTPKSLGTKTGVFVGVMTDYRLLLAEEGSAVGASPSSIANRVSYFLNLHGPS
MAVDTACSSSLVAIHLACESLRSGECEMALAGGVNLSLHPNKYISLSKAGMLSSDGRCKTFGAGADGY
VPGEGVGAVLLKPLSDAEADGDHIYAVIKGSAVNHGGKTNGLTVPNPKAQADVIQAALDKAGIDPRTI
SYIEAHGTGTKLGDPIEINGLTKAFRQYTKQFCGIGSVKSNIGHLEAAAGIAGLIKVLLQLKHKTLVP
SLHSEELNPYIDFEDSPFYVVQETKEWKRPYPRRAGISSFGAGGTNAHVILEEYIPEESA-DAEGPAL
IVLSAKNEERLREYAKQLLAFLE-TDVDLADLAYTLQVGREAMEERLAFIASSIEELKEKLKAFLNGG
CYRGADGEEIEKWLAKGKLAKLAELWVGAVDWNKLYGKPRRISLPTYPFAKERYWIPDAEKKAS-GAA
VLHPLLHSDLSEQRFSSTFTGEEFFLADHVVKKRVLPGVAYLEMARAAVERAAGGSVIRLKNVVWVRP
IVVEEPEVHIRIAFEIYSE-EPVVHSQGSAVLVEAPVLDLELKQCQLSAEECYEAFRIGIDYGPAFRG
IEQLYIVLAKLSLPASVTKDQYVLHPSLLDSALQASGLLL-DNKLSLPFALEELEVGPPMWAYVRYSE
GVQKLDIDLCDENGRVCVRLKGFSSRALEGEA-GTLLLEPVWEEAPLEAE--EHIVVLCGDDE

Sector 1	Sector 2	Sector 3	Sector 4	Sector 5	Sector 6
Sector 7	Sector 8	Sector 9	Sector 10	Sector 11	Sector 12
Sector 13	Sector 14	Sector 15	Sector 16	Sector 17	

Figure S12 continued.

KS–FSD–MT

SISIVELNSRLN-LLGSLSPTLLFEYPTIAALAEHLAEGHPEAPAKWLG---A--ARPESANVGRDED
IAIIGMSGRFPGADDLEEFWENLAEGRDAIREIPAERWDWIAYYDGKSYSKWGGFLDDIDEFDPLFFN
ISPREAENMDPQQRLFLQEAWEALEDAGYSPKDLSGSRTGVFVGVTTQQYQLLLPEVESALPGNSFAS
LANRISYFLNLTGPSLAVDTACSSSLVAIHEACQSLRSGECEMALAGGVNLYLSPSSYVLSQAGMLSP
DGRCKAFDADADGFVPGEGVGAVVLKPLDQAVADGDHIYAVIRGSAVNHDGRTNGITAPNPAAQAAVI
REALERAGIDPETISYIEAHGTGTRLGDPIEVEALTEAFGKYTDKQFCAIGSVKSNIGHLEAAAGIAG
LIKVVLAMKHRQIPPTLHFKEPNPHIDFANSPFYVNRELKEWPPGPRRAGVSSFGAGGTNAHVVLEEY
PGPREASASGPYLFVLSAKTEERLRAYAERLLAFLKAHLDLADIAYTLQVGREAMACRLAFVANSVEE
LIQKLERFLEGGD-AG-ARTKEGGSPAPVRRAIEEGDLEELAKLWVQGVDIDWAALYGPRRVSLPTYP
FARRRYWIEPKAQQGPAASGFGIVERYERWLQAGYLEFDEEYLTLAALPALFPGGSMELVEGFYKGNP
DYYNLVLAEQVEQRADFSRRIRILEIGAGTGGTSIQLAALAPHQVEYCYDISKAFLLHAERRFAPAYP
FVRFQIFNIESSEQGFPPQYDIVIAANVLHATRDIRATLSHVKALLAPGGLLLLNELVANSLFSFEDT
DLGSPLLTPETWEQLLREEGFTIVEIPDVSAPELGQQILLAFNDSGVAGQTPI-

Sector 1 Sector 2 Sector 3 Sector 4 Sector 5 Sector 6
Sector 7 Sector 8

KS–FSD–PP-binding-KS

DRLPGPPTLFFEYGTLGGLAGYLRDSHLNALVRLPGFDDPRAAAHAGACADDIAIIGLSGRYPGAPTL
DAFWRNLVAGRDSISEIPAERWDWRDHYEPDPTHGKSYGKWGGFLDGFDAFDPLFFQISPREAEFMDP
QERLFLEACWHALEDAGYPPEALQGARVGVFVGVTKQGYNLYGAGGAYQGTSLASLANRVSYFLDFNG
PSVAFDTMCSSSLVAIHEACQSLRRGECEIAIAGGVNLNLHPSNYQQLSKAGMLSSDGRCRSFGSGGD
GYVPGEGVGAVVLKPLRRALADGDPIYGVIRGSAVNHGGRTNGFTVPSPKAQAAVIRAALARAGVDPR
SISYVEAHGTGTALGDPIEIAALTRAFGARTRDGRCAIGSVKSNIGHLEAAAGIAGLTKVLLQLRHGQ
LPPSLHCEALNPDIDFDATPFEVQRELAPWARARVPRRAGISSFGAGGSNAHLVLEEYAAPAVPEADA
GPHLFVLSARTRERLRDYARDLLAFLNDDLADIAYTLQVGREAMACRLAVVAADLRELAGKLARFLEG
AHDGVFRGEARAAHREAARDARELRDLARLWVGGAVVDWAARHAPPRRISLPTYPFARRRYWPGAAAA
AAAAPATDAAARLEAALLPRLRELVADVLRLPADELDADRPFDEYGLDSILIVELNVRLNEILGRLSS
TLFFEYRTAGELARHLLTAHRDACAAWVFDGSSSIKRPGATWDEPIAIVGMSGRFPQARDLDAFWDNL
ARGRDSITEIPPERWDLDGGRSYCKWGGFIDGVDEFDPLFFNISPREAENMDPQQRLFLEEAWKALED
AGYTRARLAGGRCGVFVGITRGEYQLLGAGNLQGKATGNSFSSLANRVSYFLDLNGPSIAVDTACSSS
LVAVHLACDSLRSGECEVALAGGVNLSLHPYMYVSLSAAGMLSSDGRCKTFDAGADGYVPGEGVGVVV
LKPLSDALADGDRIHGVIRGSGVNHGGKTNGYTAPNPIAQQALIRSALDRAGIDPRTISYVEAHGTGT
ELGDPIEIAGLSRAFRRDTSDRGFCAIGSVKSNIGHLEAAAGIAGLTKVLLQMKHGQLPPSLHAEELN
PNIDFPASPFYVNRETRPWERPVEHPRRAGVSSFGAGGTNAHVVLEEYPRQASAGAPALIVLSAKTPE
QLRRYASRLLARLRDADYARLAYTLQVGREAMEERLAVVADSVQELEGKLRQFLDGKTD

Sector 1 Sector 2 Sector 3 Sector 4 Sector 5 Sector 6
Sector 7 Sector 8 Sector 9 Sector 10 Sector 11 Sector 12
Sector 13 Sector 14 Sector 15 Sector 16 Sector 17 Sector 18
Sector 19 Sector 20 Sector 21 Sector 22

Figure S12 continued.

KR–PP-binding–KS

LIQIVEQQLLSGLSGLLKTAGLENPKLTGQLIEIEPEESLAEILREPDDVHIRYERYVADWKETD-EA
DVPWKDGGVYLITGGAGGLGLIFAKEIARTKDATLILTGRSPLDEDKKQLEELGARVEYRQADVTDKE
AVERLIAEIKEYGGLNGIIHSAGVIRDSYILKKTAEEFQQVLAPKVAGTVNLDEATKDLPLDFFILFS
SISGVLGNAGQADYAAANAFMDAYAAYRNALVRHGKTLSINWPLWKGMQVDAEAMLLKESGMVPLETE
EGIQALYQALSGVLVLEGDRARIALLQTASAAEIDEDSLLEKVEHYLKQLISELLKLPAEKIDADAPL
EDYGFDSIMITELTNKLEKTFGLSKTLFFEYQTIRELAGYLLEEHREALAALLGAAAKEAQAA-P---
PVAAQAKQHPEPIAIIGISGRYPQADDLDEFWENLKEKDCITEIPKDRWDWREYYGDD-EAGKTYSKW
GGFIDGVDEFDPLFFGISPREAELMDPQERLFLETAWKAIEDAGYTRESLSGTKVGVFVGVMYTGYQL
LGAEI-AAGSSPSSIANRVSYFLNLHGPSMAIDTACSSSLVAIHLACESLRRGECEMAIAGGVNLSLH
PNKYIALSQAGMLSSDGRCKSFGEGGDGYVPGEGVGAVLLKPLSKAEADGDHIYGVIKGSAVNHGGKT
NGYTVPNPKAQAEVIKEALKKAGIDPRTVSYIEAHGTGTELGDPIEINGLTKAFSELTQFCAIGSVKS
NIGHLEAAAGIAGLTKVLLQMKHKTLVPSLHSETLNPIDFEDSPFYVQQELEEWKRPRELPRRAGISS
FGAGGVNAHVVIEEYIPEADE--EEGPQLIVLSAKNEERLKEYARRLLDATDADLADIAYTLQVGREA
MEERLAIVASSLEELEEKLKAFLAGK-EGLYRGDEE-QAALEAWIERGKLAKLAELWVKGDIDWDKLY
GGKPRRISLPTYPFAKERYWIPDSAQAAEAA-ADHVMAAW

Sector 1	Sector 2	Sector 3	Sector 4	Sector 5	Sector 6
Sector 7	Sector 8	Sector 9	Sector 10	Sector 11	Sector 12
Sector 13	Sector 14	Sector 15	Sector 16	Sector 17	Sector 18
Sector 19	Sector 20				

Figure S12 continued.

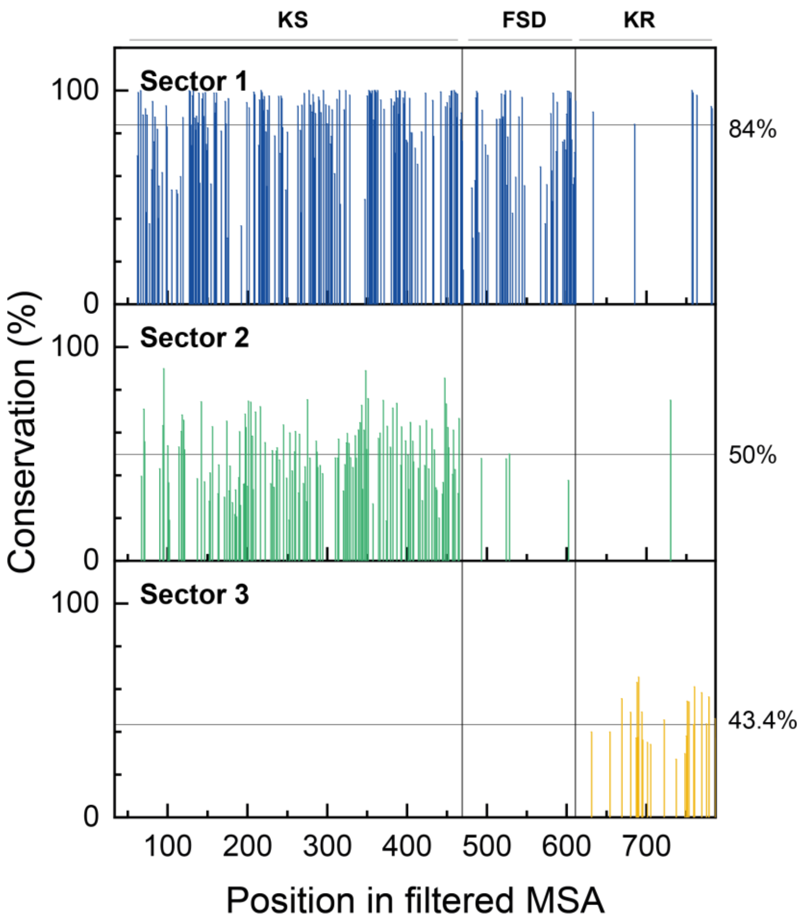

Figure S13 Positional conservation of the amino acid positions in the filtered MSA for sectors 1, 2, and 3 as obtained from the SCA of the Clustal alignments of the KS–FSD–KR tridomain. The consensus sequence can be found in Fig. S12. The horizontal dashed lines indicate the average conservation in the sector.

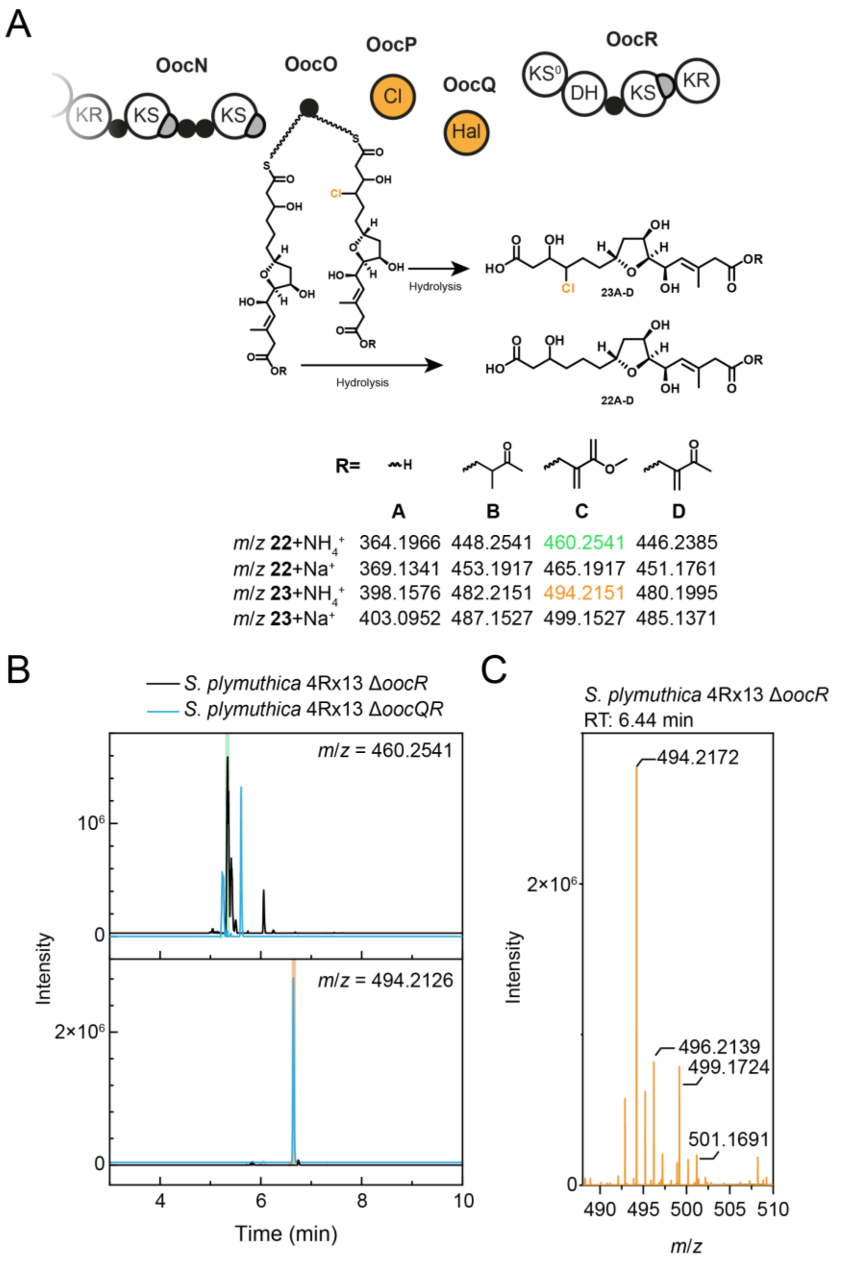

Figure S14 (**A**) The polyketide intermediate attached to the OocO ACP can be halogenated by OocPQ. Hydrolysis of non-chlorinated and chlorinated intermediates results in the release of congeners **22** and **23**, respectively. (**B**) EICs for ions of the ammonium adducts of **22C** (top) and **23C** (bottom) from expression cultures of *S. plymuthica* 4Rx13 Δ*oocR* and *S. plymuthica* 4Rx13 Δ*oocQR*. (**C**) Zoom of the mass spectrum at a retention time of 6.44 minutes from HPLC-MS analysis of an expression culture of *S. plymuthica* 4Rx13 Δ*oocR*, showing that the ion with an *m/z* corresponding to **23C** is chlorinated. The absence of this signal in the *S. plymuthica* 4Rx13 Δ*oocQR* mutant indicates that knocking out and supplementing *oocQ* suffice to abolish and restore chlorination of polyketide intermediates, respectively. For these measurements, a solvent gradient (A = H_2O + 0.1% formic acid and B = acetonitrile + 0.1% formic acid) with B at 1% for 0–2 min, 5–50% for 2–4 min, 50–95% for 4-10 min and 95% for 10-13 min at a flow rate of 1.0 mL/min) was used on a Phenomenex Kinetex 2.6 µM C18 100A (150 × 4.6 mm) column at 27 °C.

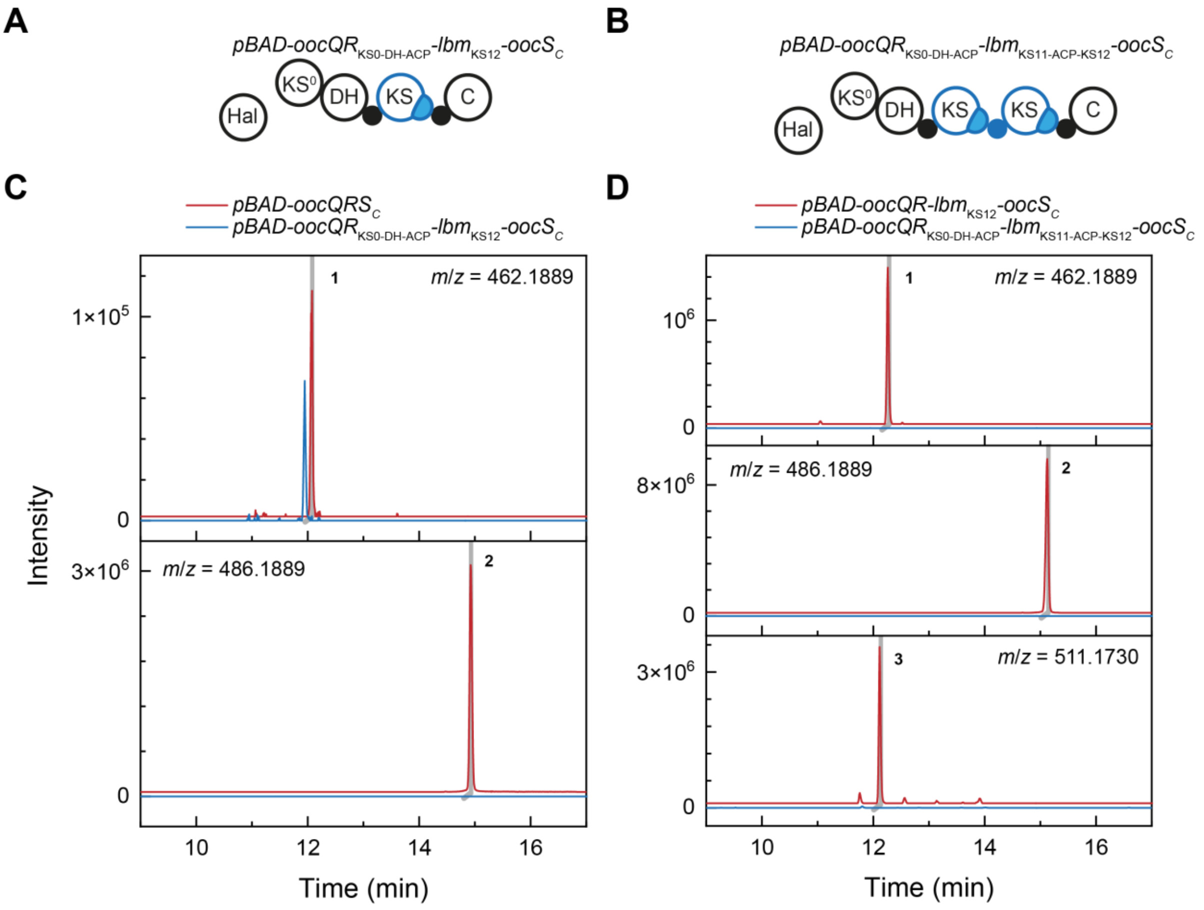

Figure S15 (**A**) Domain architecture of the modules encoded by the *S. plymuthica* 4Rx13 mutant harboring the *pBAD-oocQR*$_{KS0\text{-}DH\text{-}ACP}$*-lbm*$_{KS12}$*-oocS*$_C$ plasmid. (**B**) Domain architecture of the modules encoded by the *S. plymuthica* 4Rx13 mutant harboring the *pBAD-oocQR*$_{KS0\text{-}DH\text{-}ACP}$-*lbm*$_{KS11\text{-}ACP\text{-}KS12}$-*oocS*$_C$ plasmid. In these constructs, the foreign domains are inserted downstream of the ACP, rather than downstream of the KS-FSD. This fusion site is employed in a productive truncated disorazol PKS, reported by Wang et. al. (*24*). (**C**) EICs of ions corresponding to ammonium adducts of **1** and **2**, as obtained from expression cultures of *S. plymuthica* Δ*oocQR* supplemented with *pBAD-oocQR*$_{KS0\text{-}DH\text{-}ACP}$-*lbm*$_{KS12}$-*OocS*$_C$ and *pBAD-oocQRS*$_C$ (domain architecture in Figure 2 in main text) . (**D**) EICs of ions corresponding to ammonium adducts of **1** and **2**, as obtained from expression cultures of *S. plymuthica* Δ*oocQR* supplemented with *pBAD-oocQR*$_{KS0\text{-}DH\text{-}ACP}$-*lbm*$_{KS11\text{-}ACP\text{-}S12}$*OocS*$_C$ and *pBAD-oocQR-lbm*$_{KS12}$-*oocS*$_C$ (domain architecture in Figure 2 in the main text).

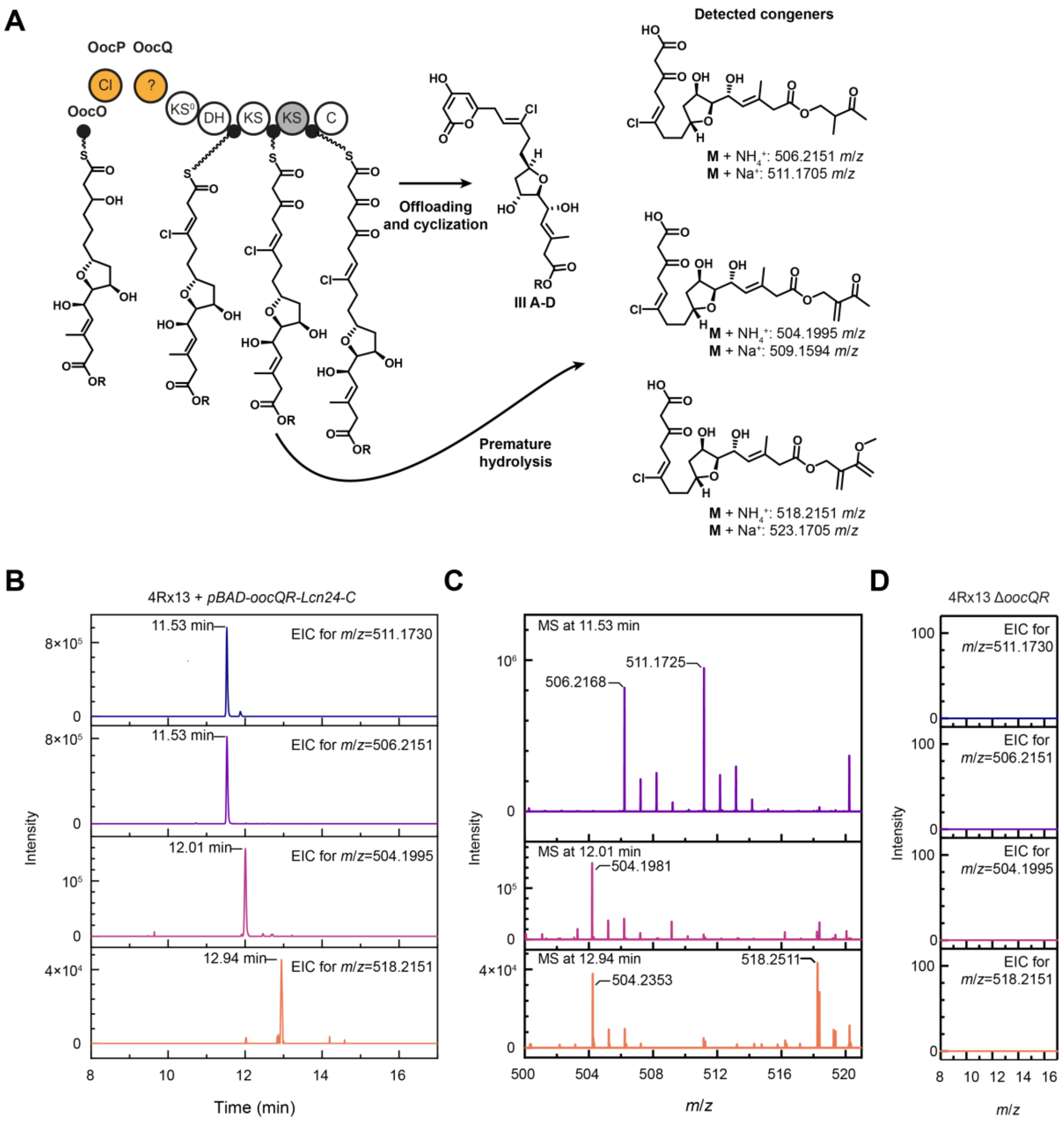

Figure S16 (**A**) Hydrolysis of stalled intermediates in chimeric assembly lines that incorporate a foreign minimal ACP-KS domain series (e.g., OocQR-Lcn24-ACP-C) leads to premature offloading from the assembly line, producing the carboxylic acids shown on the right, whereas normal processing by the foreign minimal ACP-KS domain series and offloading results in biosynthesis of **3** and related congeners (**III A-D**) (see Table S12). (**B**) Extracted ion chromatograms of an expression culture extract of *S. plymuthica* 4Rx13 + *pBAD-oocQR-lcn24-C* of ions of the various prematurely offloaded polyketide congener adducts. (**C**) Mass spectra at various elution times of the expression culture extract of *S. plymuthica* 4Rx13, indicating that the compounds with *m/z* values corresponding to congener **II** adducts are chlorinated. (**D**) EICs for ions of the various prematurely hydrolyzed polyketide congeners from an expression culture of *S. plymuthica* 4Rx13 Δ*oocQR*.

Structure elucidation of 2 and 3

Compound **2** with the molecular formular $C_{23}H_{29}ClO_8$ and 9 degrees of unsaturation was established from the HR-MS data HRMS (*m/z* 469.1633 $[M+H]^+$, Δ 0.98 mmu). Although the parent ion *m/z* intensity was very low, the ammonium ion adduct was the most intense (*m/z* 486.1905 $[M+NH_4]^+$).

The substructure II spin system was established from the COSY correlations of H_2-8 to H_2-7/H-9, H_2-10 to H-9/H-11/ H-12 to H-11/H- and H-13 to H-12/H-14. This assignment was supported by the HMBC correlations of H_2-8 to C-7/C-9, H_2-10 to C-8/C-9/C-11/C-12, H-11 to C-10/C-12/C-13 and H-12 to C-10/C-11. The downfield shift of C-12 (δ 83.5) together with the HMBC cross peaks observed between H-9 and C-12 confirm the oxolane ring. Substructure III was deduced and connected to substructure II from the HMBC correlations of H_3-23 to C-14/C-15/C-16, H_2-16 to C-14/C-15/C-17/C-23 and H-13 to C-11/C-12/C-14/C-15. The substructure I was established from the HMBC correlations of H_2-2 to C-1/C-3/C-4, H_2-4 to C-2/C-3/C-5/C-6. This substructure was connected to II based on the HMBC correlations of H-5 to C-6/C-7and H_2-7 to C-5. The macrolide ring was deduced based on the downfield shift of H-13 (δ 6.18) and the HMBC correlation of this proton to C-1 (δ 169.1). The substructure IV was elucidated from the HMBC correlations of H_3-21 to C-19/C-20, H_2-22 to C-18/C-19-C-20 and H_2-18 to C-19/C-20/C-22. This substructure was connected to III based on the HMBC correlations of H_2-18/H_2-16 to the carbonyl carbon C-17.

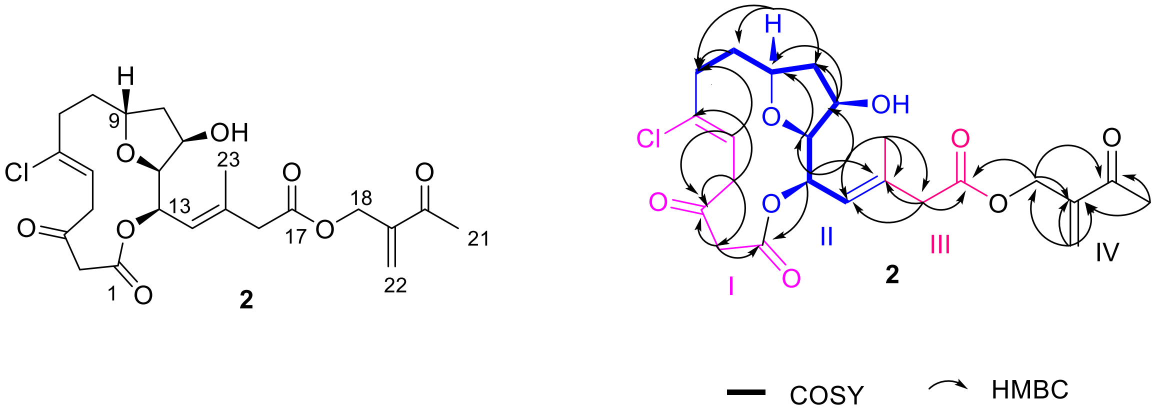

Figure S17 Structural assignment of **2.**

Table S13 NMR data for compound **2** in methanol-d$_4$ (^{1}H 500 MHz, ^{13}C 125 MHz).

No.	^{13}C/HSQC	^{1}H
1	169.1, C	
2	48.3, CH_2	3.24 (d), *J*=15.8 Hz
		3.74 (d), *J*=15.8 Hz
3	202.6, C	
4	45.4, CH_2	3.02 (m)
		3.57 (dd), *J*=10,4, 16.2 Hz
5	121.0, CH	5.72 (m)
6	139.6, C	
7	36.9, CH_2	2.46 (ddd), J=2.4, 5.0, 13.9 Hz
		2.77 (ddd), *J*=4.6, 12.6, 14.1 Hz
8	31.4, CH_2	1.52 (m)
		2.10 (m)
9	76.4, CH	4.14 (m)
10	44.5, CH_2	1.70 (td), *J*=5.1, 12.1 Hz
		1.94 (dd), *J*=3.7,12.6 Hz
11	73.6 CH	4.56 (td), *J*=2.0, 2.6, 5.4 Hz
12	83.5 CH	4.09 (t), *J*=5.5 Hz
13	73.0, CH	6.18 (t), *J*=5.0 Hz
14	124.9, CH	5.69 (m)
15	136.6, C	
16	45.4, CH_2	3.11 (s)
17	172.3, C	
18	63.2, CH_2	4.77 (q), J=1.3 Hz
19	144.6, C	
20	200.0, C	
21	25.9, CH_3	2.36 (s)
22	128.4, CH_2	6.06 (t); *J*=1.4 Hz
		6.32 (t), *J*=1.4 Hz
23	17.1, CH_3	1.79 (d), *J*=1.3 Hz

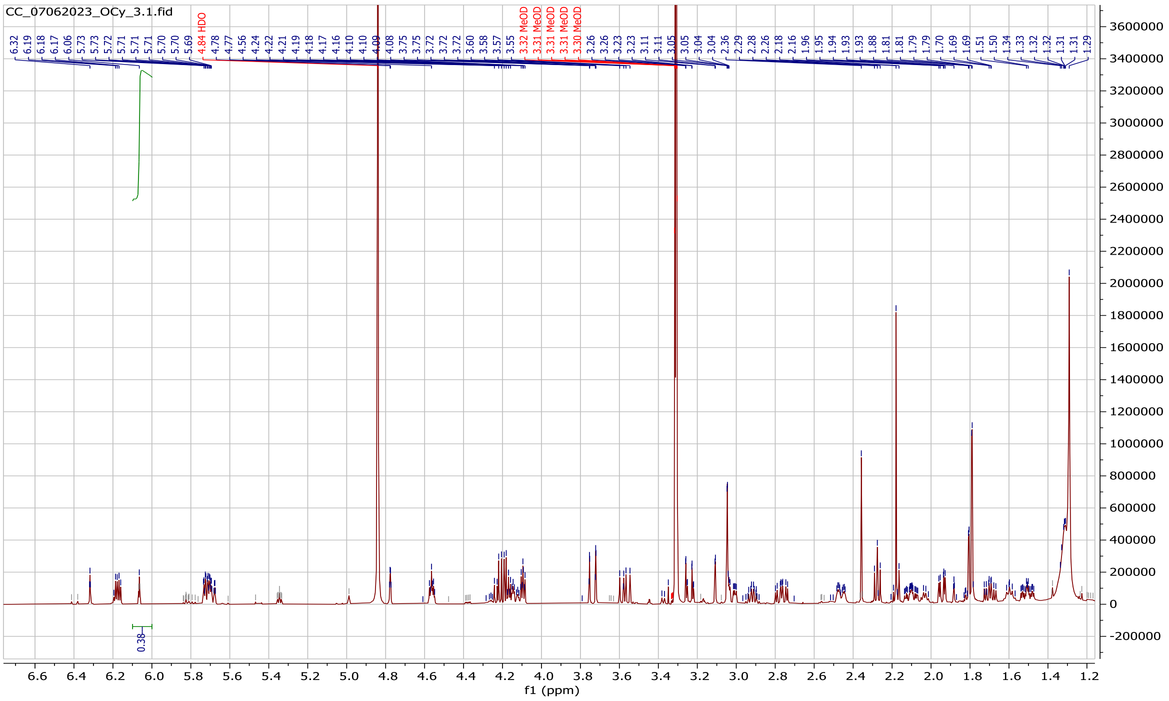

Figure S18 ^1H NMR spectrum of **2** in methanol-d_4 (^1H 500 MHz).

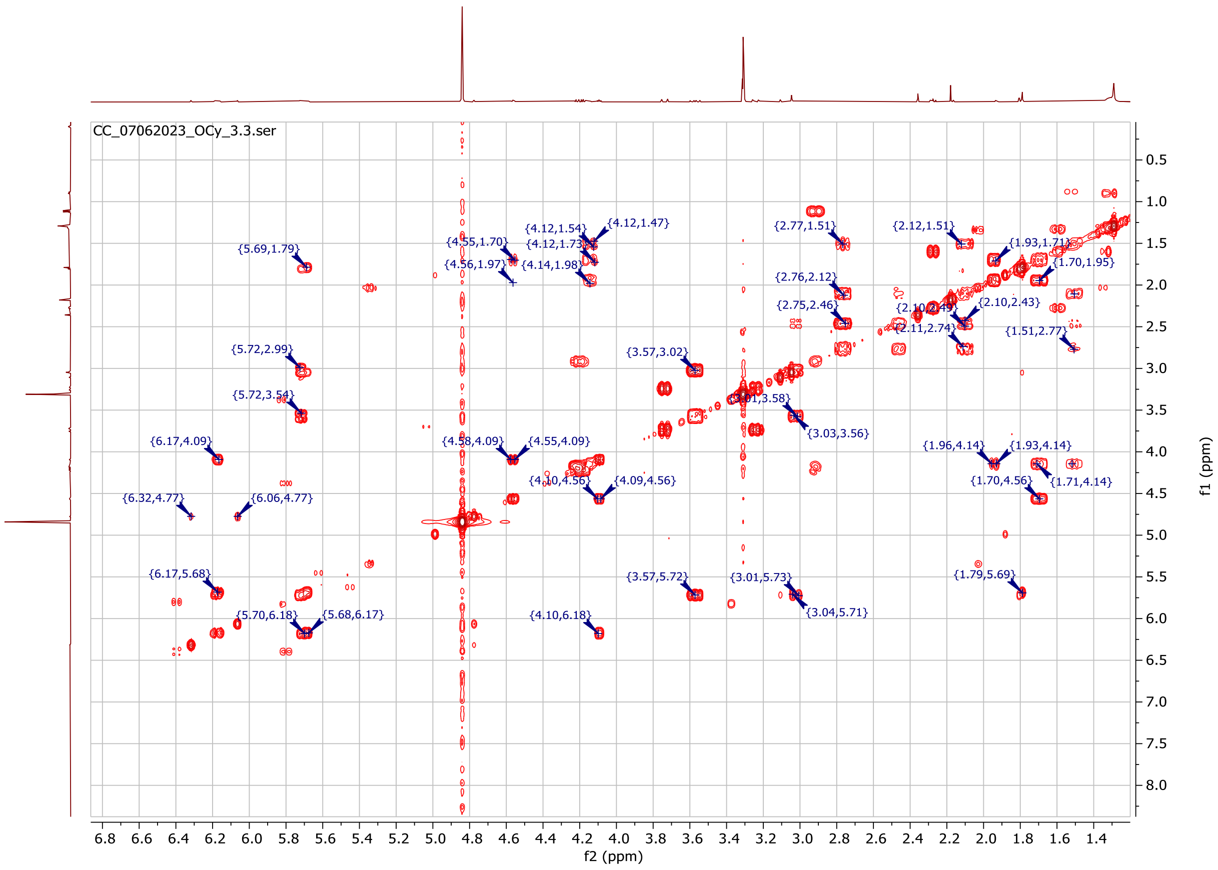

Figure S19 COSY spectrum of **2** in methanol-d_4 (^1H 500 MHz).

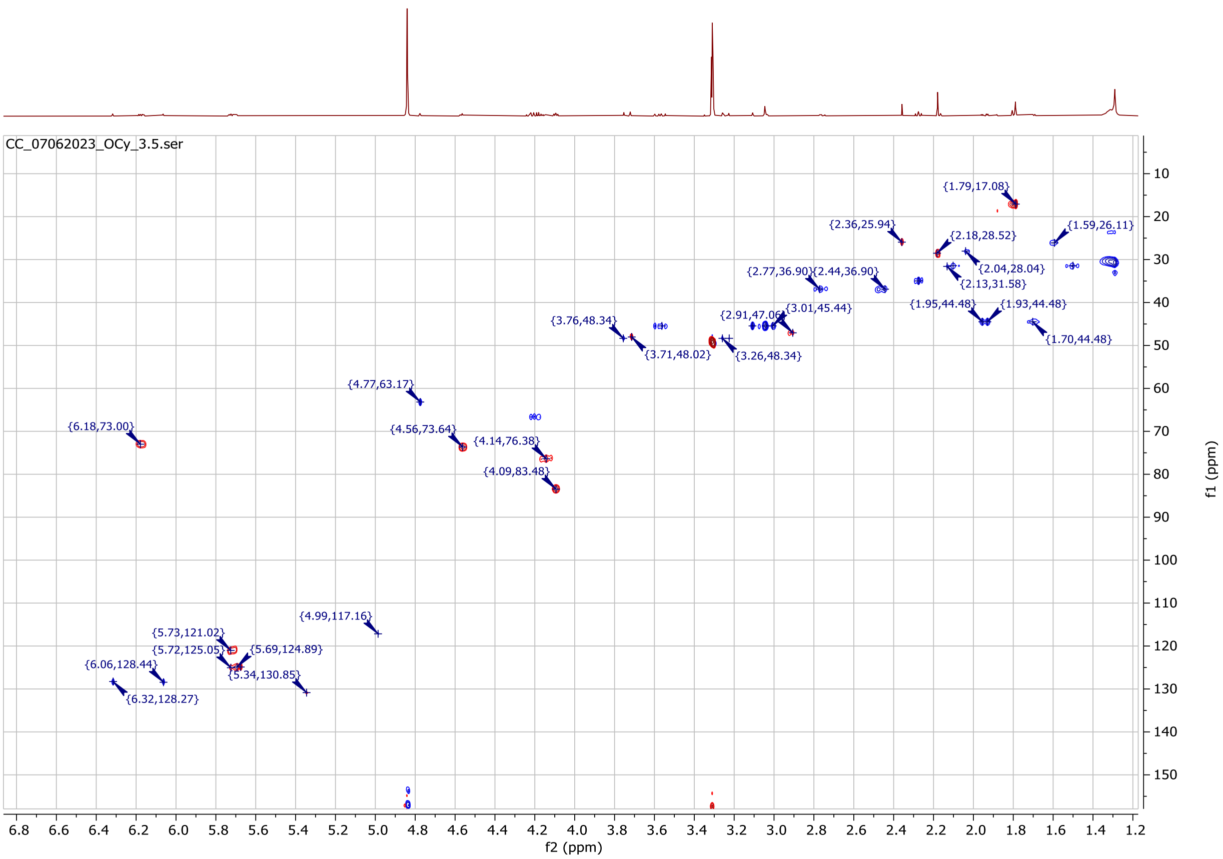

Figure S20 HSQC spectrum of **2** in methanol-d_4 (^1H 500 MHz, ^{13}C 125 MHz).

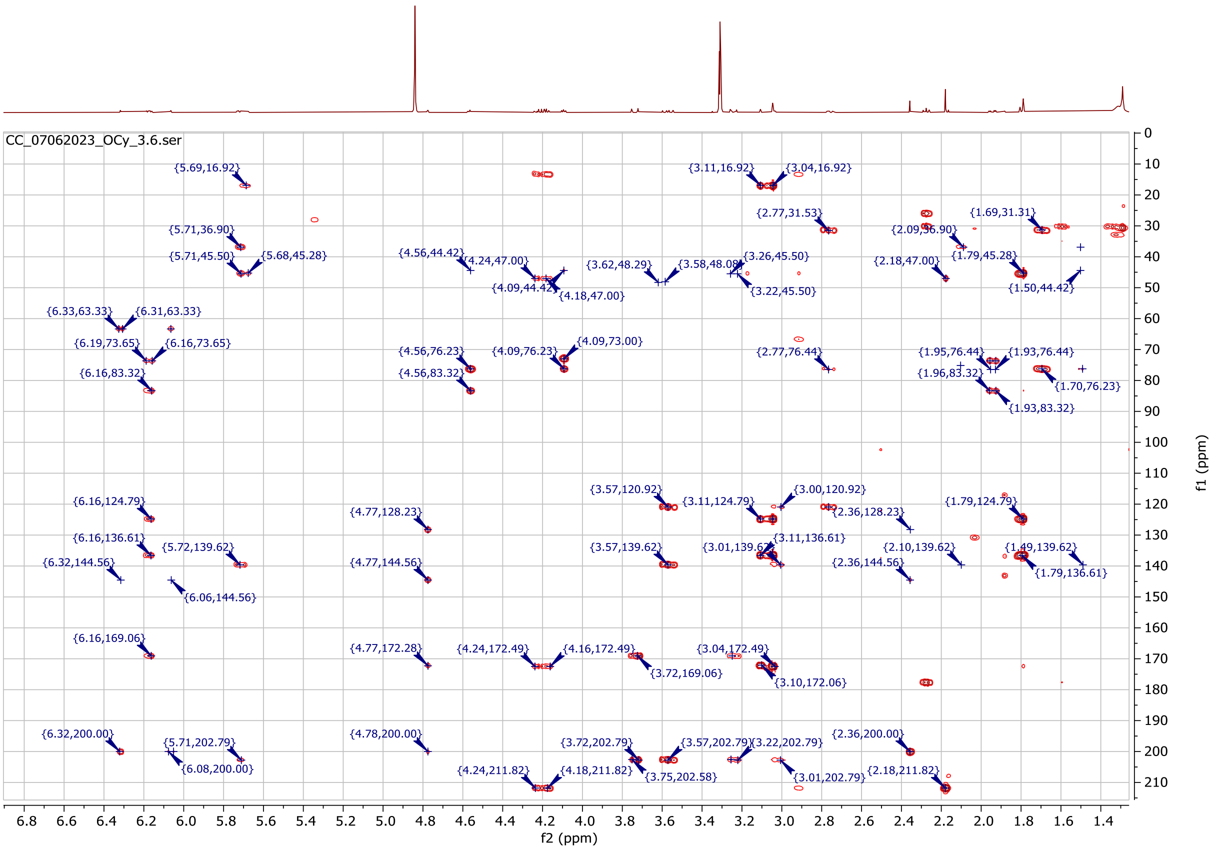

Figure S21 HMBC spectrum of **2** in methanol-d_4 (^1H 500 MHz, ^{13}C 125 MHz).

Compound **3** was assigned the molecular formula $C_{25}H_{31}ClO_9$ and 10 degrees of unsaturation based on the deduction from the HR-MS data (*m/z* 511.1712 $[M+H]^+$, Δ -1.69 mmu). The substructure I was elucidated from the HMBC correlations of H-3 to C-5/C-1, H-5 to C-3/C-6. COSY correlations of H_2-7 to H-8 in conjunction with the HMBC correlations of H-8 to C-6 connected the side chain to the pyrone ring. Typical pyrone ^{13}C and ^{1}H chemical shifts at δ 87.9 (δ 5.88) and δ 104.2 (δ 6.67) for position 3 and 5 respectively were observed in the HSQC spectrum. The second spin system for substructure II was elucidated from the COSY correlations of H-10 to H-9/H-11, H-12 to H-11/H-13/, H-14 to H-13/H-15 and H-15 to H-16. HMBC correlations of H_3-26 (δ 1.79) to C-17/C-18/C-19 and H_2-19 (δ 3.02) to C-17/C-18/C-20 was recorded. The oxolane ring was deduced from downfield shift of C-12 (δ 77.8) and C-15 (δ 81.5). These chemical shifts were in agreement with those reported for oocydin B (*17*). The substructure III was deduced from the HMBC correlations of H_2-21 to C-25 and H_3-24 to C-22/C-23. The three sub structures were connected based on the HMBC correlations of the H_2-21 to C-20 and H-8 to C-10. The chloride atom predicted from the HR-MS data HRM was attached to the only remaining open position to at C-9 (δ 136.3). The NMR data assignment in compound **3** from C-10 to C-20 were in agreement with those previously assigned for oocydin B[35] with the exception of the slightly difference in chemical shifts for oxolane ring. The upfield shift in the chemical shift at position 14 (δ 4.48, δ 73.3) compared to the oocydin B chemical shifts (δ 5.31, δ 76.5)[35], supported the absence of the macrolide ring in **3.**

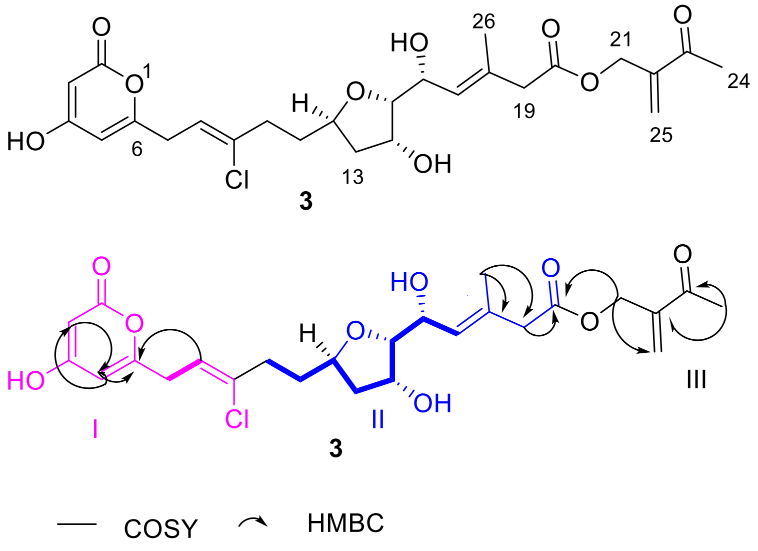

Figure S22 Structural assignment of **3**.

Table S14 NMR data for compound **3** in methanol-d_4 (^{1}H 500 MHz, ^{13}C 150 MHz).

No.	^{13}C/HSQC	^{1}H
2	160.7, C	
3	87.9, CH	5.88, (s)
3	172.6, C	
5	104.2, CH	6.67 (s)
6	149.5, C	
7	33.5, CH_2	3.39 (d), *J*= 6.9
8	119.7, CH	5.79 (t), *J*=7.2 Hz
9	136.3, C	
10	36.9, CH_2	2.52 (m)
11	34.5, CH_2	1.72 (m)
		1.78 (m)
12	77.8, CH	4.24 (m)
13	34.5, CH_2	1.70(m)
		2.06 (m)
14	73.3, CH	4.48 (m)
15	85.1, CH	3.68 (m)
16	67.7, CH	4.60 (m)
17	130.5, CH	5.43 (d), *J*=11.2 Hz
18	134.4, C	
19	45.6, CH_2	3.02 (S, br)
20	175.8, C	
21	63.2, CH_2	4.77 (s)
22	144.7, C	
23	201.1, C	
24	25.1, CH_3	1.37 (s)
25	128.1, CH_2	6.03 (s)
		6.36 (s)
26	16.9, CH_3	1.79 (s)

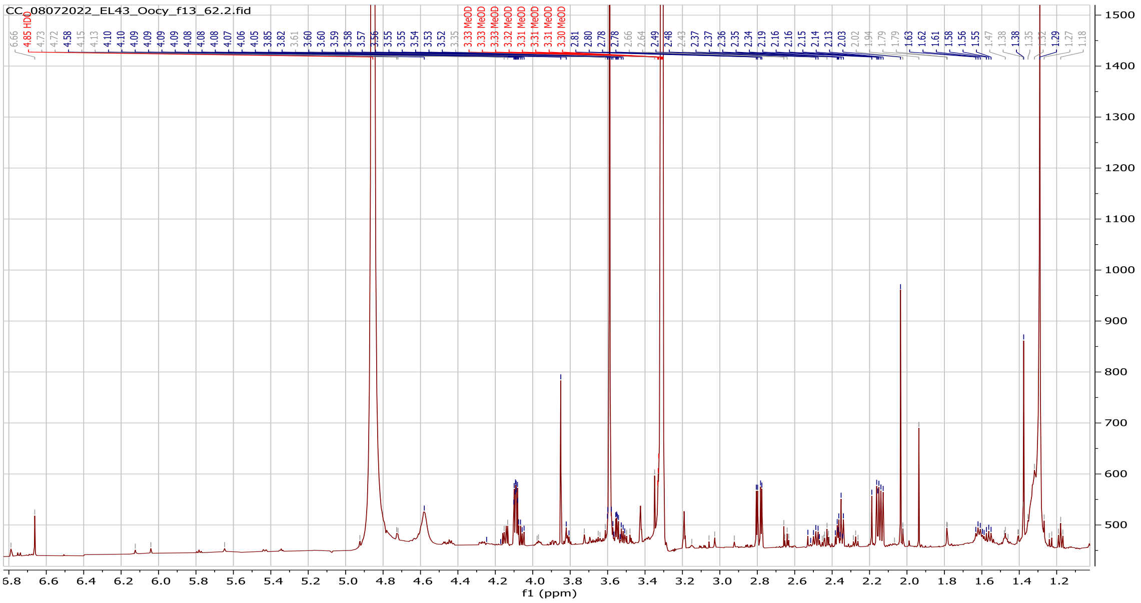

Figure S23 ^{1}H NMR spectrum of **3** in methanol-d_4 (^{1}H 600 MHz).

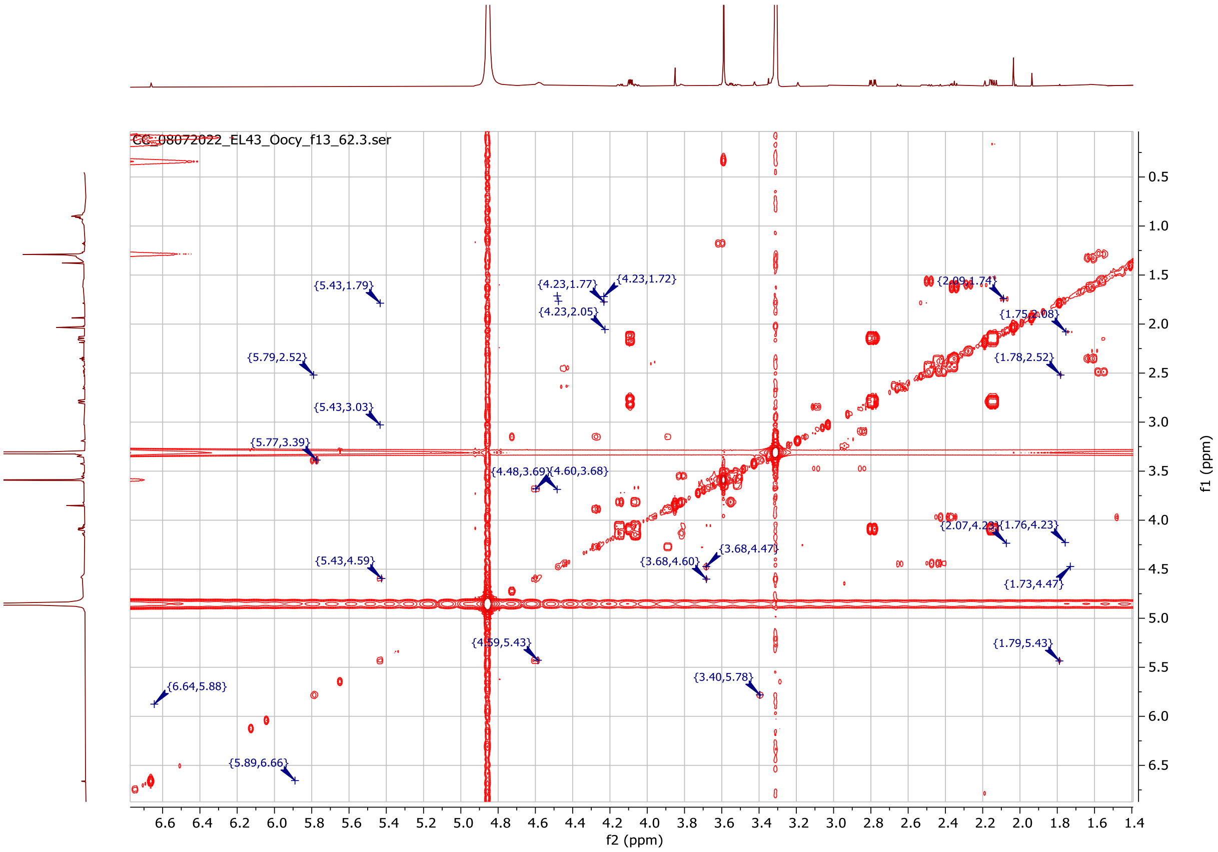

Figure S24 COSY spectrum of **3** in methanol-d_4 (^{1}H 600 MHz).

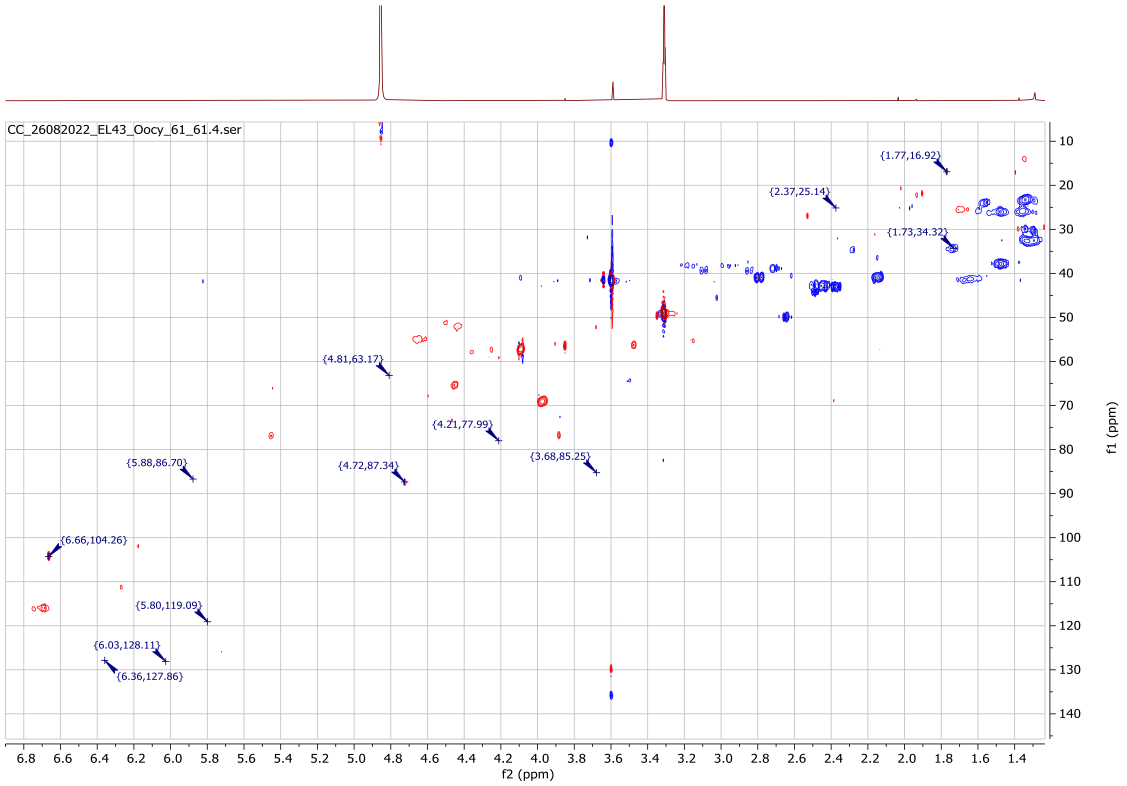

Figure S25 HSQC spectrum of **3** in methanol-d_4 (^{1}H 600 MHz, ^{13}C 150 MHz).

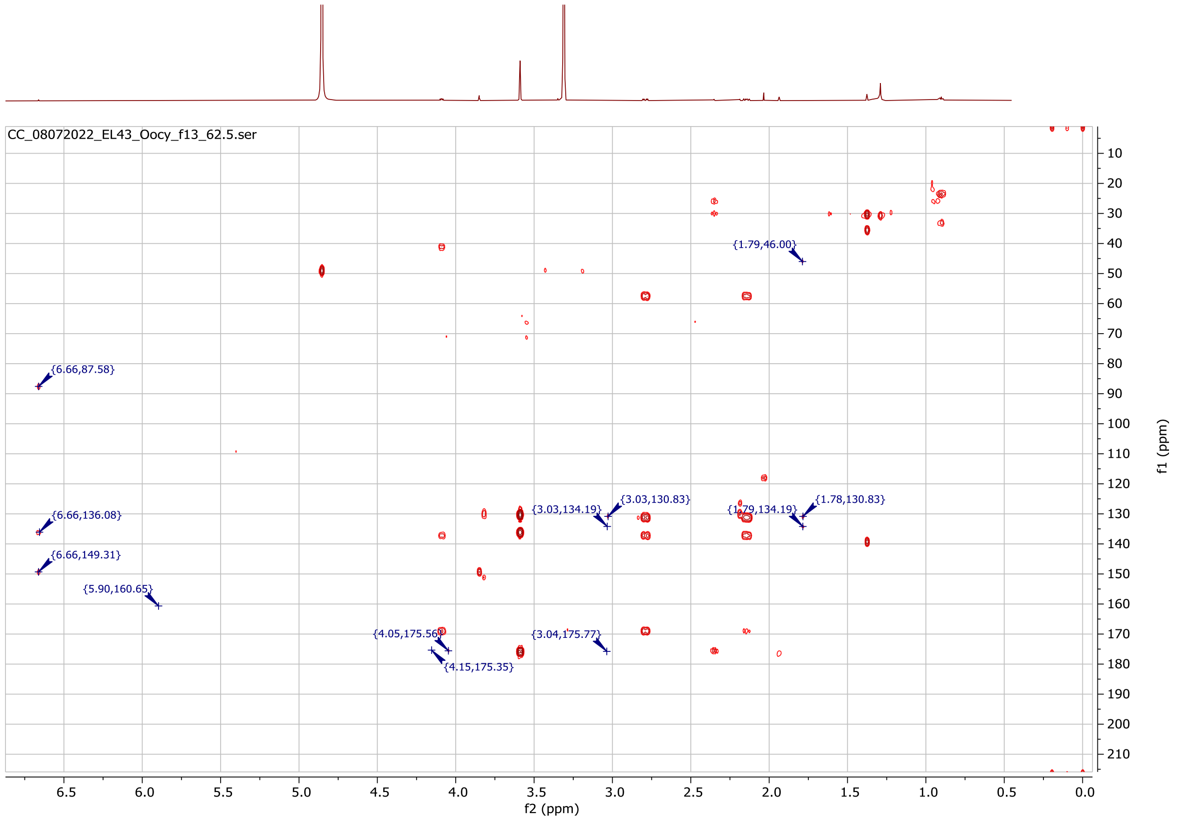

Figure S26 HMBC spectrum of **3** in methanol-d_4 (^{1}H 600 MHz, ^{13}C 150 MHz).

Extracted ion chromatograms for I-V and oocydin congeners of cultures of *Serratia plymuthica* mutants

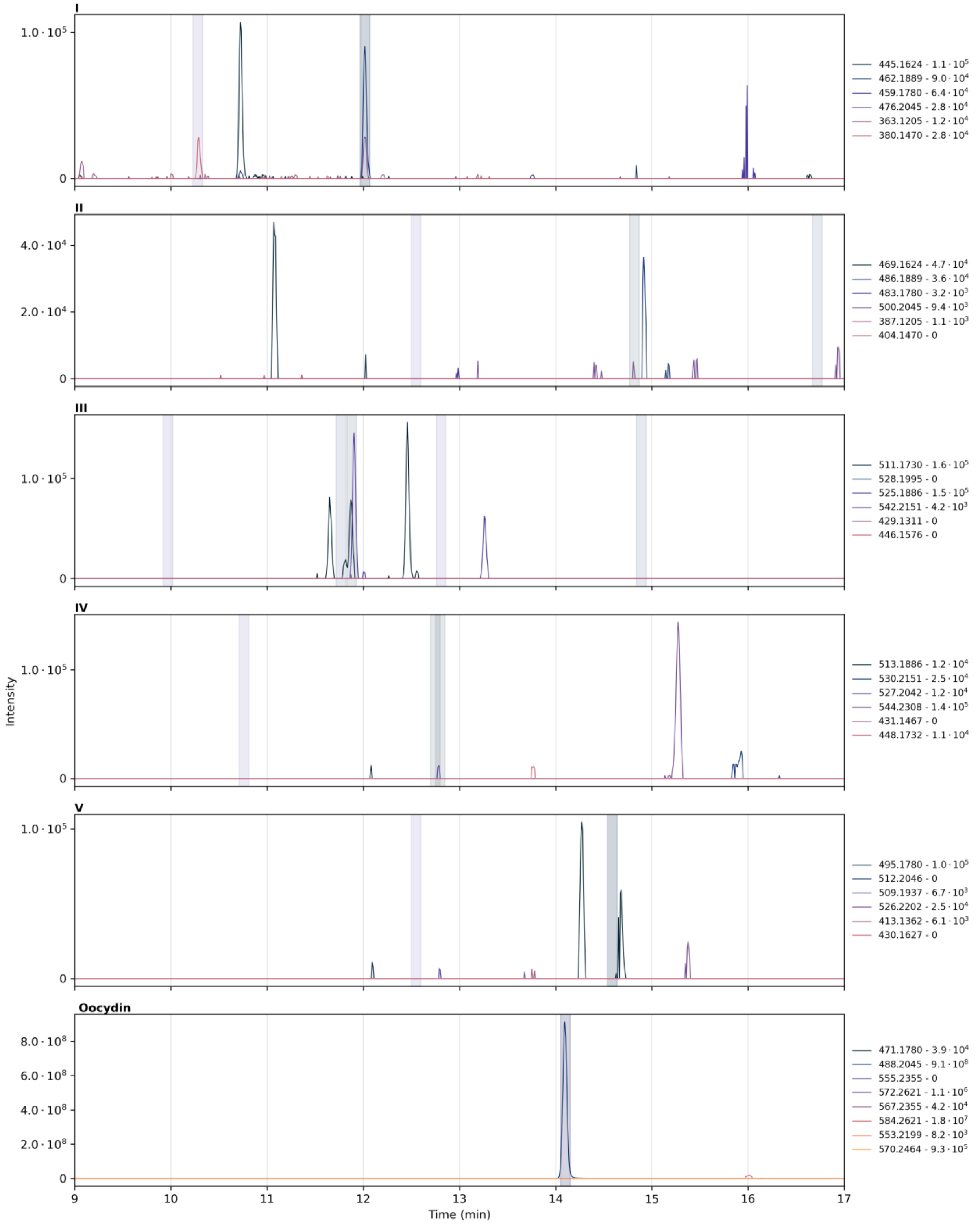

Figure S27 Extracted ion chromatograms of an expression culture extract of *S. plymuthica* 4Rx13. The *m/z* values of proton and ammonium adducts are indicated in the legend of each subplot, with the maximum intensity detected for each *m/z* value. The shaded areas indicate the consensus retention time for the compounds with corresponding color coding.

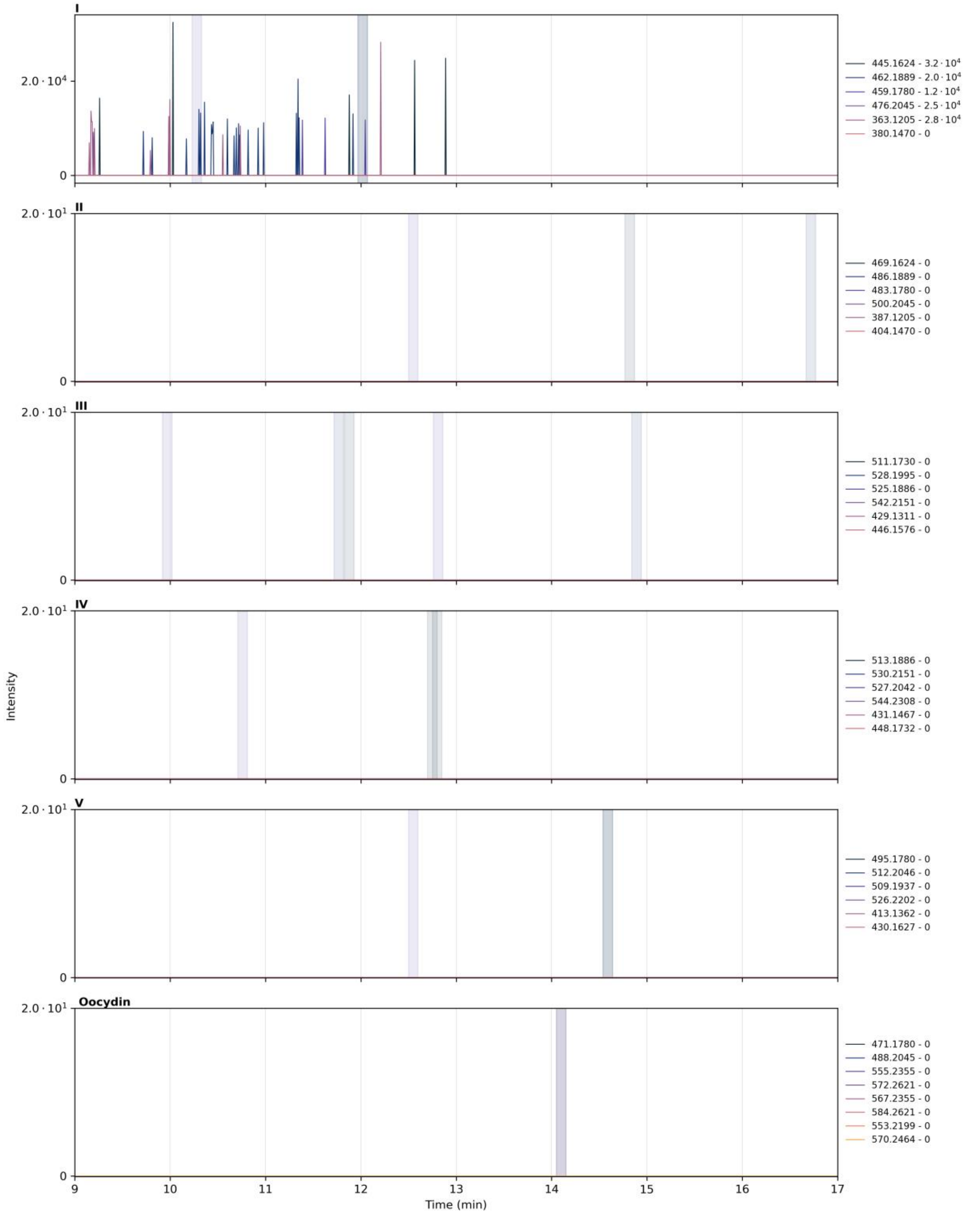

Figure S28 Extracted ion chromatograms of an expression culture extract of *S. plymuthica* 4Rx13 Δ*oocQR*. The *m/z* values of proton and ammonium adducts are indicated in the legend of each subplot, with the maximum intensity detected for each *m/z* value. The shaded areas indicate the consensus retention time for the compounds with corresponding color coding.

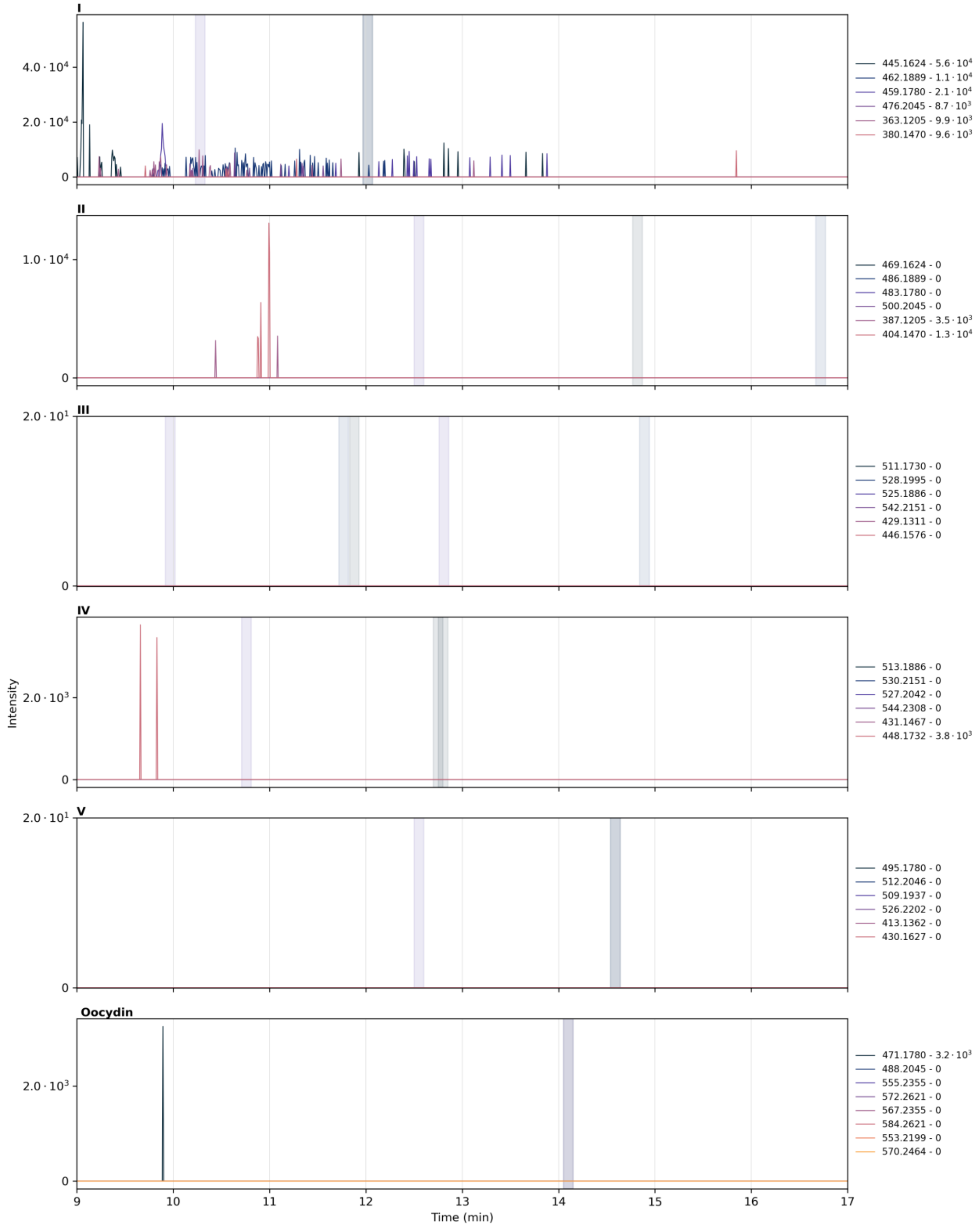

Figure S29 Extracted ion chromatograms of an expression culture extract of *S. plymuthica* 4Rx13 Δ*oocQR* + *pBAD*. The *m/z* values of proton and ammonium adducts are indicated in the legend of each subplot, with the maximum intensity detected for each *m/z* value. The shaded areas indicate the consensus retention time for the compounds with corresponding color coding.

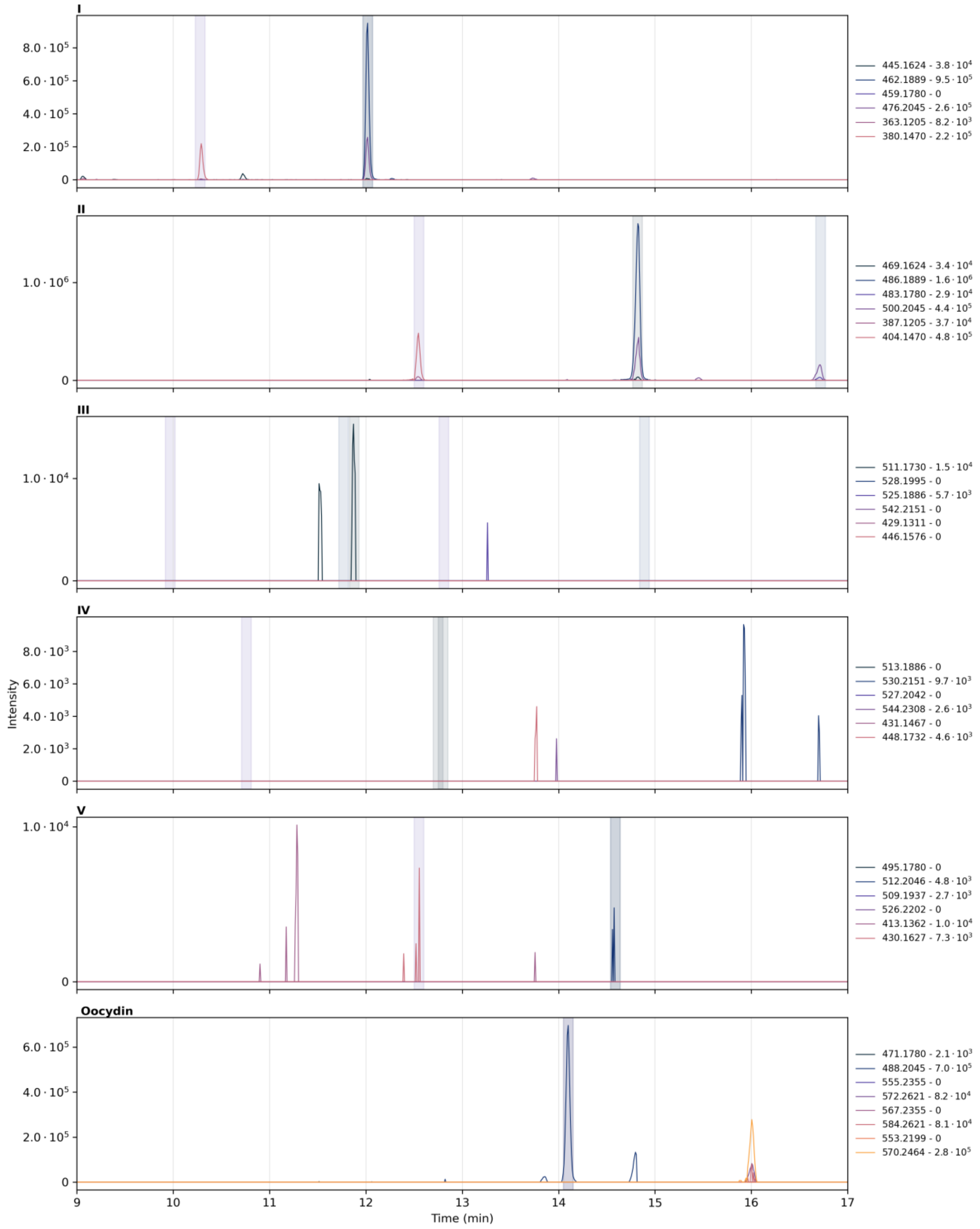

Figure S30 Extracted ion chromatograms of an expression culture extract of *S. plymuthica* 4Rx13 Δ*oocQR* + *pBAD-oocQR*. The *m/z* values of proton and ammonium adducts are indicated in the legend of each subplot, with the maximum intensity detected for each *m/z* value. The shaded areas indicate the consensus retention time for the compounds with corresponding color coding.

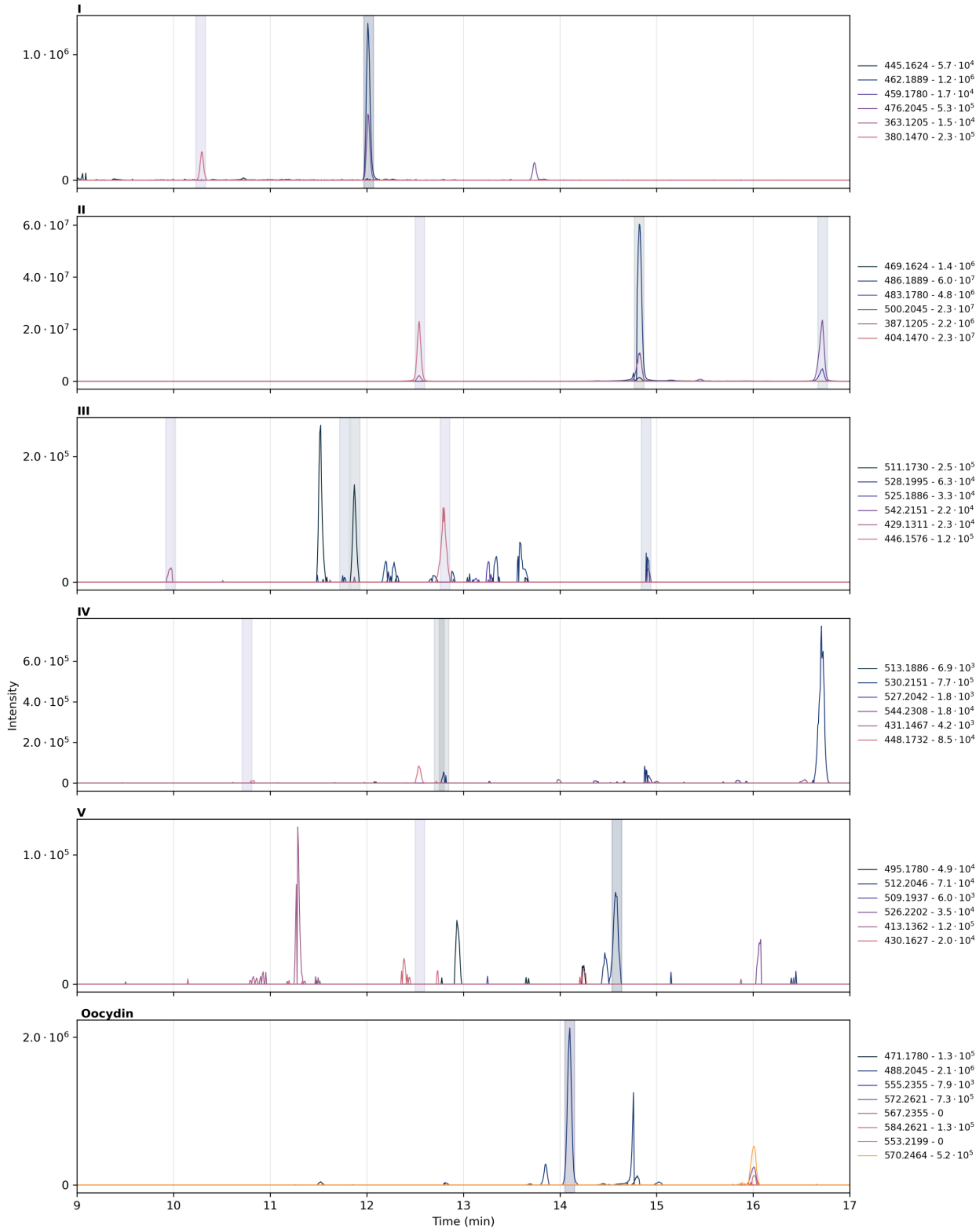

Figure S31 Extracted ion chromatograms of an expression culture extract of *S. plymuthica* 4Rx13 Δ*oocQR* + *pBAD-oocQRS*$_C$. The *m/z* values of proton and ammonium adducts are indicated in the legend of each subplot, with the maximum intensity detected for each *m/z* value. The shaded areas indicate the consensus retention time for the compounds with corresponding color coding.

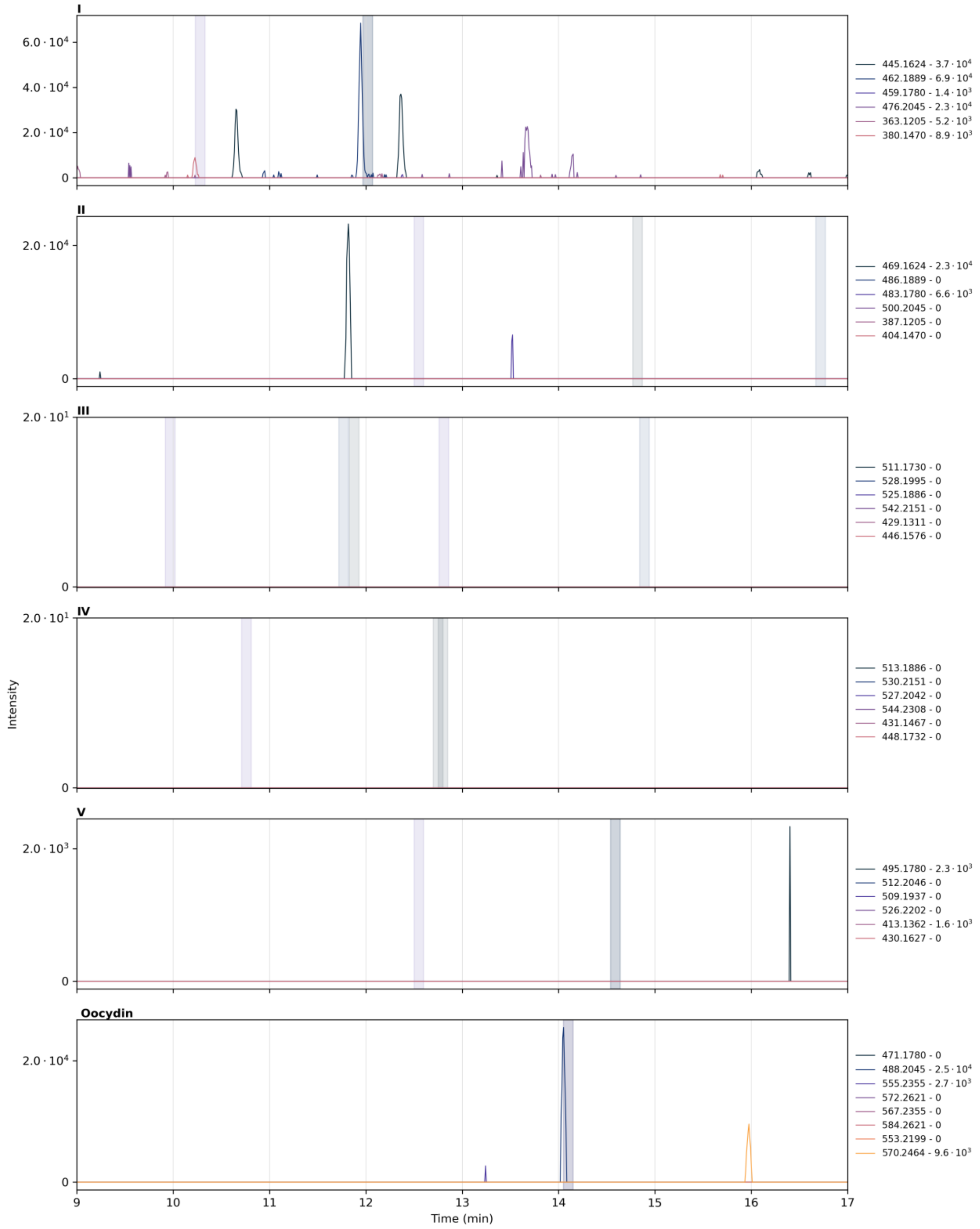

Figure S32 Extracted ion chromatograms of an expression culture extract of *S. plymuthica* 4Rx13 Δ*oocQR* + *pBAD-oocQR*$_{\text{KS0-DH-ACP}}$-*lbm*$_{\text{KS12}}$-*OocS*$_{\text{C}}$. The *m/z* values of proton and ammonium adducts are indicated in the legend of each subplot, with the maximum intensity detected for each *m/z* value. The shaded areas indicate the consensus retention time for the compounds with corresponding color coding.

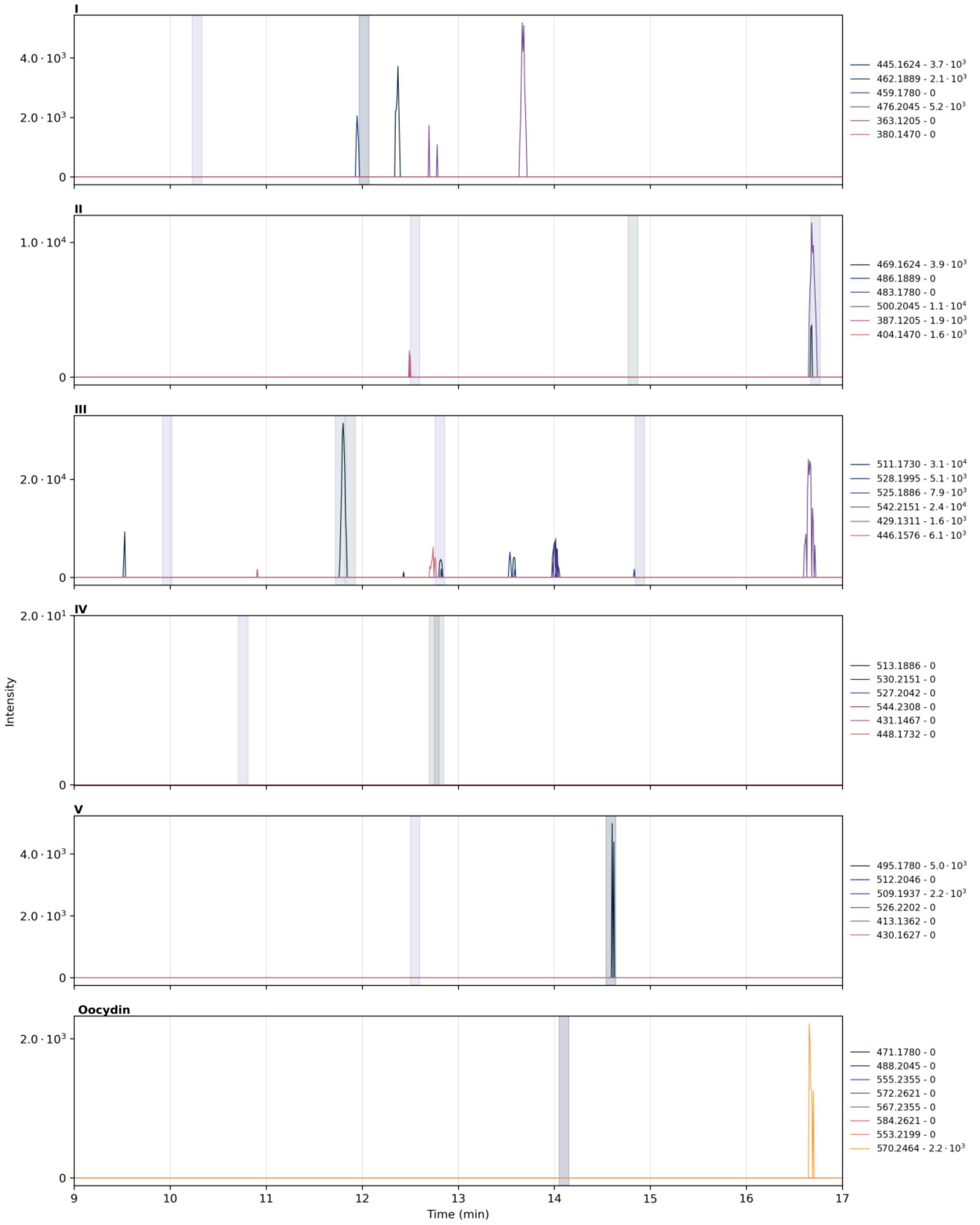

Figure S33 Extracted ion chromatograms of an expression culture extract of *S. plymuthica* 4Rx13 Δ*oocQR* + *pBAD*-*oocQR*$_{\text{KS0-DH-ACP}}$-*lbm*$_{\text{KS11-ACP-KS12}}$-*oocS*$_{\text{C}}$. The *m/z* values of proton and ammonium adducts are indicated in the legend of each subplot, with the maximum intensity detected for each *m/z* value. The shaded areas indicate the consensus retention time for the compounds with corresponding color coding.

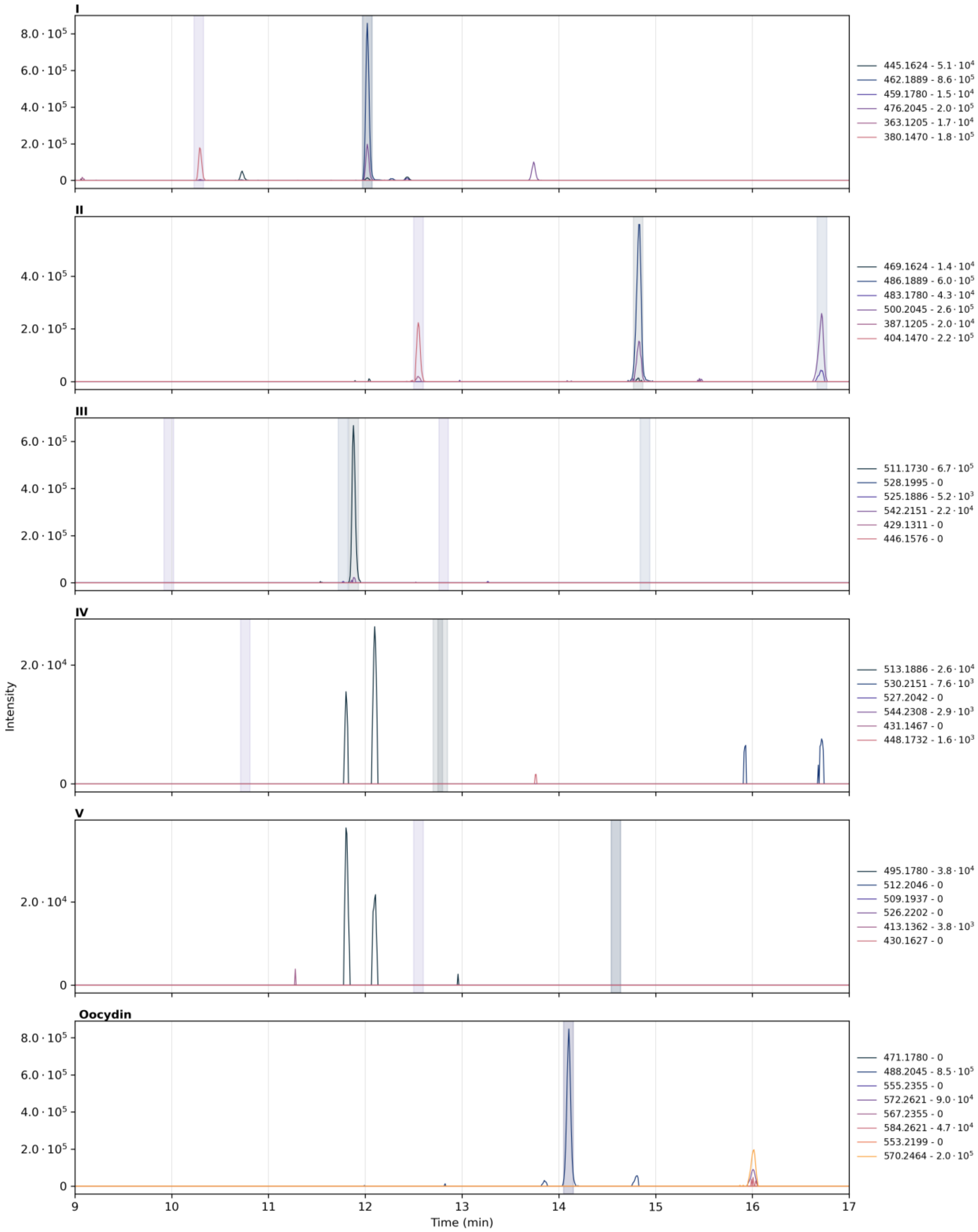

Figure S34 Extracted ion chromatograms of an expression culture extract of *S. plymuthica* 4Rx13 Δ*oocQR* + *pBAD-oocQR-Psy*$_{KS11}$ – Fusion site: LPTYPFx_5W. The *m/z* values of proton and ammonium adducts are indicated in the legend of each subplot, with the maximum intensity detected for each *m/z* value. The shaded areas indicate the consensus retention time for the compounds with corresponding color coding.

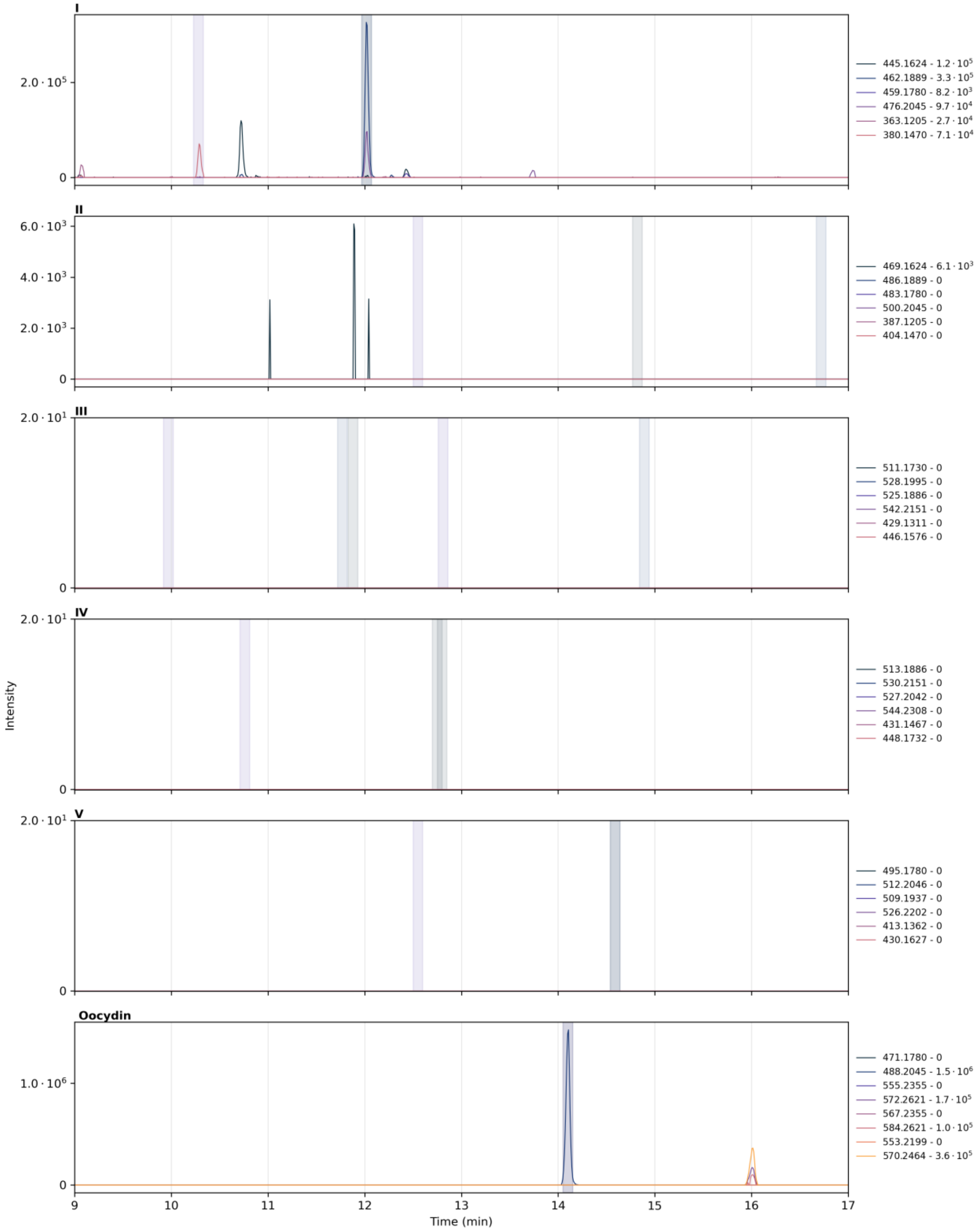

Figure S35 Extracted ion chromatograms of an expression culture extract of *S. plymuthica* 4Rx13 Δ*oocQR* + *pBAD-oocQR-Psy*$_{KS11}$ – Fusion site: NAHVILEE. The *m/z* values of proton and ammonium adducts are indicated in the legend of each subplot, with the maximum intensity detected for each *m/z* value. The shaded areas indicate the consensus retention time for the compounds with corresponding color coding.

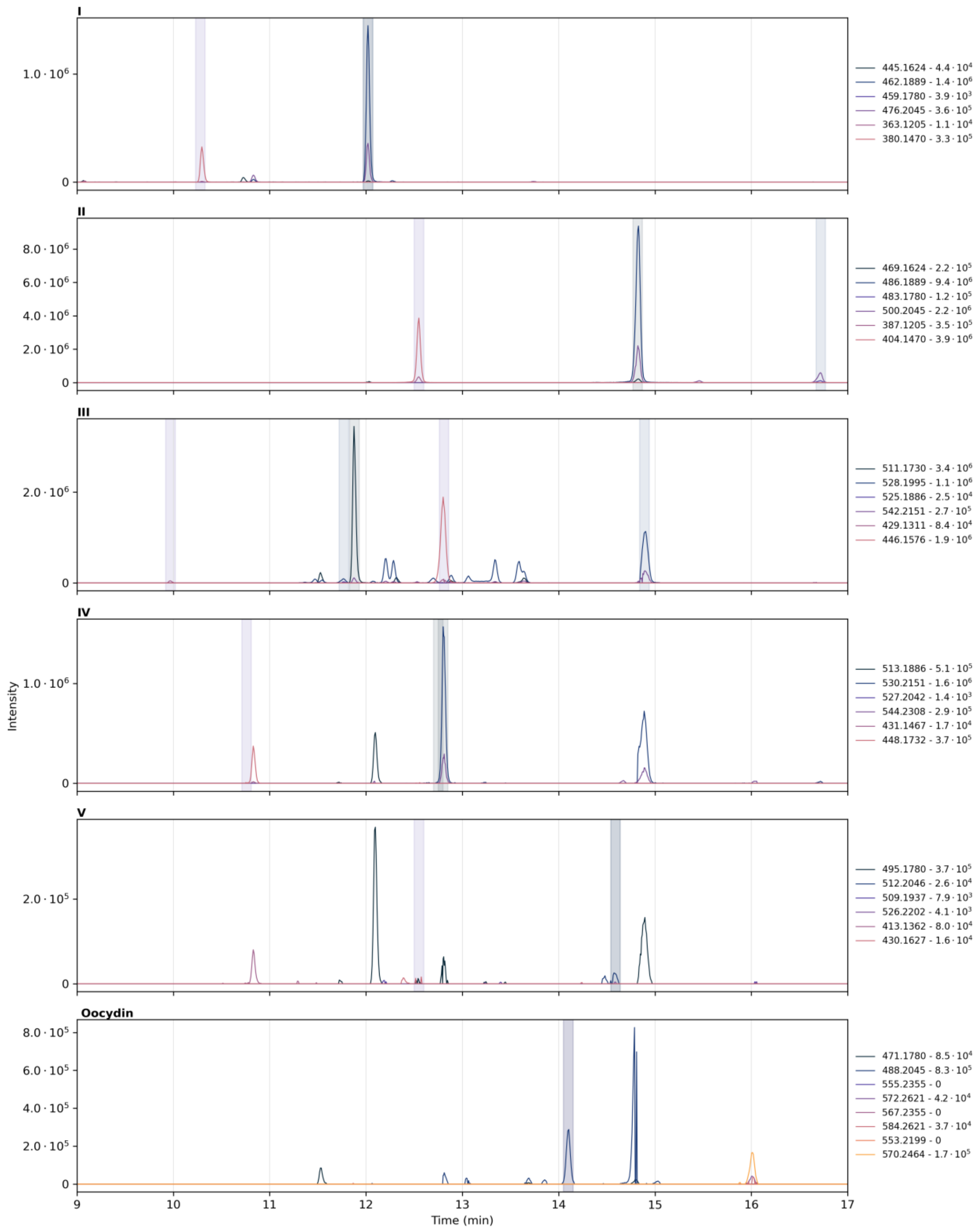

Figure S36 Extracted ion chromatograms of an expression culture extract of *S. plymuthica* 4Rx13 Δ*oocQR* + *pBAD-oocQR-Lbm12-oocS*$_C$ – Upstream fusion site: LPTYPFx$_5$W, downstream fusion site: LPTYPFx$_5$W. The *m/z* values of proton and ammonium adducts are indicated in the legend of each subplot, with the maximum intensity detected for each *m/z* value. The shaded areas indicate the consensus retention time for the compounds with corresponding color coding.

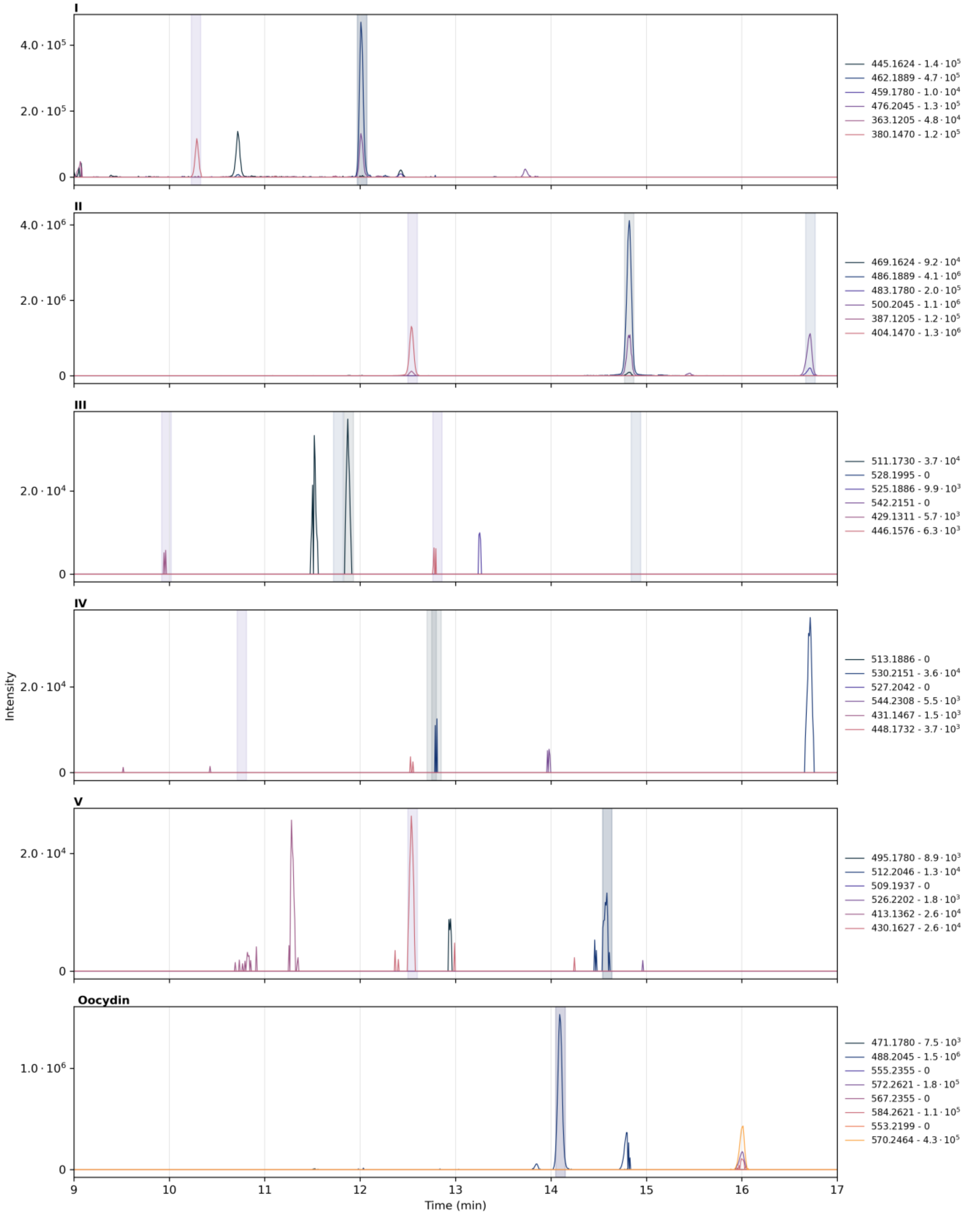

Figure S37 Extracted ion chromatograms of an expression culture extract of *S. plymuthica* 4Rx13 Δ*oocQR* + *pBAD-oocQR-Lbm12-oocS*$_C$ – Upstream fusion site: NAHVILEE, downstream fusion site: LPTYPFx_5W. The *m/z* values of proton and ammonium adducts are indicated in the legend of each subplot, with the maximum intensity detected for each *m/z* value. The shaded areas indicate the consensus retention time for the compounds with corresponding color coding.

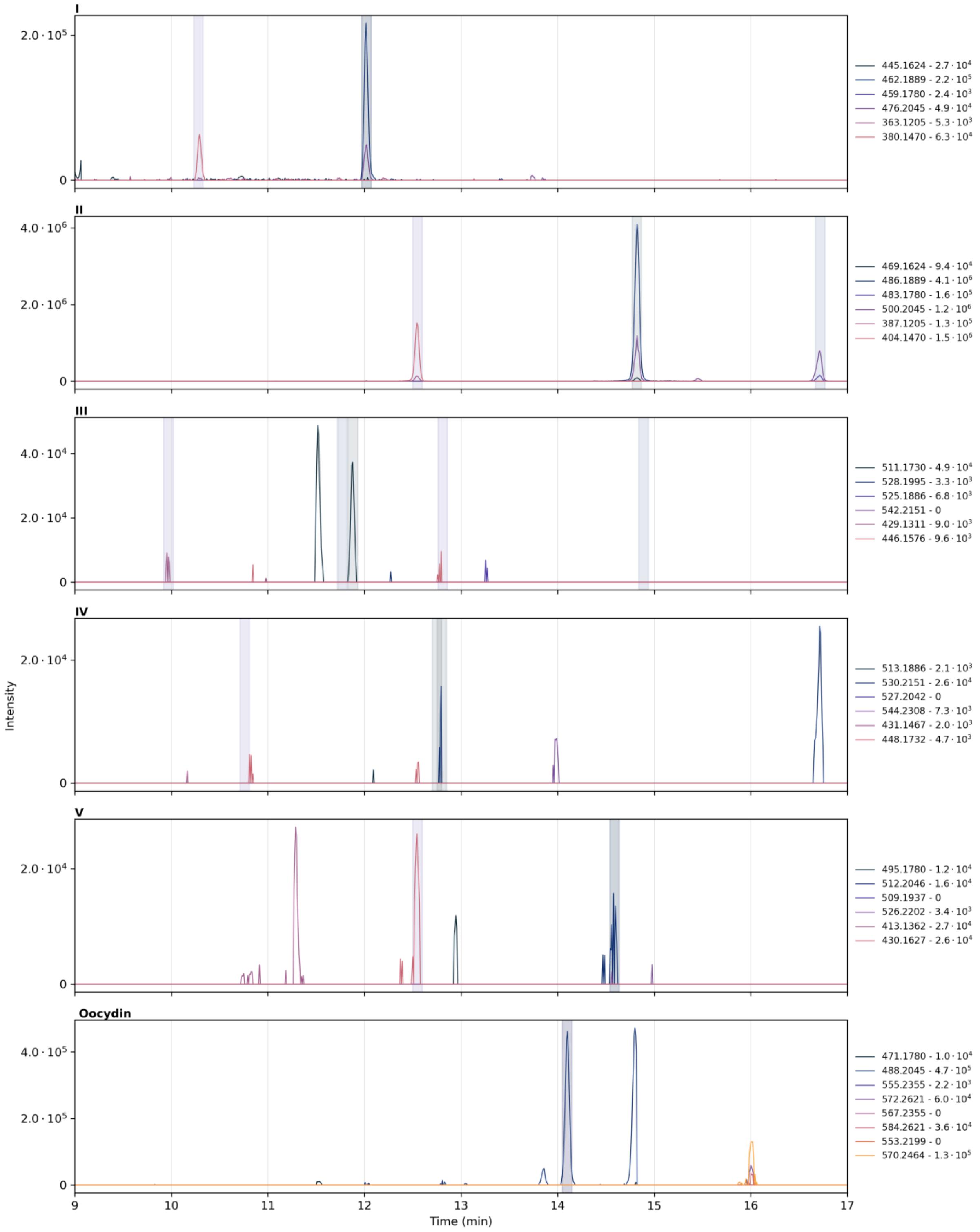

Figure S38 Extracted ion chromatograms of an expression culture extract of *S. plymuthica* 4Rx13 Δ*oocQR* + *pBAD-oocQR-Lbm12-oocS*$_C$ – Upstream fusion site: LPTYPFx$_5$W, downstream fusion site: NAHVILEE. The *m/z* values of proton and ammonium adducts are indicated in the legend of each subplot, with the maximum intensity detected for each *m/z* value. The shaded areas indicate the consensus retention time for the compounds with corresponding color coding.

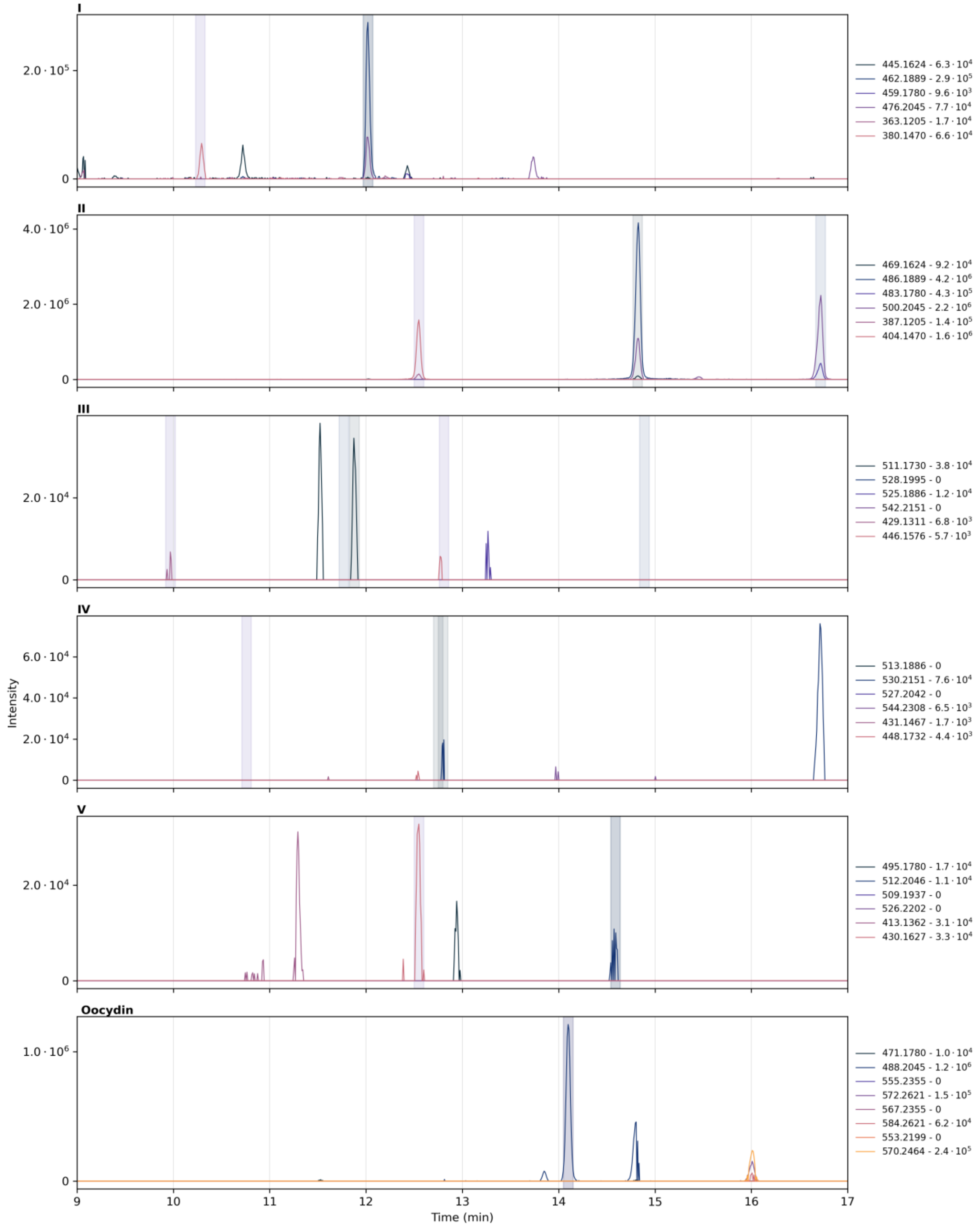

Figure S39 Extracted ion chromatograms of an expression culture extract of *S. plymuthica* 4Rx13 Δ*oocQR* + *pBAD-oocQR-Lbm12-oocS*$_C$ – Upstream fusion site: NAHVILEE, downstream fusion site: NAHVILEE. The *m/z* values of proton and ammonium adducts are indicated in the legend of each subplot, with the maximum intensity detected for each *m/z* value. The shaded areas indicate the consensus retention time for the compounds with corresponding color coding.

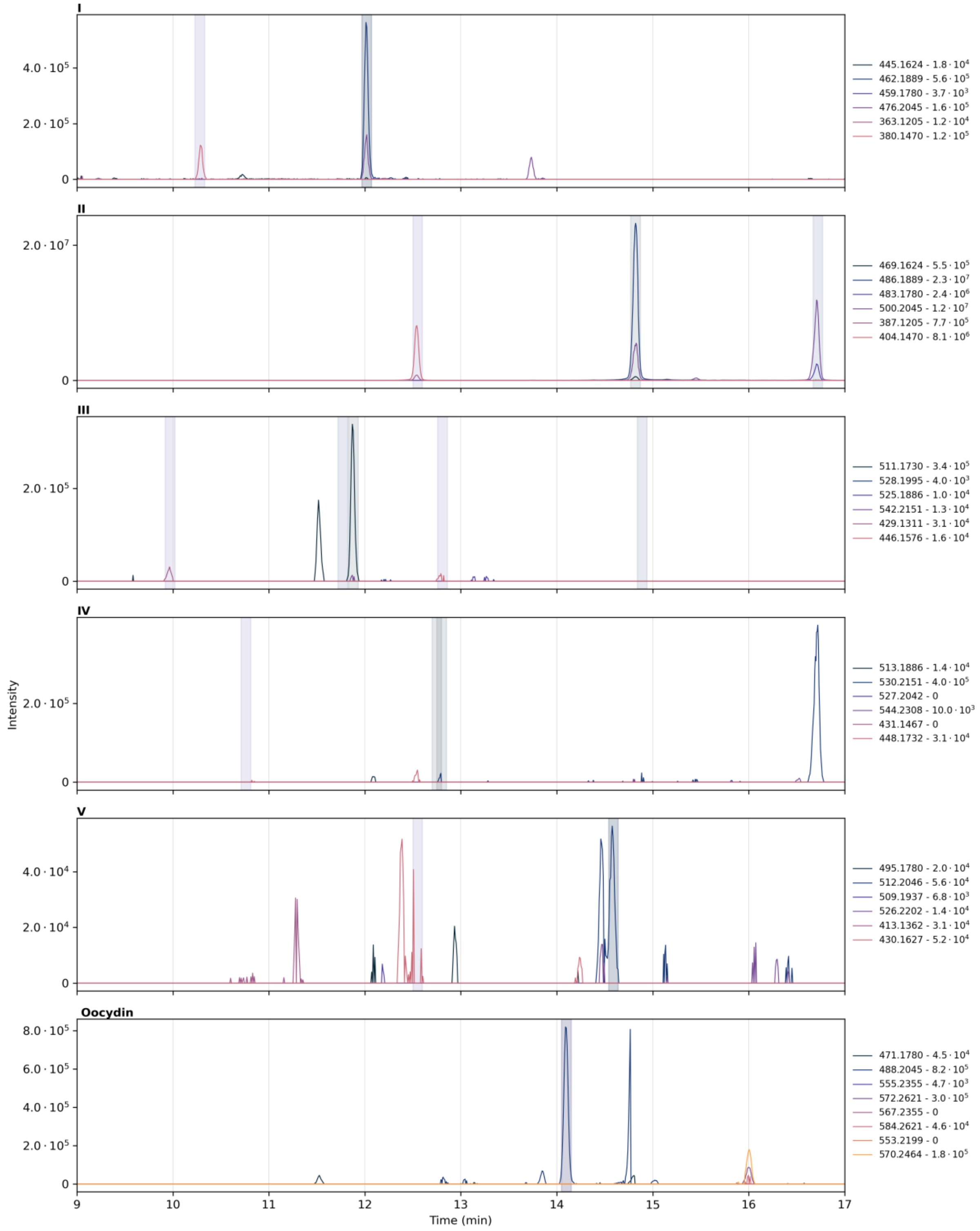

Figure S40 Extracted ion chromatograms of an expression culture extract of *S. plymuthica* 4Rx13 Δ*oocQR* + *pBAD-oocQR-Lbm11-oocS*$_C$. The *m/z* values of proton and ammonium adducts are indicated in the legend of each subplot, with the maximum intensity detected for each *m/z* value. The shaded areas indicate the consensus retention time for the compounds with corresponding color coding.

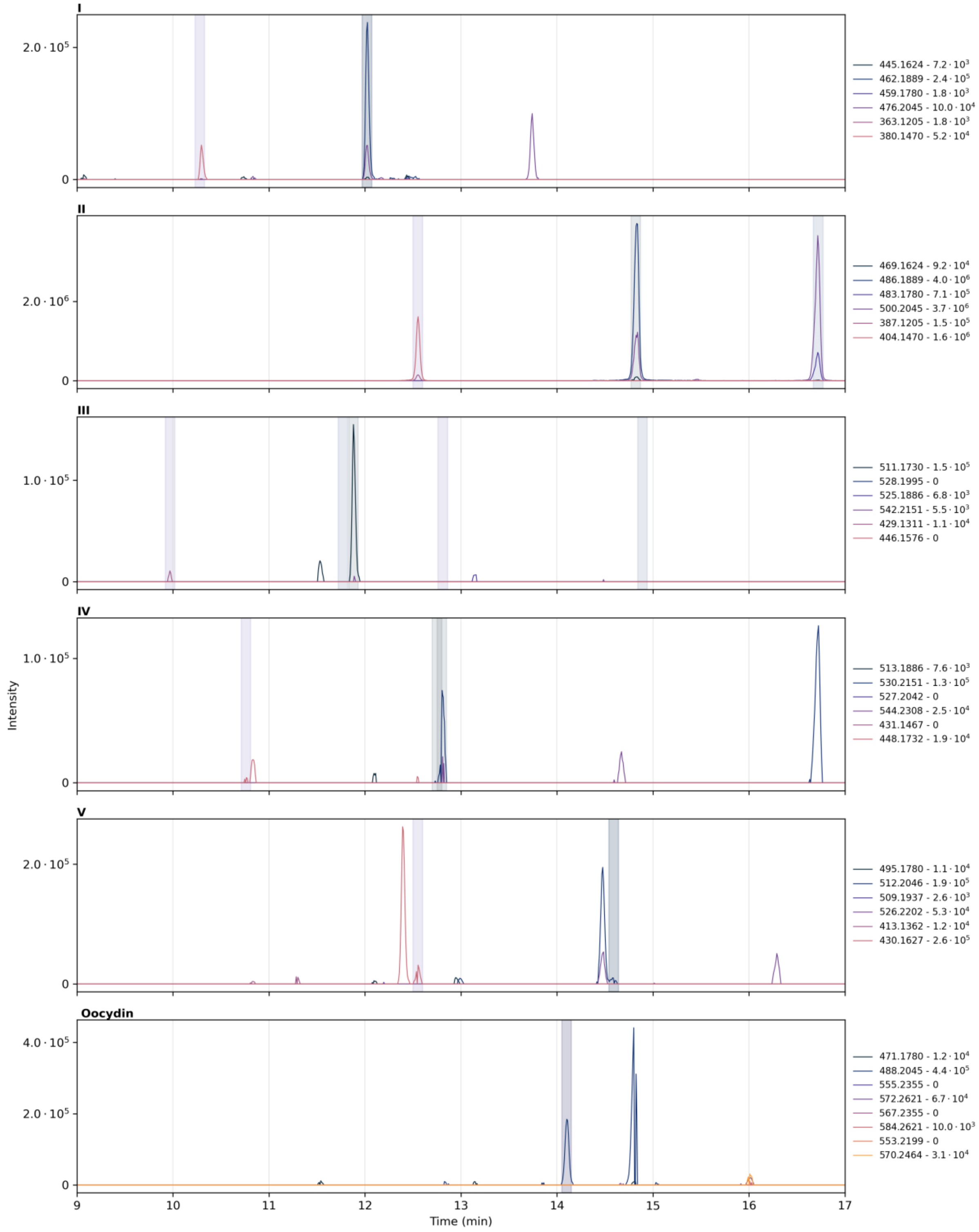

Figure S41 Extracted ion chromatograms of an expression culture extract of *S. plymuthica* 4Rx13 Δ*oocQR* + *pBAD-oocQR-Pks5-oocS*$_C$. The *m/z* values of proton and ammonium adducts are indicated in the legend of each subplot, with the maximum intensity detected for each *m/z* value. The shaded areas indicate the consensus retention time for the compounds with corresponding color coding.

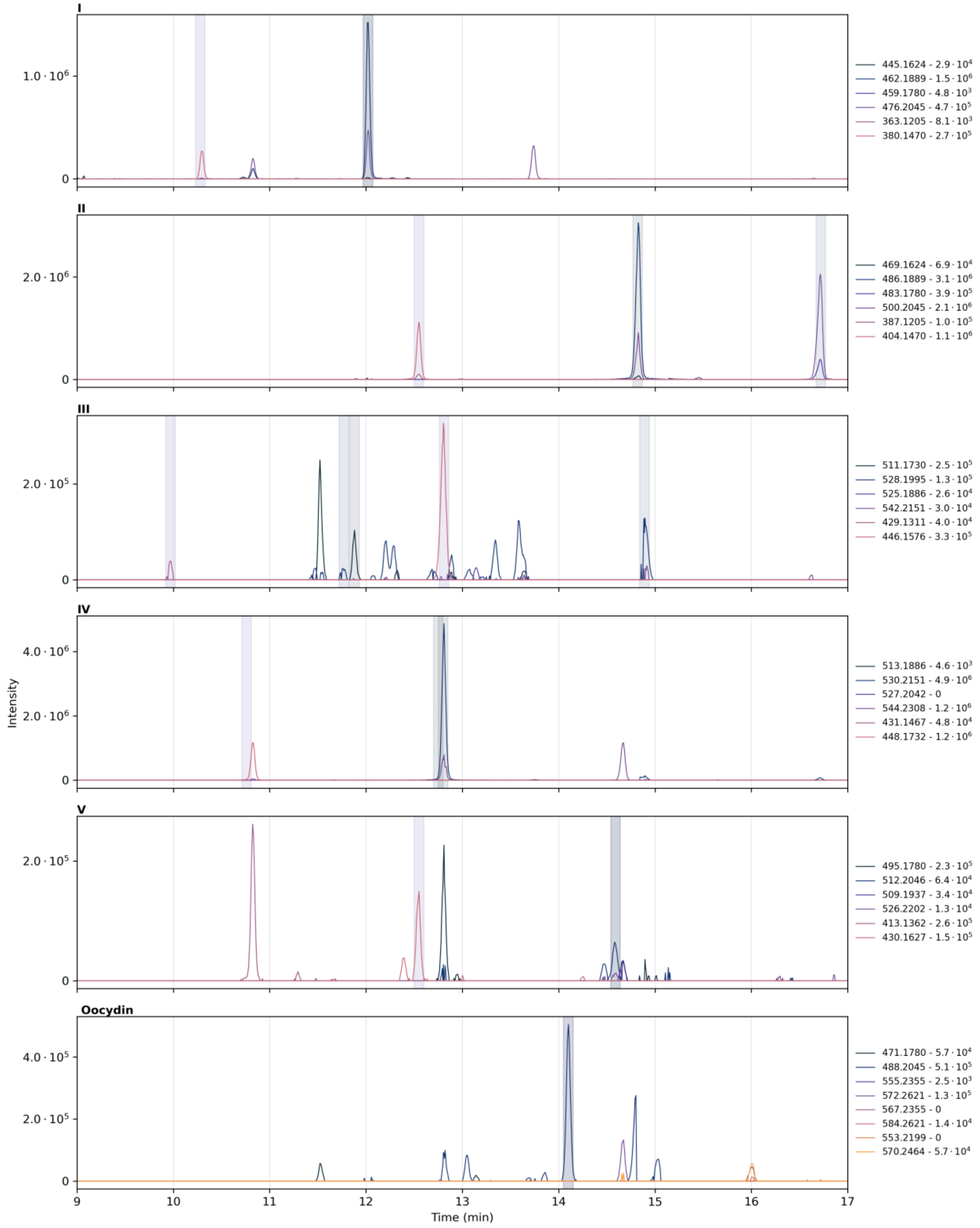

Figure S42 Extracted ion chromatograms of an expression culture extract of *S. plymuthica* 4Rx13 Δ*oocQR* + *pBAD-oocQR-Tar10-oocS*$_C$. The *m/z* values of proton and ammonium adducts are indicated in the legend of each subplot, with the maximum intensity detected for each *m/z* value. The shaded areas indicate the consensus retention time for the compounds with corresponding color coding.

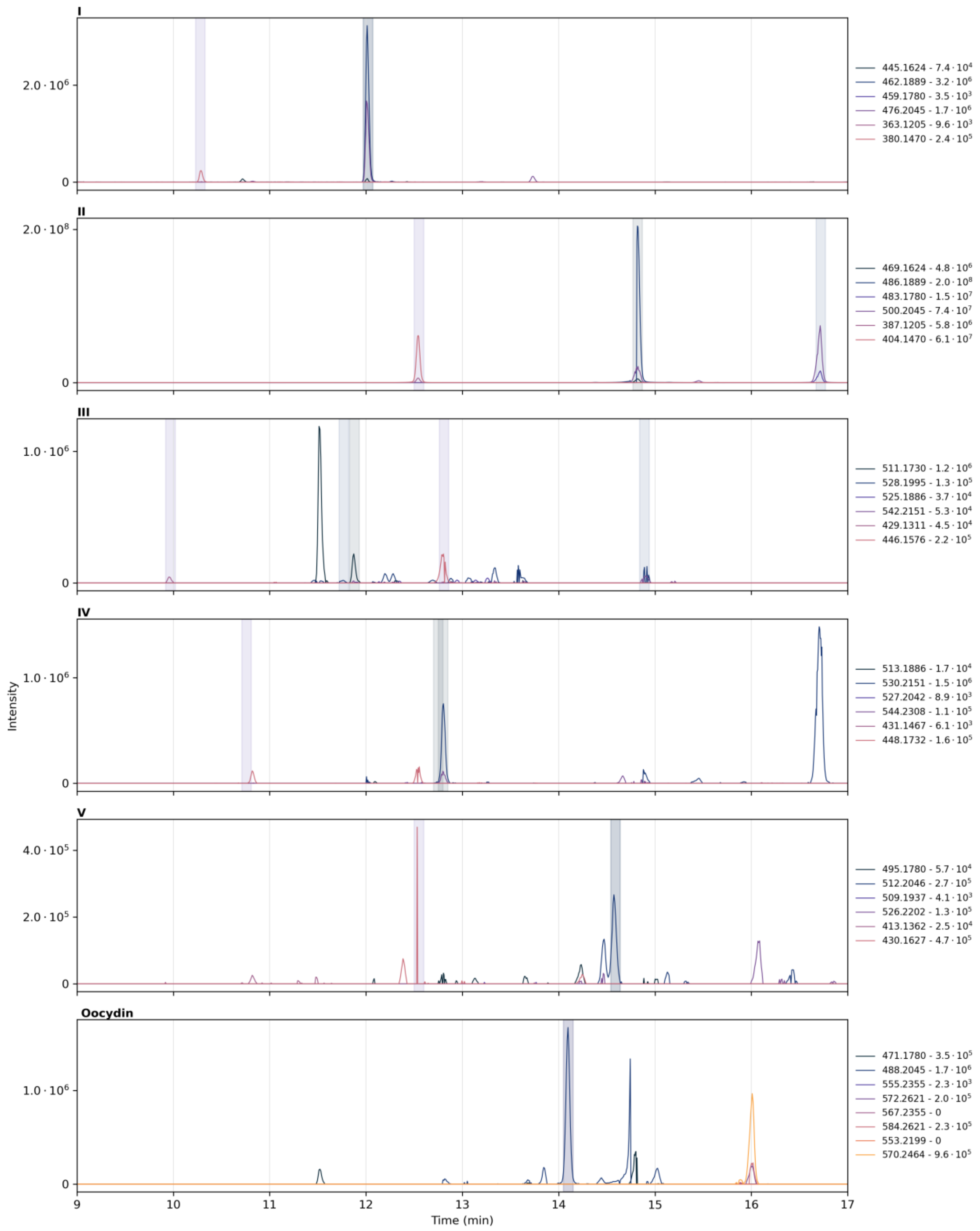

Figure S43 Extracted ion chromatograms of an expression culture extract of *S. plymuthica* 4Rx13 Δ*oocQR* + *pBAD-oocQR-Tar13-oocS*$_C$. The *m/z* values of proton and ammonium adducts are indicated in the legend of each subplot, with the maximum intensity detected for each *m/z* value. The shaded areas indicate the consensus retention time for the compounds with corresponding color coding.

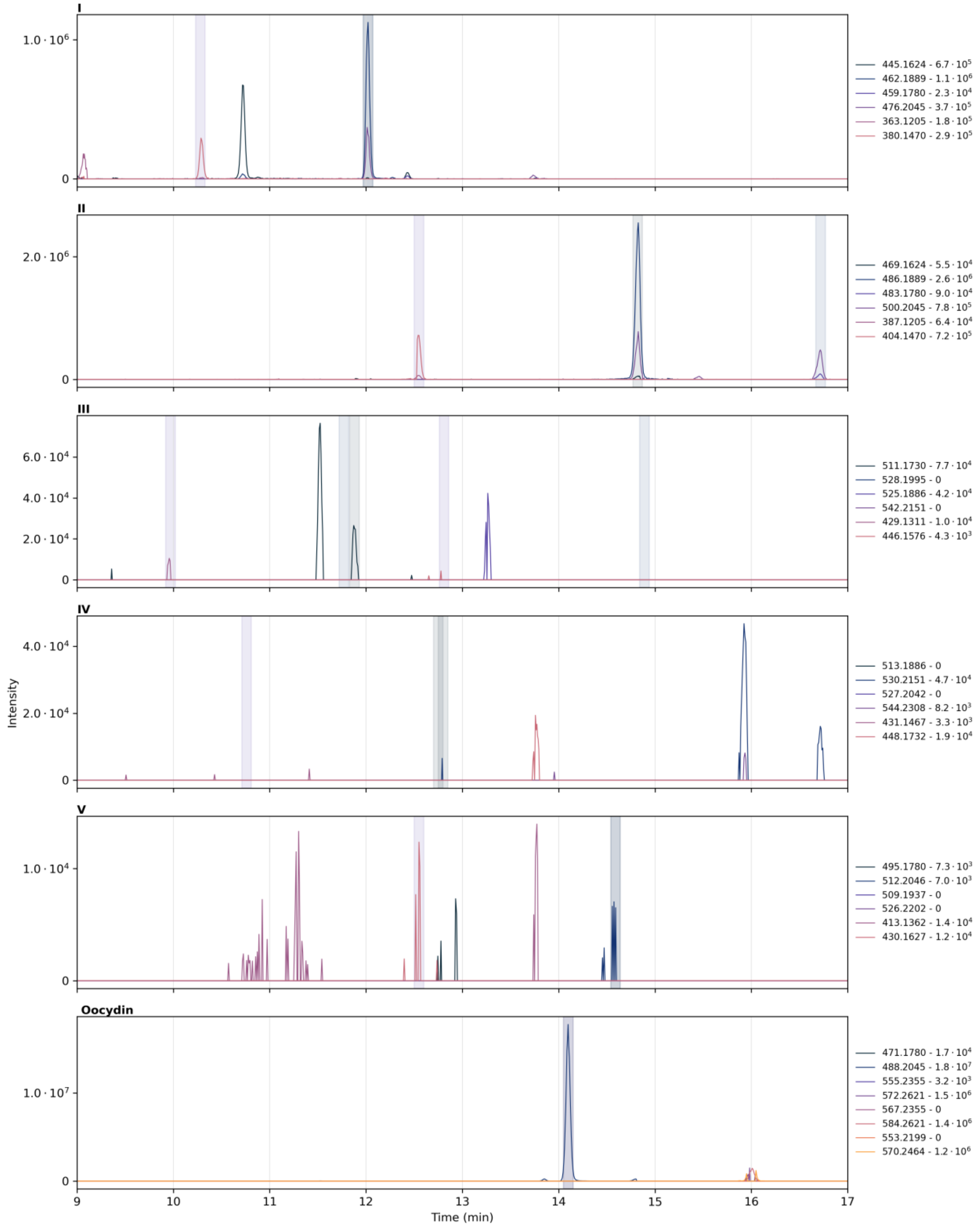

Figure S44 Extracted ion chromatograms of an expression culture extract of *S. plymuthica* 4Rx13 Δ*oocQR* + *pBAD-oocQR-Lcn13-oocS*$_C$. The *m/z* values of proton and ammonium adducts are indicated in the legend of each subplot, with the maximum intensity detected for each *m/z* value. The shaded areas indicate the consensus retention time for the compounds with corresponding color coding.

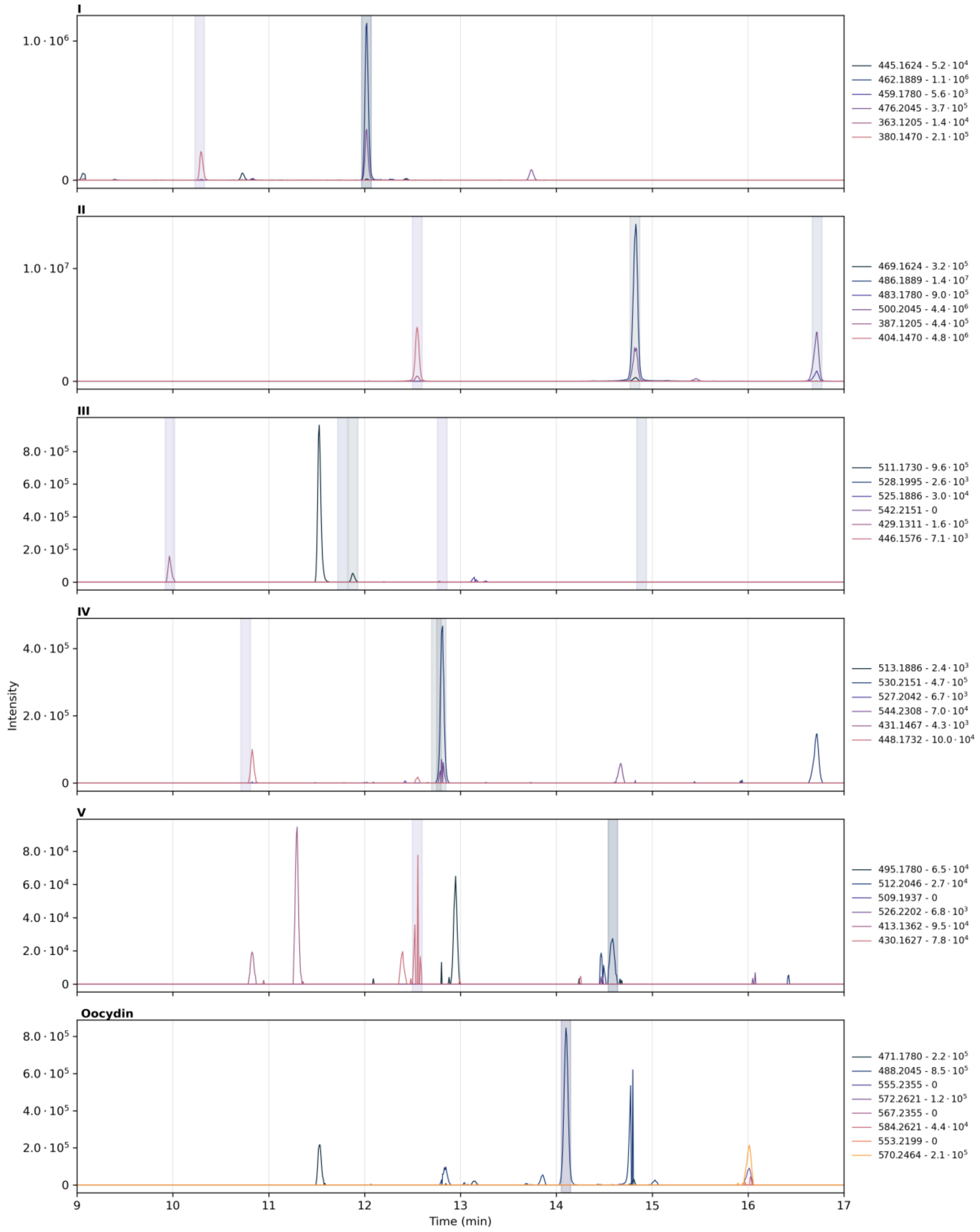

Figure S45 Extracted ion chromatograms of an expression culture extract of *S. plymuthica* 4Rx13 Δ*oocQR* + *pBAD-oocQR-Lcn24-oocS*$_C$. The *m/z* values of proton and ammonium adducts are indicated in the legend of each subplot, with the maximum intensity detected for each *m/z* value. The shaded areas indicate the consensus retention time for the compounds with corresponding color coding.

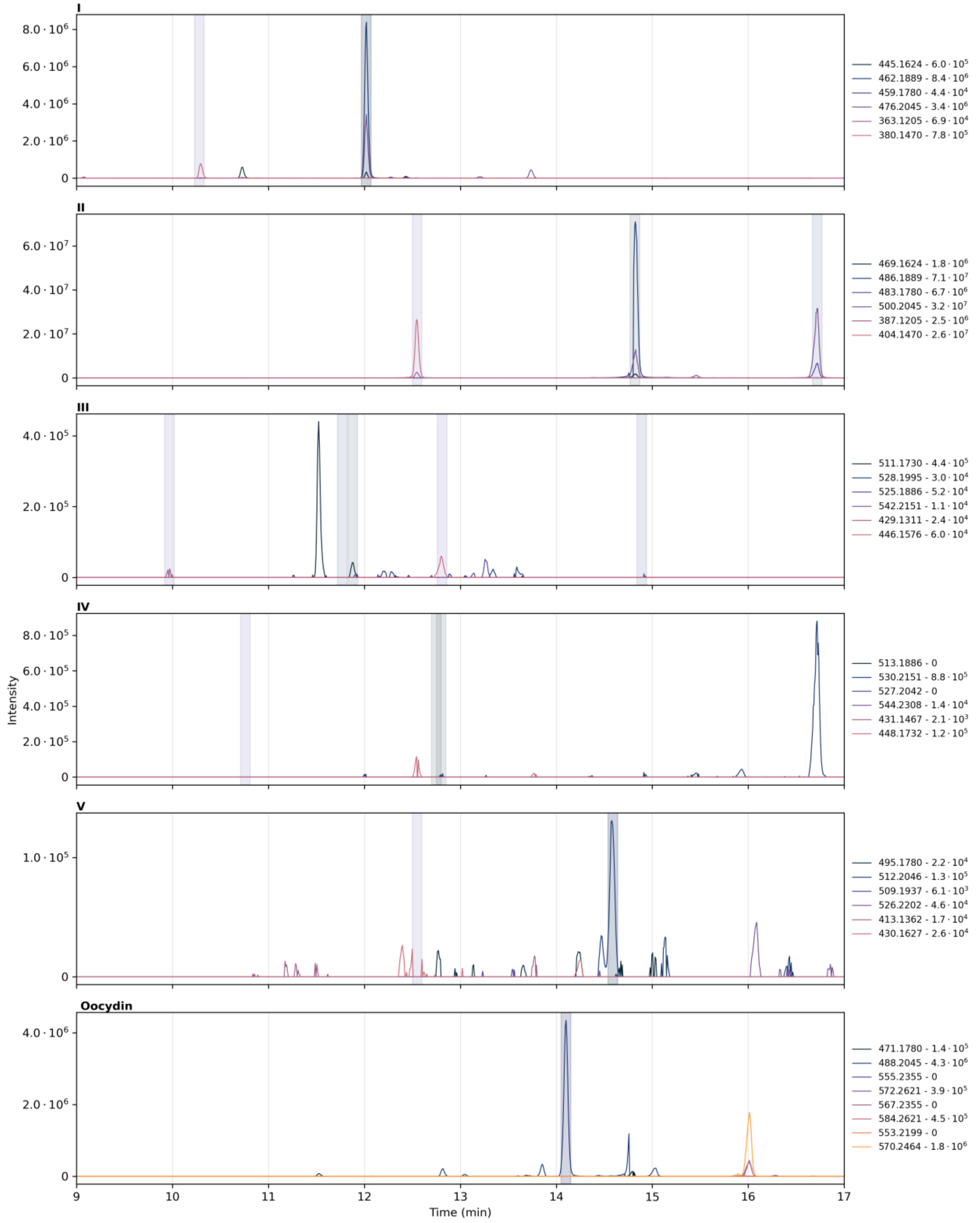

Figure S46 Extracted ion chromatograms of an expression culture extract of *S. plymuthica* 4Rx13 Δ*oocQR pBAD-oocQR-Lcn1-oocS*$_C$. The *m/z* values of proton and ammonium adducts are indicated in the legend of each subplot, with the maximum intensity detected for each *m/z* value. The shaded areas indicate the consensus retention time for the compounds with corresponding color coding.

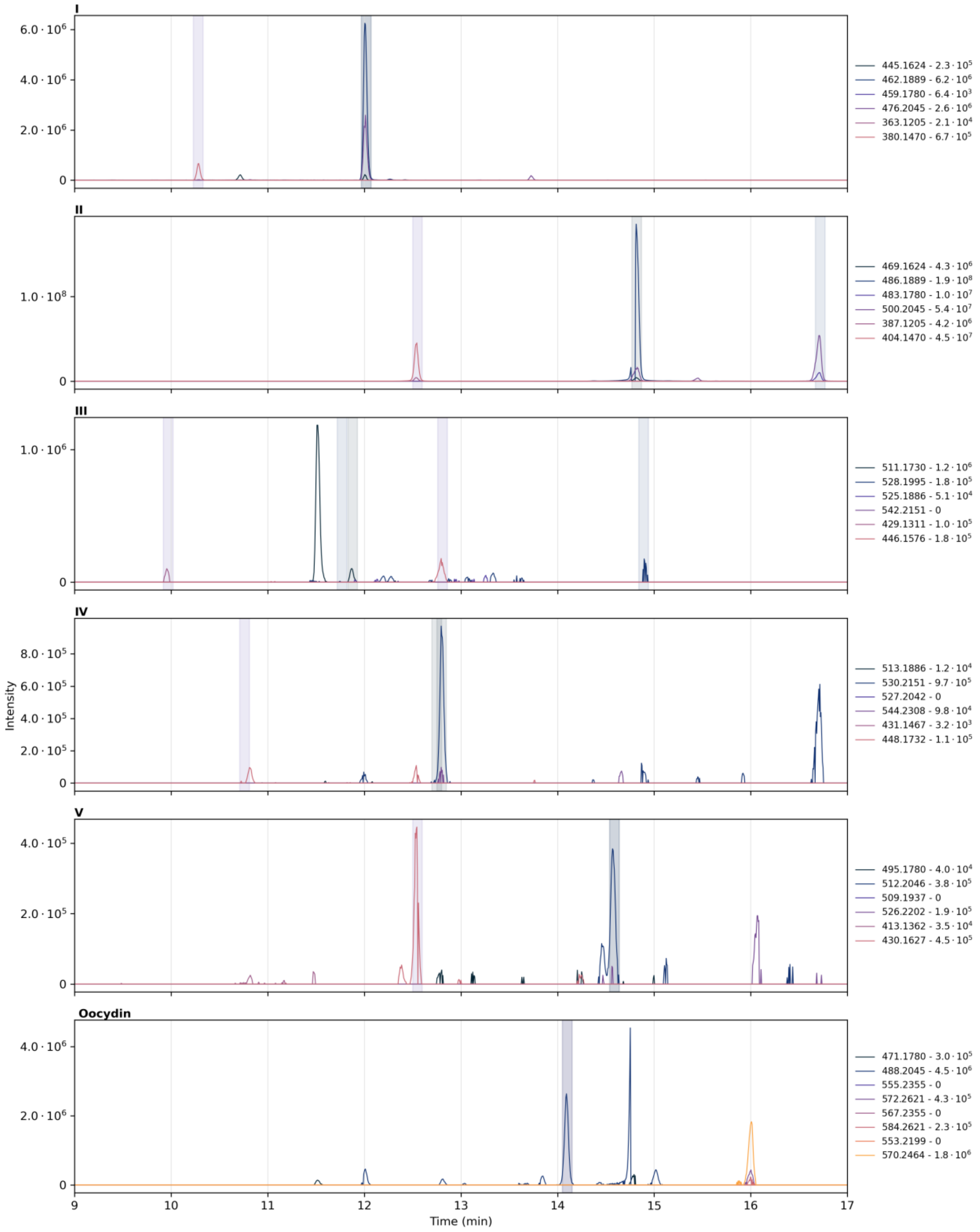

Figure S47 Extracted ion chromatograms of an expression culture extract of *S. plymuthica* 4Rx13 Δ*oocQR* + *oocQR-Tar11-oocS*$_C$. The *m/z* values of proton and ammonium adducts are indicated in the legend of each subplot, with the maximum intensity detected for each *m/z* value. The shaded areas indicate the consensus retention time for the compounds with corresponding color coding.

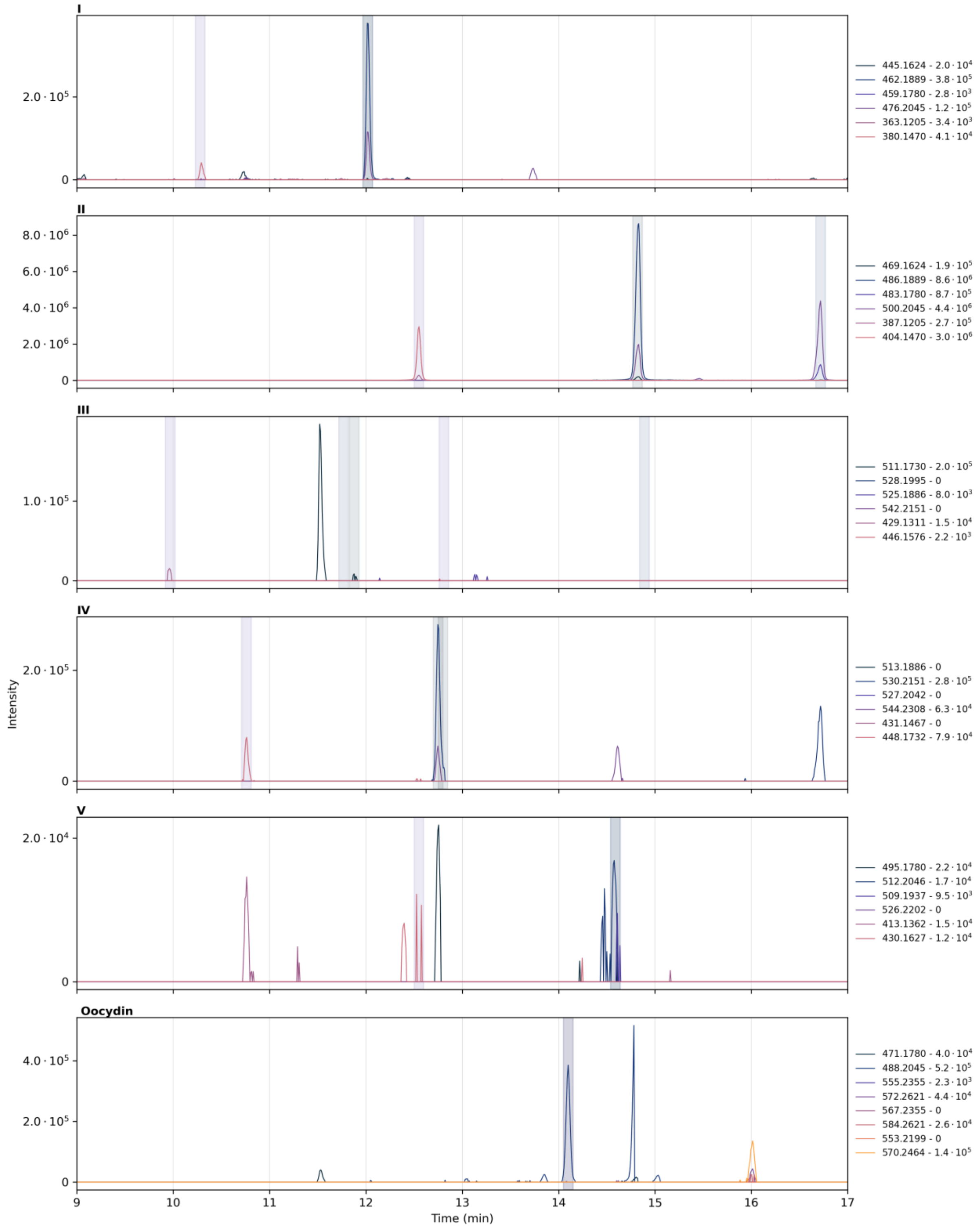

Figure S48 Extracted ion chromatograms of an expression culture extract of *S. plymuthica* 4Rx13 Δ*oocQR* + *pBAD-oocQR-Gyn3-oocS*$_C$. The *m/z* values of proton and ammonium adducts are indicated in the legend of each subplot, with the maximum intensity detected for each *m/z* value. The shaded areas indicate the consensus retention time for the compounds with corresponding color coding.

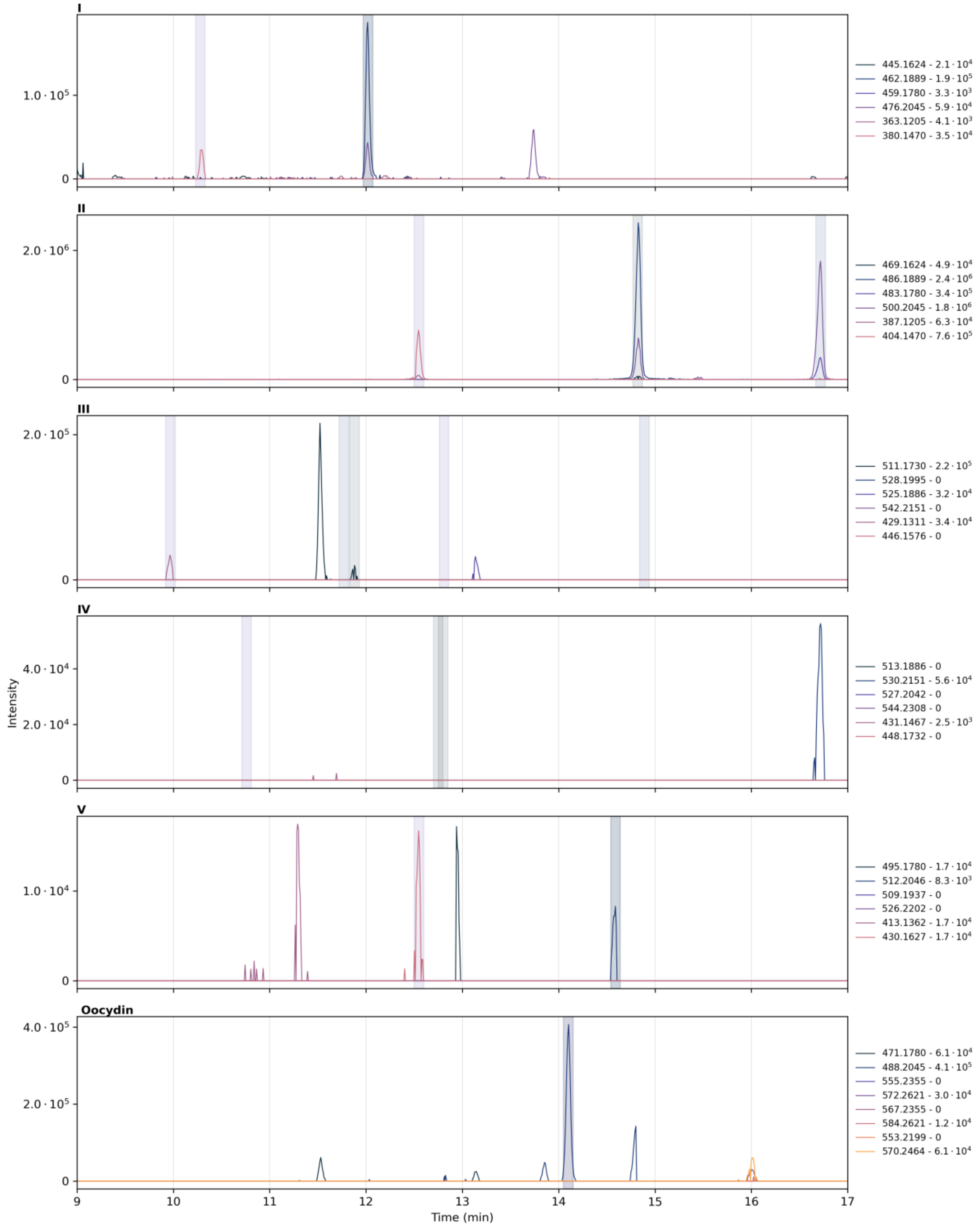

Figure S49 Extracted ion chromatograms of an expression culture extract of *S. plymuthica* 4Rx13 Δ*oocQR* + *pBAD-oocQR-Lcn6-oocS*$_C$. The *m/z* values of proton and ammonium adducts are indicated in the legend of each subplot, with the maximum intensity detected for each *m/z* value. The shaded areas indicate the consensus retention time for the compounds with corresponding color coding.

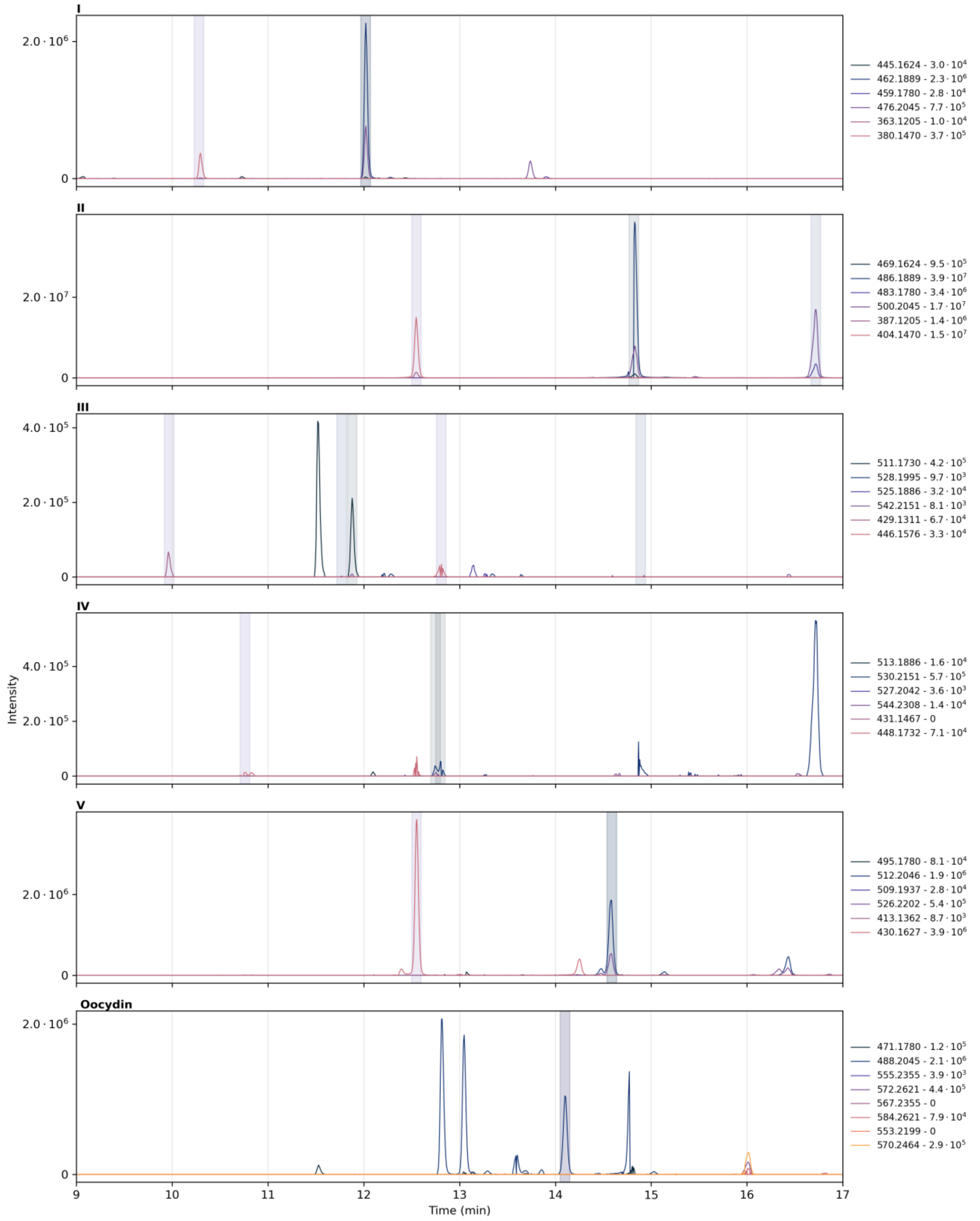

Figure S50 Extracted ion chromatograms of an expression culture extract of *S. plymuthica* 4Rx13 Δ*oocQR* + *pBAD-oocQR-Lbm11*$_{\text{DH-KR-ACP-KS}}$-*oocS*$_{\text{C}}$. The *m/z* values of proton and ammonium adducts are indicated in the legend of each subplot, with the maximum intensity detected for each *m/z* value. The shaded areas indicate the consensus retention time for the compounds with corresponding color coding.

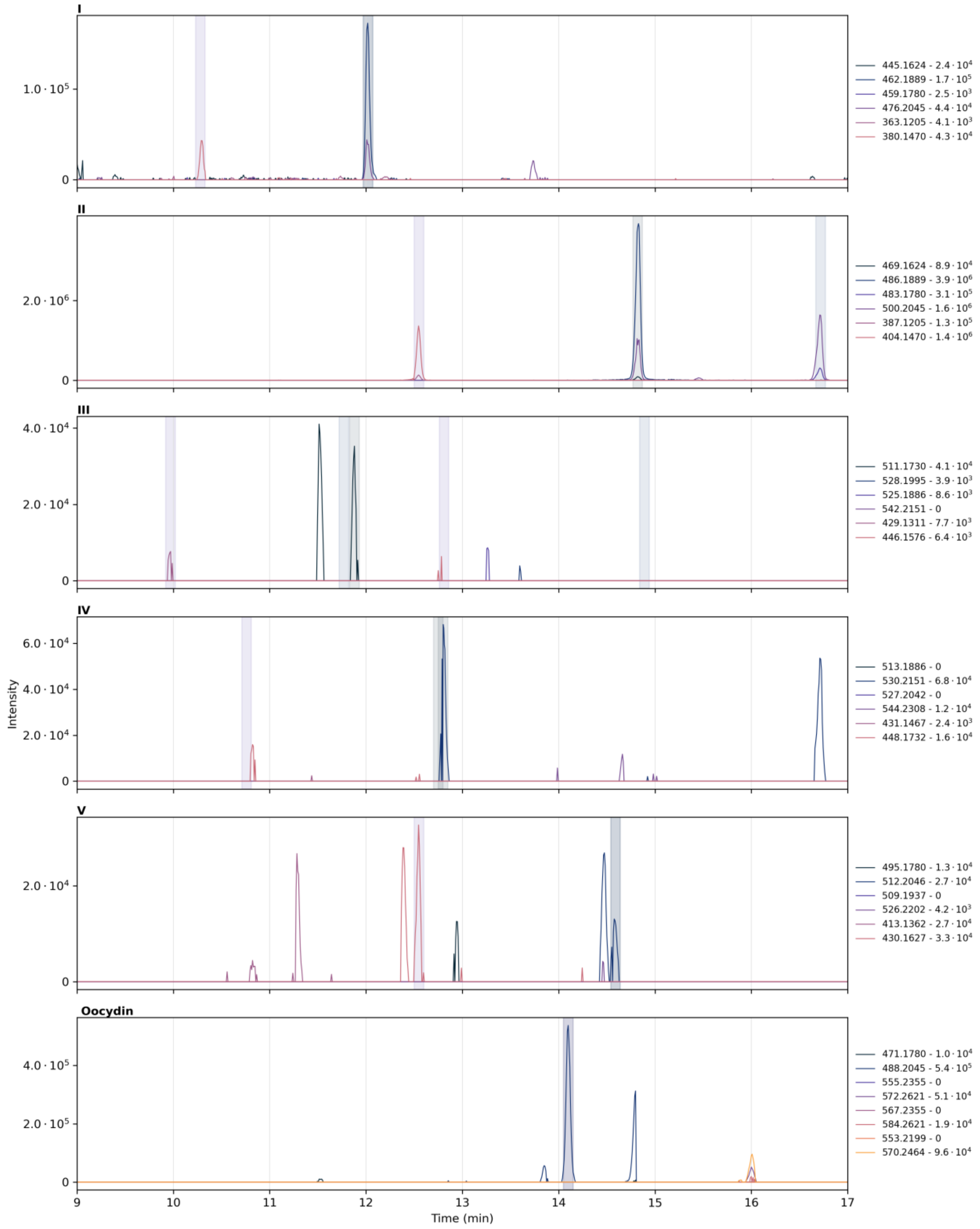

Figure S51 Extracted ion chromatograms of an expression culture extract of *S. plymuthica* 4Rx13 Δ*oocQR* + *pBAD-oocQR-Pks5*$_{\text{DH-KR-ACP-KS}}$-*oocS*$_{\text{C}}$. The *m/z* values of proton and ammonium adducts are indicated in the legend of each subplot, with the maximum intensity detected for each *m/z* value. The shaded areas indicate the consensus retention time for the compounds with corresponding color coding.

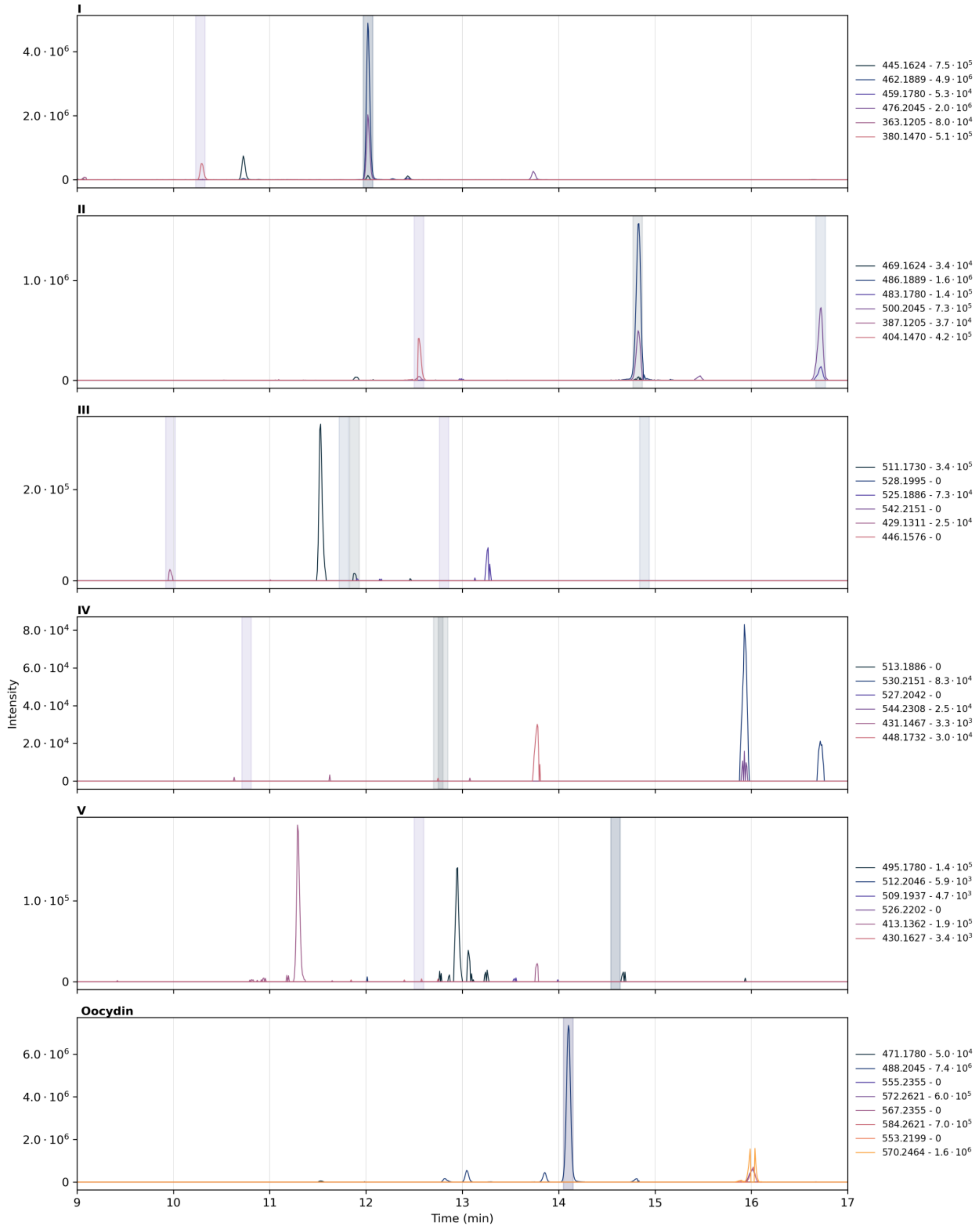

Figure S52 Extracted ion chromatograms of an expression culture extract of *S. plymuthica* 4Rx13 Δ*oocQR* + *pBAD-oocQR-Lbm9-oocS*$_C$. The *m/z* values of proton and ammonium adducts are indicated in the legend of each subplot, with the maximum intensity detected for each *m/z* value. The shaded areas indicate the consensus retention time for the compounds with corresponding color coding.

Table S15 Relative titers of oocydins **1**-**5** produced by the various *Serratia plymuthica* strains presented in Fig. 2-4 in the main text. Relative titers are calculated as fractions by dividing the integrated intensity of the respective shaded peaks in Figs. S27-S52 by the integrated intensity of the oocydin A peak detected in the *S. plymuthica* 4Rx13 wild type extract.

**Mutant**	**1** *m/z* = 462.1897	**2** m/z = 486.1889	**3** m/z = 511.1729	**4** m/z = 530.2151	**5** m/z = 512.2046
S. plymuthica 4Rx13 wild type	$7.84 \cdot 10^{-5}$	$8.11 \cdot 10^{-6}$	$8.20 \cdot 10^{-5}$	0	0
S. plymuthica 4Rx13 *ΔoocQR*	0	0	0	0	0
S. plymuthica 4Rx13 *ΔoocQR + pBAD-oocQR*	$8.04 \cdot 10^{-4}$	$1.82 \cdot 10^{-3}$	$9.61 \cdot 10^{-6}$	0	$1.45 \cdot 10^{-6}$
S. plymuthica 4Rx13 *ΔoocQR + pBAD-oocQR-oocS*$_C$	$1.17 \cdot 10^{-3}$	$6.42 \cdot 10^{-2}$	$1.34 \cdot 10^{-4}$	$2.79 \cdot 10^{-5}$	$9.03 \cdot 10^{-5}$
S. plymuthica 4Rx13 *ΔoocQR + pBAD-oocQR-Psy*$_{KS11}$<br>Fusion site: LPTYPFx_5W	$7.39 \cdot 10^{-3}$	$7.05 \cdot 10^{-4}$	$5.66 \cdot 10^{-4}$	0	0
S. plymuthica 4Rx13 *ΔoocQR + pBAD-oocQR-Psy*$_{KS11}$<br>Fusion site: NAHVILEE	$2.85 \cdot 10^{-4}$	0	0	0	0
S. plymuthica 4Rx13 *ΔoocQR + pBAD-oocQR-Lbm12-oocS*$_C$<br>Upstream*:* LPTYPFx_5W, downstream: LPTYPFx_5W	$1.22 \cdot 10^{-3}$	$1.09 \cdot 10^{-2}$	$2.82 \cdot 10^{-3}$	$1.43 \cdot 10^{-3}$	$2.65 \cdot 10^{-5}$
S. plymuthica 4Rx13 *ΔoocQR + pBAD-oocQR-Lbm12-oocS*$_C$<br>Upstream*:* NAHVILEE, downstream: LPTYPFx_5W	$4.67 \cdot 10^{-4}$	$5.15 \cdot 10^{-3}$	$3.09 \cdot 10^{-5}$	$2.92 \cdot 10^{-6}$	$1.30 \cdot 10^{-5}$
S. plymuthica 4Rx13 *ΔoocQR + pBAD-oocQR-Lbm12-oocS*$_C$<br>Upstream*:* LPTYPFx_5W, downstream: NAHVILEE	$2.17 \cdot 10^{-4}$	$5.01 \cdot 10^{-3}$	$3.57 \cdot 10^{-5}$	$4.43 \cdot 10^{-6}$	$1.22 \cdot 10^{-5}$
S. plymuthica 4Rx13 *ΔoocQR + pBAD-oocQR-Lbm12-oocS*$_C$<br>Upstream*:* NAHVILEE, downstream: NAHVILEE	$2.89 \cdot 10^{-4}$	$5.15 \cdot 10^{-3}$	$2.96 \cdot 10^{-5}$	$6.44 \cdot 10^{-6}$	$1.03 \cdot 10^{-5}$
S. plymuthica 4Rx13 *ΔoocQR + pBAD-oocQR-Lbm11-oocS*$_C$	$5.74 \cdot 10^{-4}$	$2.99 \cdot 10^{-2}$	$3.34 \cdot 10^{-4}$	$9.13 \cdot 10^{-6}$	$7.77 \cdot 10^{-5}$
S. plymuthica 4Rx13 *ΔoocQR + pBAD-oocQR-Pks5-oocS*$_C$	$2.10 \cdot 10^{-4}$	$4.96 \cdot 10^{-3}$	$1.29 \cdot 10^{-4}$	$5.70 \cdot 10^{-5}$	$4.88 \cdot 10^{-5}$
S. plymuthica 4Rx13 *ΔoocQR + pBAD-oocQR-Tar10-oocS*$_C$	$1.47 \cdot 10^{-3}$	$3.65 \cdot 10^{-3}$	$9.62 \cdot 10^{-5}$	$4.73 \cdot 10^{-3}$	$8.83 \cdot 10^{-5}$
S. plymuthica 4Rx13 *ΔoocQR + pBAD-oocQR-Tar13-oocS*$_C$	$2.69 \cdot 10^{-3}$	$1.49 \cdot 10^{-1}$	$1.89 \cdot 10^{-4}$	$7.16 \cdot 10^{-4}$	$3.17 \cdot 10^{-4}$
S. plymuthica 4Rx13 *ΔoocQR + pBAD-oocQR-Lcn13-oocS*$_C$	$1.10 \cdot 10^{-3}$	$3.12 \cdot 10^{-3}$	$2.52 \cdot 10^{-5}$	$1.09 \cdot 10^{-6}$	$4.79 \cdot 10^{-6}$
S. plymuthica 4Rx13 *ΔoocQR + pBAD-oocQR-Lcn24-oocS*$_C$	$9.95 \cdot 10^{-4}$	$1.67 \cdot 10^{-2}$	$4.58 \cdot 10^{-5}$	$4.36 \cdot 10^{-4}$	$3.62 \cdot 10^{-5}$
S. plymuthica 4Rx13 *ΔoocQR + pBAD-oocQR-Lcn1-oocS*$_C$	$7.35 \cdot 10^{-3}$	$6.81 \cdot 10^{-2}$	$3.92 \cdot 10^{-5}$	$5.37 \cdot 10^{-4}$	$1.77 \cdot 10^{-4}$
S. plymuthica 4Rx13 *ΔoocQR + pBAD-oocQR-Tar11-oocS*$_C$	$5.73 \cdot 10^{-3}$	$1.51 \cdot 10^{-2}$	$9.55 \cdot 10^{-5}$	$9.79 \cdot 10^{-4}$	$4.81 \cdot 10^{-4}$
S. plymuthica 4Rx13 *ΔoocQR + pBAD-oocQR-Gyn3-oocS*$_C$	$3.47 \cdot 10^{-4}$	$1.05 \cdot 10^{-2}$	$4.68 \cdot 10^{-6}$	$2.84 \cdot 10^{-4}$	$1.80 \cdot 10^{-5}$
S. plymuthica 4Rx13 *ΔoocQR + pBAD-oocQR-Lcn6-oocS*$_C$	$1.84 \cdot 10^{-4}$	$2.94 \cdot 10^{-3}$	$1.28 \cdot 10^{-5}$	0	$7.23 \cdot 10^{-6}$
S. plymuthica 4Rx13 *ΔoocQR + pBAD-oocQR-Lbm11*$_{DH-KR-ACP-KS}$*-oocS*$_C$	$1.91 \cdot 10^{-3}$	$2.92 \cdot 10^{-2}$	$1.80 \cdot 10^{-4}$	$6.13 \cdot 10^{-5}$	$2.17 \cdot 10^{-3}$
S. plymuthica 4Rx13 *ΔoocQR + pBAD-oocQR-Pks5*$_{DH-KR-ACP-KS}$*-oocS*$_C$	$1.78 \cdot 10^{-4}$	$4.97 \cdot 10^{-3}$	$3.11 \cdot 10^{-5}$	$5.69 \cdot 10^{-5}$	$1.85 \cdot 10^{-5}$
S. plymuthica 4Rx13 *ΔoocQR + pBAD-oocQR-Lbm9-oocS*$_C$	$4.28 \cdot 10^{-3}$	$1.89 \cdot 10^{-3}$	$1.03 \cdot 10^{-5}$	0	0

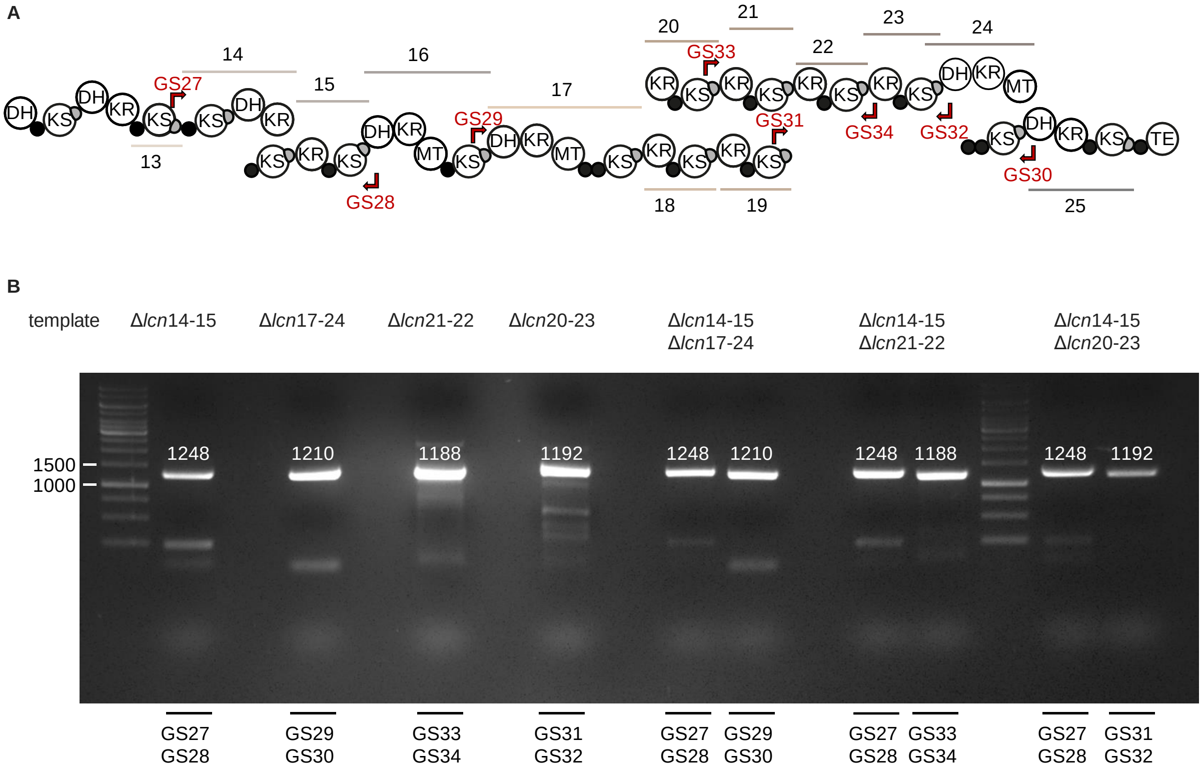

Figure S53 Verification of *Gynuella sunshinyii* deletion mutants. (**A**) Schematic representation of binding sites for primers GS27-GS34 (Table S10) in the wild-type strain *lcn* BGC (shown at the protein level. (**B**) Agarose gel of PCRs verifying the deletion in the seven different *G. sunshinyii* mutants mentioned on top. Deletion of exchange units incorporating KSs (EXU) 14-15 (GS27, GS28): 1.2 kbp instead of 9.4 kbp in the wild type; deletion of (EXU) 17-24 (GS29, GS30): 1.2 kbp instead of 36.4 kbp in the wild type; deletion of (EXU) 21-22 (GS33, GS34): 1.2 kbp instead of 8.7 kbp in the wild type; deletion of (EXU)20-23 (GS31, GS33): 1.2 kbp instead of 16.4 kbp in the wild type. Expected PCR product sizes (in bp) are shown in white. PCR bands were purified and sequenced confirming fusion at the designed site.

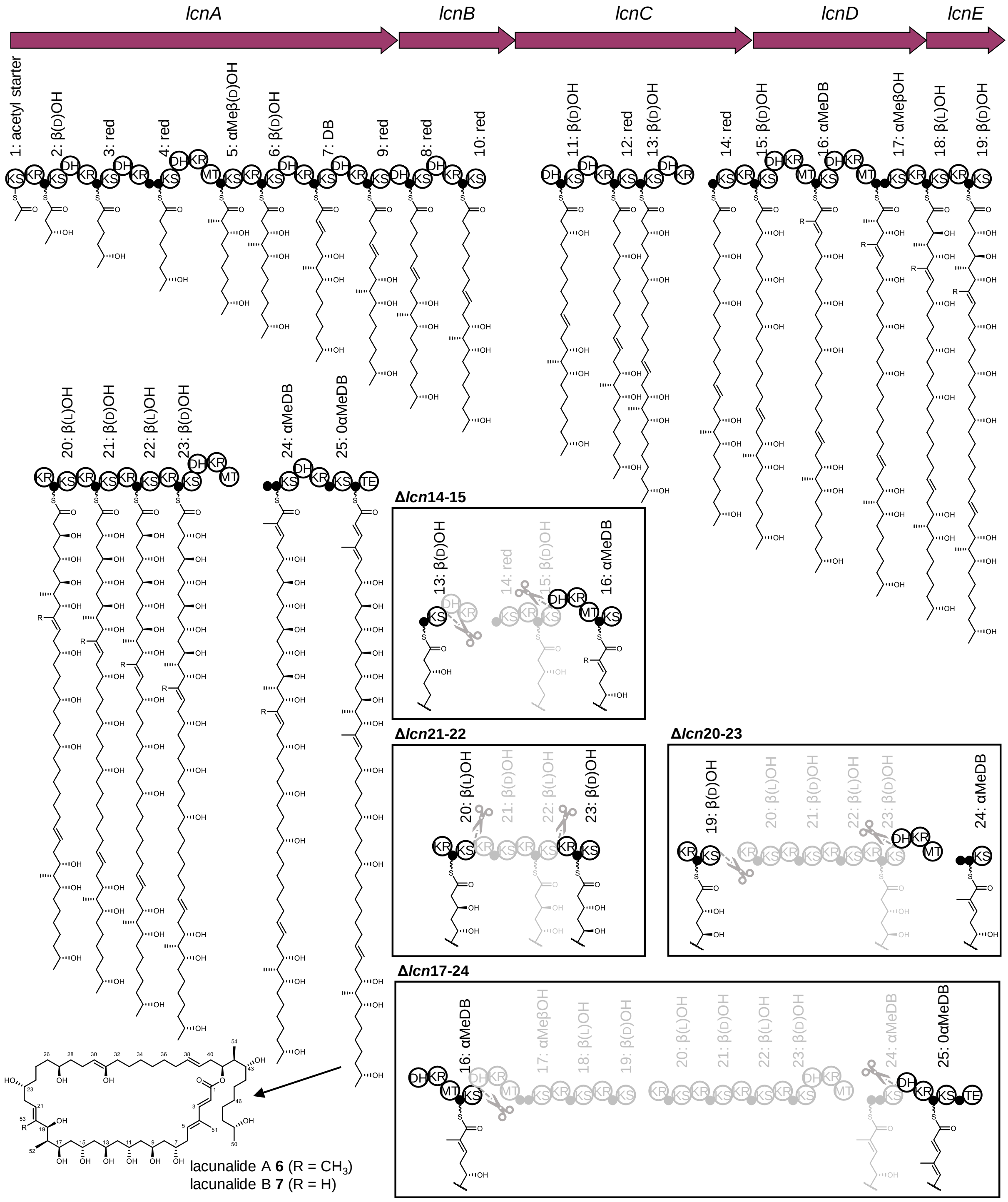

Figure S54 Lacunalides are biosynthesized by five *trans*-AT PKS proteins with a total of 25 modules. The incoming substrate's α-β region is mentioned above each KS. α/β: location relative to the thioester; (L/D): configuration relative to the thioester; OH: hydroxy group; Me: methyl group; DB: double bond; red: fully reduced α/β region. Insets: To generate functional truncated PKSs, a series of domains after a KSs accepting an intermediate type (eg. KS13: β(D)OH for Δ*lcn*14-15) up to a KS with the same intermediate type (eg. KS15: β(D)OH for Δ*lcn*14-15) were deleted. The LPTYPFx_5W motif was used as a fusion site (scissors). Exchange units deleted in the respective mutants are shown in grey. In all mutants the intermediate processed by the KS upstream of the fusion site (black on the left-hand side) is identical to that in the wild

type (grey). Deleting domain series spanning multiple proteins resulted in protein fusions (eg. LcnB fused to LcnC in the Δ*lcn*14-15 mutant).

Extracted ion chromatograms for 6-21 of cultures of *Gynuella sunshinyii* mutants

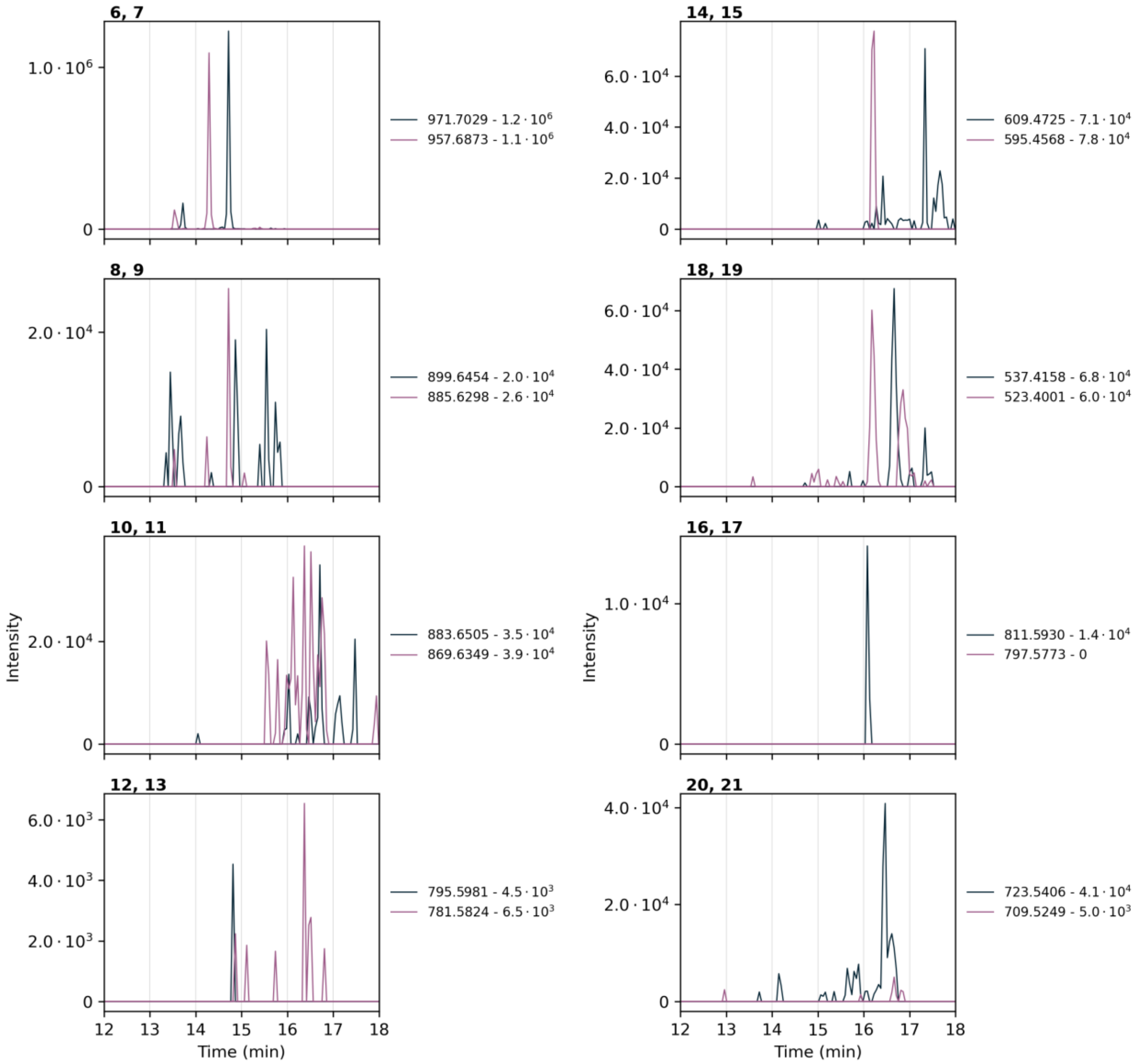

Figure S55 Extracted ion chromatograms of an expression culture extract of wild type *G. sunshinyii* YC6258. The *m/z* values of proton adducts of respective compounds are indicated in the legend of each subplot, with the maximum intensity detected for each *m/z* value.

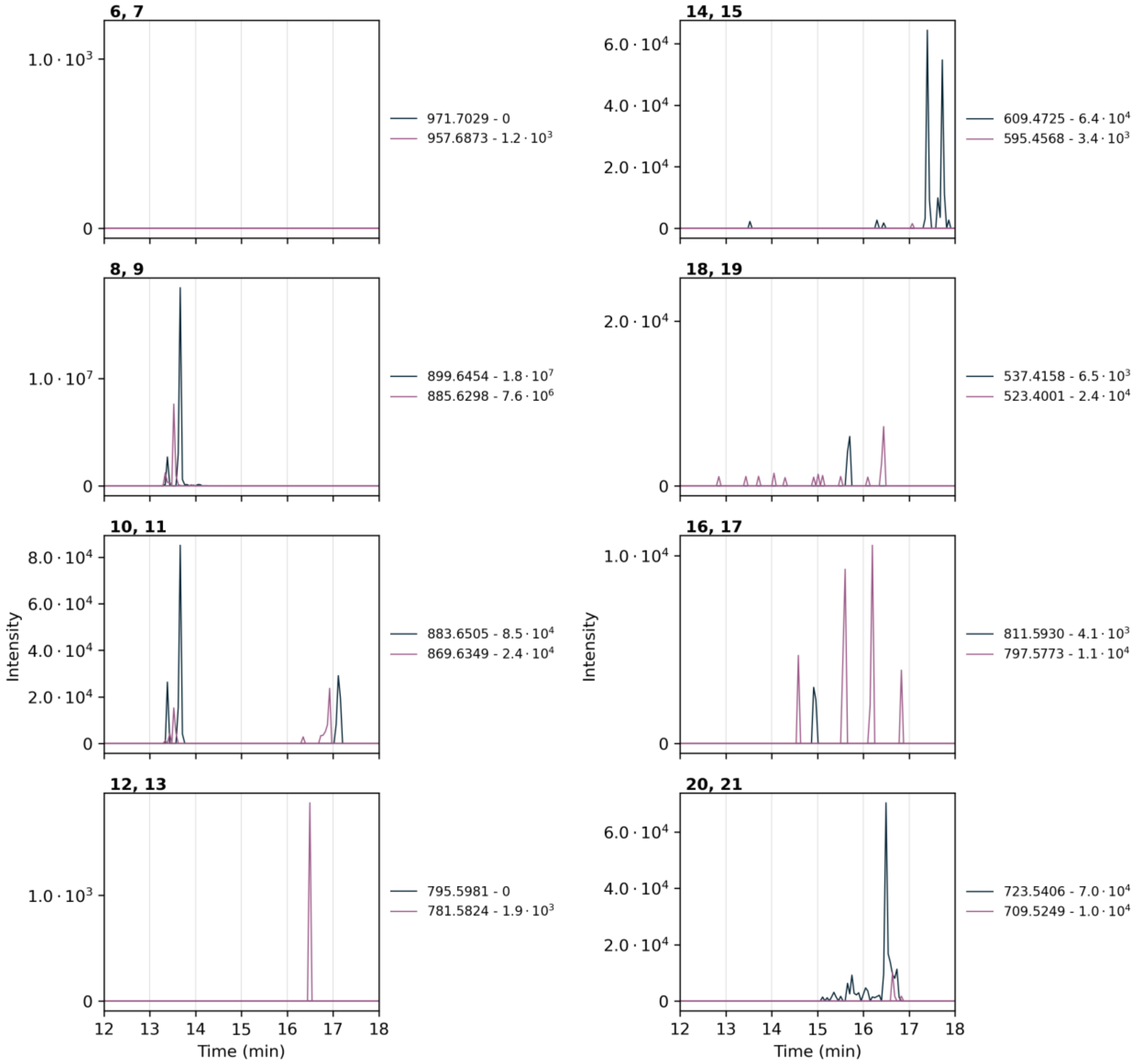

Figure S56 Extracted ion chromatograms of an expression culture extract of *G. sunshinyii* YC6258 Δ*lcn*14-15. The *m/z* values of proton adducts of respective compounds are indicated in the legend of each subplot, with the maximum intensity detected for each *m/z* value.

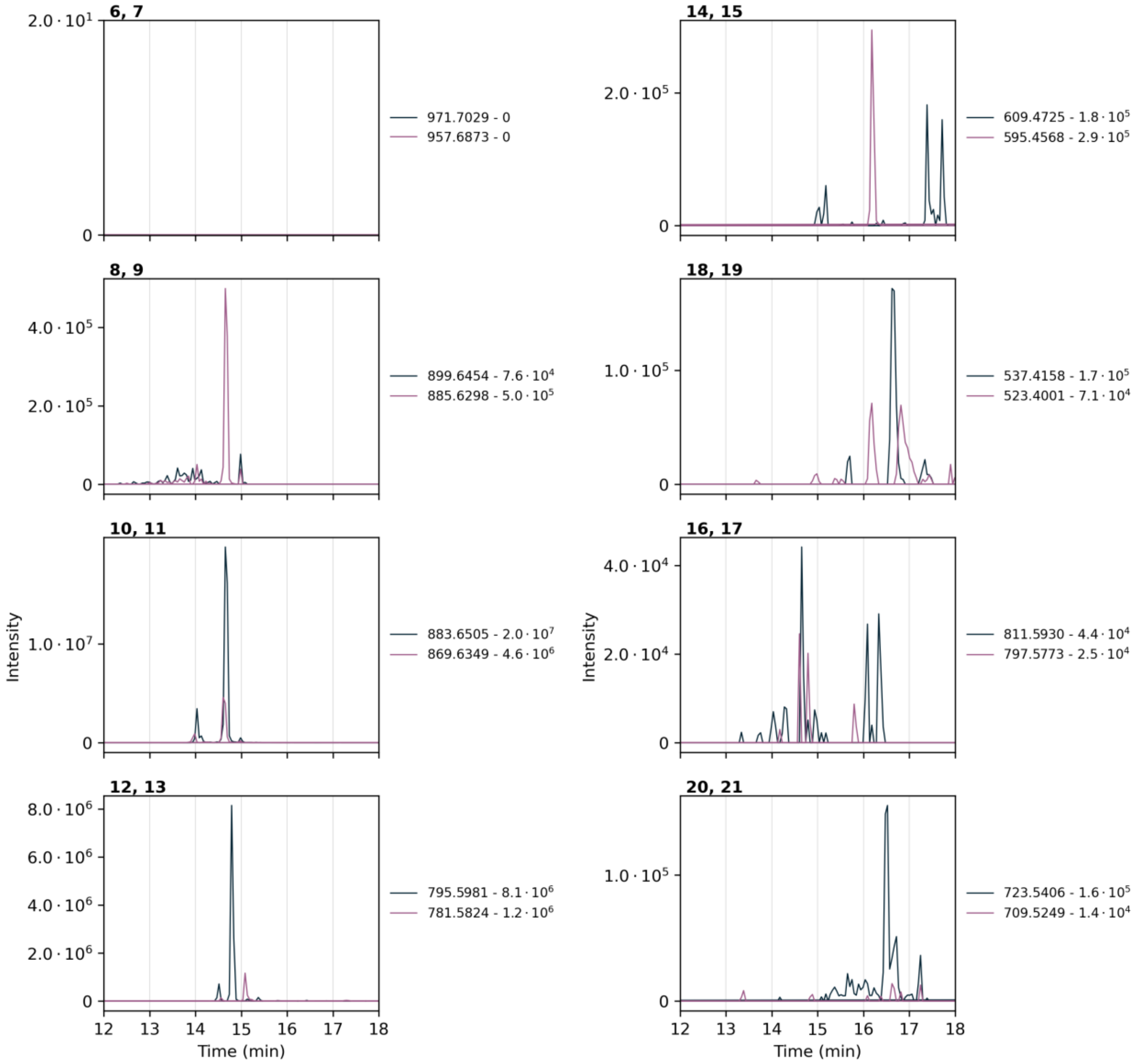

Figure S57 Extracted ion chromatograms of an expression culture extract of *G. sunshinyii* YC6258 Δ*lcn*21-22. The *m/z* values of proton adducts of respective compounds are indicated in the legend of each subplot, with the maximum intensity detected for each *m/z* value.

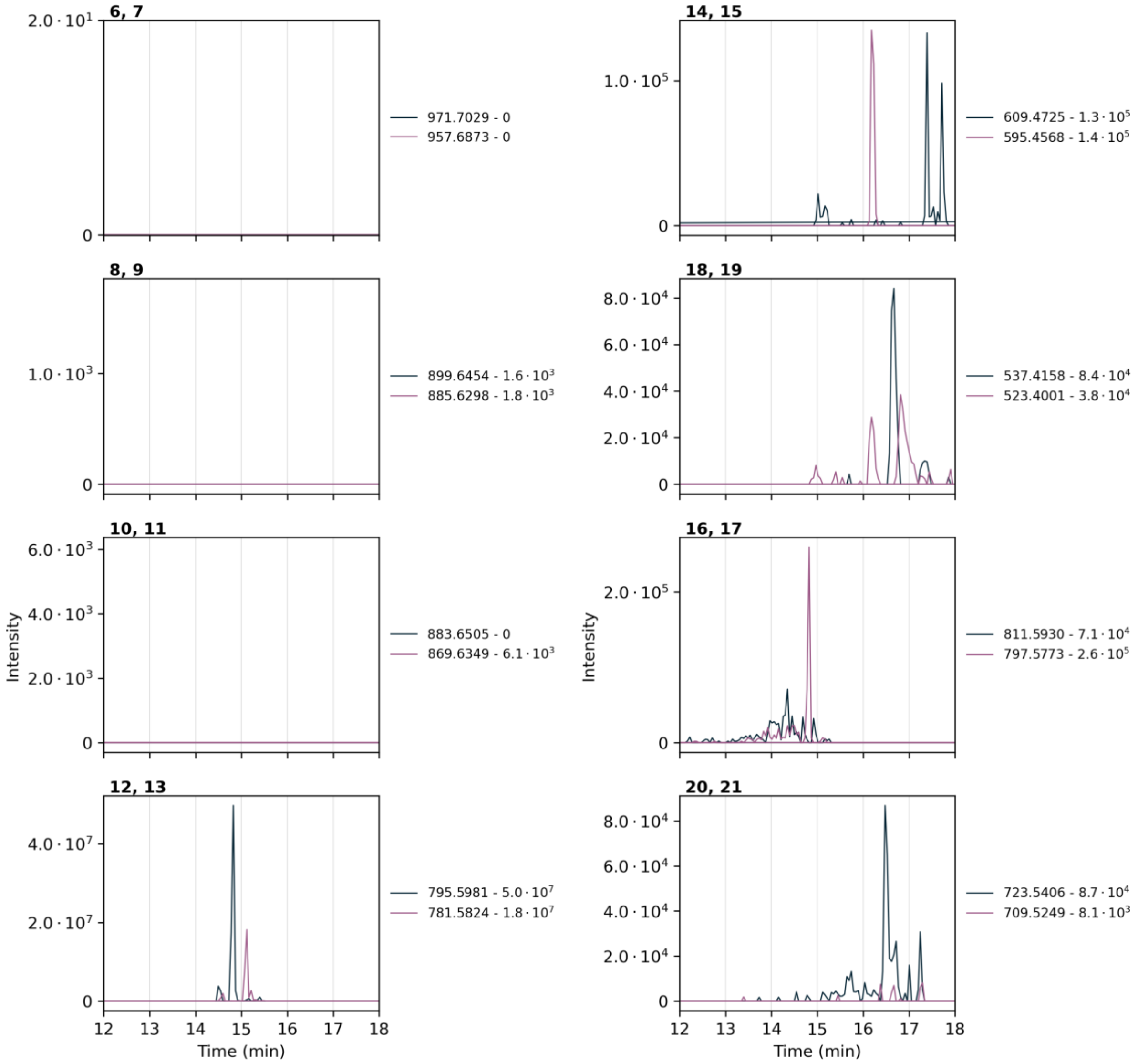

Figure S58 Extracted ion chromatograms of an expression culture extract of *G. sunshinyii* YC6258 Δ*lcn*20-23. The *m/z* values of proton adducts of respective compounds are indicated in the legend of each subplot, with the maximum intensity detected for each *m/z* value.

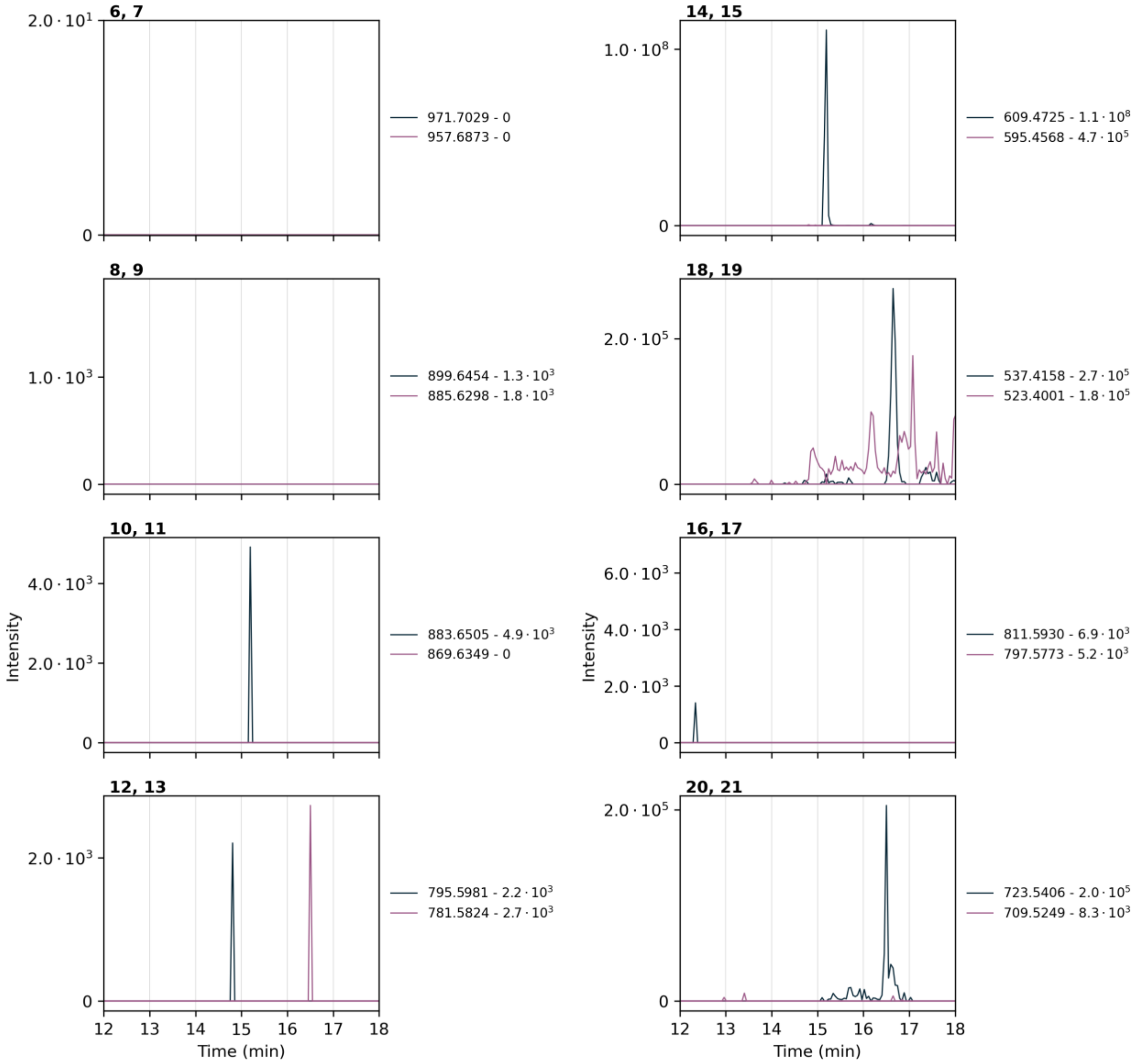

Figure S59 Extracted ion chromatograms of an expression culture extract of *G. sunshinyii* YC6258 Δ*lcn*17-24. The *m/z* values of proton adducts of respective compounds are indicated in the legend of each subplot, with the maximum intensity detected for each *m/z* value.

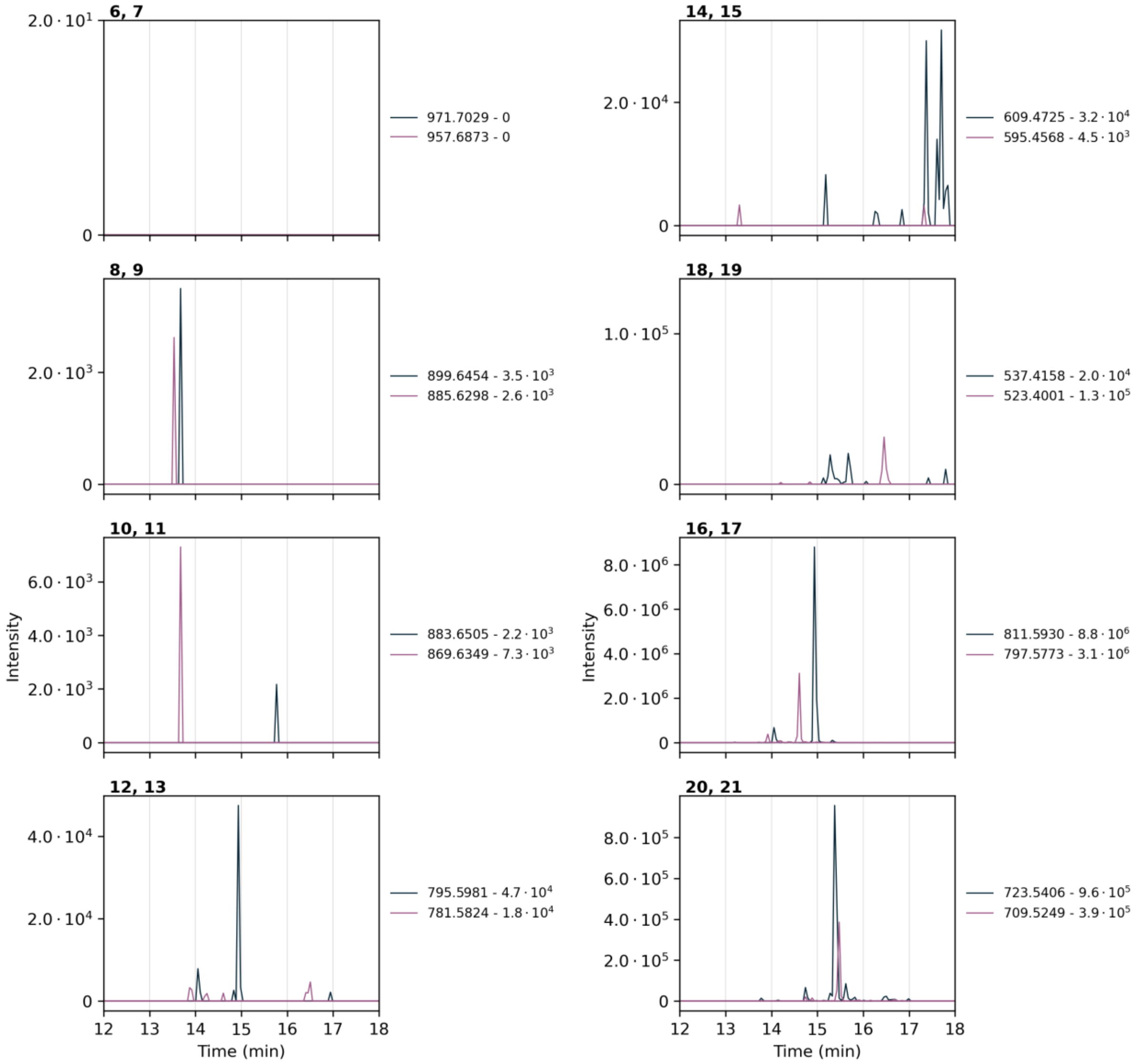

Figure S60 Extracted ion chromatograms of an expression culture extract of *G. sunshinyii* YC6258 Δ*lcn*14-15, Δ*lcn*21-22. The *m/z* values of proton adducts of respective compounds are indicated in the legend of each subplot, with the maximum intensity detected for each *m/z* value.

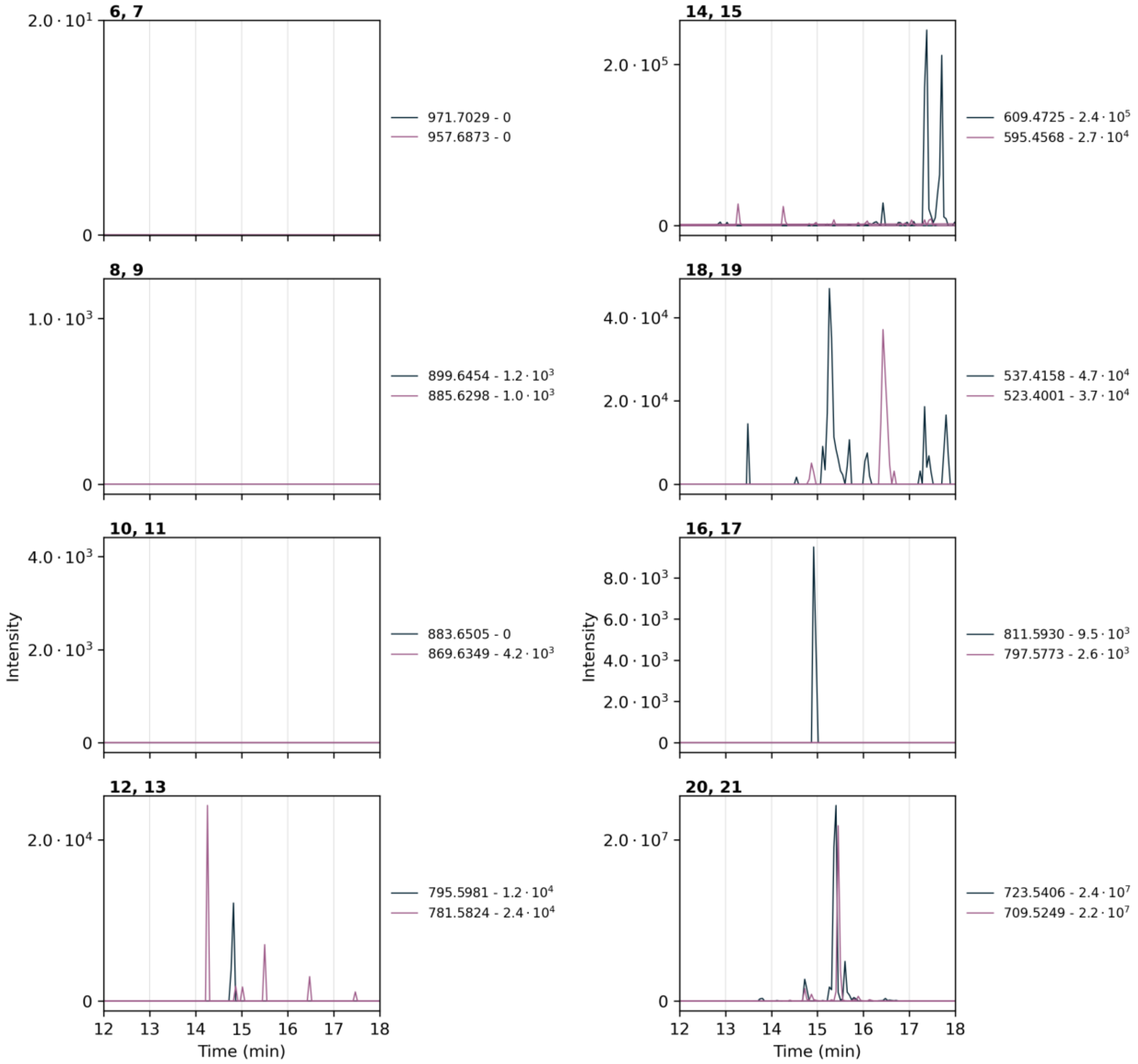

Figure S61 Extracted ion chromatograms of an expression culture extract of *G. sunshinyii* YC6258 Δ*lcn*14-15, Δ*lcn*20-23. The *m/z* values of proton adducts of respective compounds are indicated in the legend of each subplot, with the maximum intensity detected for each *m/z* value.

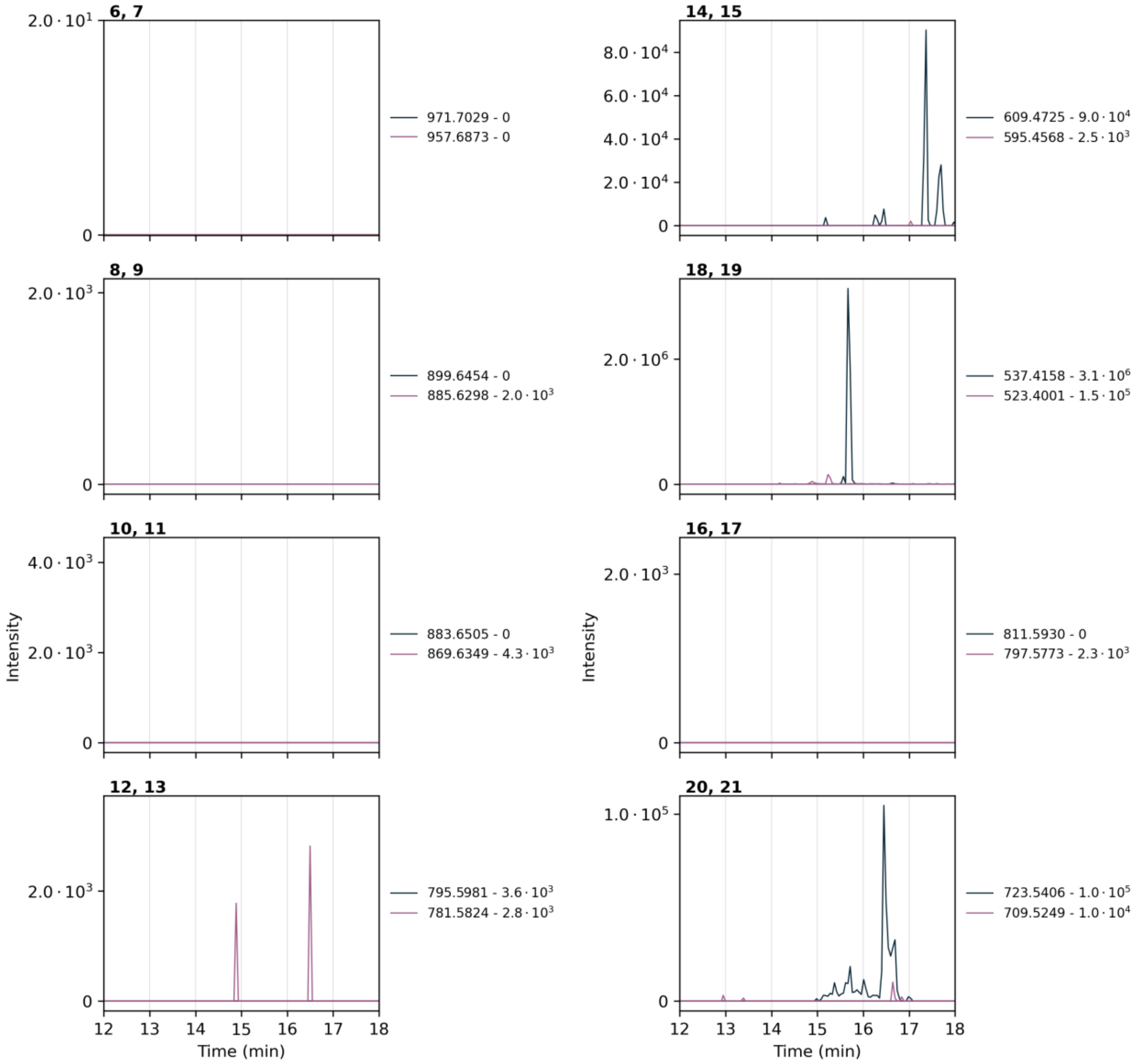

Figure S62 Extracted ion chromatograms of an expression culture extract of *G. sunshinyii* YC6258 Δ*lcn*14-15, Δ*lcn*17-24. The *m/z* values of proton adducts of respective compounds are indicated in the legend of each subplot, with the maximum intensity detected for each *m/z* value.

Table S16 Relative titers of the lacunalides **6**-**21** produced by the various *Gynuella sunshinyii* strains presented in Fig. 5 in the main text. Relative titers are calculated as fractions by dividing the integrated intensity of the respective shaded peaks in Figs. S55-S62 by the integrated intensity of the lacunalide A peak detected in the *G. sunshinyii* wild type extract.

**Mutant**	**6**<br>*m/z* = 971.7029	**7**<br>*m/z* = 957.6873	**8**<br>*m/z* = 899.6455	**9**<br>*m/z* = 885.6298	**10**<br>*m/z* =883.6505	**11**<br>*m/z* = 869.6349
Gynuella sunshinyii wild type	$1.00 \cdot 10^{0}$	$8.96 \cdot 10^{-1}$	$1.57 \cdot 10^{-2}$	$4.58 \cdot 10^{-3}$	0	0
Gynuella sunshinyii Δ*lcn*14-15	0	0	$1.98 \cdot 10^{-1}$	$7.52 \cdot 10^{-2}$	0	0
Gynuella sunshinyii Δ*lcn*21-22	0	0	$1.00 \cdot 10^{-1}$	$1.62 \cdot 10^{-2}$	$3.31 \cdot 10^{1}$	$8.28 \cdot 10^{1}$
Gynuella sunshinyii Δ*lcn*20-23	0	0	0	0	0	0
Gynuella sunshinyii Δ*lcn*17-24	0	0	0	0	0	0
Gynuella sunshinyii Δ*lcn*14-15, Δ*lcn*21-22	0	0	$3.35 \cdot 10^{-3}$	$2.57 \cdot 10^{-3}$	0	0
Gynuella sunshinyii Δ*lcn*14-15, Δ*lcn*20-23	0	0	0	0	0	0
Gynuella sunshinyii Δ*lcn*14-15, Δ*lcn*17-24	0	0	0	0	0	0

**Mutant**	**12**<br>*m/z* = 795.5981	**13**<br>*m/z* = 781.5824	**14**<br>*m/z* = 609.4725	**15**<br>*m/z* = 595.4568	**16**<br>*m/z* = 811.5930	**17**<br>*m/z* = 797.5773
Gynuella sunshinyii wild type	$4.57 \cdot 10^{-3}$	$1.87 \cdot 10^{-3}$	$2.09 \cdot 10^{-3}$	0	$5.15 \cdot 10^{-3}$	$2.24 \cdot 10^{-3}$
Gynuella sunshinyii Δ*lcn*14-15	0	0	0	0	$8.59 \cdot 10^{-5}$	0
Gynuella sunshinyii Δ*lcn*21-22	$1.06 \cdot 10^{1}$	$1.42 \cdot 10^{2}$	$6.48 \cdot 10^{1}$	0	$3.83 \cdot 10^{1}$	0
Gynuella sunshinyii Δ*lcn*20-23	$5.17 \cdot 10^{1}$	$2.19 \cdot 10^{1}$	$2.55 \cdot 10^{-2}$	0	$4.26 \cdot 10^{-3}$	0
Gynuella sunshinyii Δ*lcn*17-24	$2.20 \cdot 10^{-3}$	0	$1.53 \cdot 10^{2}$	$4.49 \cdot 10^{-1}$	$1.25 \cdot 10^{-2}$	$4.22 \cdot 10^{-2}$
Gynuella sunshinyii Δ*lcn*14-15, Δ*lcn*21-22	$2.59 \cdot 10^{-3}$	0	$8.33 \cdot 10^{-3}$	0	$3.17 \cdot 10^{-2}$	0
Gynuella sunshinyii Δ*lcn*14-15, Δ*lcn*20-23	$1.23 \cdot 10^{-2}$	0	$1.91 \cdot 10^{-3}$	$4.17 \cdot 10^{-3}$	$1.13 \cdot 10^{-2}$	0
Gynuella sunshinyii Δ*lcn*14-15, Δ*lcn*17-24	0	0	$3.73 \cdot 10^{-3}$	0	$4.97 \cdot 10^{0}$	$2.56 \cdot 10^{-1}$

**Mutant**	**18**<br>*m/z* = 537.4158	**19**<br>*m/z* = 523.4001	**20**<br>*m/z* = 723.5406	**21**<br>*m/z* = 709.5249
Gynuella sunshinyii wild type	0	0	$1.88 \cdot 10^{-2}$	0
Gynuella sunshinyii Δ*lcn*14-15	$5.14 \cdot 10^{-5}$	$4.54 \cdot 10^{-5}$	$5.00 \cdot 10^{-5}$	0
Gynuella sunshinyii Δ*lcn*21-22	$1.23 \cdot 10^{-2}$	$1.45 \cdot 10^{-2}$	$2.28 \cdot 10^{-2}$	0
Gynuella sunshinyii Δ*lcn*20-23	$4.27 \cdot 10^{-2}$	$1.73 \cdot 10^{-2}$	$0.98 \cdot 10^{-2}$	$1.62 \cdot 10^{-3}$
Gynuella sunshinyii Δ*lcn*17-24	0	0	1.446	0
Gynuella sunshinyii Δ*lcn*14-15, Δ*lcn*21-22	$1.06 \cdot 10^{1}$	$2.29 \cdot 10^{0}$	$1.43 \cdot 10^{0}$	$4.42 \cdot 10^{-1}$
Gynuella sunshinyii Δ*lcn*14-15, Δ*lcn*20-23	$1.41 \cdot 10^{-2}$	0	$4.19 \cdot 10^{1}$	$1.91 \cdot 10^{1}$
Gynuella sunshinyii Δ*lcn*14-15, Δ*lcn*17-24	0	0	$1.66 \cdot 10^{-2}$	0

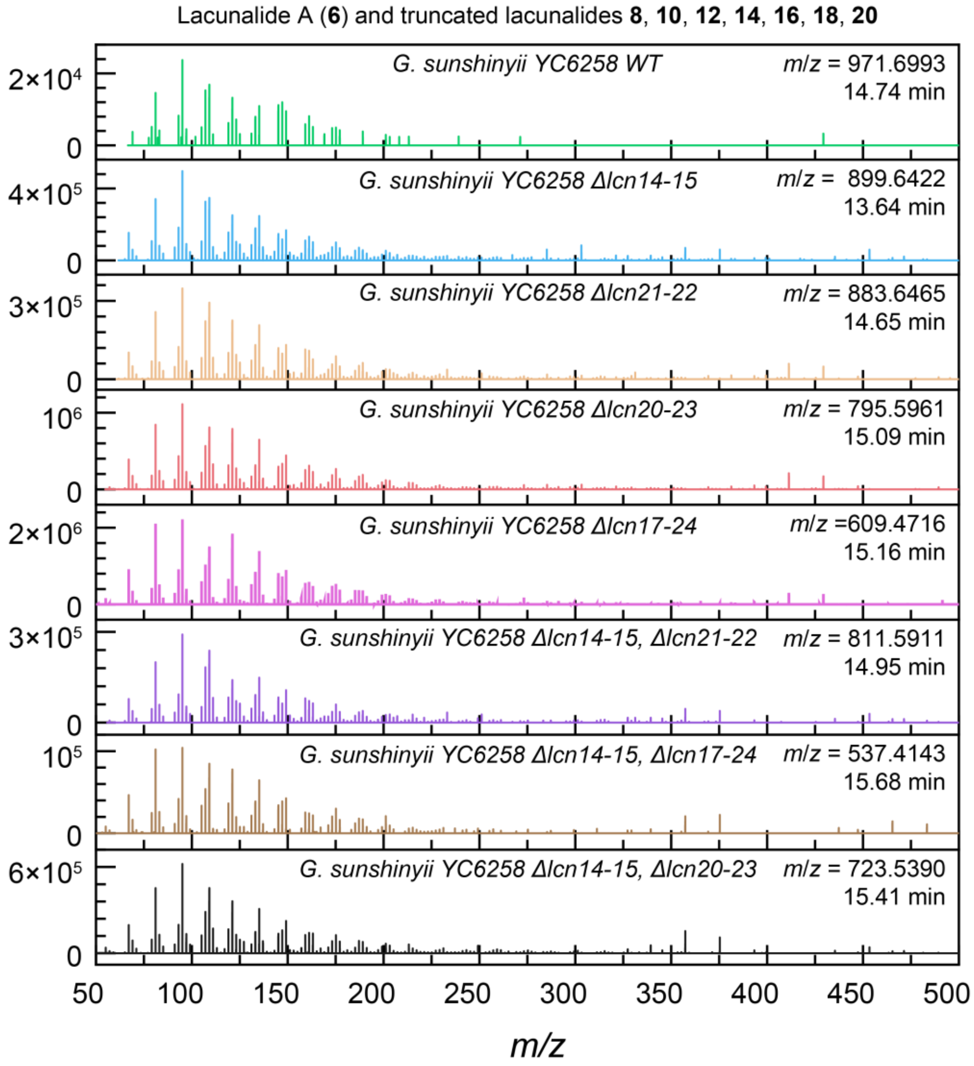

Figure S63 HPLC-HESI-HRMS/MS fragmentation patterns of lacunalide A (**6**) and truncated lacunalides (**8**, **10**, **12**, **14**, **16**, **18**, **20**) obtained during HPLC-MS measurements of extracts of expression cultures of the mutant indicated in the respective graphs. The retention time and experimental *m/z* value of the fragmented ion is indicated. The highly similar fragmentation patterns across a wide *m/z* ratio suggests chemical relatedness.

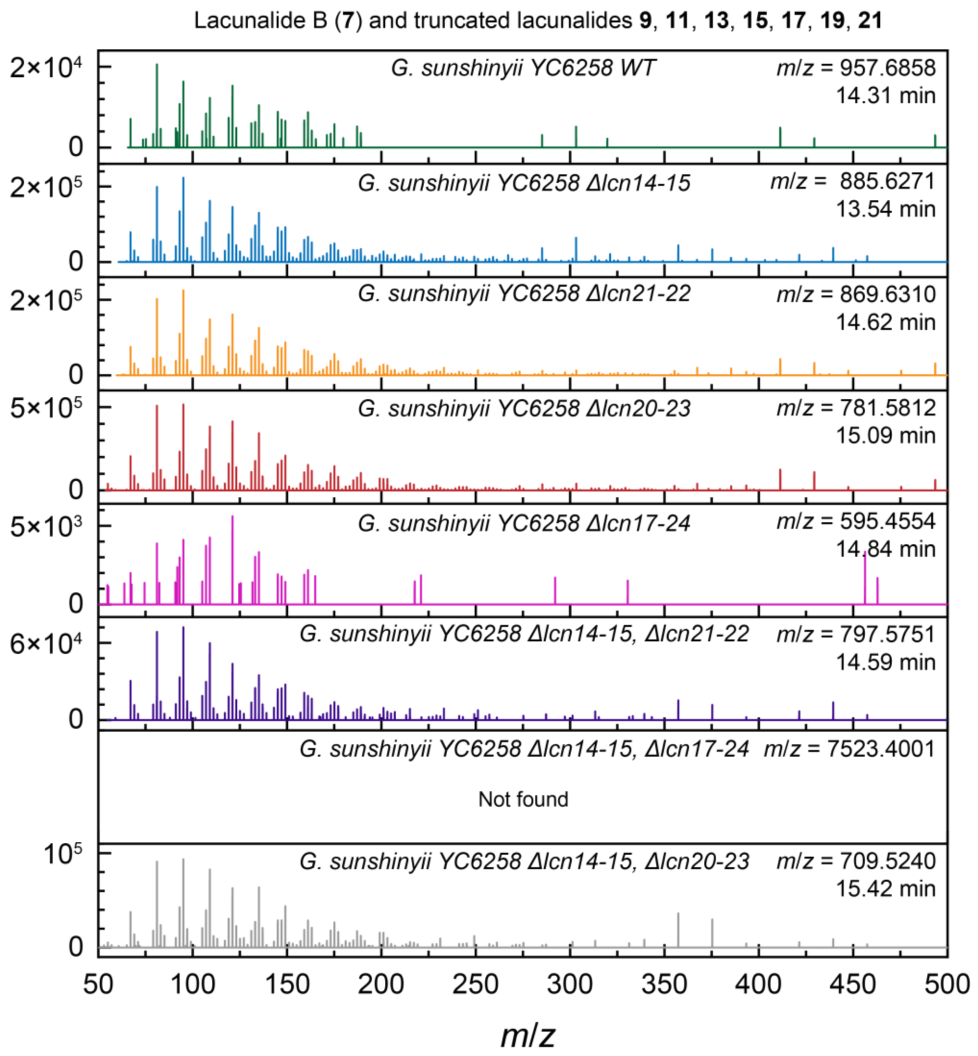

Figure S64 HPLC-HESI-HRMS/MS fragmentation patterns of lacunalide A (**7**) and truncated lacunalides (**9**, **11**, **13**, **15**, **17**, **19**, **21**) obtained during HPLC-MS measurements of extracts of expression cultures of the mutant indicated in the respective graphs. The retention time and experimental *m/z* value of the fragmented ion is indicated. The highly similar fragmentation patterns across a wide *m/z* ratio suggests chemical relatedness. For **19**, no peak was observed and hence no MS^2 spectrum is given.

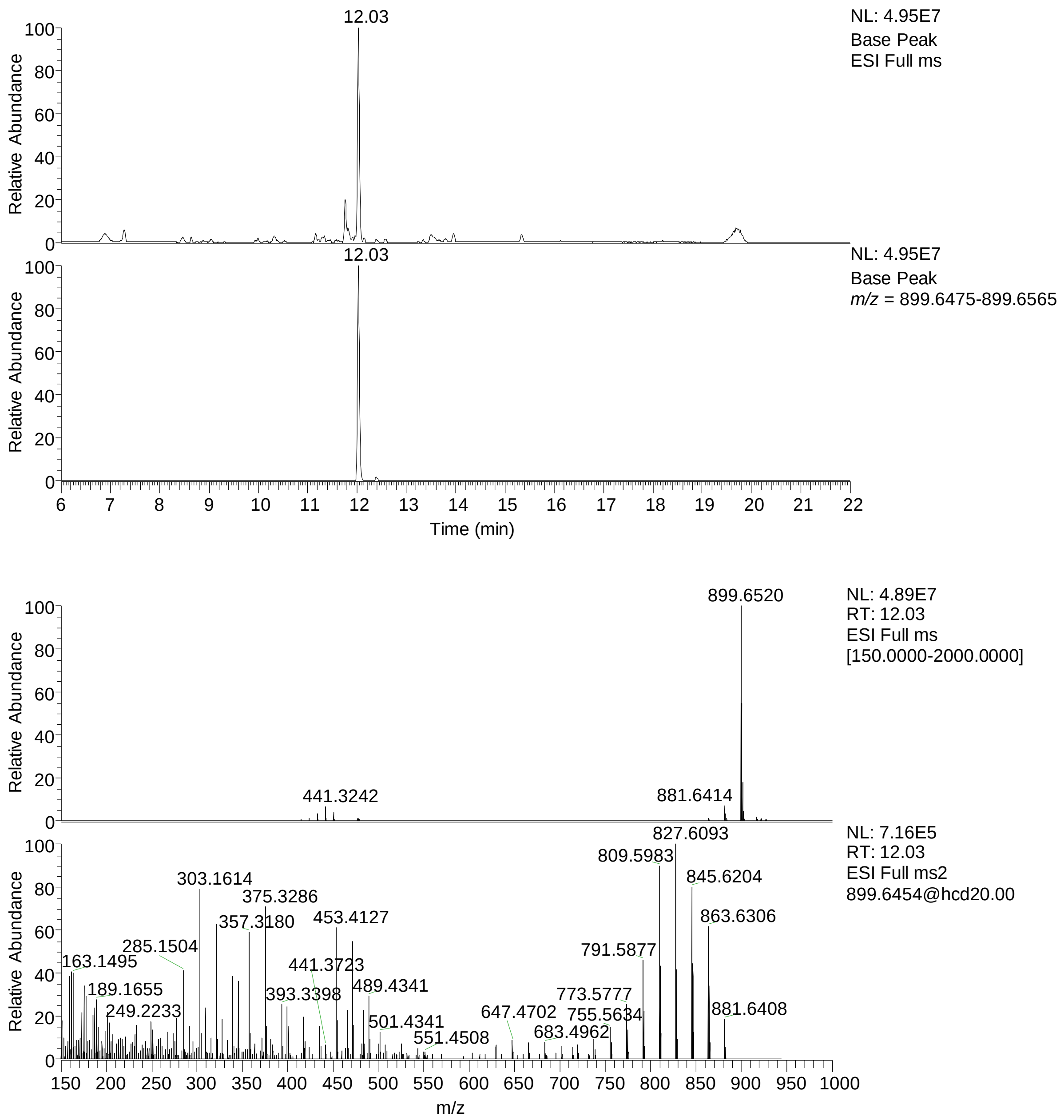

Figure S65 LC-MS analysis of purified lacunalide C (**8**). Top: Base peak full MS and extracted ion chromatogram (*m/z* 899.6520 ± 5 ppm) of purified natural product. Bottom: MS and MS/MS fragmentation spectra of **8**.

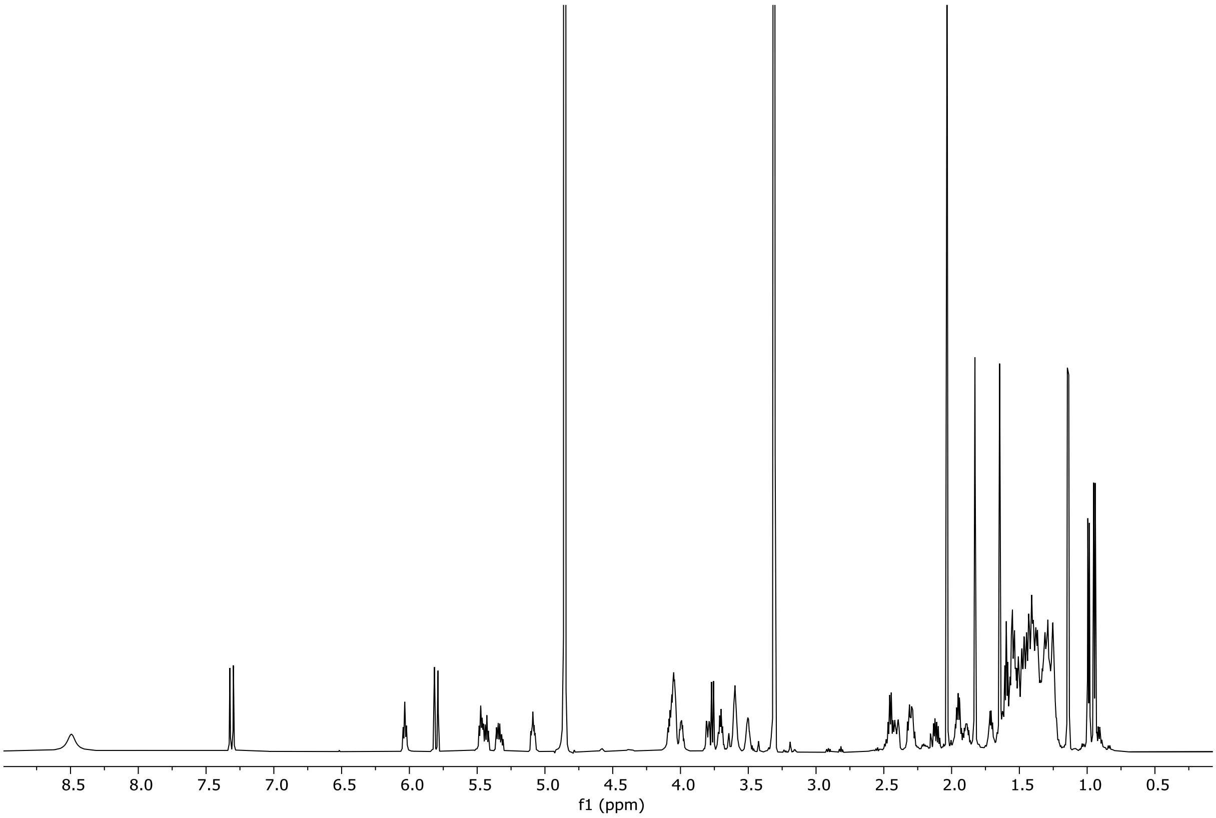

Figure S66 ^{1}H NMR spectrum of **8** in methanol-d_4 (^{1}H 600 MHz).

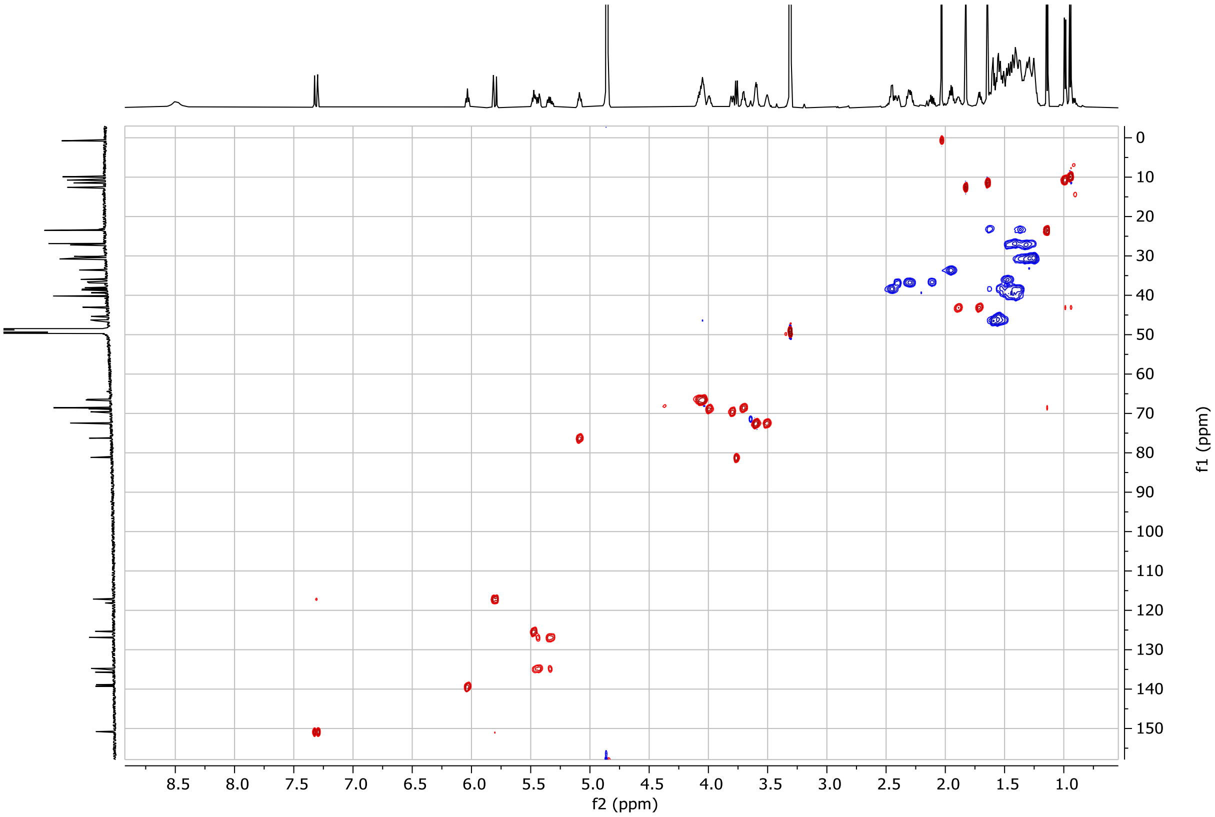

Figure S67 HSQC spectrum of **8** in methanol-d_4 (^{1}H 600 MHz).

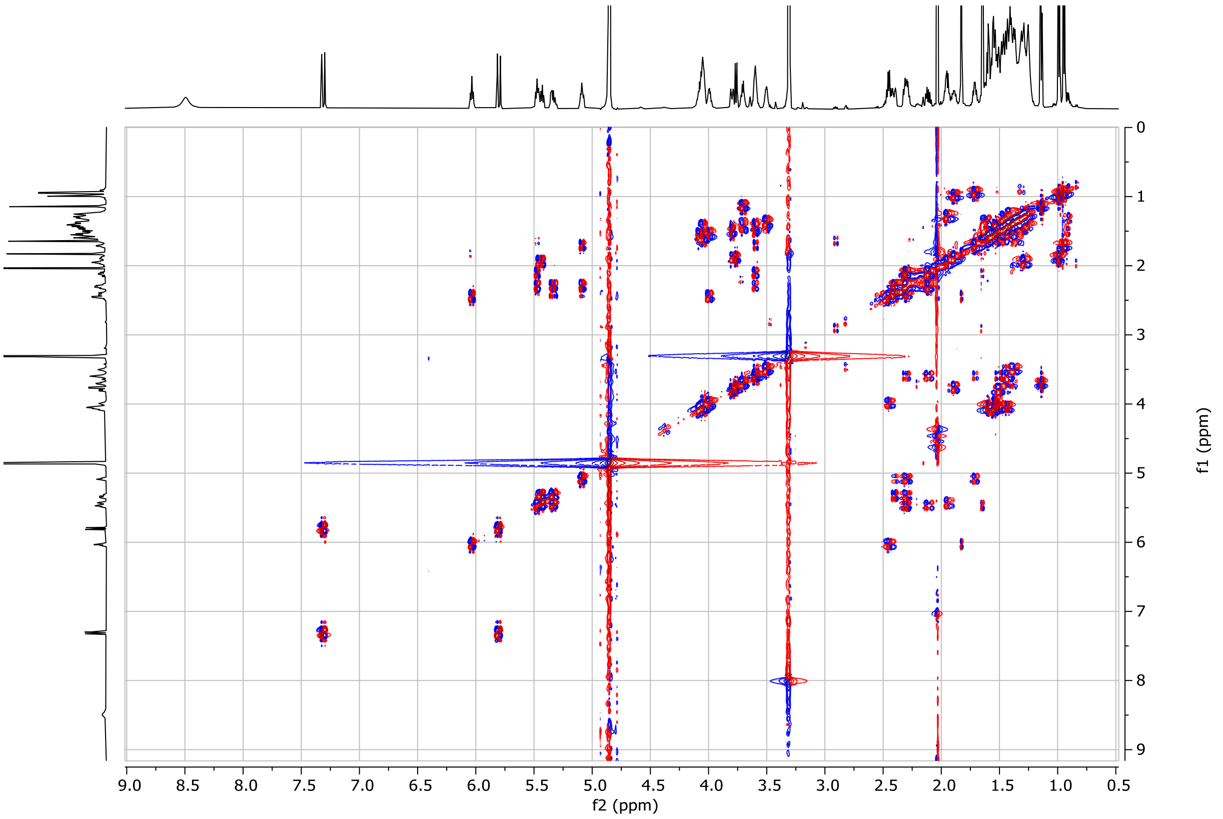

Figure S68 COSY spectrum of **8** in methanol-d_4 (^{1}H 600 MHz, ^{13}C 151 MHz).

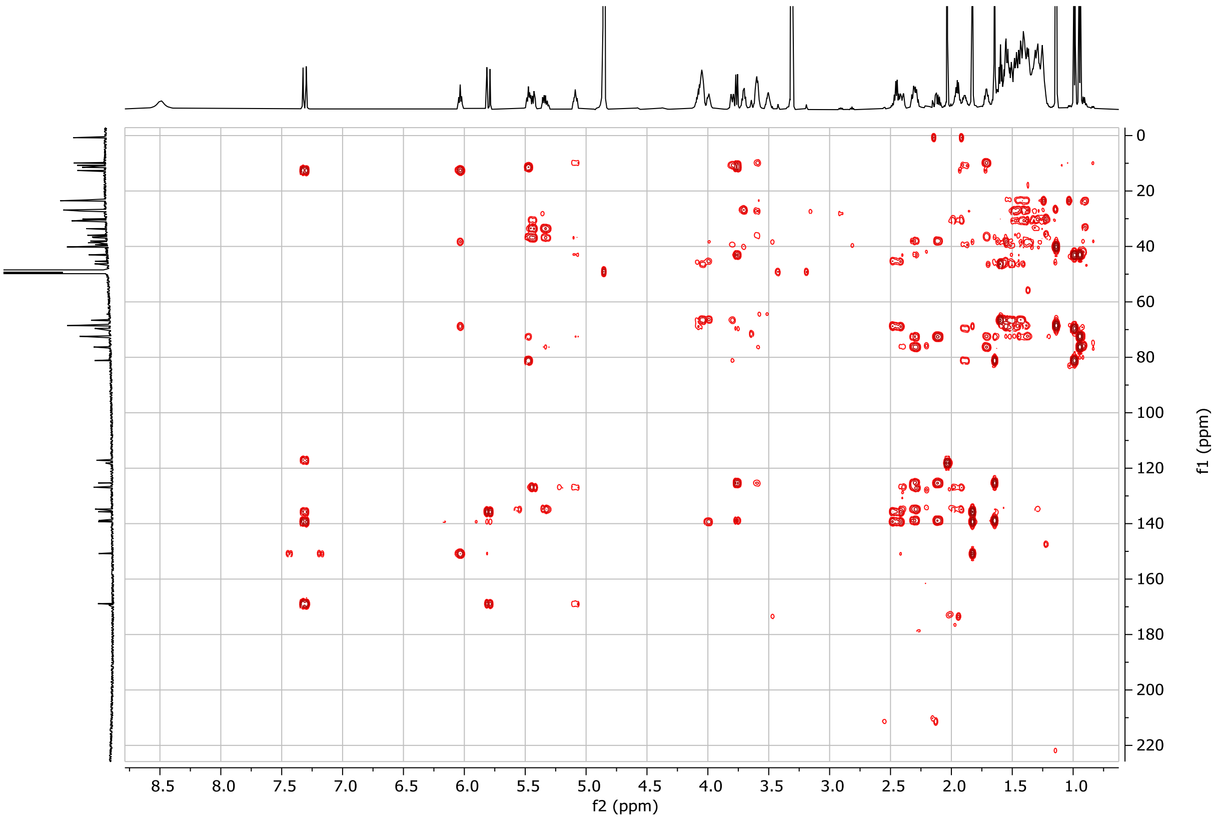

Figure S69 HMBC spectrum of **8** in methanol-d_4 (^{1}H 600 MHz, ^{13}C 151 MHz).

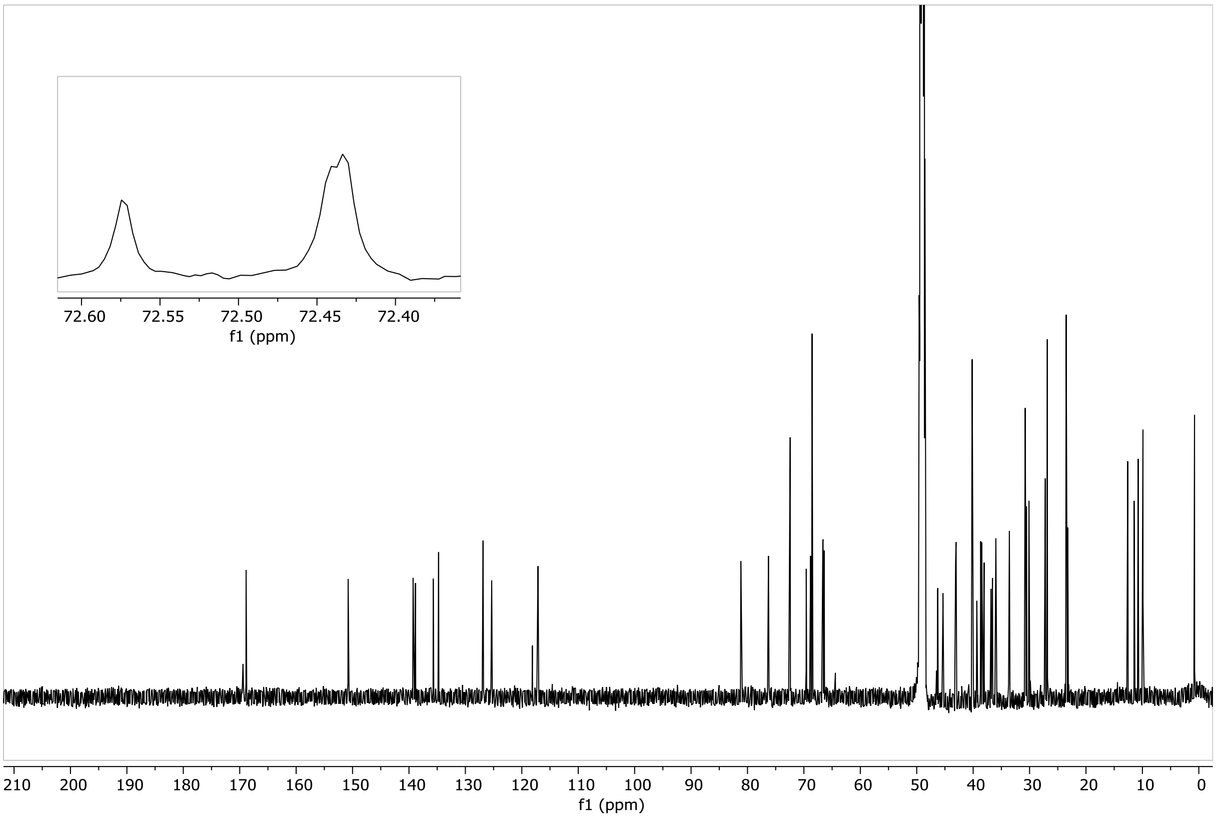

Figure S70 ^{13}C NMR spectrum of **8** in methanol-d_4 (^{13}C 151 MHz).

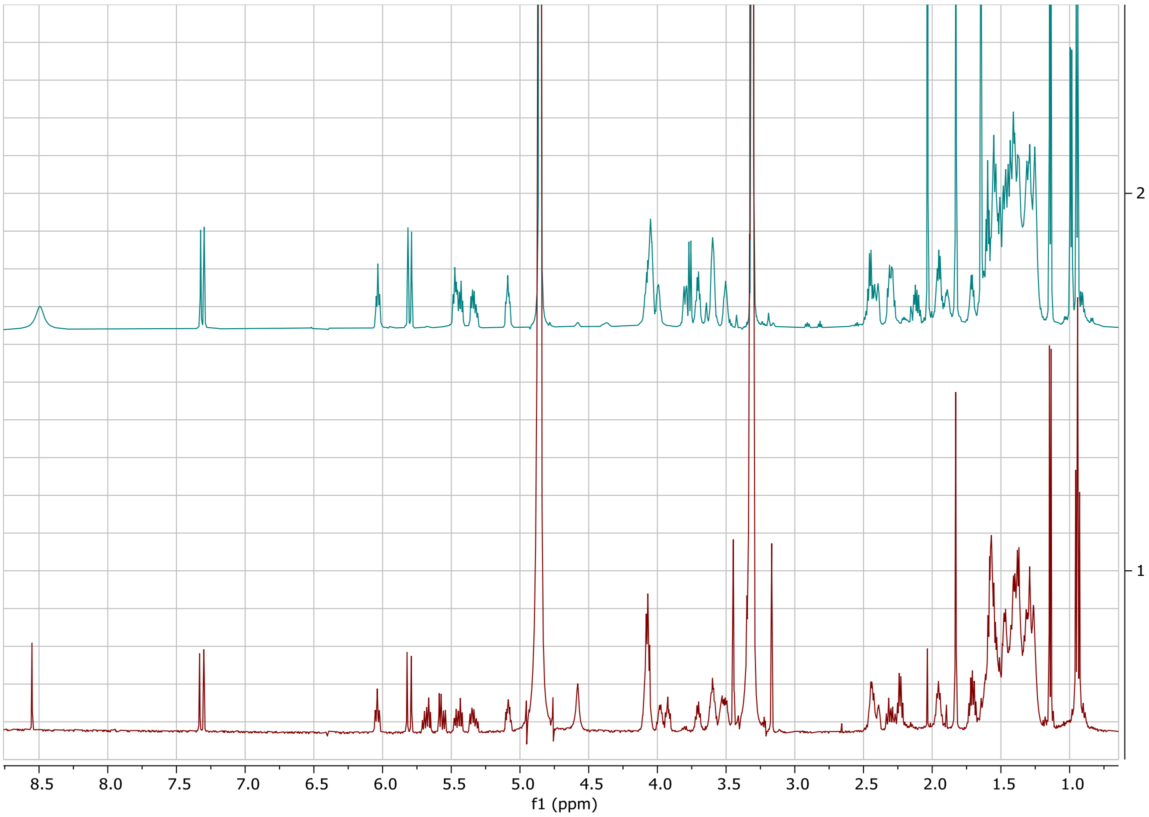

Figure S71 Overlay of ^{1}H spectra of **7** (bottom, (*60*)) and **8** (top).

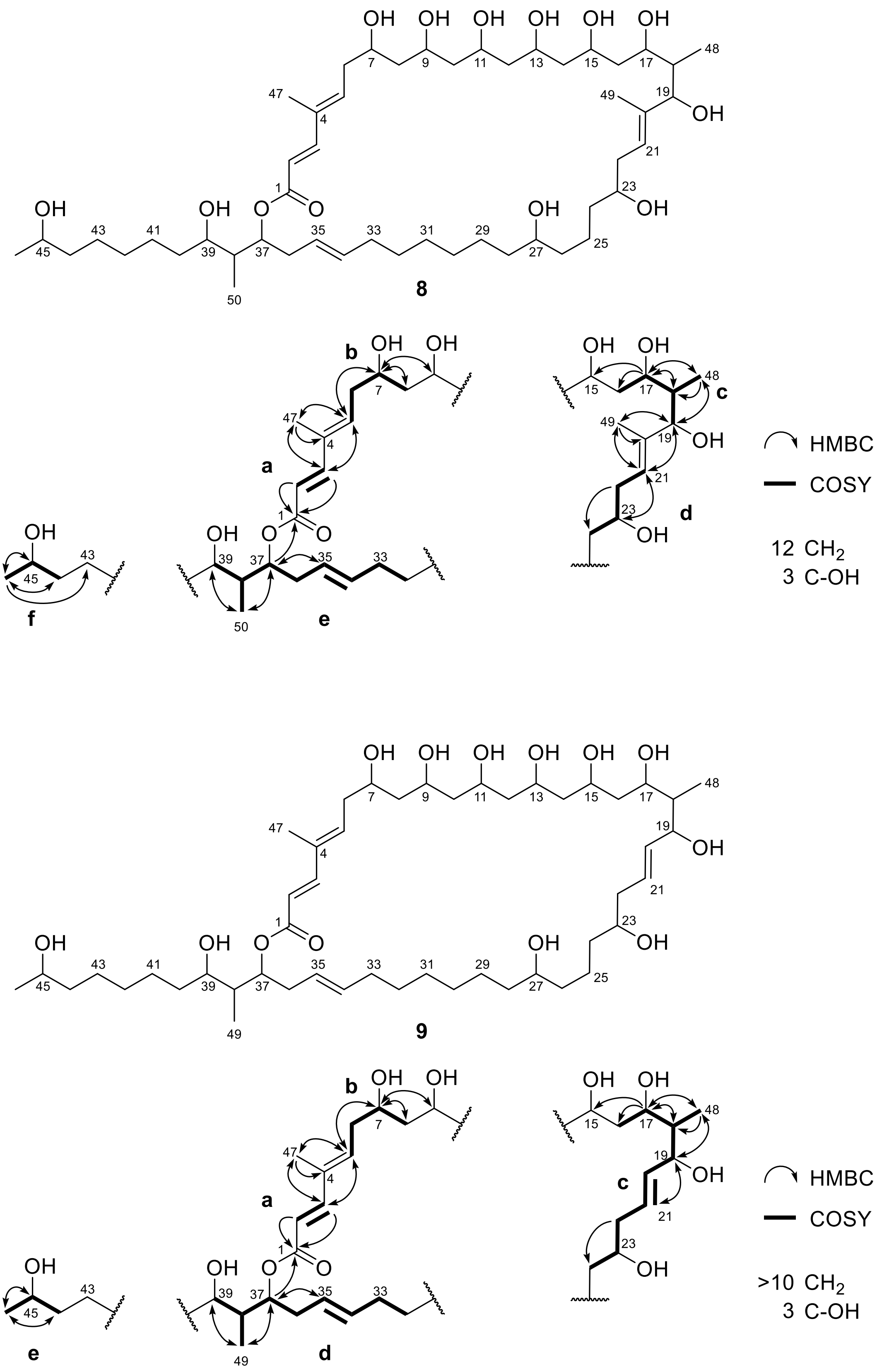

Figure S72 Structural assignment of **8** and **9**. Due to overlapping signals not all carbons could be structurally assigned.

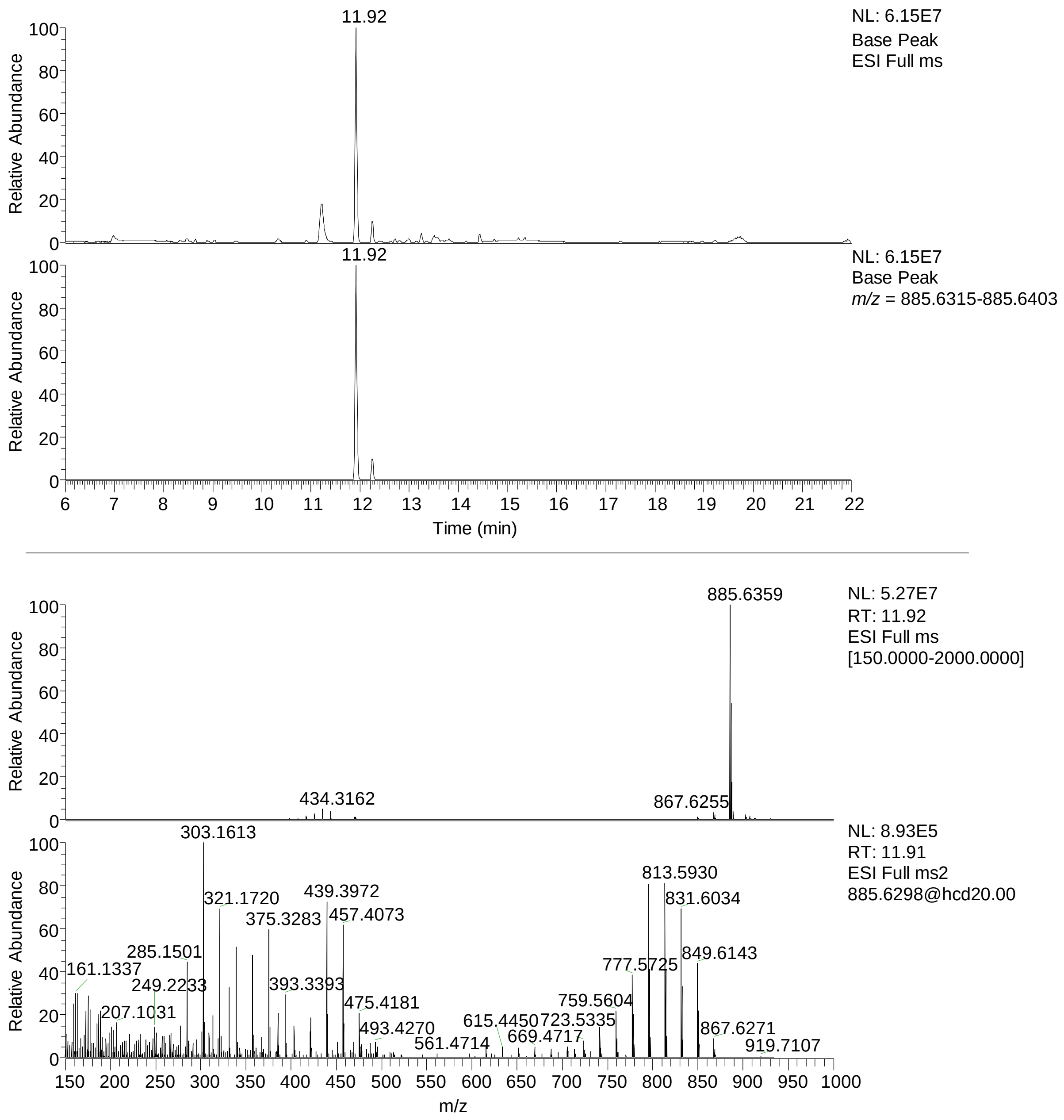

Figure S73 LC-MS analysis of purified **9**. Top: Base peak full MS and extracted ion chromatogram (*m/z* 885.6359 ± 5 ppm) of purified natural product. Bottom: MS and MS/MS fragmentation spectra of **9**.

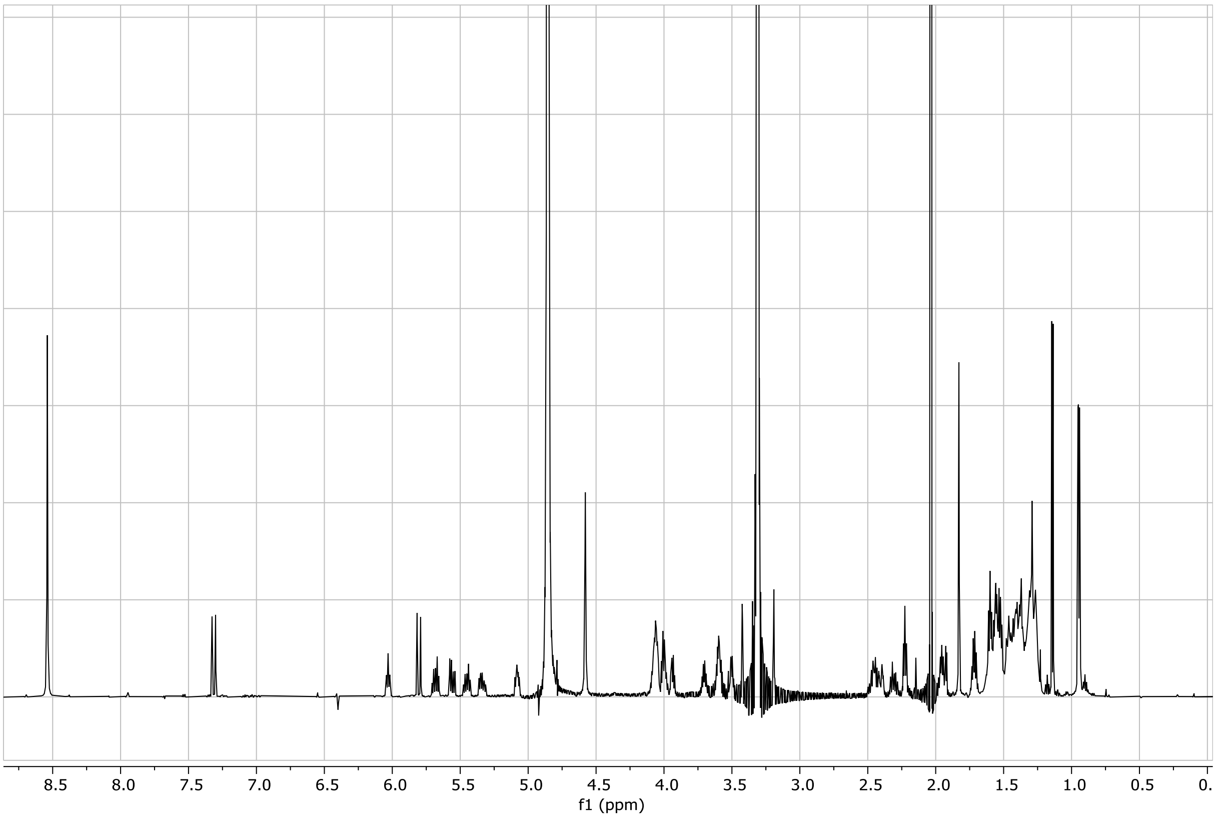

Figure S74 ^{1}H NMR spectrum of **9** in methanol-d_4 (^{1}H 600 MHz).

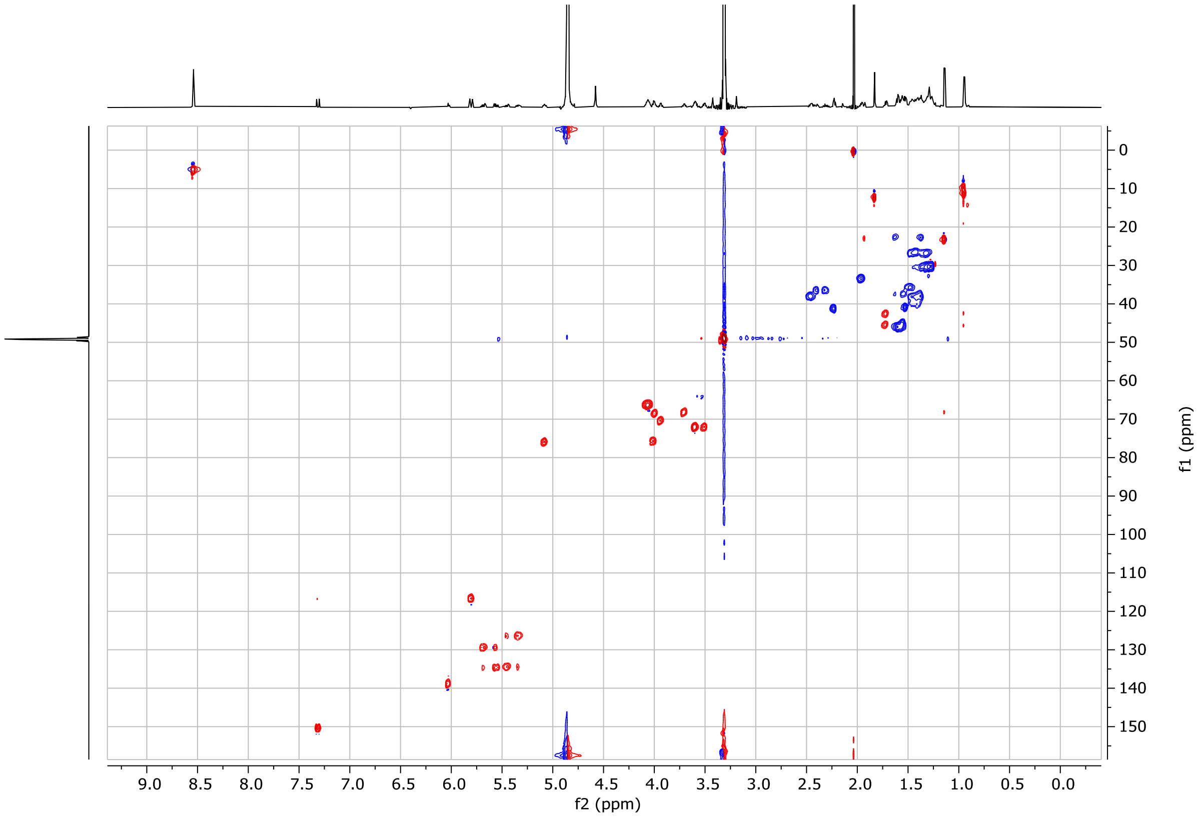

Figure S75 HSQC spectrum of **9** in methanol-d_4 (^{1}H 600 MHz).

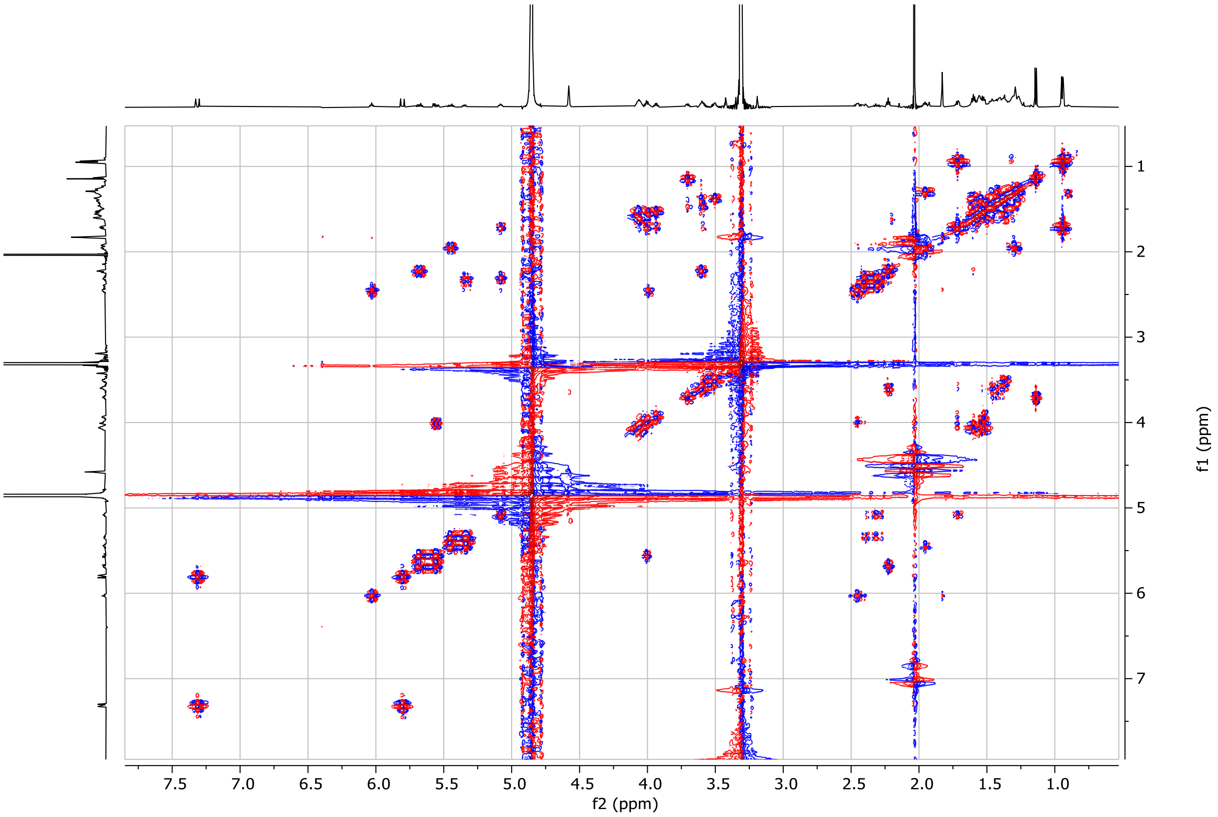

Figure S76 COSY spectrum of **9** in methanol-*d*₄ (^{1}H 600 MHz, ^{13}C 151 MHz).

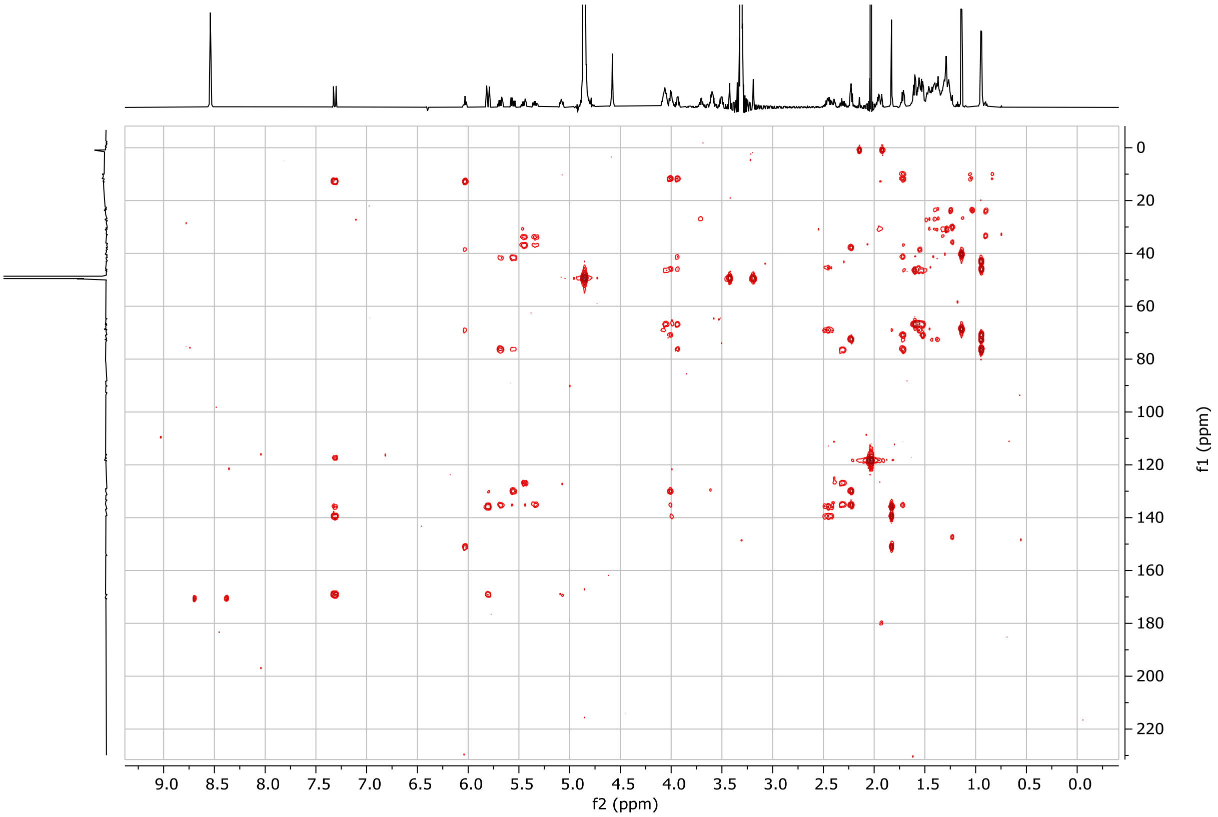

Figure S77 HMBC spectrum of **9** in methanol-*d*₄ (^{1}H 600 MHz, ^{13}C 151 MHz).

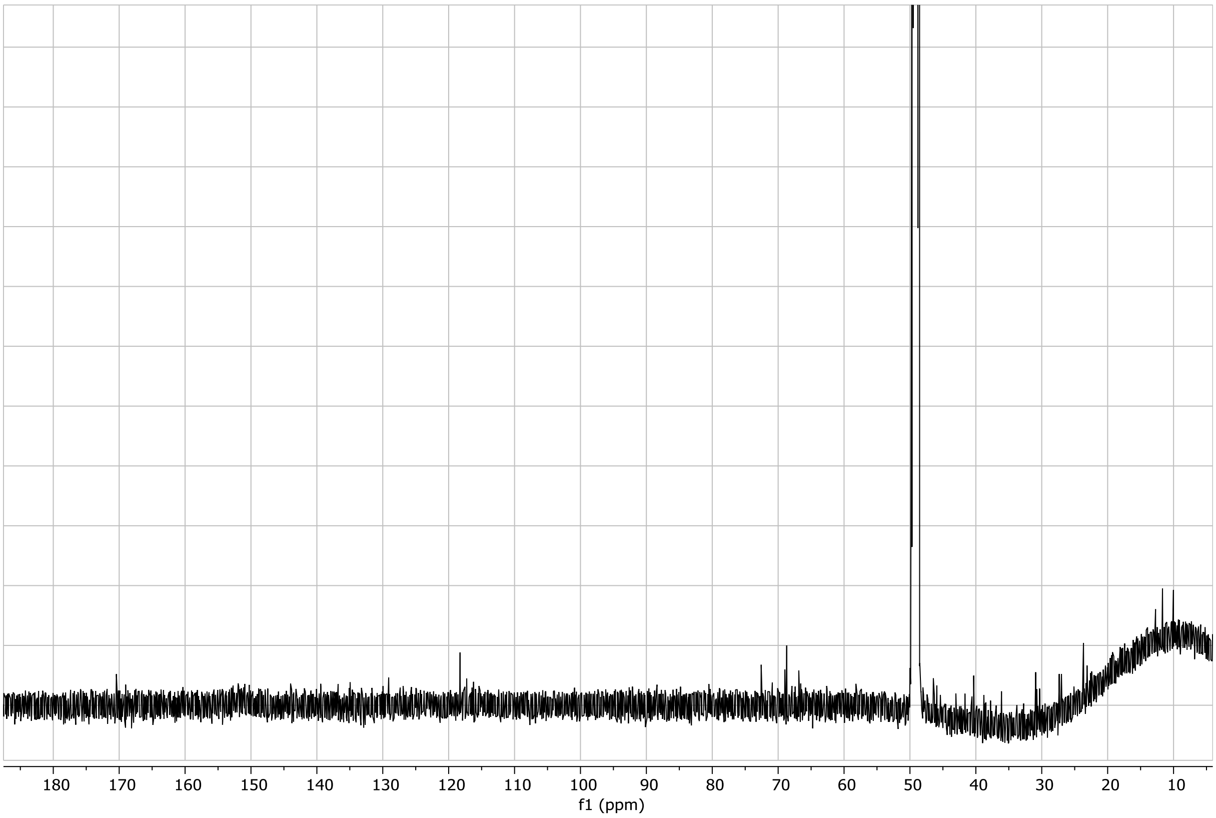

Figure S78 ^{13}C NMR spectrum of **9** in methanol-d_4 (^{13}C 151 MHz).

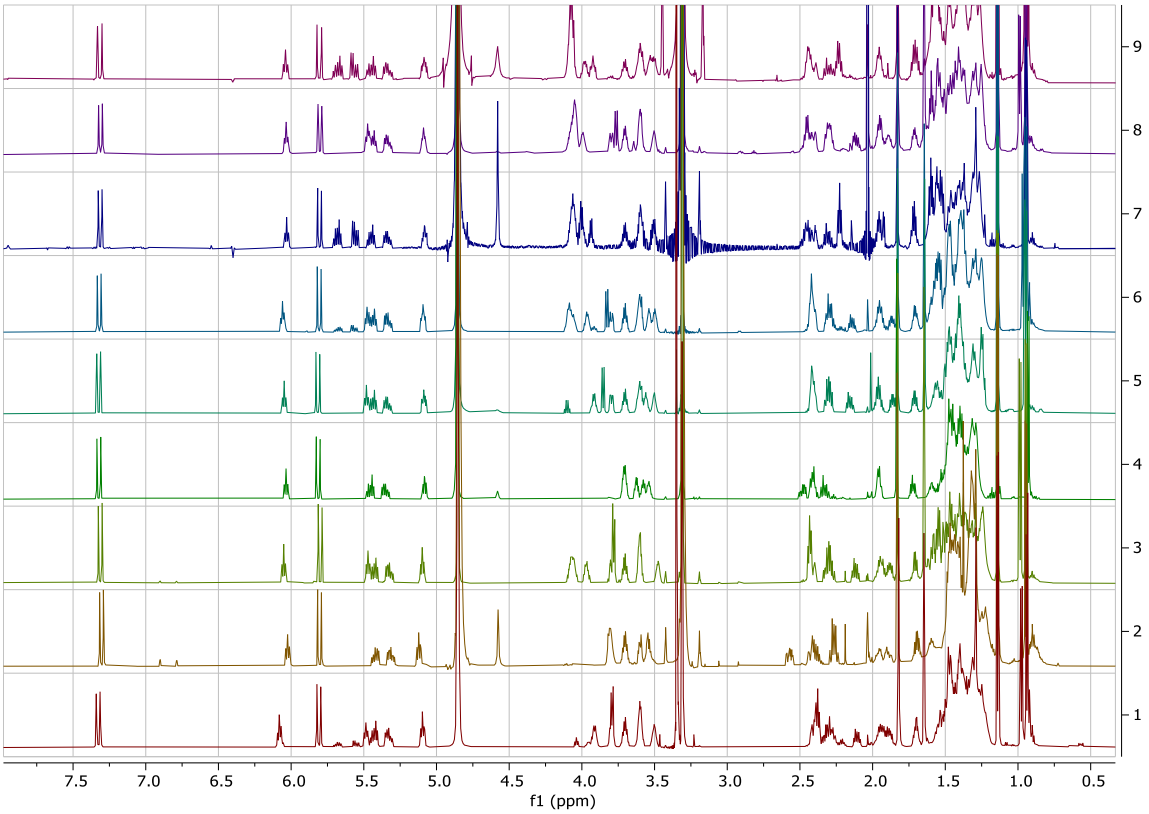

Figure S79 Comparison of ^{1}H spectra of **6** with isolated compounds **8**, **9**, **10**+**11**, **12**, **14**, **16**, **18**, **20**+**21** (top to bottom). The number of methanetriyl groups connected to oxygens in the region of 3.5 to 4.1 ppm differs based on how much of the lacunalide PKS is deleted (Figure 5).

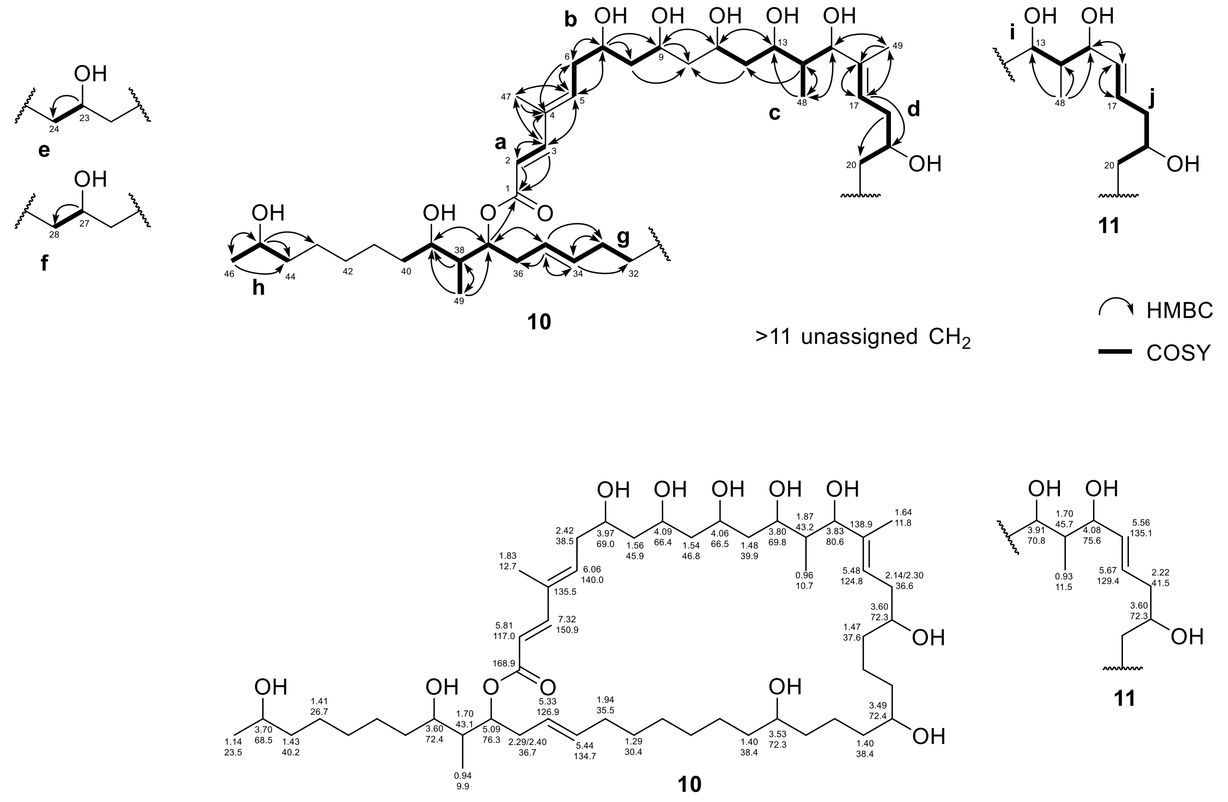

Figure S80 Structural assignment of **10**+**11**. Due to overlapping signals not all carbons could be structurally assigned.

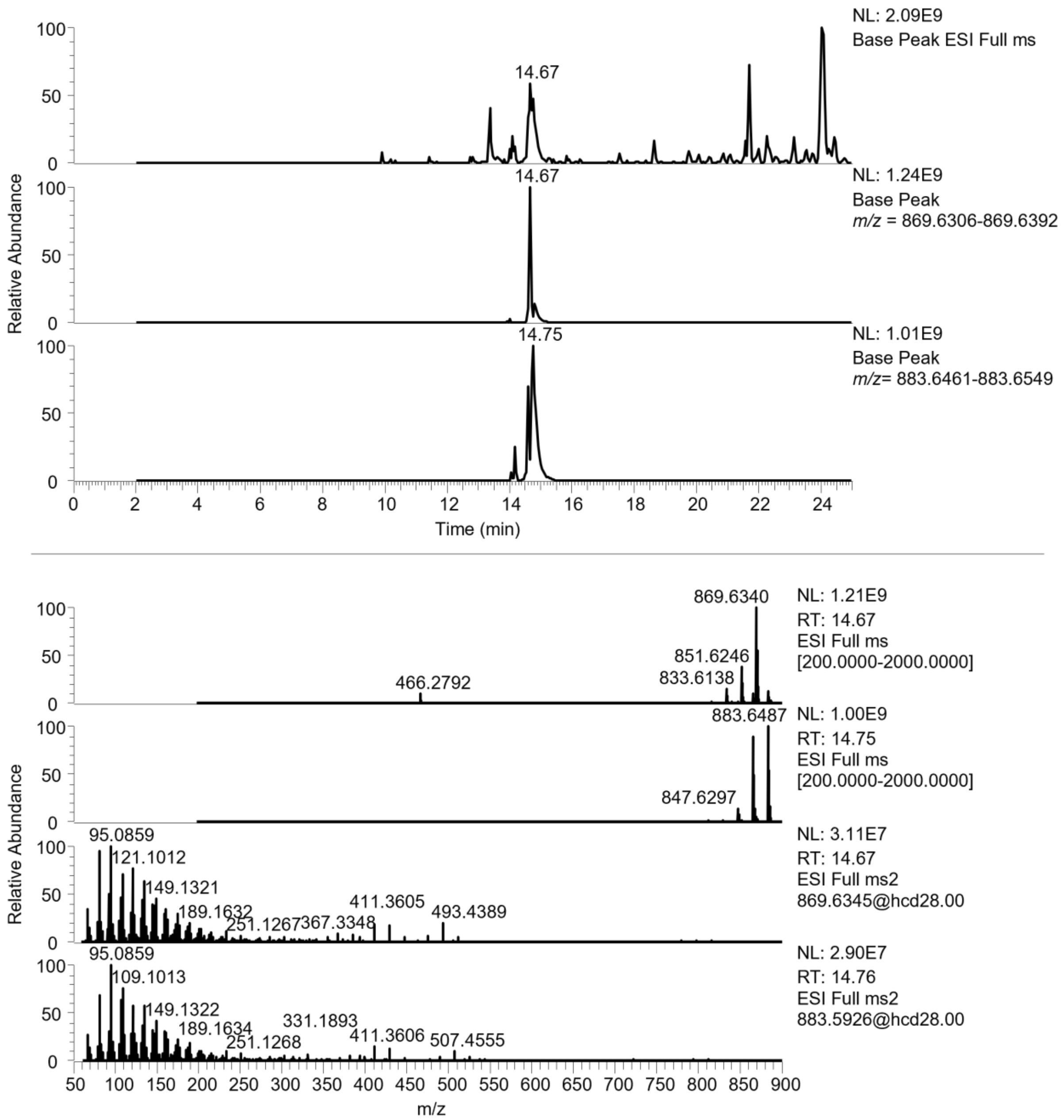

Figure S81 LC-MS analysis of purified **10**+**11**. Top: Base peak full MS and extracted ion chromatogram (*m/z* 883.6505 and 869.6349 ± 5 ppm) of purified natural product. Bottom: MS and MS/MS fragmentation spectra of **10**+**11**.

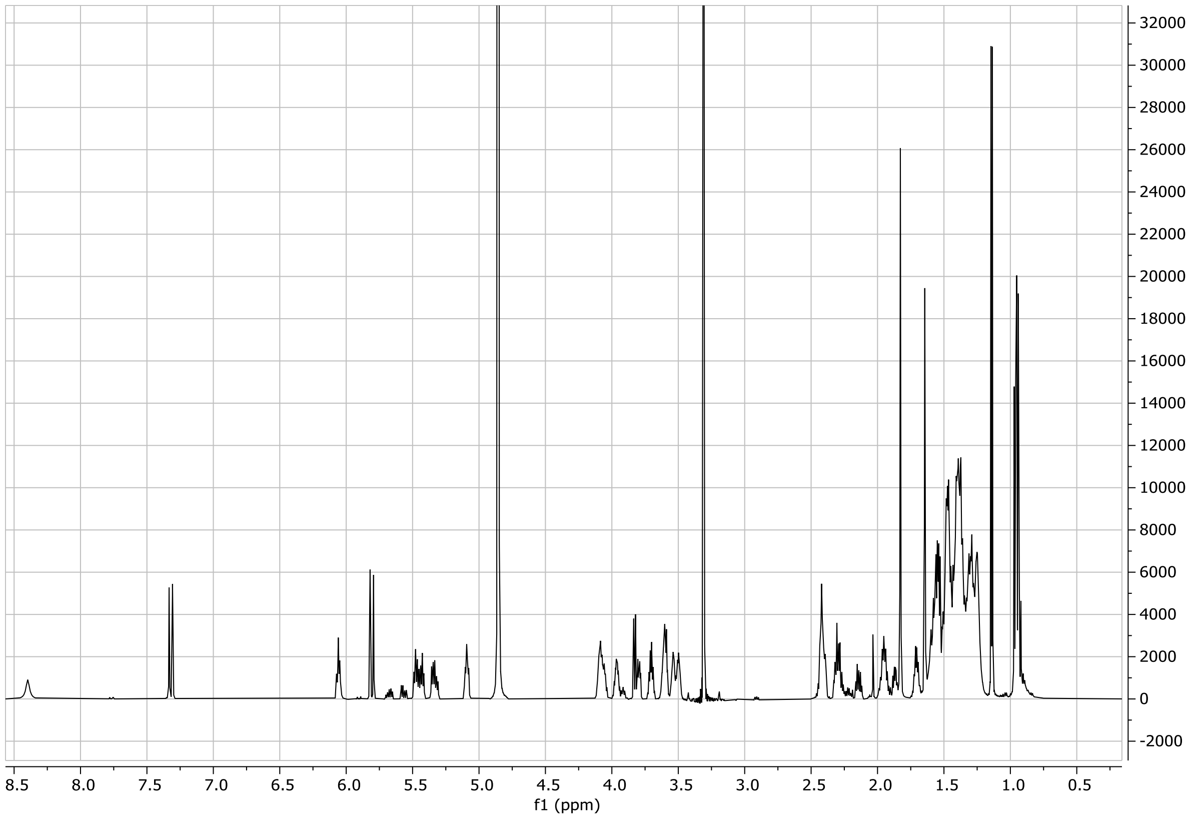

Figure S82 ^{1}H NMR spectrum of **10**+**11** in methanol-d_4 (^{1}H 600 MHz).

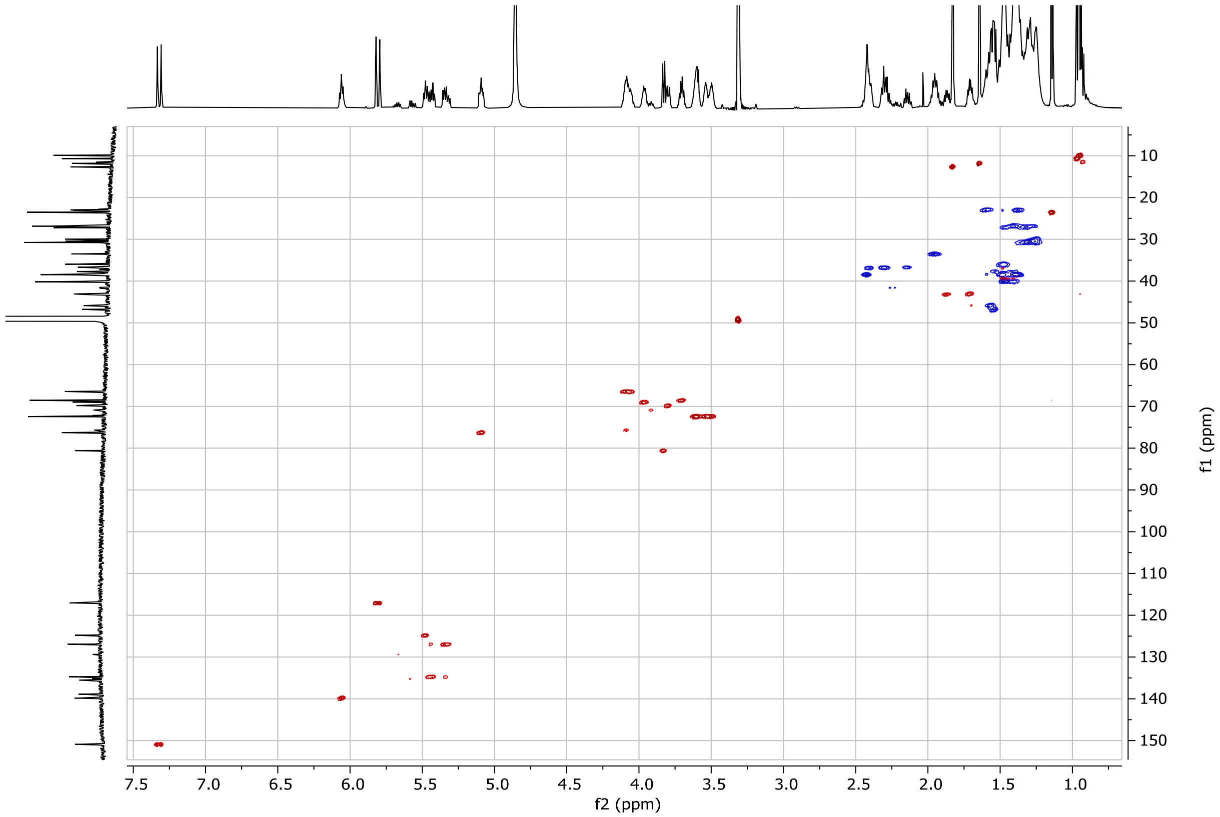

Figure S83 HSQC spectrum of **10**+**11** in methanol-d_4 (^{1}H 600 MHz).

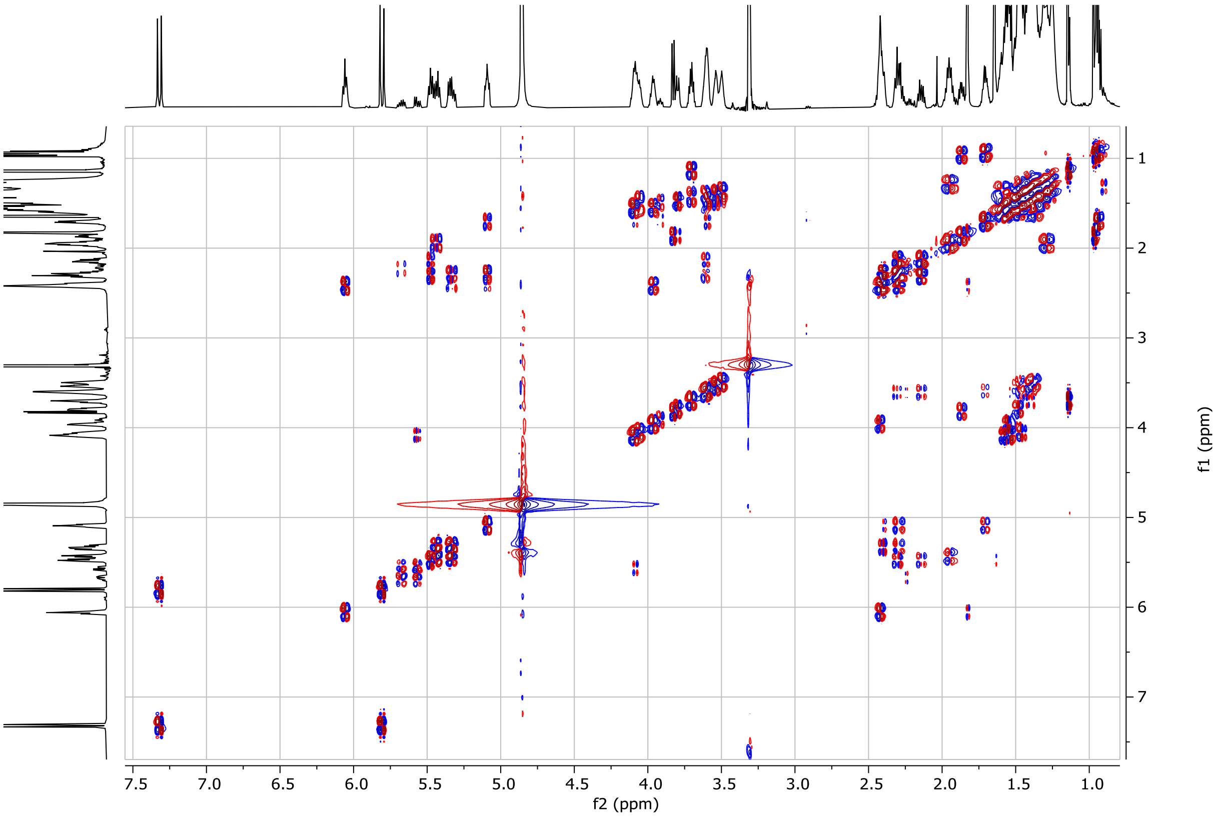

Figure S84 COSY spectrum of **10**+**11** in methanol-d_4 (^{1}H 600 MHz, ^{13}C 151 MHz).

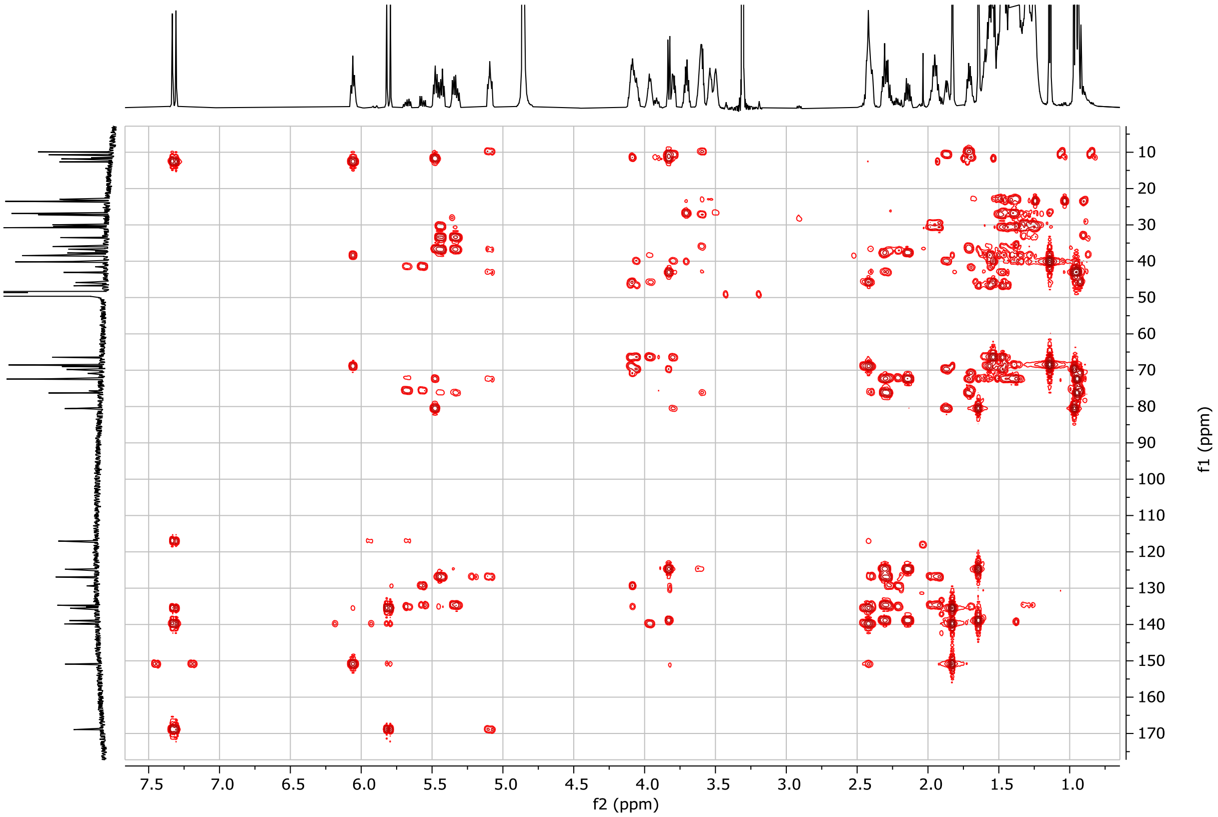

Figure S85 HMBC spectrum of **10**+**11** in methanol-d_4 (^{1}H 600 MHz, ^{13}C 151 MHz).

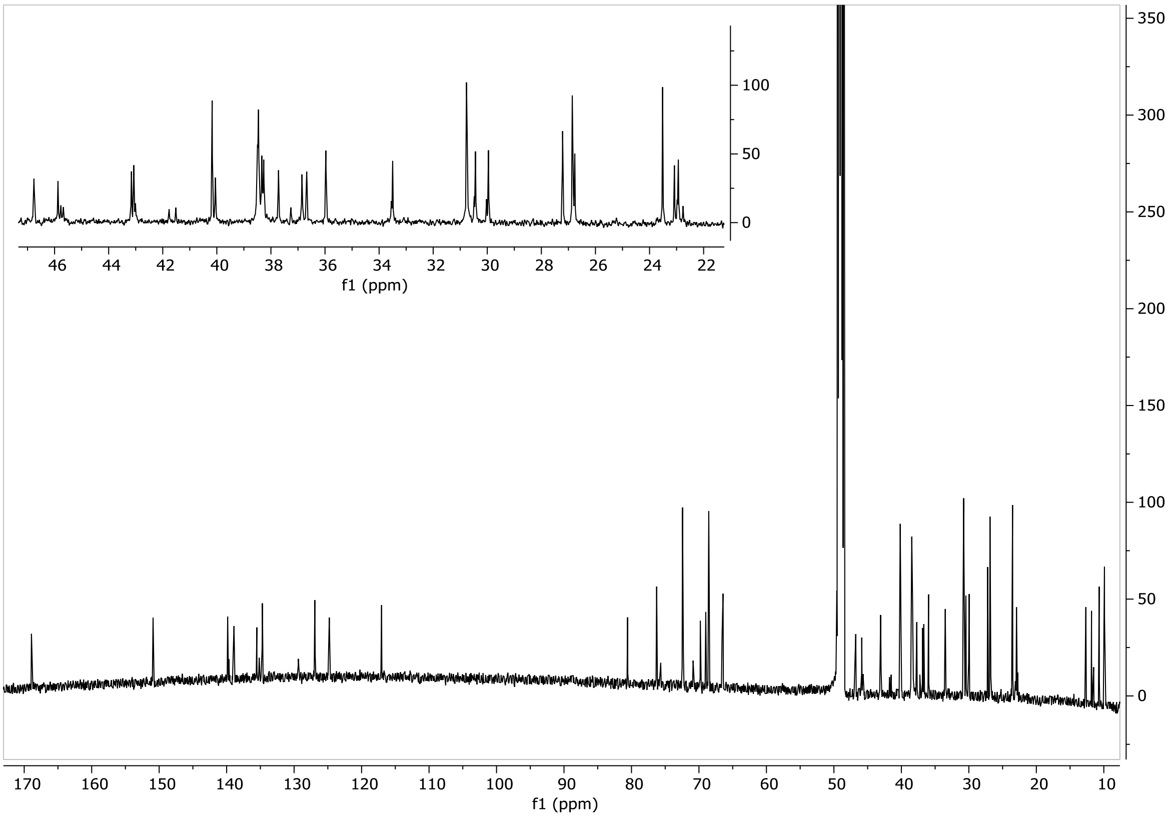

Figure S86 ^{13}C NMR spectrum of **10**+**11** in methanol-d_4 (^{13}C 151 MHz).

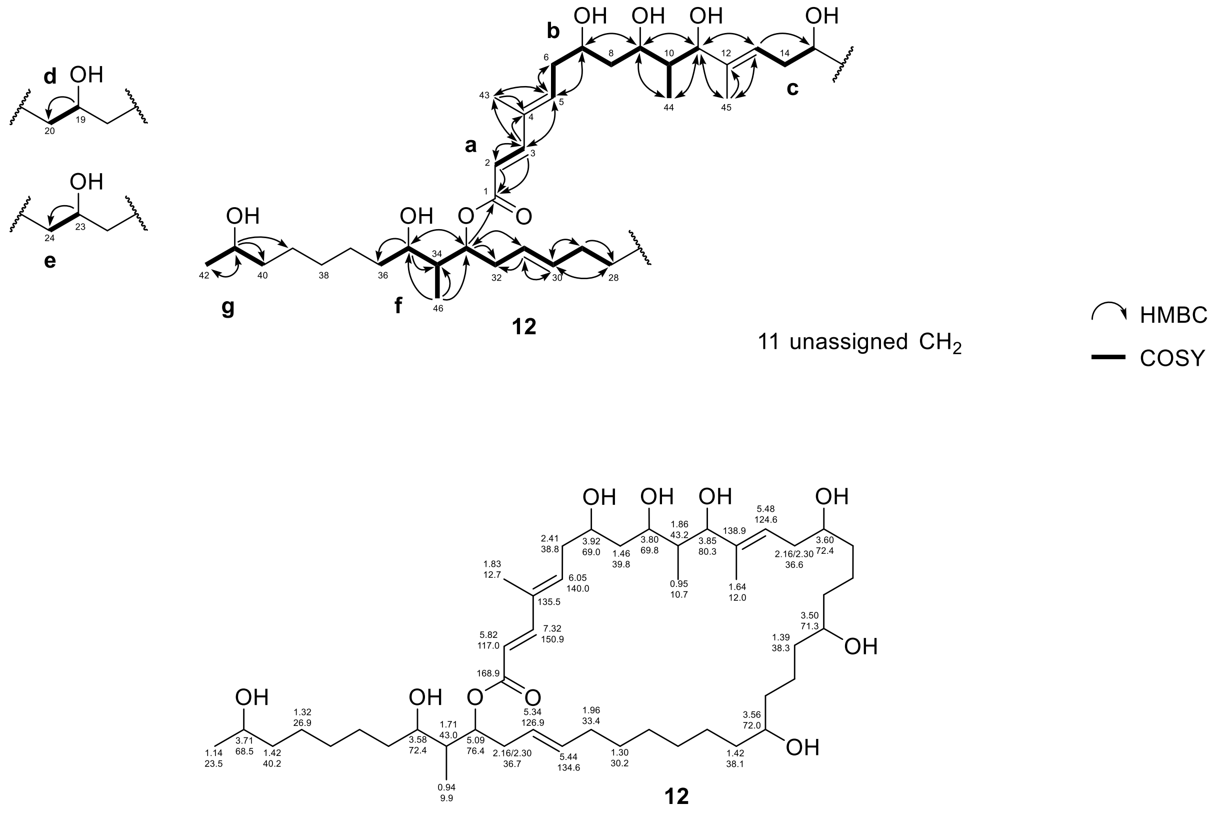

Figure S87 Structural assignment of **12**. Due to overlapping signals not all carbons could be structurally assigned.

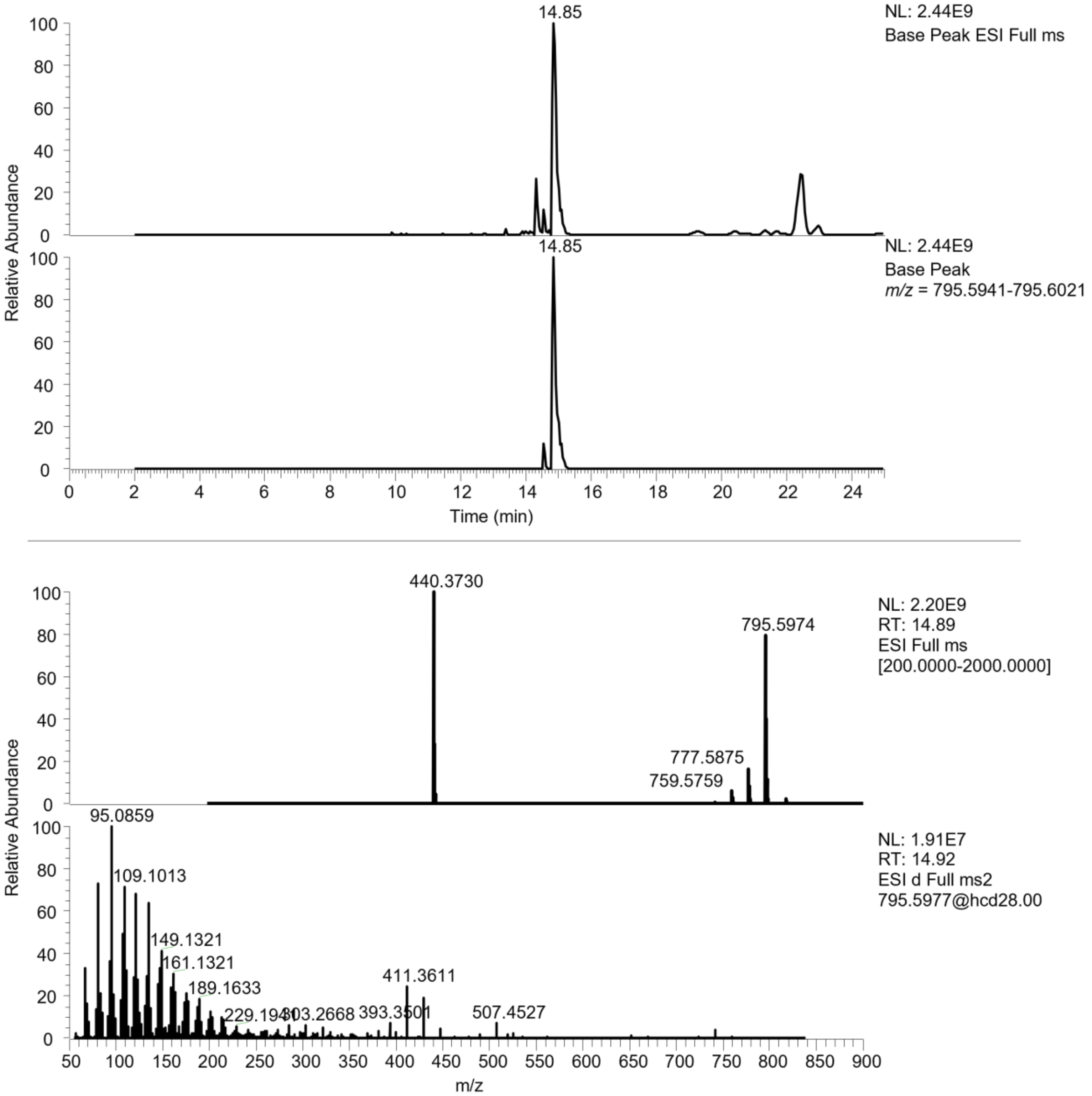

Figure S88 LC-MS analysis of purified **12**. Top: Base peak full MS and extracted ion chromatogram (*m/z* 795.5981 ± 5 ppm) of purified natural product. Bottom: MS and MS/MS fragmentation spectra of **12**.

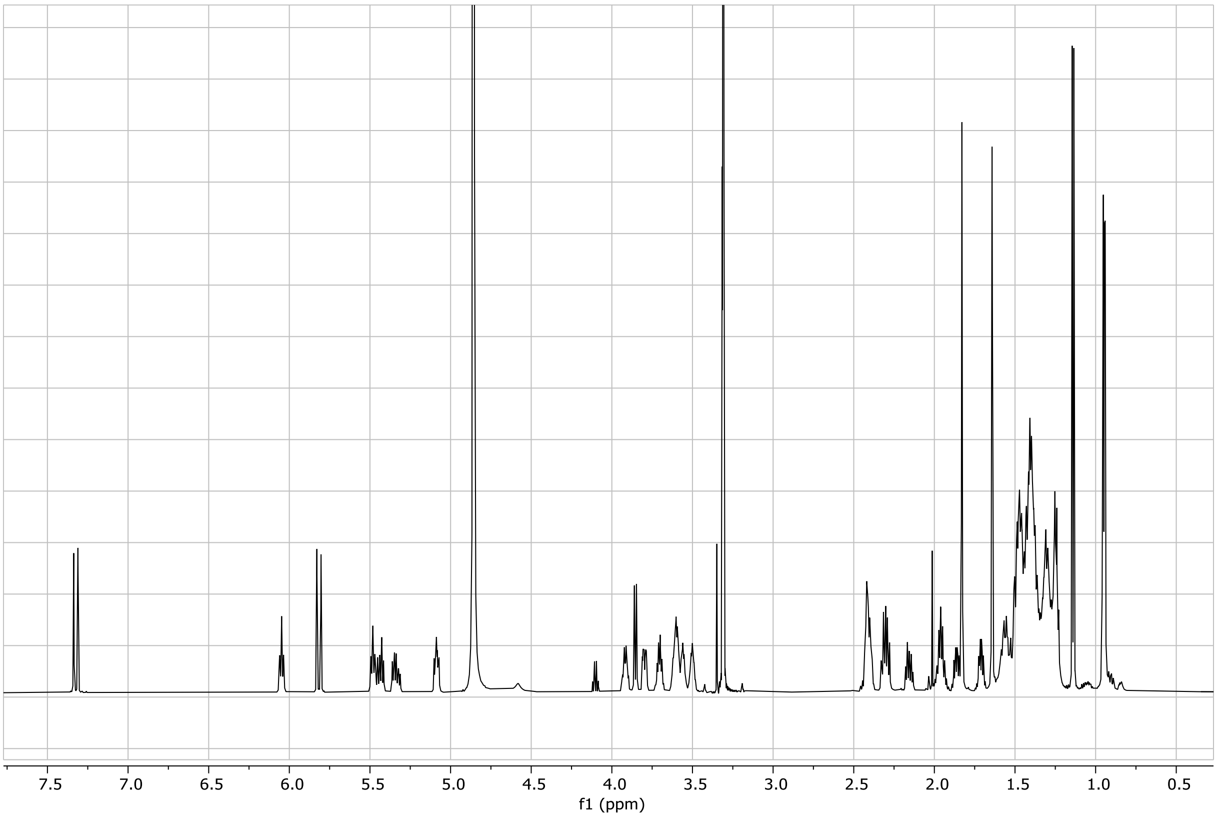

Figure S89 ^{1}H NMR spectrum of **12** in methanol-d_4 (^{1}H 600 MHz).

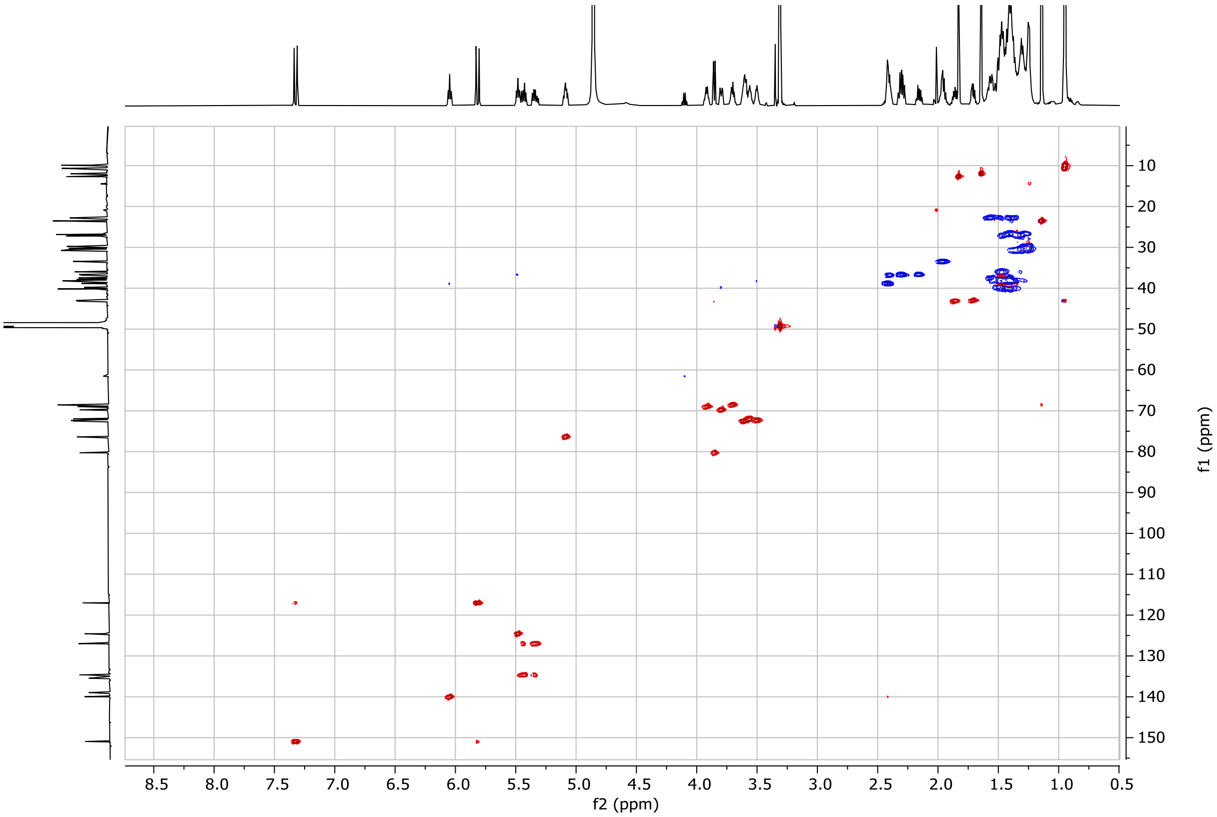

Figure S90 HSQC spectrum of **12** in methanol-d_4 (^{1}H 600 MHz).

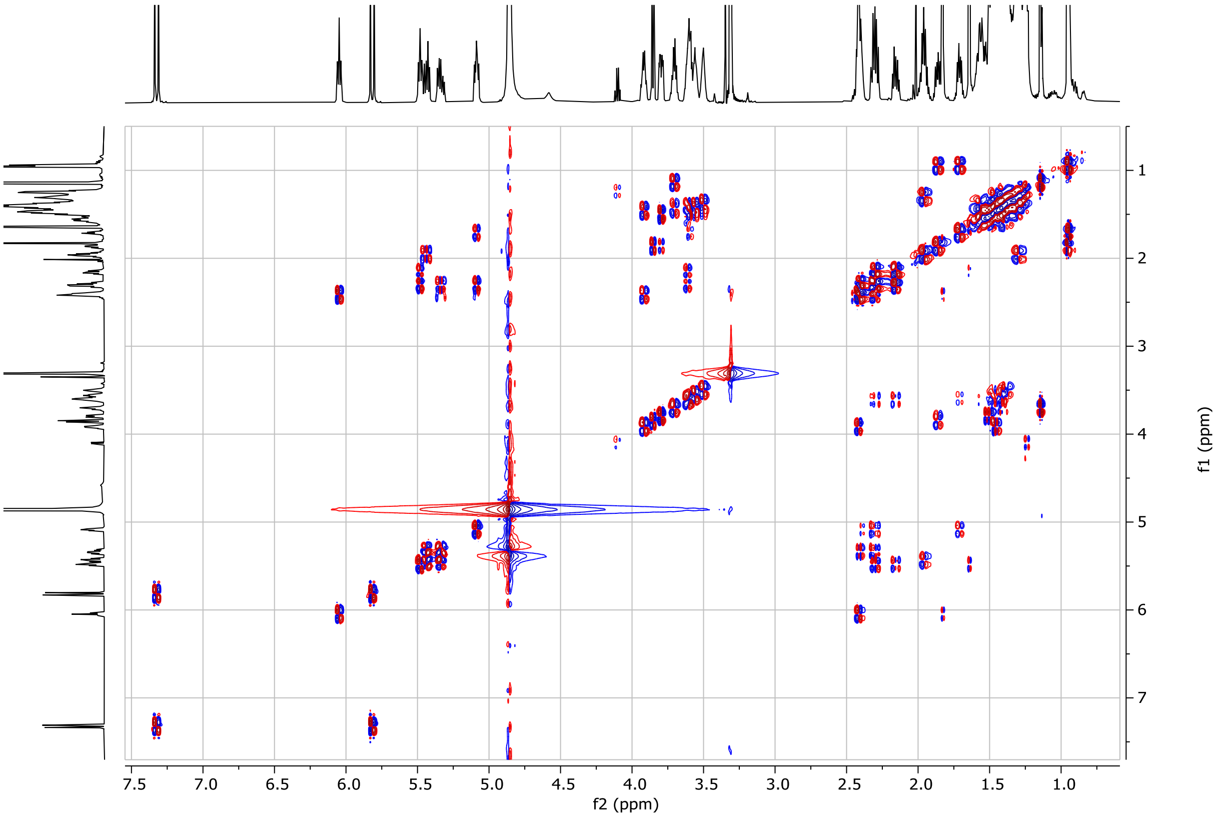

Figure S91 COSY spectrum of **12** in methanol-d_4 (^{1}H 600 MHz, ^{13}C 151 MHz).

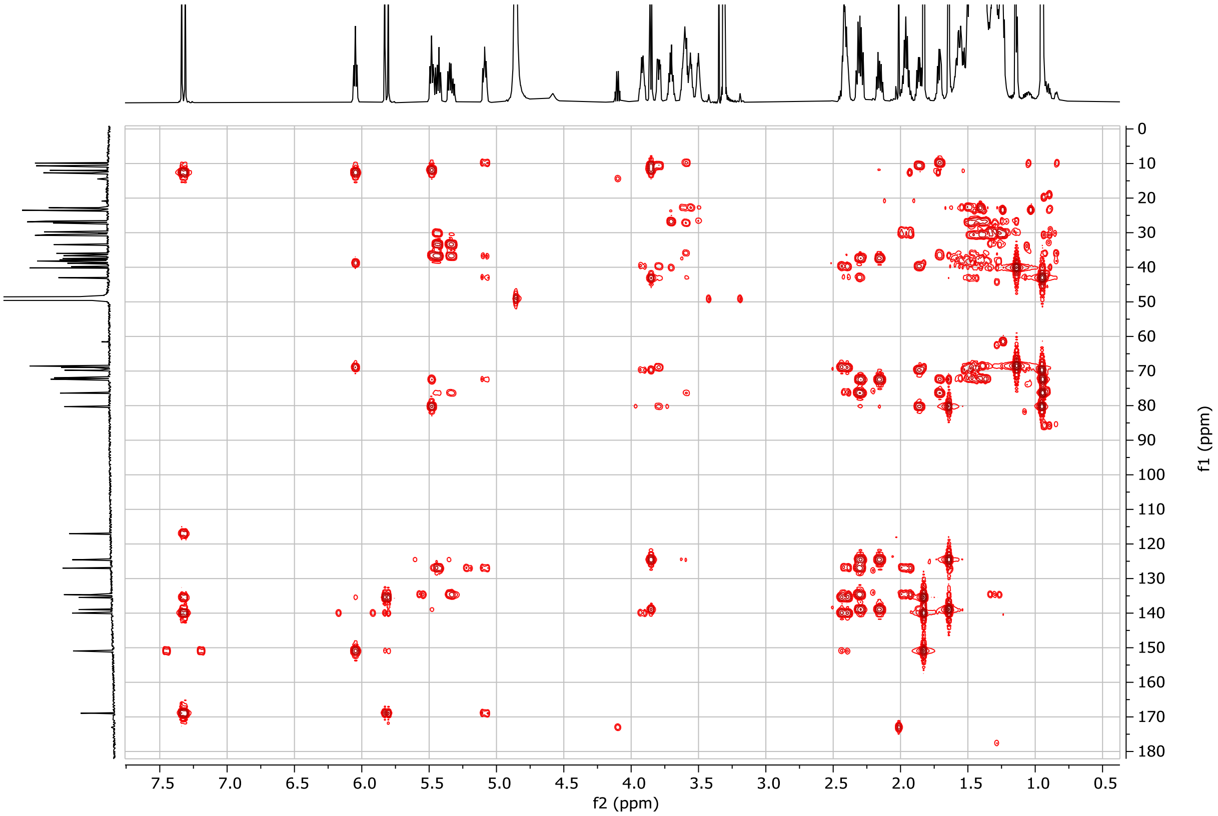

Figure S92 HMBC spectrum of **12** in methanol-d_4 (^{1}H 600 MHz, ^{13}C 151 MHz).

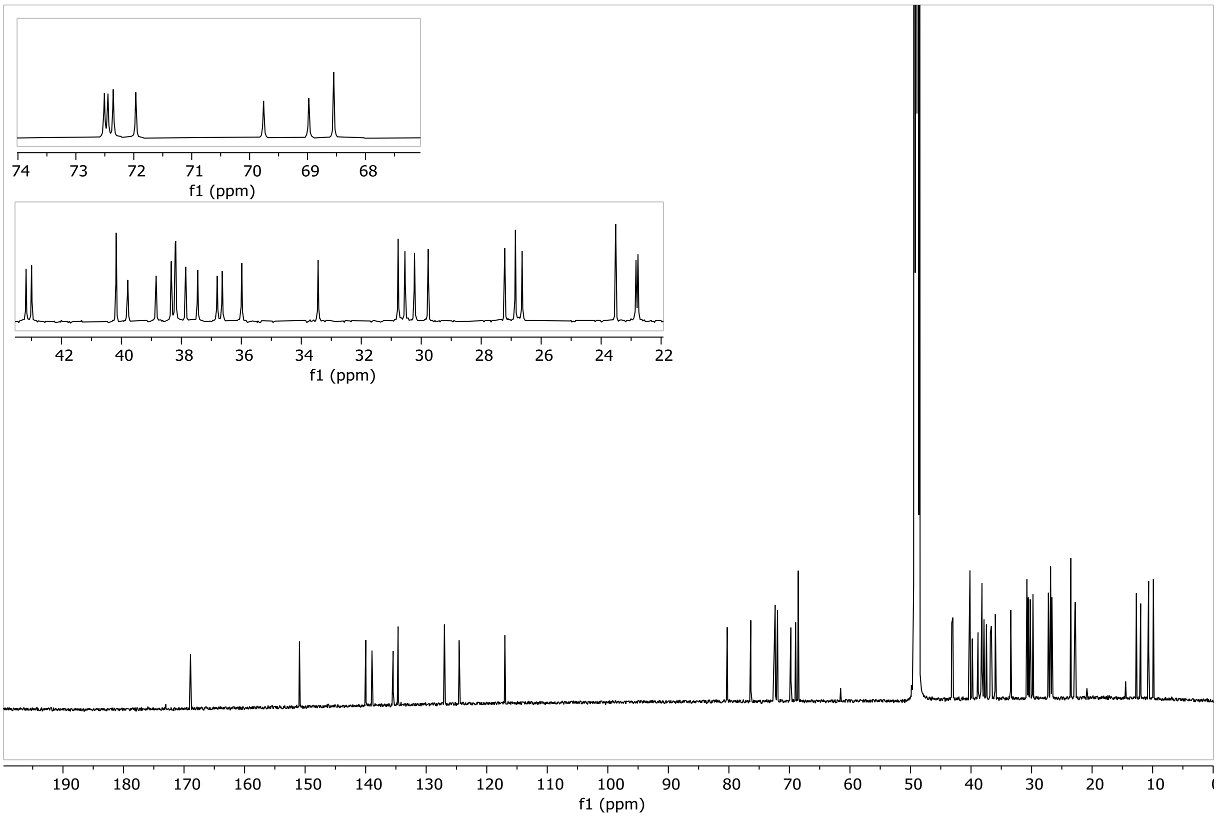

Figure S93 ^{13}C NMR spectrum of **12** in methanol-d_4 (^{13}C 151 MHz).

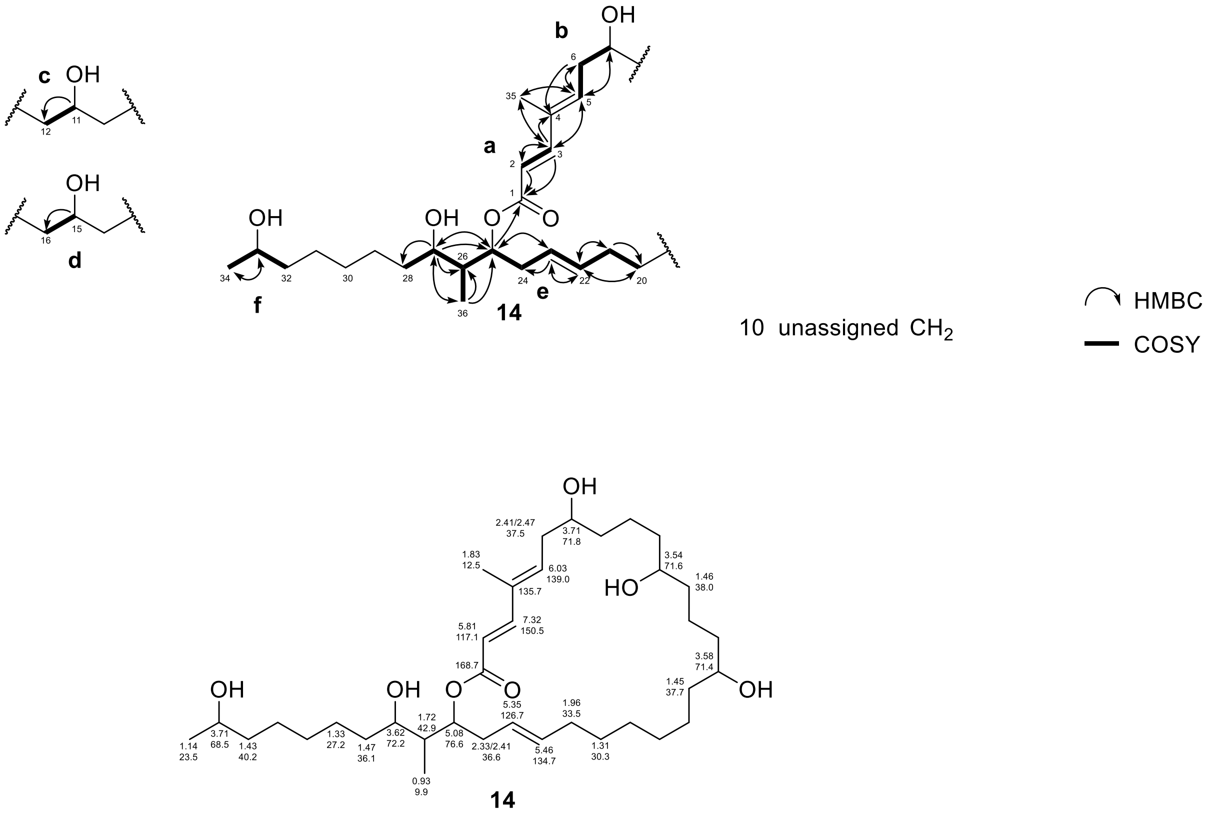

Figure S94 Structural assignment of **14**. Due to overlapping signals not all carbons could be structurally assigned.

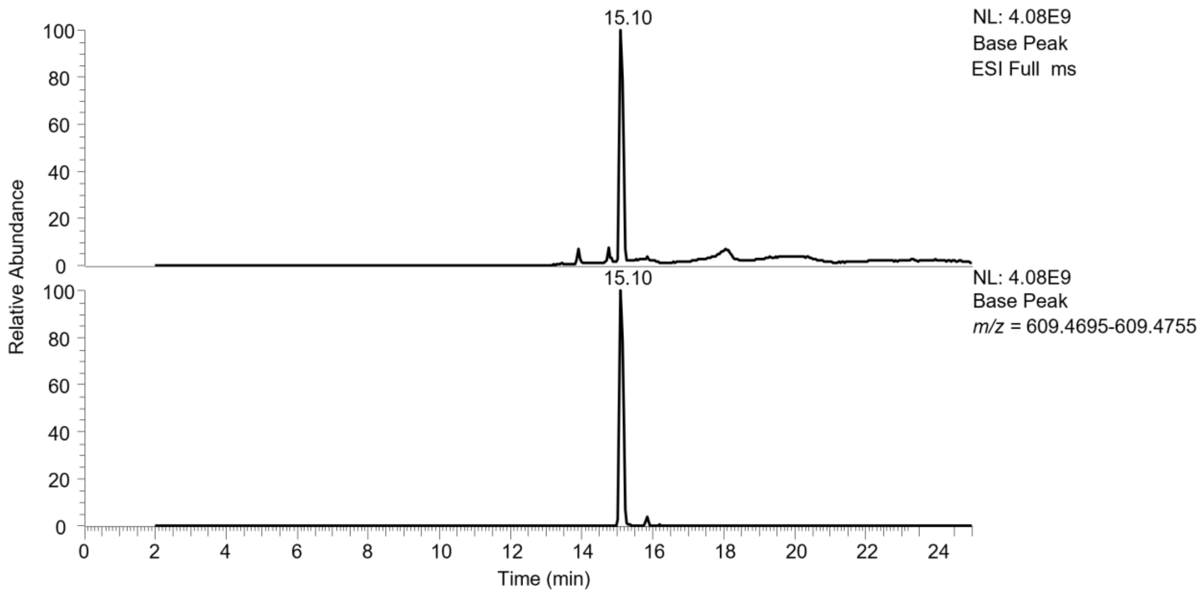

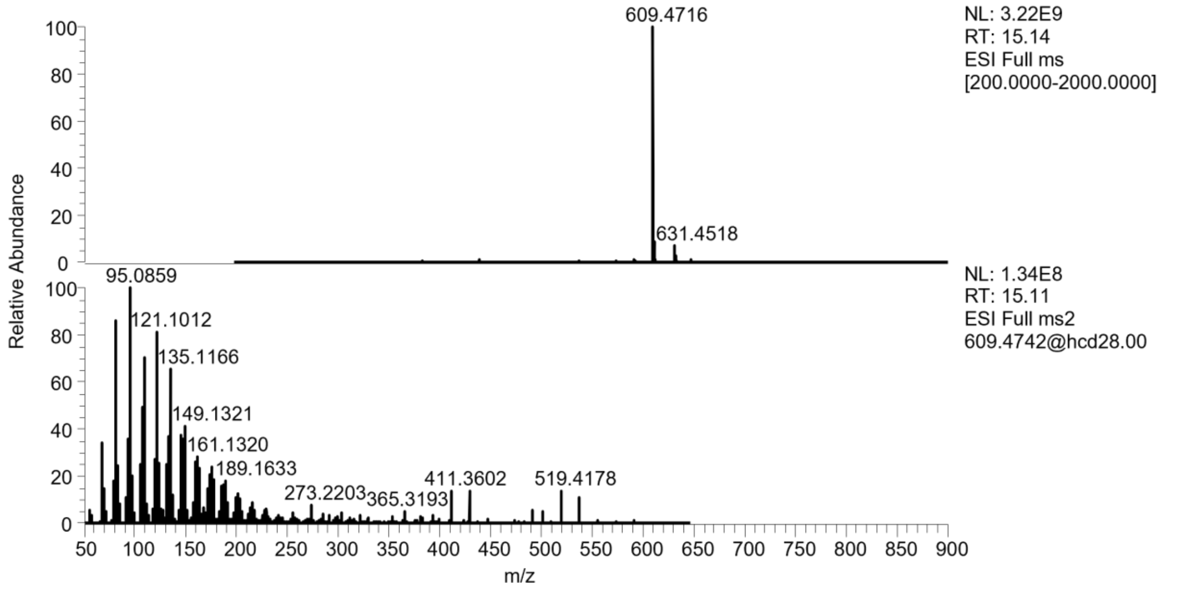

Figure S95 LC-MS analysis of purified **14**. Top: Base peak full MS and extracted ion chromatogram (*m/z* 609.4725 ± 5 ppm) of purified natural product. Bottom: MS and MS/MS fragmentation spectra of **14**.

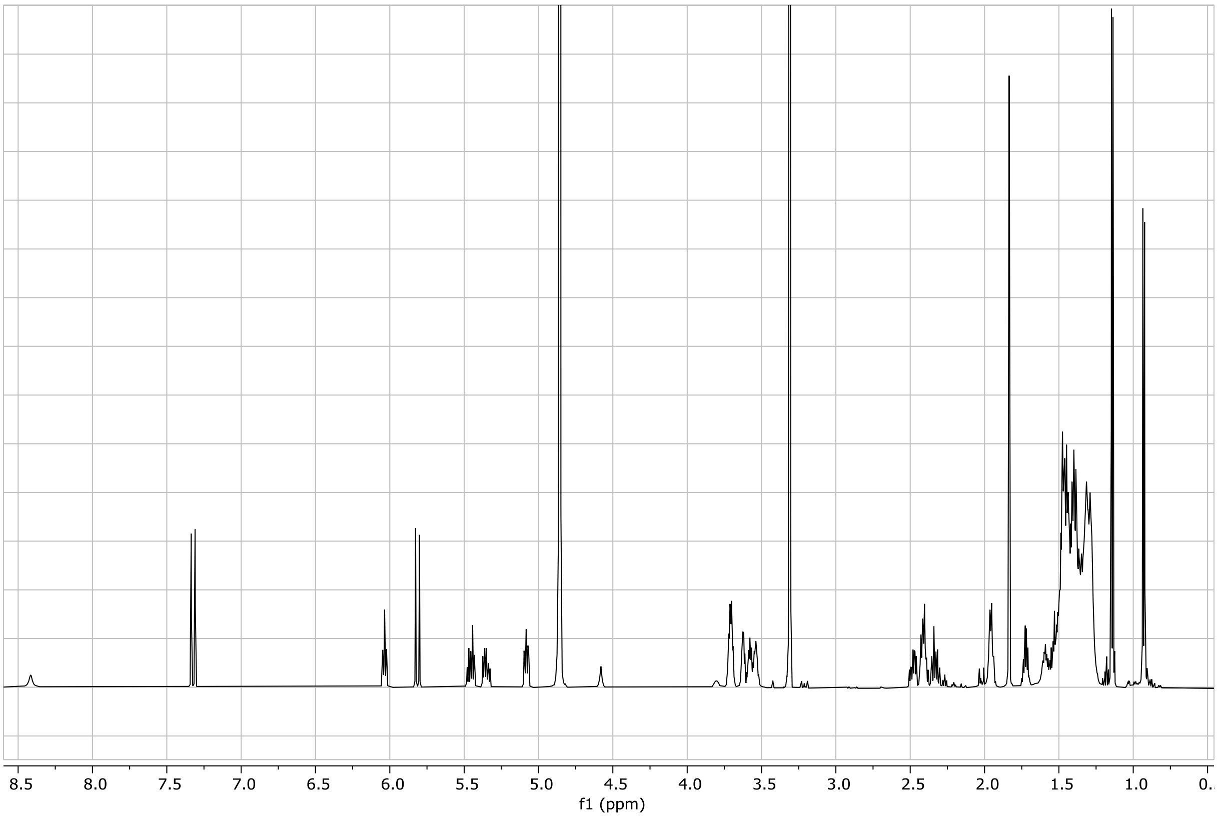

Figure S96 ^{1}H NMR spectrum of **14** in methanol-d_4 (^{1}H 600 MHz).

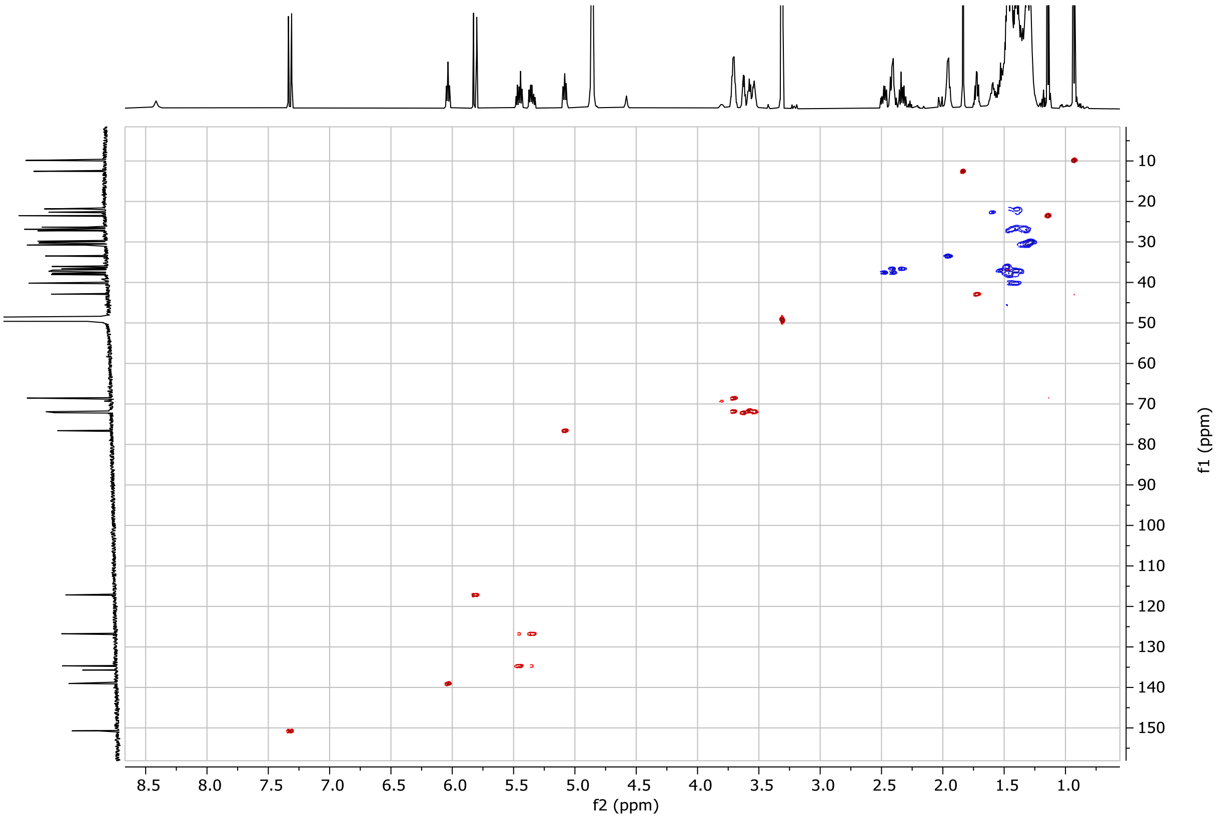

Figure S97 HSQC spectrum of **14** in methanol-d_4 (^{1}H 600 MHz).

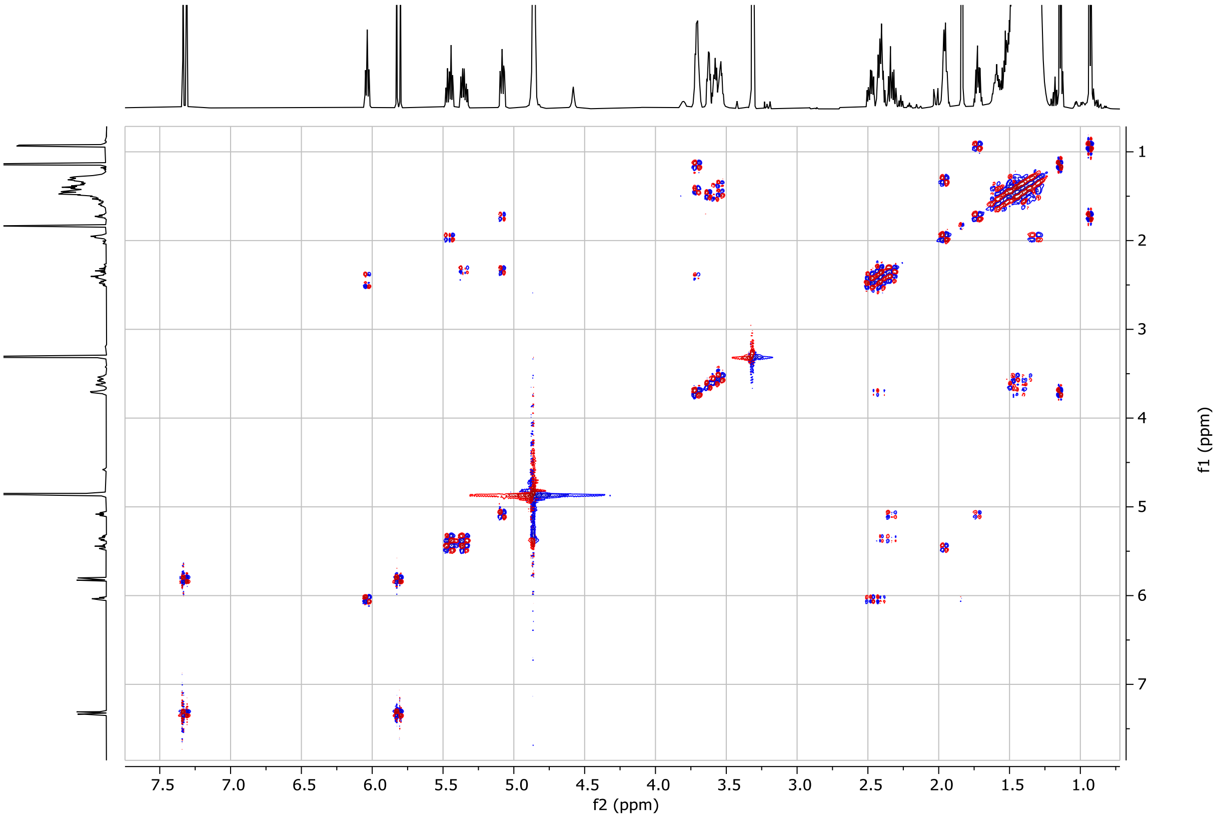

Figure S98 COSY spectrum of **14** in methanol-d_4 (^{1}H 600 MHz, ^{13}C 151 MHz).

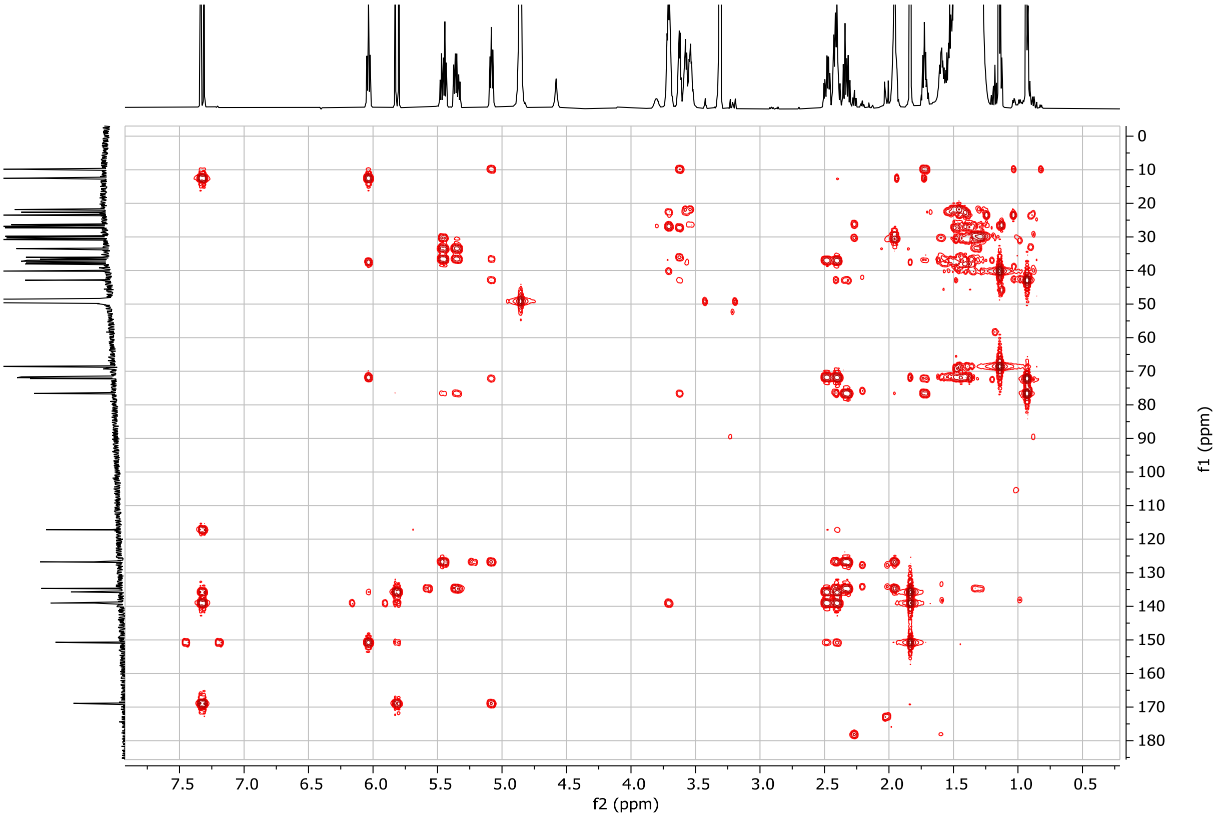

Figure S99 HMBC spectrum of **14** in methanol-d_4 (^{1}H 600 MHz, ^{13}C 151 MHz).

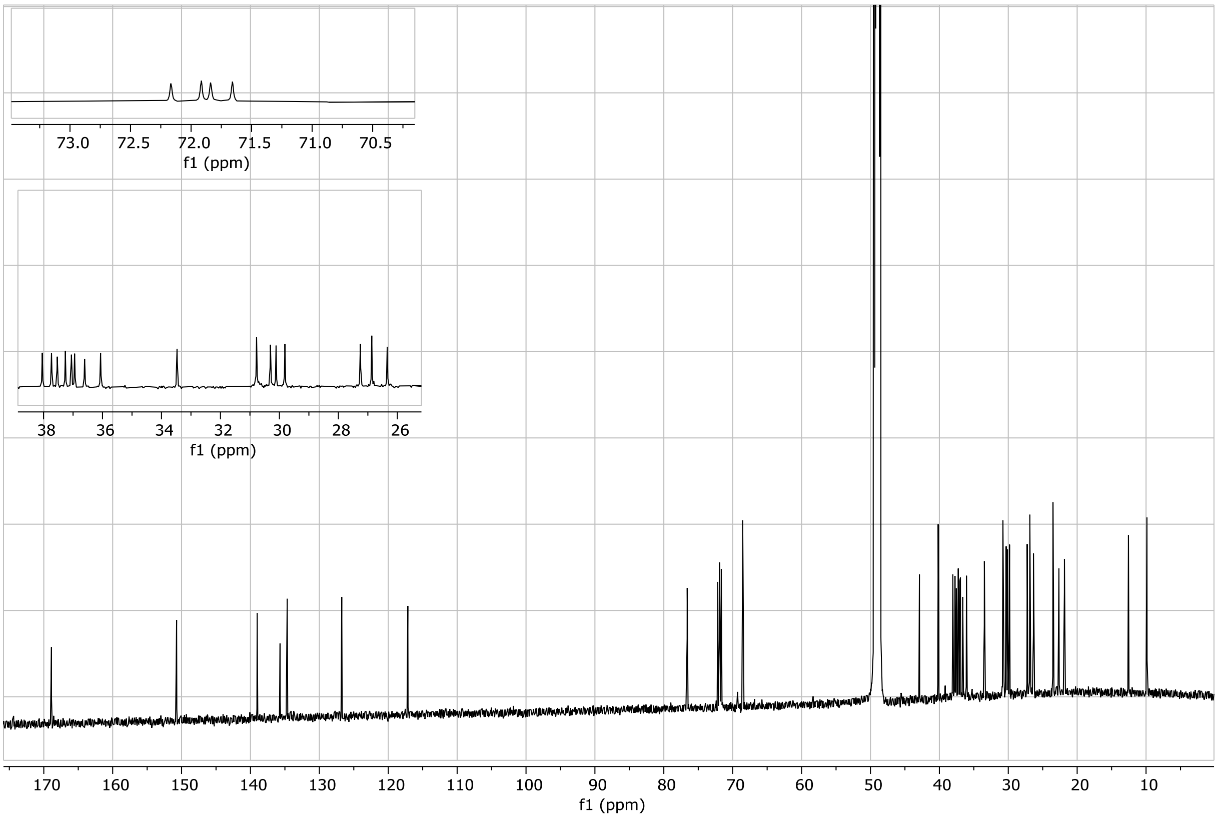

Figure S100 ^{13}C NMR spectrum of **14** in methanol-d_4 (^{13}C 151 MHz).

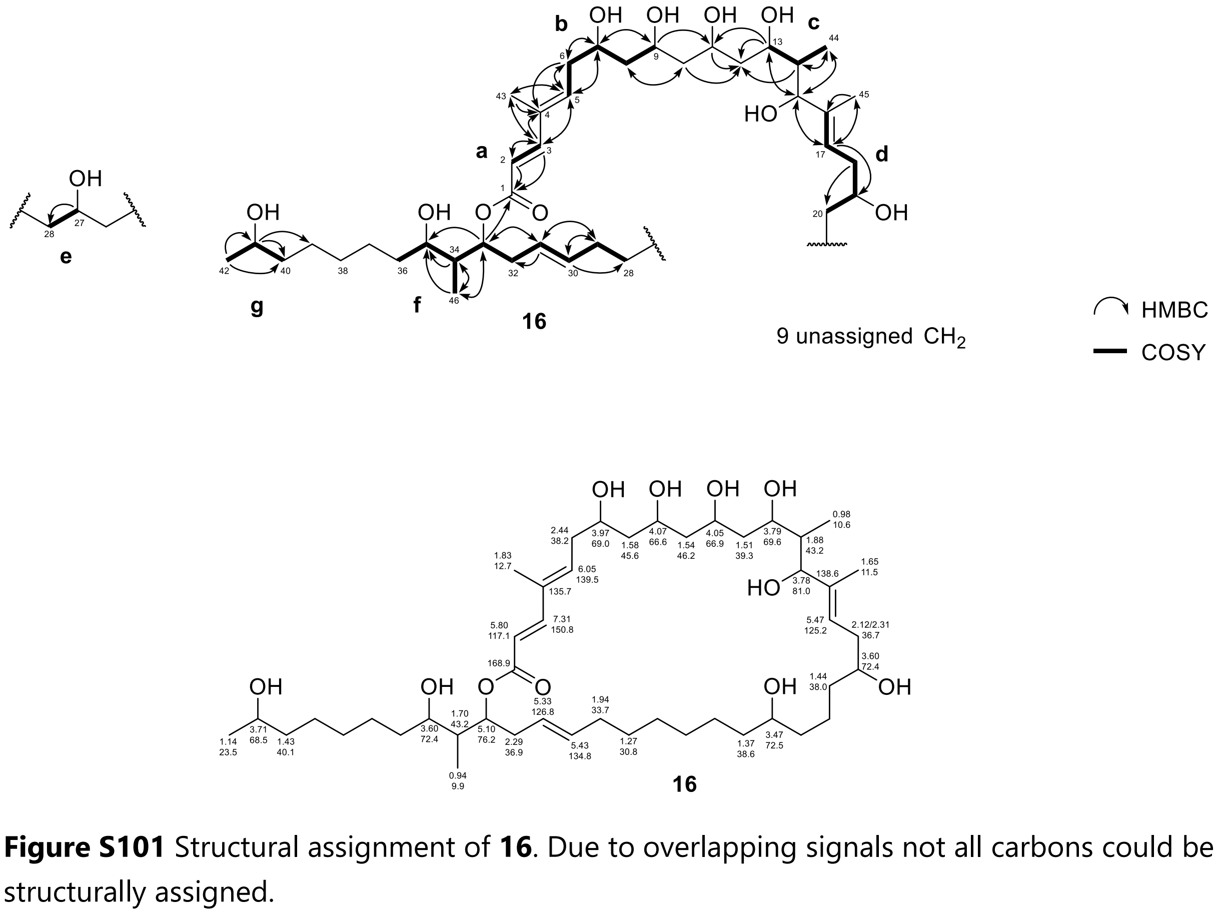

Figure S101 Structural assignment of **16**. Due to overlapping signals not all carbons could be structurally assigned.

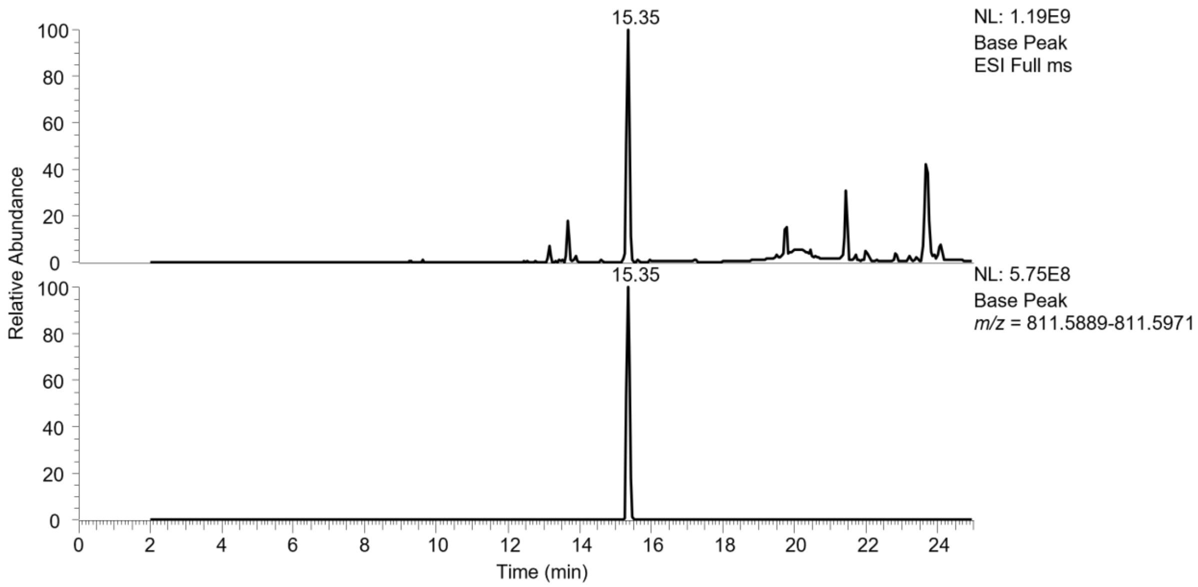

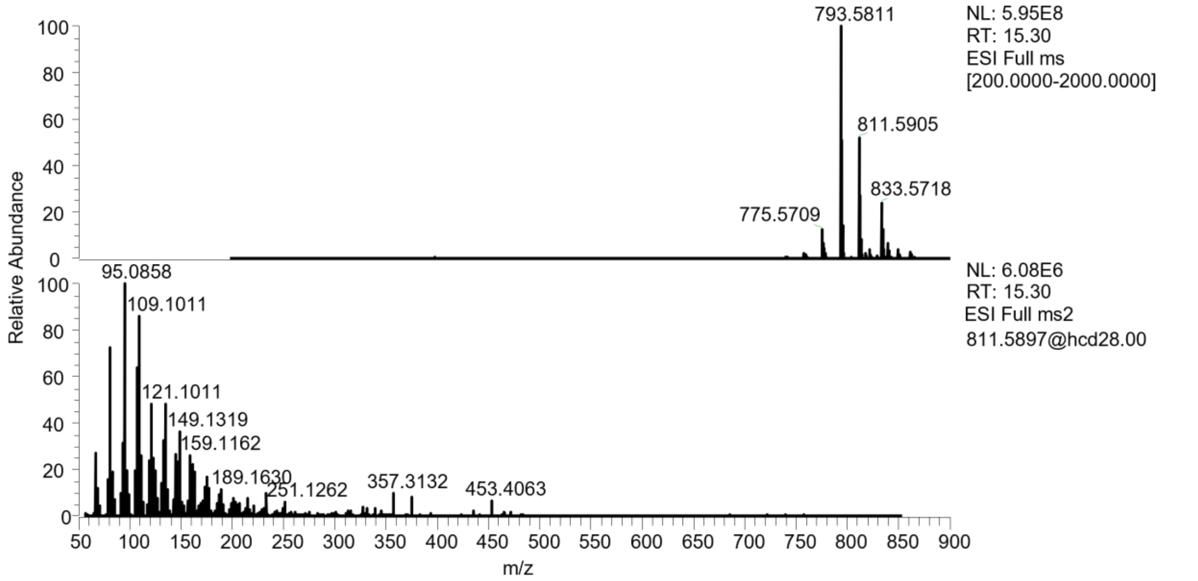

Figure S102 LC-MS analysis of purified **16**. Top: Base peak full MS and extracted ion chromatogram (*m/z* 811.5930 ± 5 ppm) of purified natural product. Bottom: MS and MS/MS fragmentation spectra of **16**.

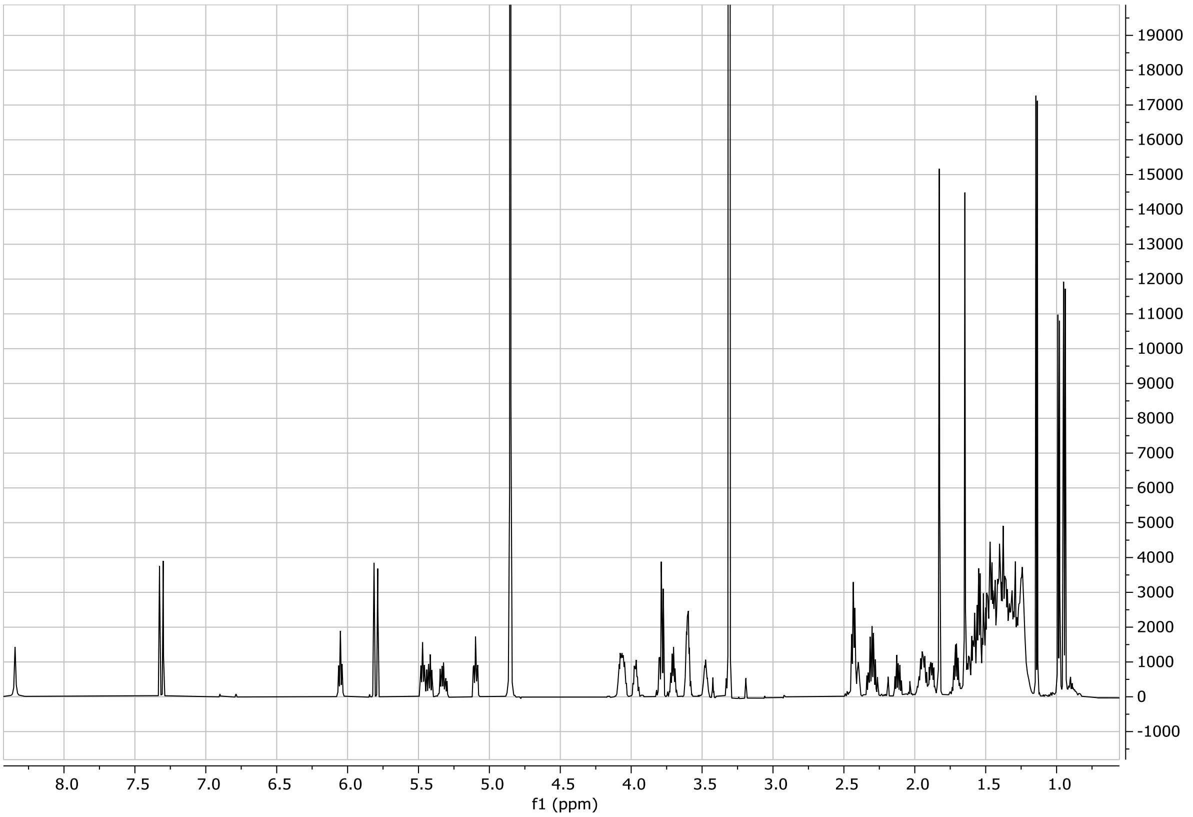

Figure S103 ^1H NMR spectrum of **16** in methanol-d_4 (^1H 600 MHz).

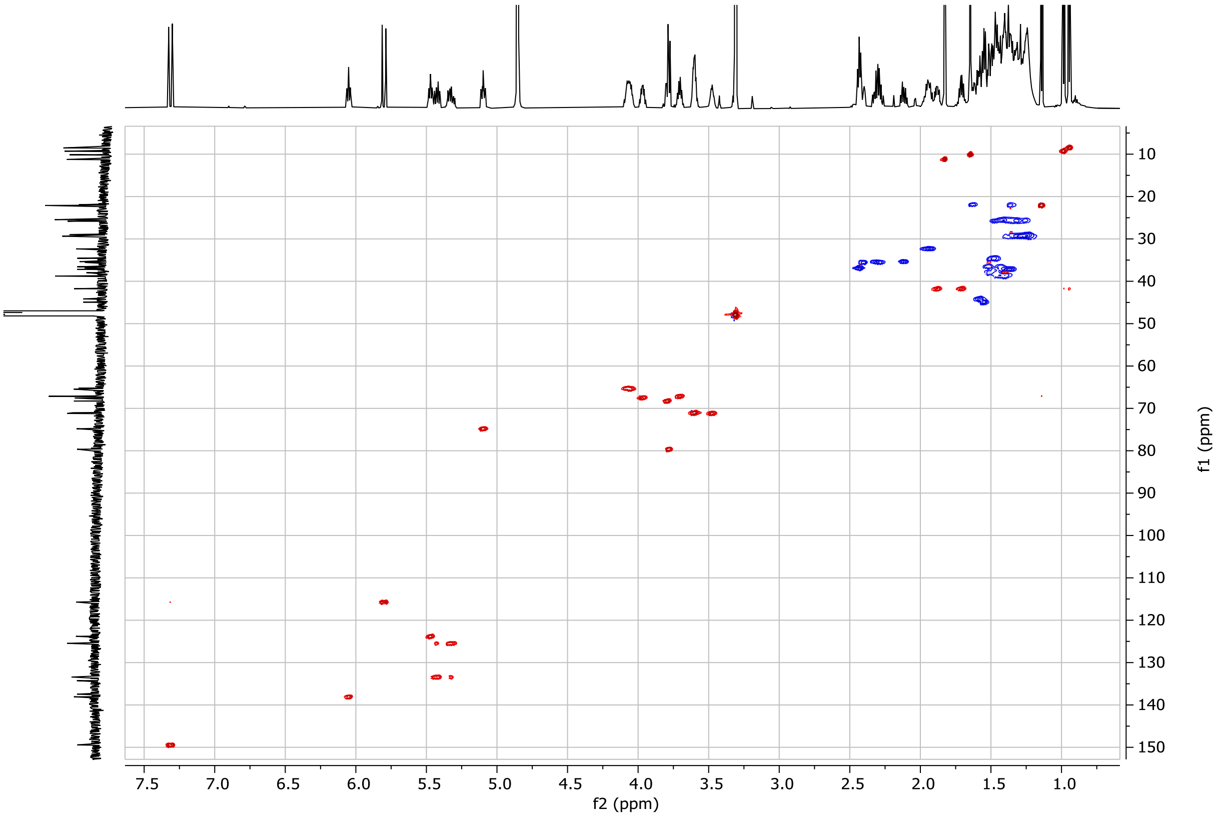

Figure S104 HSQC spectrum of **16** in methanol-d_4 (^1H 600 MHz).

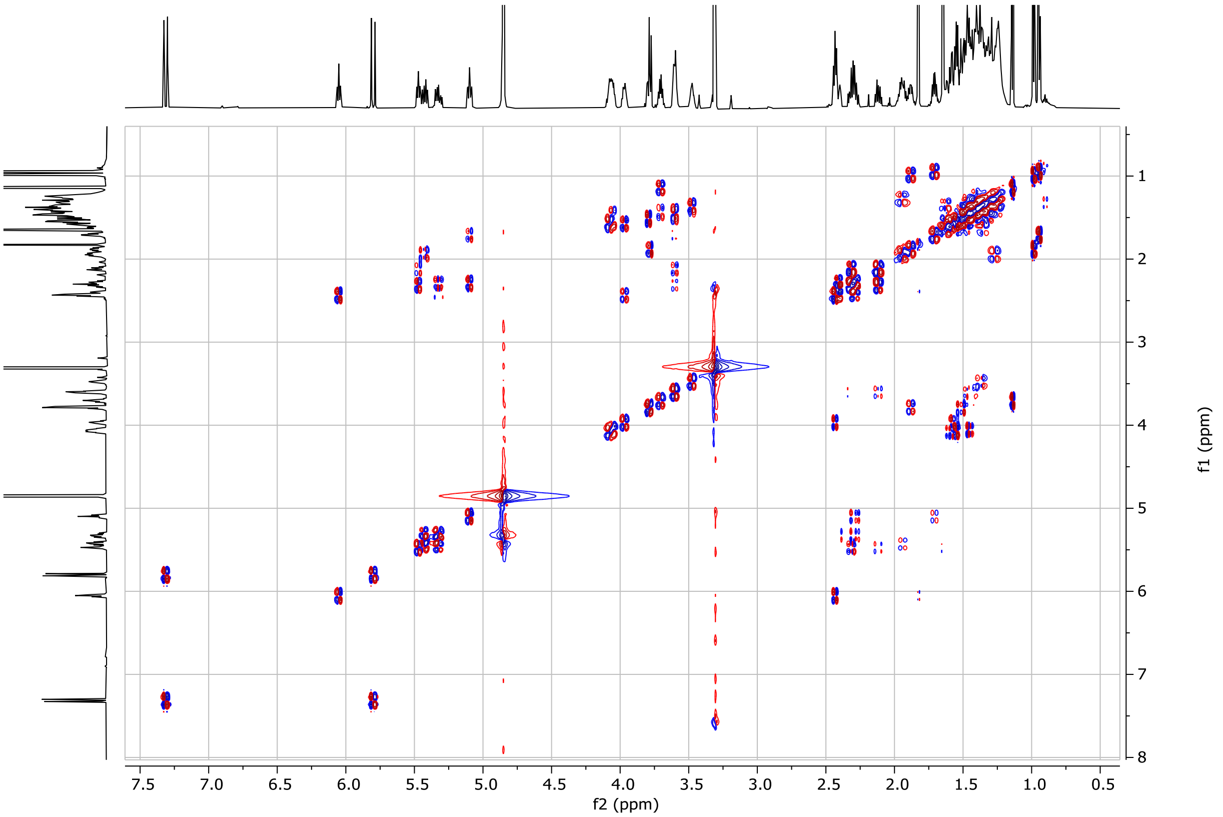

Figure S105 COSY spectrum of **16** in methanol-*d*$_4$ (^{1}H 600 MHz, ^{13}C 151 MHz).

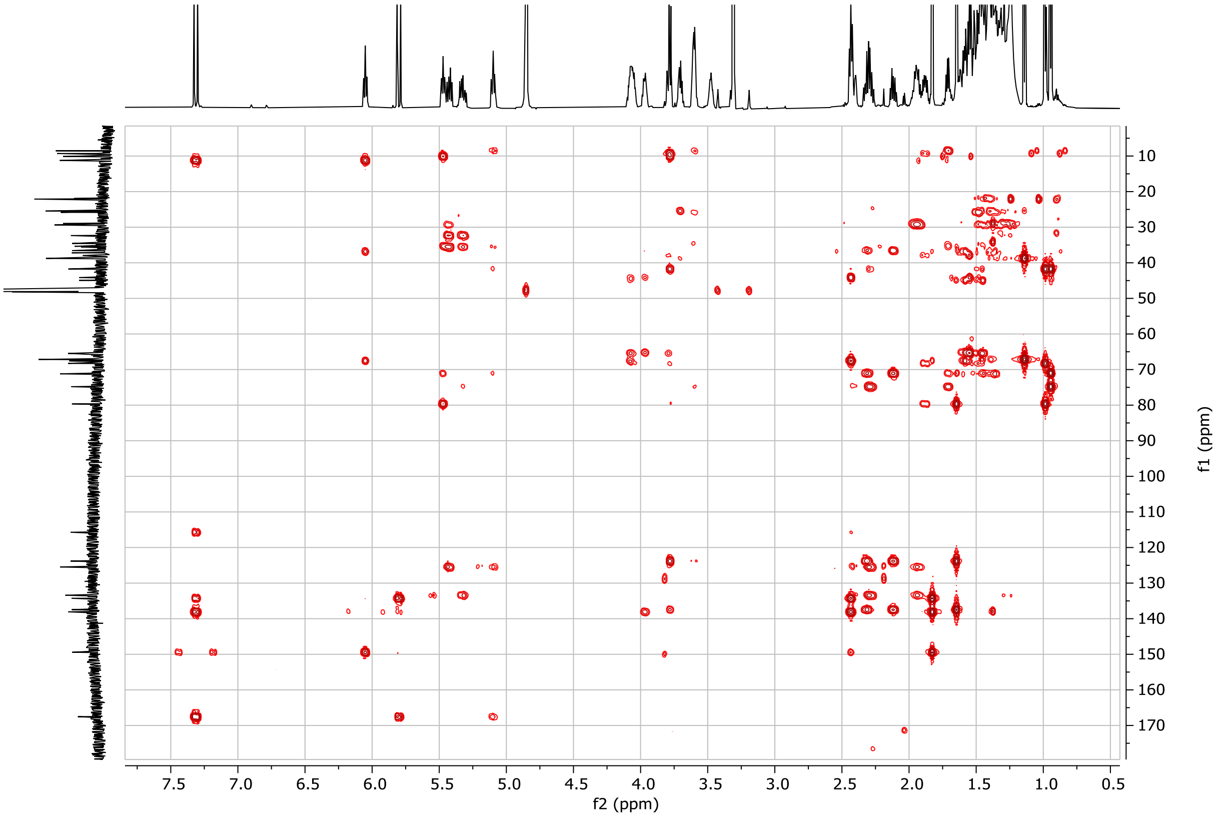

Figure S106 HMBC spectrum of **16** in methanol-*d*$_4$ (^{1}H 600 MHz, ^{13}C 151 MHz).

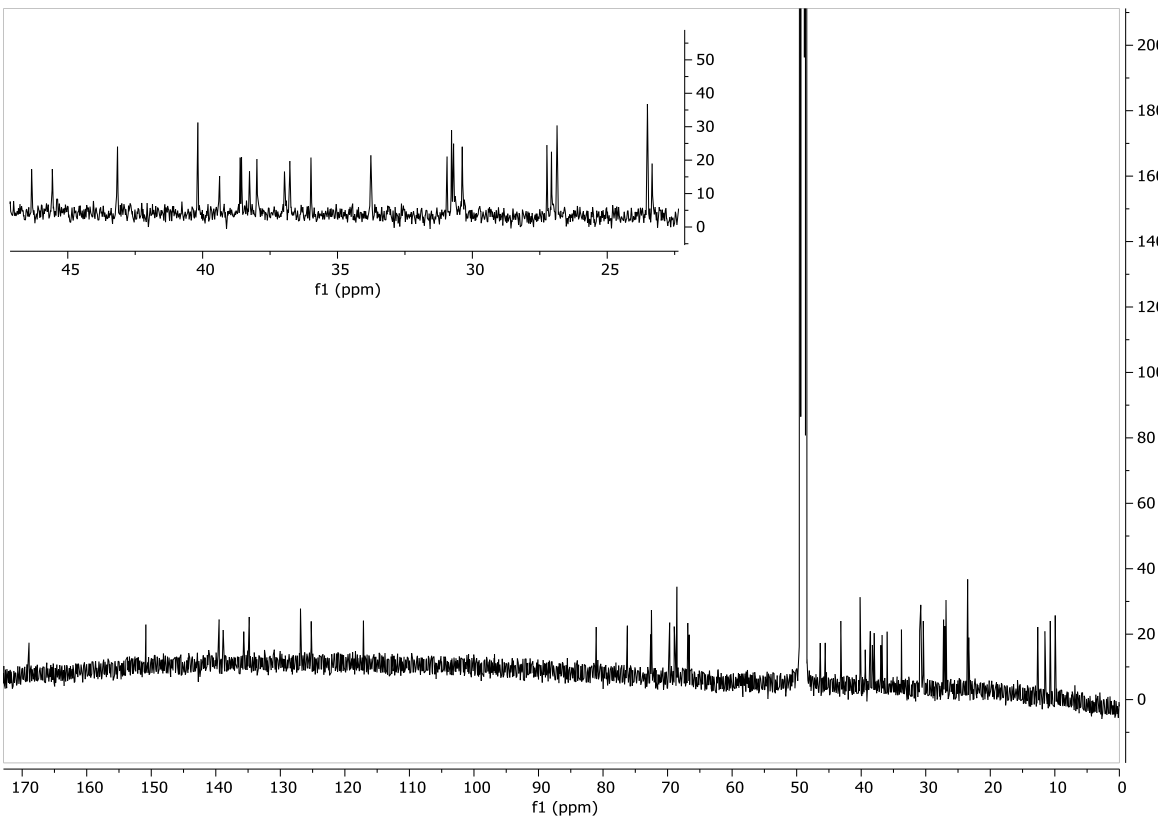

Figure S107 ^{13}C NMR spectrum of **16** in methanol-d_4 (^{13}C 151 MHz).

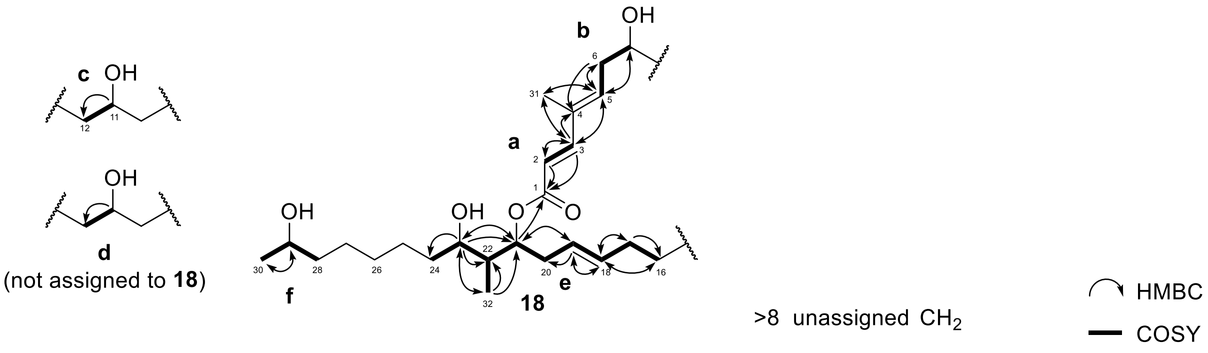

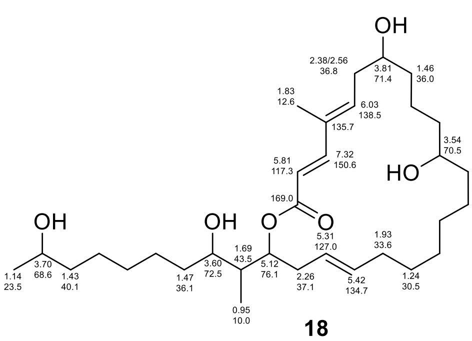

Figure S108 Structural assignment of **18**. Due to overlapping signals not all carbons could be structurally assigned.

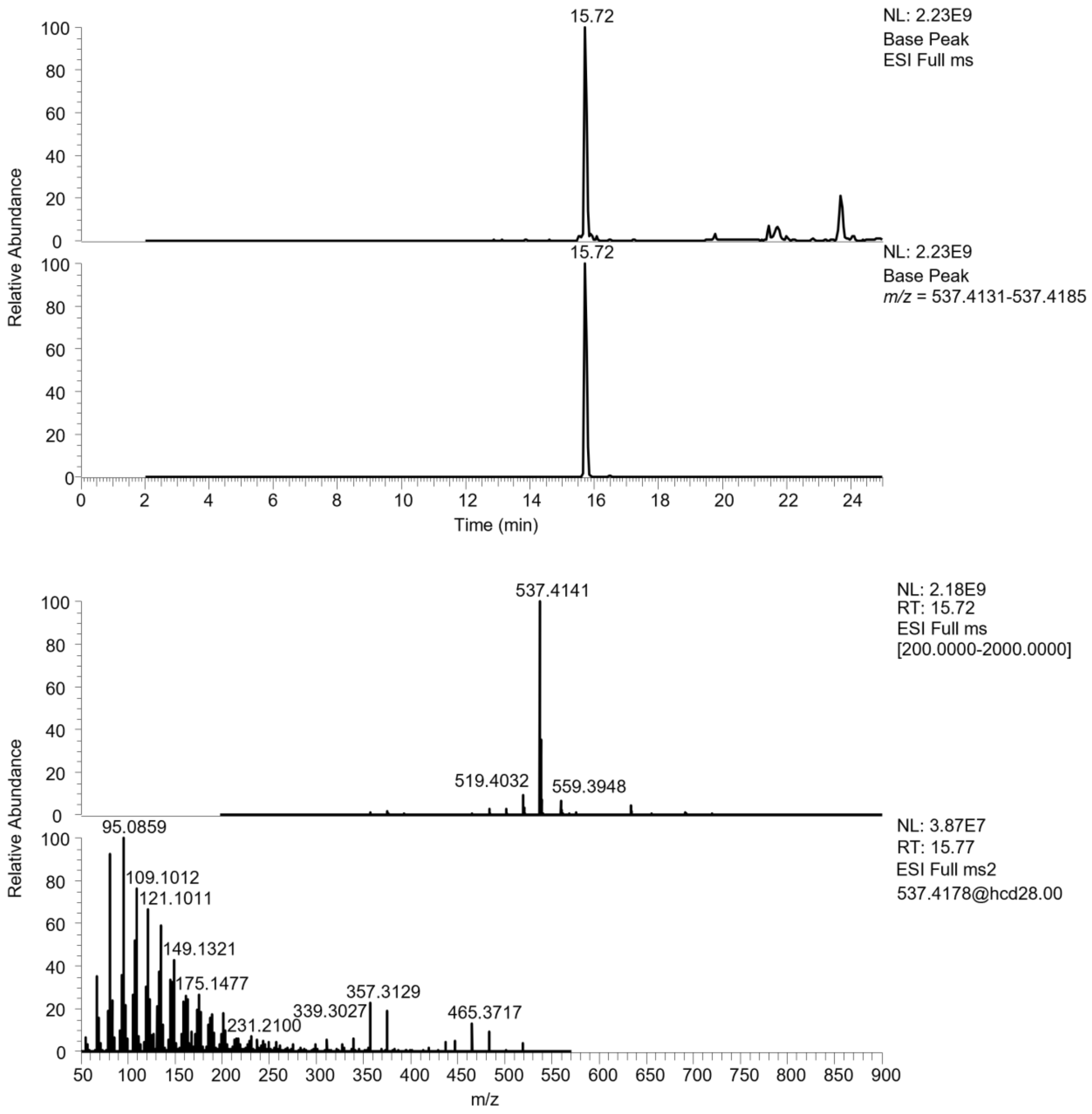

Figure S109 LC-MS analysis of purified **18**. Top: Base peak full MS and extracted ion chromatogram (*m/z* 537.4158 ± 5 ppm) of purified natural product. Bottom: MS and MS/MS fragmentation spectra of **18**.

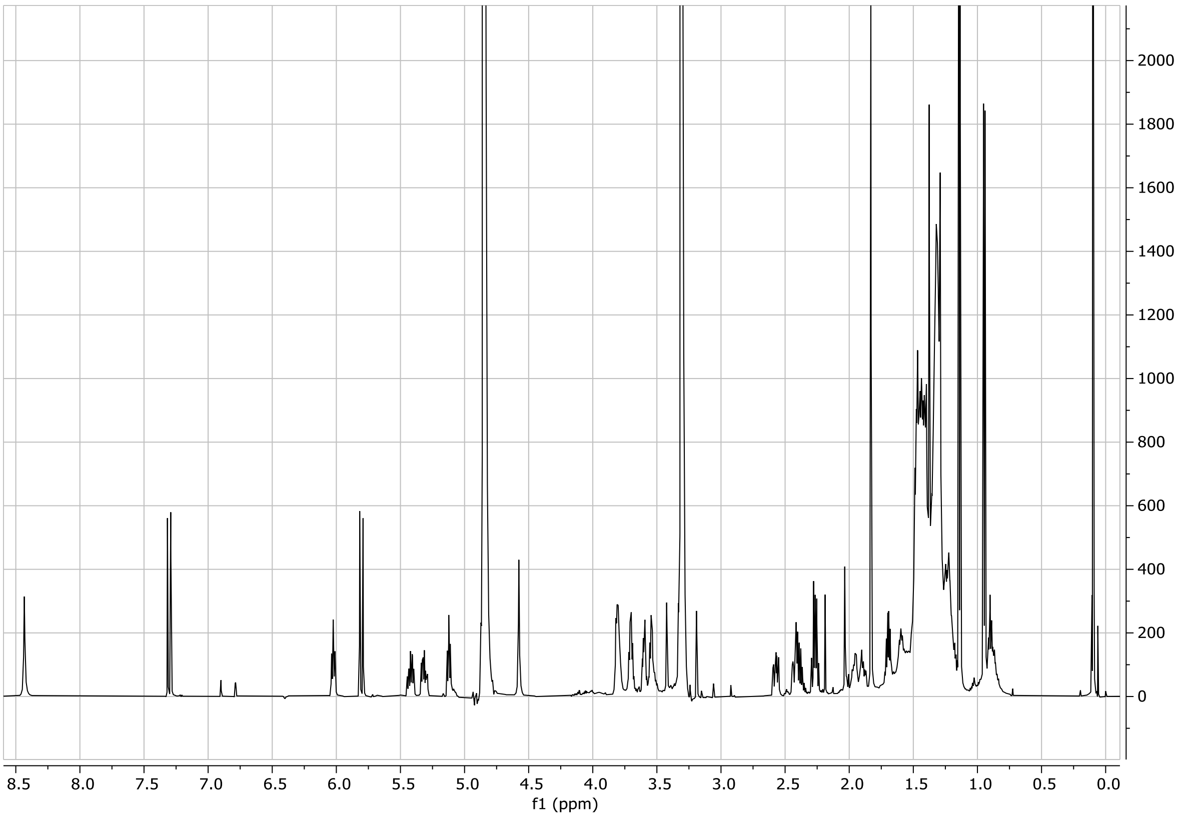

Figure S110 ^{1}H NMR spectrum of **18** in methanol-*d*$_4$ (^{1}H 600 MHz).

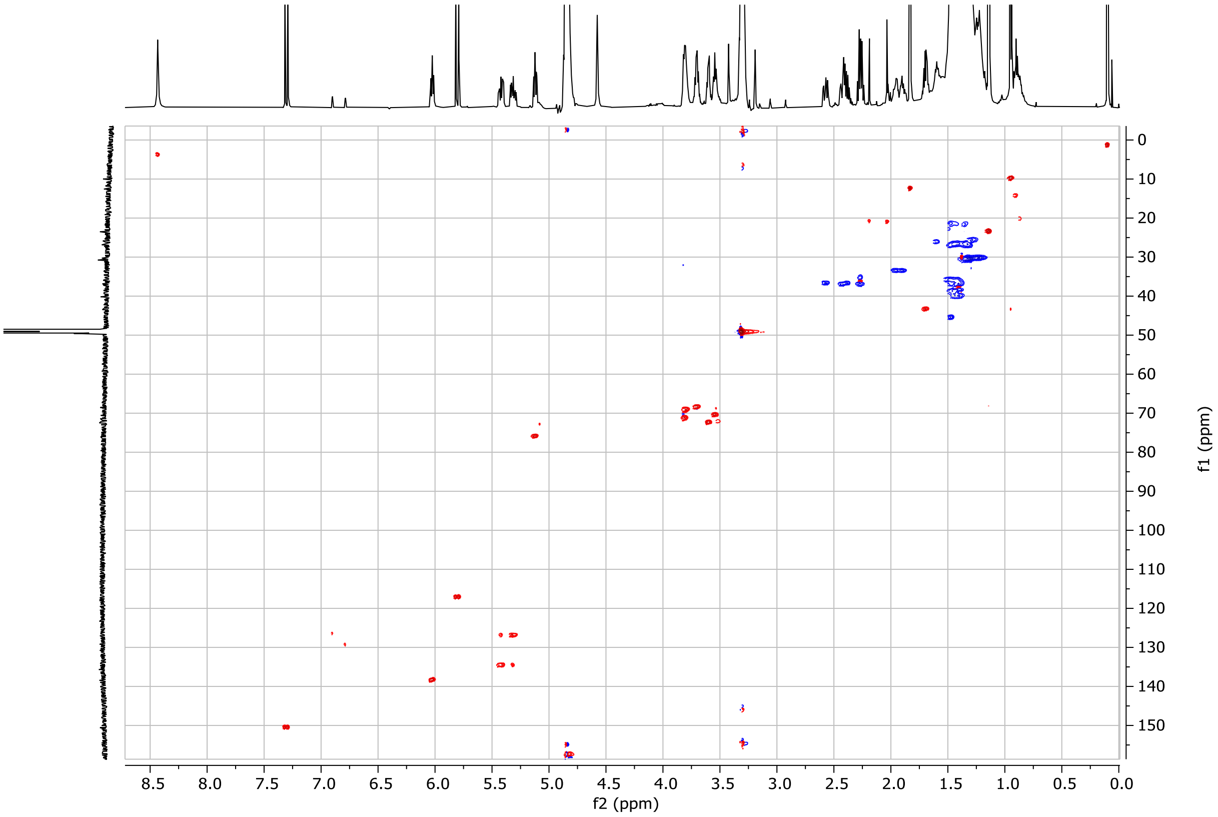

Figure S111 HSQC spectrum of **18** in methanol-*d*$_4$ (^{1}H 600 MHz).

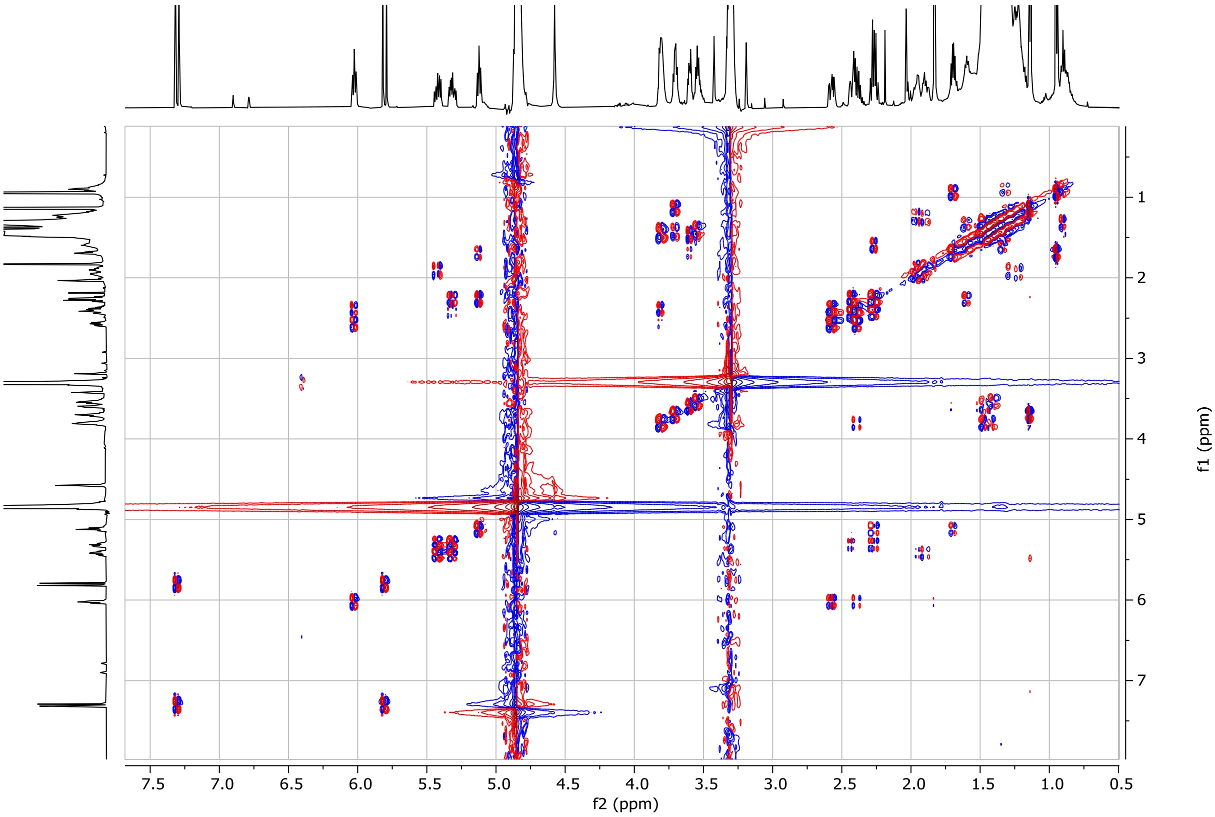

Figure S112 COSY spectrum of **18** in methanol-d_4 (^{1}H 600 MHz, ^{13}C 151 MHz).

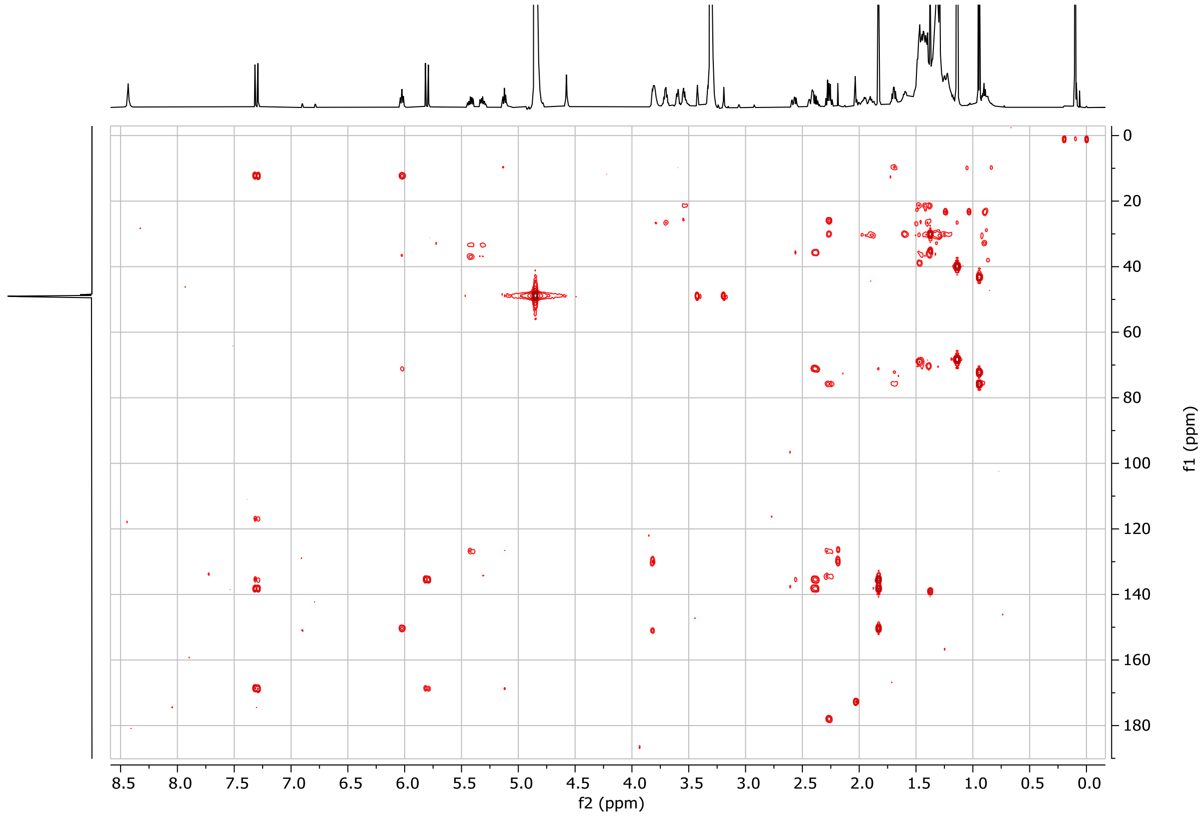

Figure S113 HMBC spectrum of **18** in methanol-d_4 (^{1}H 600 MHz, ^{13}C 151 MHz).

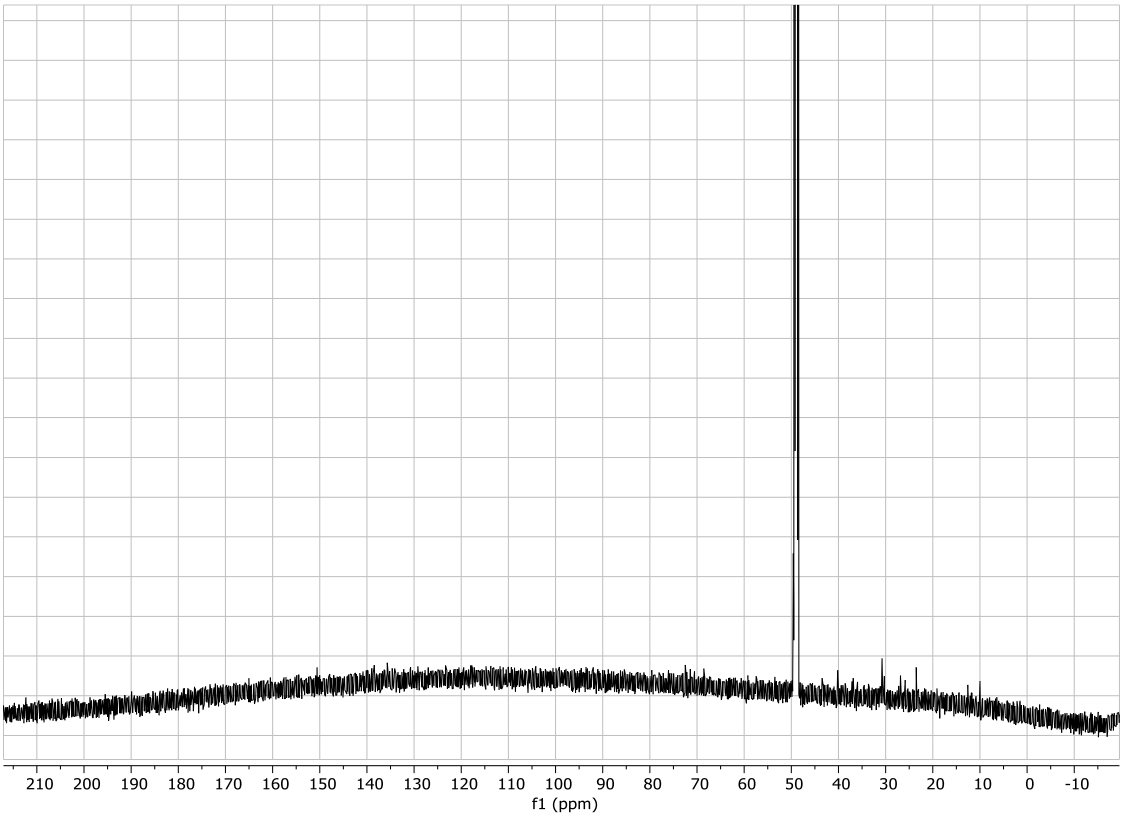

Figure S114 ^{13}C NMR spectrum of **18** in methanol-d_4 (^{13}C 151 MHz).

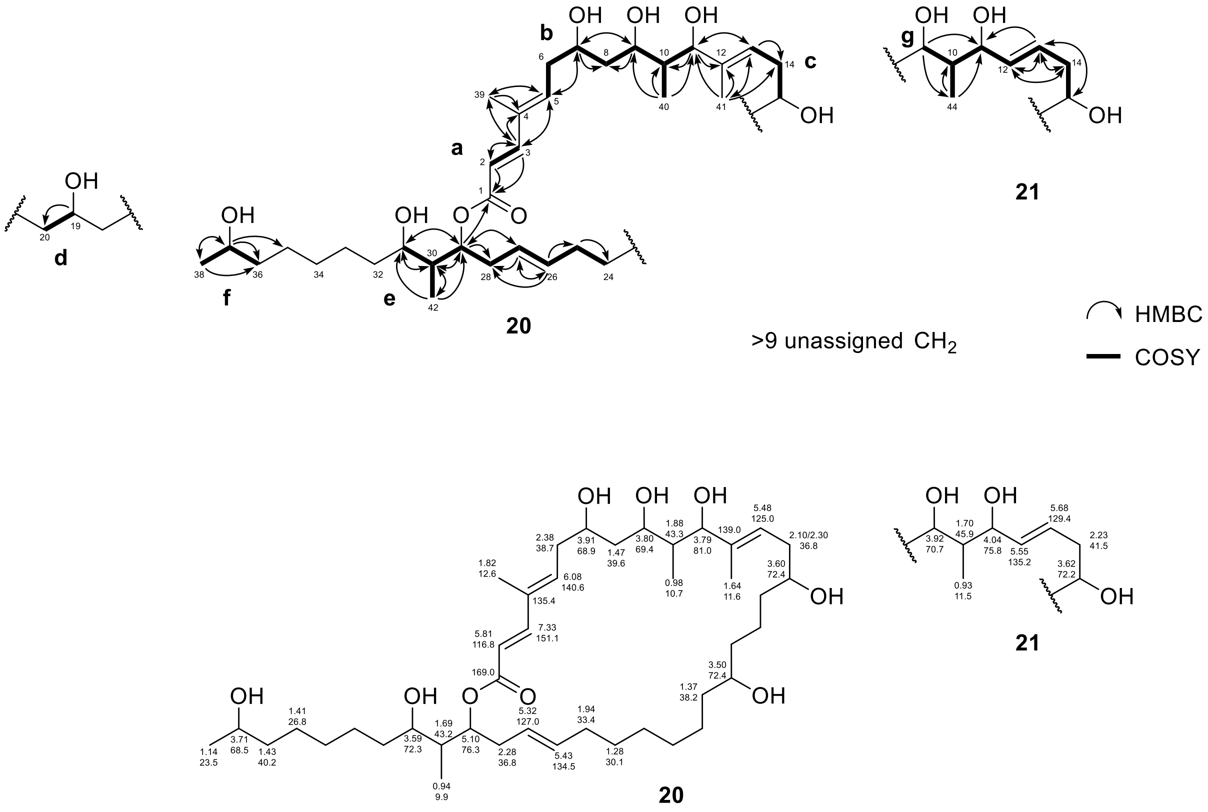

Figure S115 Structural assignment of **20**+**21**. Due to overlapping signals not all carbons could be structurally assigned.

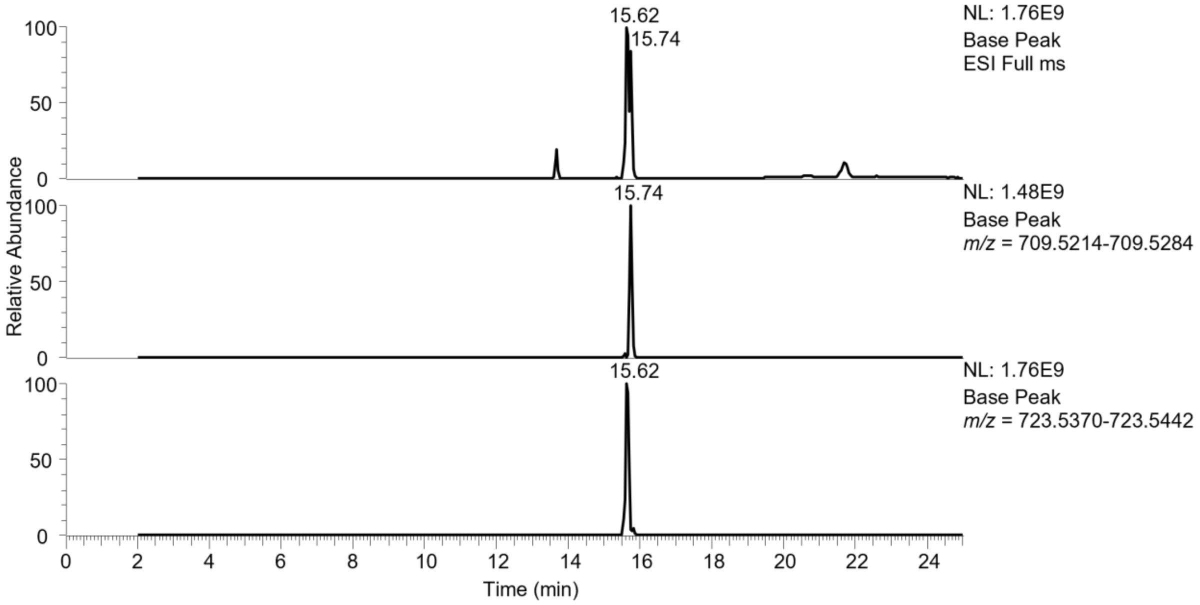

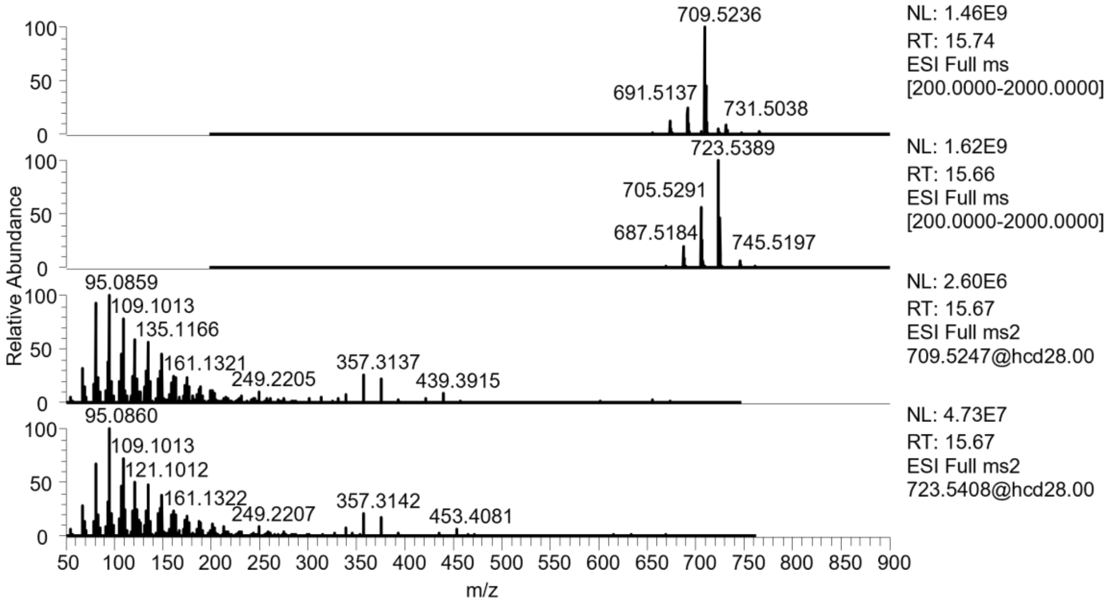

Figure S116 LC-MS analysis of purified **20**+**21**. Top: Base peak full MS and extracted ion chromatogram (*m/z* 723.5406 and *m/z* 709.5239 ± 5 ppm) of purified natural product. Bottom: MS and MS/MS fragmentation spectra of **20+21**.

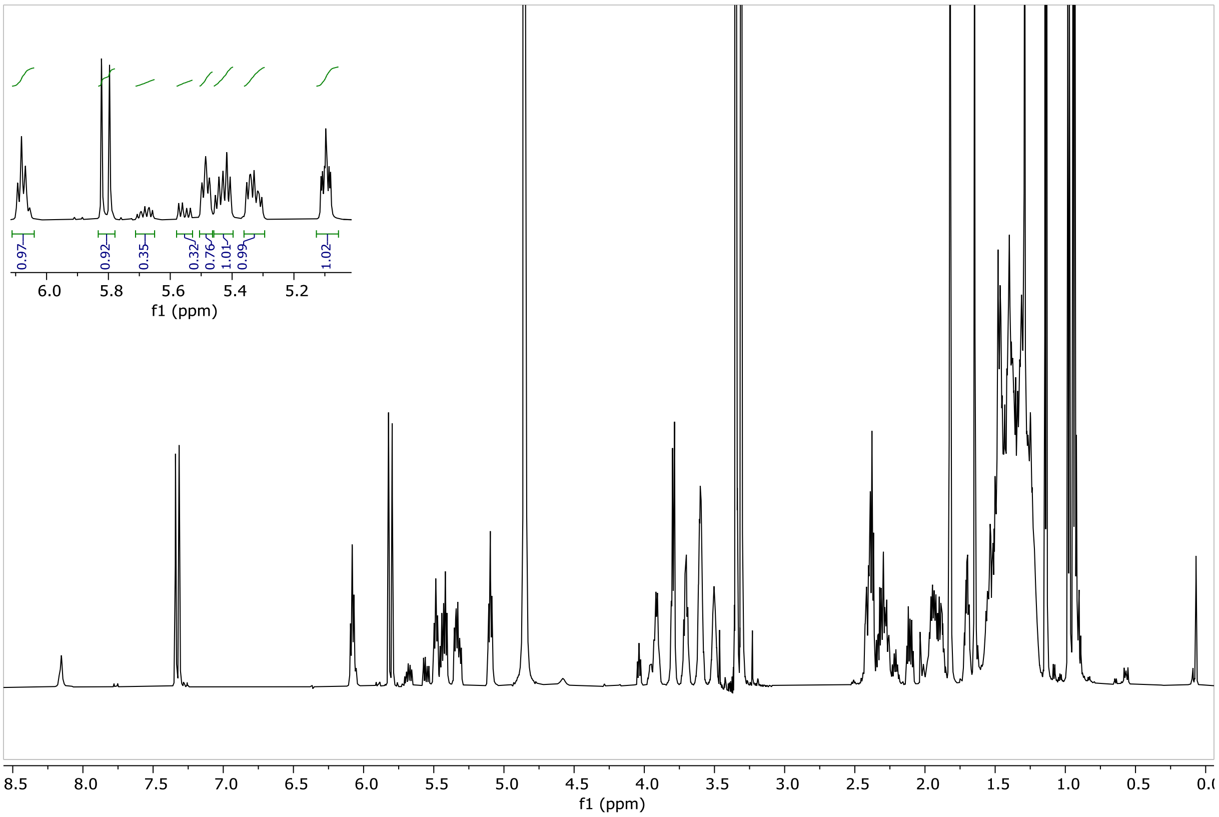

Figure S117 ^{1}H NMR spectrum of **20** + **21** in methanol-d_4 (^{1}H 600 MHz).

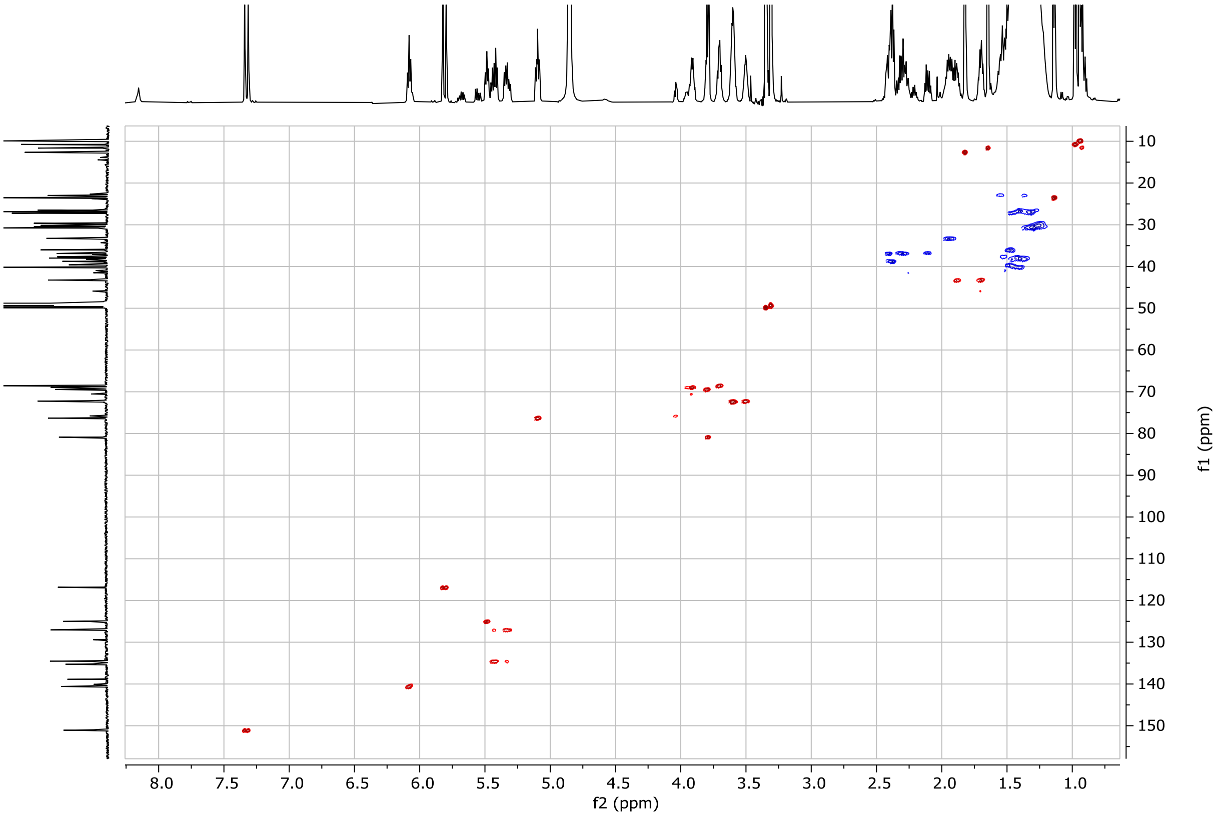

Figure S118 HSQC spectrum of **20** + **21** in methanol-d_4 (^{1}H 600 MHz).

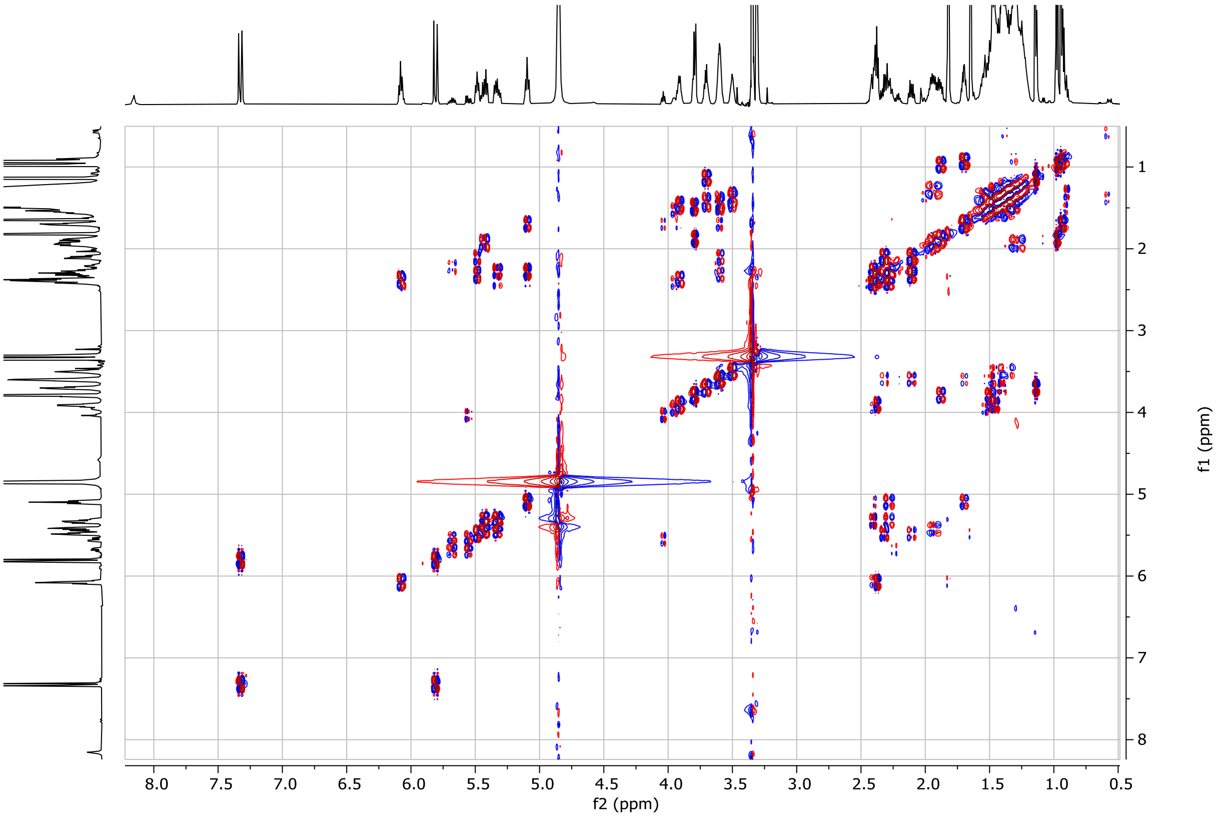

Figure S119 COSY spectrum of **20** + **21** in methanol-d_4 (^{1}H 600 MHz, ^{13}C 151 MHz).

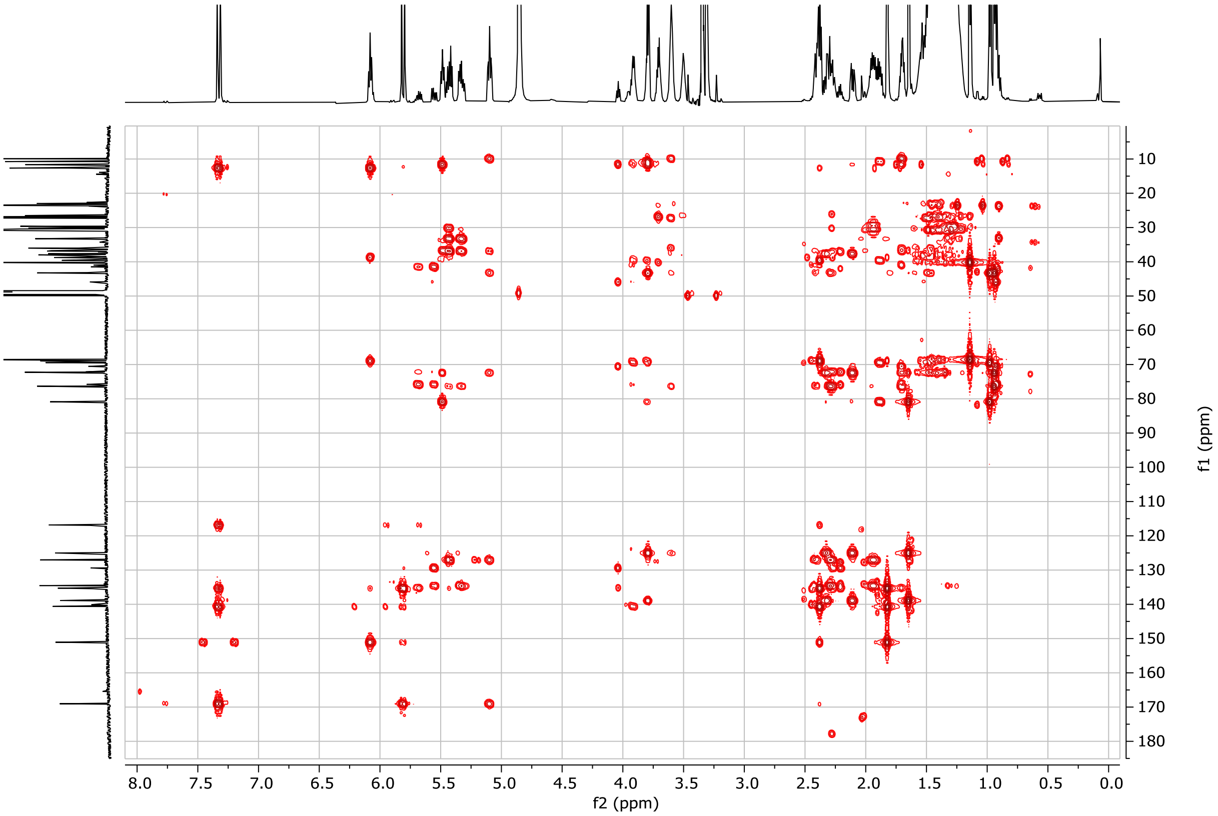

Figure S120 HMBC spectrum of **20** + **21** in methanol-d_4 (^{1}H 600 MHz, ^{13}C 151 MHz).

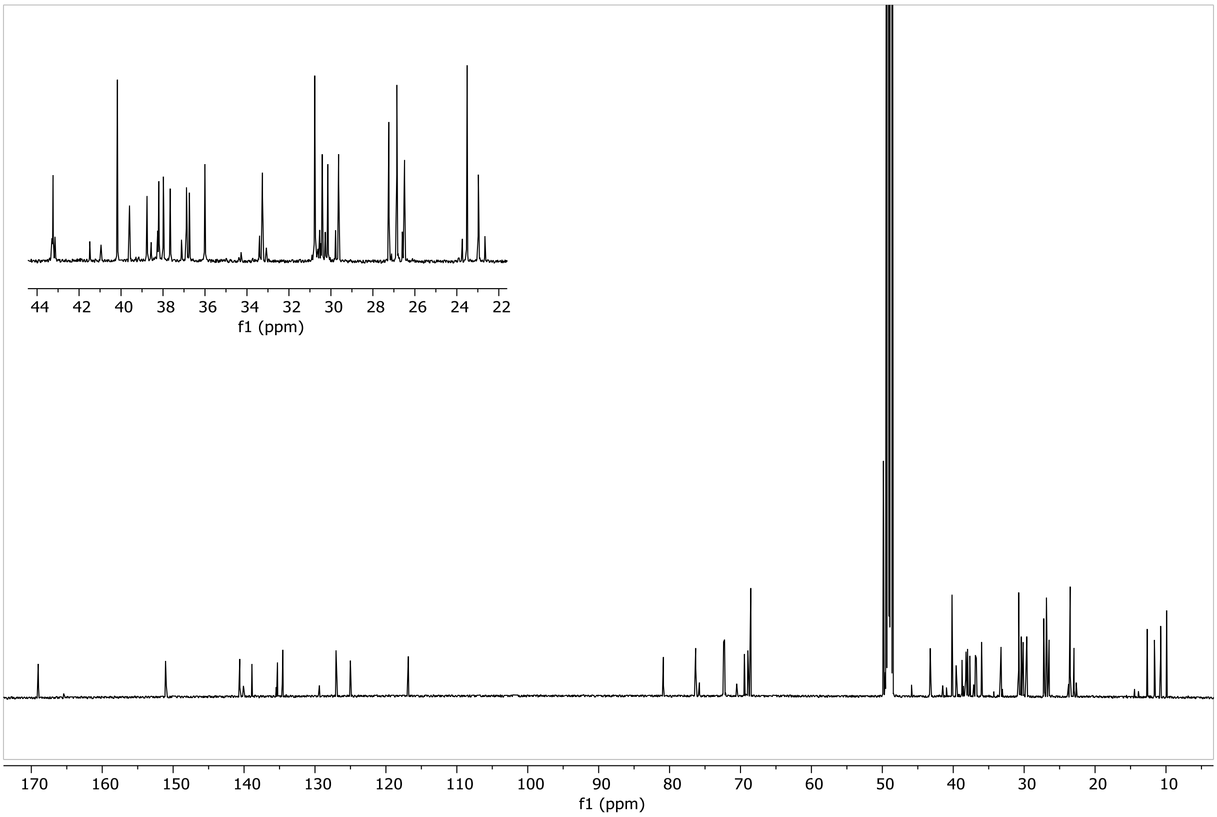

Figure S121 ^{13}C NMR spectrum of **20** + **21** in methanol-d_4 (^{13}C 151 MHz).

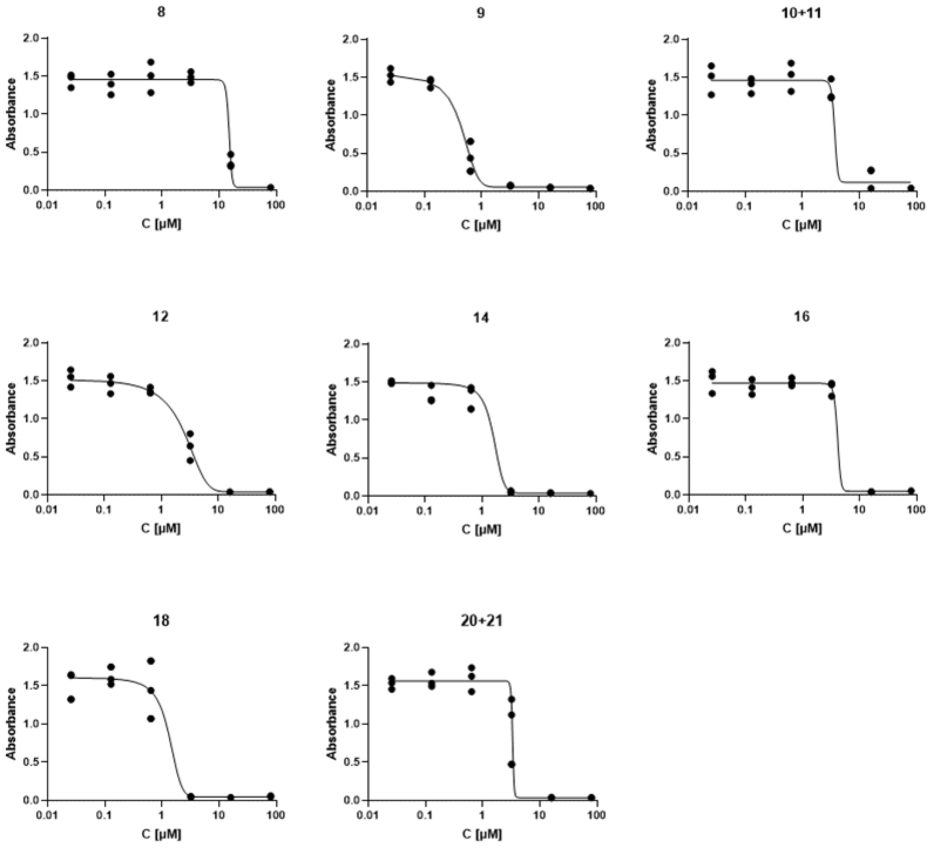

Figure S122 Bioactivity of isolated compounds against HeLa human cervical cancer cells. Cells were treated with different concentrations of compounds **8**-**21** in triplicates. From the data measured in an MTT assay after three days of incubation, the IC_{50}-values were calculated: Compound **8**: IC_{50} = 15.0 µM, R^2 = 0.971. Compound **9**: IC_{50} = 0.4 µM, R^2 = 0.997. Compounds **10+11**: IC_{50} = 3.7 µM, R^2 = 0.959. Compound **12**: IC_{50} = 1.9 µM, R^2 = 0.984- Compound **14**: IC_{50} = 1.6 µM, R^2 = 0.999. Compound **16**: IC_{50} = 4.1 µM, R^2 = 0.987. Compound **18**: IC_{50} = 1.3 µM, R^2 = 0.999. Compound **20**+**21**: $IC_{50,}$ = 3.3 µM, R^2 = 0.988. The curves were fit with a least squares regression using a four-parameter logistic function. For the fit, outliers were eliminated using a ROUT coefficient Q=1% in GraphPad Prism 9.0.2 (GraphPad Software). The NMR determined ratio of the mixtures **10**+**11** (8:2 ratio; effective MW 880.4526 g/mol) and **20**+**21** (7:3 ratio; effective MW 718.8369 g/mol) was used to calculate the effective molecular weight. Lacunalide A (**6**) has an IC_{50} of 1 µM (*51*).

Table S17 Purification of lacunalides from different *G. sunshinyii* mutants. All solvents contained 0.1 vol% formic acid.

***Gynuella sunshinyii* mutant**	**compound**	**Retention time 1st purification step - tR [min]**	**Retention time 2nd purification step - tR [min]**	**Initial MeCN in H_2O ratio steps 1 and 2**	**Retention time 3rd purification step - tR [min]**	**initial MeCN in H_2O ratio step 3**	**Yield [mg]**	**Yield per volume [mg/L]**
Δ1415	**8**	26-30	25	37	NA	NA	4.2	0.70
Δ1415	**9**	26-30	21-23	37	33	40	0.2	0.04
Δ2122	**10**	27-30	18	43	40	43	2.4	0.40
Δ2122	**11**	27-30	18	43	40	43	0.6	0.10
Δ2023	**12**	27-30	25	43	NA	NA	4.0	0.67
Δ1724	**14**	27-30	26	43	NA	NA	1.5	0.26
Δ1415 + Δ2122	**16**	27-30	28	43	48	43*	0.7	0.11
Δ1415 + Δ1724	**18**	27-30	28-32	43	26	43	0.2	0.03
Δ1415 + Δ2023	**20**	27-30	25-28	43	61	43	2.9	0.49
Δ1415 + Δ2023	**21**	27-30	25-28	43	61	43	1.3	0.21
column		Phenomenex Luna 5µ C18, φ 20 x 250 mm	Phenomenex Luna 5µ Phenyl-Hexyl, φ 10 x 250 mm		Phenomenex Synergi 4µ Hydro-RP, φ 10 x 250 mm,			
flow rate and UV		15.0 mL/min, 200 nm	2.0 mL/min, 200 nm		2.0 mL/min, 200 nm			
gradient		MeCN in H2O + 0.1% formic acid as mobile phase, starting from isocratic 5% MeCN for 5 min, gradient from 5% to 95% MeCN for 32 min, and isocratic elution 95% MeCN for 10 min to afford 40 fractions		Isocratic flow for 55 min; 10 min at 100 % MeCN		Isocratic flow for 55 min; 10 min at 100 % MeCN; *: 40 min isocratic; grad to 100 for 15 min; 10 min at 100%		

Table S18 NMR data for compounds **6, 8, 9**, **10**, **11**, **12**, **14**, **16**, **18**, **20**, and **21**. Carbons missing in the deletion mutants are highlighted in gray. The positions of each proton ID can be found in the structural assignment figure of each compound, which is indicated in the column header. In the multiplicity column, the *J*-coupling of the peak in Hz is indicated in brackets. Ovlp. Indicates overlapping peaks. N.A. is not assigned.

**6**, structural assignment: Fig. S54				**8**, structural assignment: Fig. S72				**9**, structural assignment: Fig. S72				**10**, structural assignment: Fig. S80			
**ID**	**C**	**H**	**Multi-plicity**	**ID**	**C**	**H**	**Multi-plicity**	**ID**	**C**	**H**	**Multi-plicity**	**ID**	**C**	**H**	**Multi-plicity**
1	168.9			1	168.9	-		1	168.9	-		1	168.9	-	
2	117.1	5.81	d (15.7)	2	117.1	5.80	d (15.7)	2	117.0	5.80	d (15.7)	2	117.0	5.81	d (15.7)
3	150.8	7.32	d (15.7)	3	150.8	7.31	d (15.7)	3	150.7	7.31	d (15.7)	3	150.9	7.32	d (15.7)
4	135.7			4	135.7	-		4	135.7	-		4	135.5	-	
5	139.4	6.04	t (7.3)	5	139.3	6.04	t (7.3)	5	139.2	6.04	t (7.4)	5	139.9	6.06	t (7.1)
6	38.3	2.44	m	6	38.5	2.45	m	6	38.3	2.45	m	6	38.5	2.42	
7	68.9	3.98	m	7	68.8	4.00	m	7	68.8	4.00	m	7	69.0	3.97	
8	45.6	1.56	ovlp.	8	46.5	1.53	ovlp.	8	45.7	1.53	ovlp.	8	45.9	1.56	
9	66.5	4.09	ovlp.	9	66.6	4.03-4.09	ovlp.	9	66.6	4.03-4.09	ovlp.	9	66.4	4.09	
10	45.6	1.58	ovlp.	10	N.A.			10	N.A.						
11	66.5	4.07	ovlp.	11	N.A.			11	N.A.						
12	46.5	1.58	ovlp.	12	N.A.			12	N.A.						
13	66.5	4.07	ovlp.	13	N.A.			13	N.A.						
14	46.5	1.58	ovlp.	14	N.A.			14	N.A.			10	46.8	1.54	
15	66.5	4.07	ovlp.	15	66.6	4.03-4.09	ovlp.	15	66.6	4.03-4.09	ovlp.	11	66.5	4.06	
16	39.7	1.48	ovlp.	16	39.4	1.35-1.55	ovlp.	16	40.8	1.52		12	39.9	1.48	
17	69.7	3.8	m	17	69.6	3.80	m	17	70.6	3.93		13	69.8	3.80	
18	43.0	1.87	m	18	43.1	1.89	s	18	45.7	1.72	ovlp.	14	43.2	1.87	
19	80.7	3.81	d (8.3)	19	81.2	3.76	d (8.7)	19	76.0	4.00		15	80.6	3.83	
20	138.9			20	138.9	-		20	134.8	5.56	dd (7.4, 15.5)	16	138.9	-	

Table S18 continued

**6**, structural assignment: Fig. S54				**8**, structural assignment: Fig. S72				**9**, structural assignment: Fig. S72				**10**, structural assignment: Fig. S80			
**ID**	**C**	**H**	**Multi-plicity**	**ID**	**C**	**H**	**Multi-plicity**	**ID**	**C**	**H**	**Multi-plicity**	**ID**	**C**	**H**	**Multi-plicity**
21	125.0	5.48	ovlp.	21	125.3	5.48	m	21	129.7	5.68	dt (7.1, 15.5)	17	124.8	5.48	
22	36.6	2.13	ddd (6.9, 6.9, 14.4)	22	36.6	2.11	ovlp.	22	41.5	2.23		18	36.7	2.14	
		2.3	ovlp.			2.30	ovlp.							2.30	
23	72.4	3.6	ovlp.	23	72.6	3.60	m	23	72.3	3.60	m	19	72.3	3.60	
24	38.0	1.45	ovlp.	24	38.1	1.35-1.55	ovlp.	24	38.1	1.35-1.55	ovlp.	20	37.6	1.47	
25	27.2	1.46	ovlp.	25	N.A.			25	N.A.			21	N.A.		
26 or 28	38.5	1.37	ovlp.	26	N.A.			26	N.A.			22	N.A.		
27	72.4	3.49	m	27	72.4	3.51		27	72.4	3.50		23	72.4	3.49	
26 or 28	38.5	1.37	ovlp.	28	N.A.			28	N.A.			24	38.4	1.38	
30 or 32	38.5	1.39	ovlp.	29	N.A.			29	N.A.			25	N.A.		
		1.49	ovlp.	30	N.A.			30	N.A.			26	N.A.		
31	72.4	3.54	m	31	N.A.			31	N.A.			27	72.3	3.53	
30 or 32	38.5	1.39	ovlp.									28	38.4	1.40	
		1.49	ovlp.									29	N.A.		
33	N.A.														
34	N.A.											30	N.A.		
35	29.8	1.32	ovlp.									31	N.A.		

Table S18 continued

**6**, structural assignment: Fig. S54				**8**, structural assignment: Fig. S72				**9**, structural assignment: Fig. S72				**10**, structural assignment: Fig. S80			
**ID**	**C**	**H**	**Multi-plicity**	**ID**	**C**	**H**	**Multi-plicity**	**ID**	**C**	**H**	**Multi-plicity**	**ID**	**C**	**H**	**Multi-plicity**
36	30.8	1.29	ovlp.	32	30.5	1.28	ovlp.	32	30.6	1.29	ovlp.	32	30.4	1.29	
37	33.6	1.95	m	33	33.6	1.95	m	33	33.6	1.96	m	33	35.5	1.94	
38	134.7	5.44	ovlp.	34	134.8	5.43	dt (15.3)	34	134.9	5.45	dt (15.2)	34	134.7	5.44	dt (15.2)
39	126.8	5.33	ddd (7.0, 7.0, 15.2)	35	126.9	5.35	dt (15.3)	35	126.8	5.35	dt (15.2)	35	126.9	5.33	dt (15.2)
40	36.8	2.3	ovlp.	36	36.8	2.30	m	36	36.7	2.31	m	36	36.7	2.29	
		2.41	ovlp.			2.39	m			2.40	m			2.40	
41	76.2	5.09	ddd (3.5, 6.4, 8.8)	37	76.3	5.09	m	37	76.2	5.08	m	37	76.3	5.09	
42	43.0	1.71	m	38	43	1.71		38	43.0	1.72		38	43.1	1.70	
43	72.4	3.6	ovlp.	39	72.6	3.60	ovlp.	39	72.4	3.60	ovlp.	39	72.4	3.60	
44	38.3	1.46	m	40	N.A.			40	N.A.			40	N.A.		
45	N.A.			41	N.A.			41	N.A.			41	N.A.		
46	30.5	1.31	ovlp.	42	N.A.			42	N.A.			42	N.A.		
47	26.9	1.31	ovlp.	43	26.9	1.41	ovlp.	43	26.9	1.41	ovlp.	43	26.7	1.41	
		1.41	ovlp.												
48	40.0	1.43	ovlp.	44	40.2	1.33-1.45		44	40.2	1.33-1.45		44	40.2	1.43	
49	68.5	3.71	m	45	68.6	3.71	m	45	68.6	3.71	m	45	68.5	3.70	
50	23.5	1.14	d (6.2)	46	23.5	1.14	d (6.2)	46	23.5	1.14	d (6.2)	46	23.5	1.14	d (6.2)
51	12.6	1.83, brs		47	12.6	1.83	s	47	12.6	1.83	s	47	12.7	1.83	s
52	10.6	0.97	d (6.8)	48	10.8	0.99	d (6.8)	48	11.6	0.95	d (6.9)	48	10.7	0.96	d (6.9)
53	11.7	1.64	brs	49	11.5	1.65	s					49	11.8	1.64	s
54	9.9	0.95	d (6.9)	50	9.9	0.95	d (7.0)	49	9.9	0.94	d (7.0)	50	9.9	0.94	d (7.0)

Table S18 continued

**6**, structural assignment: Fig. S54				**8**, structural assignment: Fig. S72				**9**, structural assignment: Fig. S72				**10**, structural assignment: Fig. S80			
**ID**	**C**	**H**	**Multi-plicity**	**ID**	**C**	**H**	**Multi-plicity**	**ID**	**C**	**H**	**Multi-plicity**	**ID**	**C**	**H**	**Multi-plicity**
				unassigned C-OH				unassigned C-OH				unassigned C-OH			
					66.6	4.03-4.09			66.6	4.03-4.09			66.6	4.03-4.09	
				unassigned CH_2				more than 10 unassigned CH_2				unassigned CH_2			
					46.3	1.43-1.55			25.0-46.8	1.22-1.56			46.3	1.43-1.55	
					46.3	1.43-1.55							46.3	1.43-1.55	
					45.4	1.43-1.55							45.4	1.43-1.55	
					38.7	1.35-1.55							38.7	1.35-1.55	
					38.3	1.35-1.55							38.3	1.35-1.55	
					36	1.48							36.0	1.48	
					30.8	1.22-1.40							30.8	1.22-1.40	
					30.8	1.22-1.40							30.8	1.22-1.40	
					30.1	1.22-1.40							30.1	1.22-1.40	
					27.2	1.31							27.2	1.31	
					26.9	1.37							26.9	1.37	
					23.2	1.37/1.62							23.2	1.37/1.62	

Table S18 continued

**11**, structural assignment Fig. S80				**12**, structural assignment Fig. S82				**13**				**14**, structural assignment Fig. S94			
**ID**	**C**	**H**	**Multi-plicity**	**ID**	**C**	**H**	**Multi-plicity**	**ID**	**C**	**H**	**Multi-plicity**	**ID**	**C**	**H**	**Multi-plicity**
1	168.9	-		1	168.9	-		Not isolated				1	168.7		
2	117.0	5.81	d (15.7)	2	117.0	5.82	d (15.7)					2	117.1	5.81	d (15.7)
3	150.9	7.32	d (15.7)	3	150.9	7.35	d (15.7)								
4	135.5	-		4	135.5	-									
5	139.9	6.06	t (7.1)	5	140.0	6.05	t (7.2)								
6	38.5	2.42		6	38.8	2.41									
7	69.0	3.97		7	69.0	3.92									
8	45.9	1.56		8	39.8	1.46									
9	66.4	4.09		9	69.8	3.80									
10	46.8	1.54													
11	66.5	4.06													
12	39.9	1.48													
13	70.8	3.91													
14	45.7	1.70		10	43.2	1.86									
15	75.6	4.08		11	80.3	3.85	d (8.0)					3	150.5	7.32	d (15.7)
16	135.1	5.56	dd (7.1, 15.5)	12	138.9	-						4	135.7	-	
17	129.4	5.67	dt (7.8, 15.5)	13	124.6	5.48						5	139.0	6.03	t (7.5)
18	41.5	2.22		14	36.6	2.16						6	37.5	2.41	
						2.30								2.47	
19	72.3	3.60		15	72.4	3.60						7	71.8	3.71	
20	37.6	1.47		16	N.A.							8	N.A.		

Table S18 continued

**11**, structural assignment Fig. S80				**12**, structural assignment Fig. S82				**13**				**14**, structural assignment Fig. S94			
**ID**	**C**	**H**	**Multi-plicity**	**ID**	**C**	**H**	**Multi-plicity**	**ID**	**C**	**H**	**Multi-plicity**	**ID**	**C**	**H**	**Multi-plicity**
21	N.A.			17	N.A.			Not isolated				9	N.A.		
22	N.A.			18	N.A.							10	N.A.		
23	72.4	3.49		19	71.3	3.50						11	71.6	3.54	
24	38.4	1.38		20	38.3	1.39						12	38.0	1.46	
25	N.A.			21	N.A.							13	N.A.		
26	N.A.			22	N.A.							14	N.A.		
27	72.3	3.53		23	72.0	3.56						15	71.4	3.58	
28	38.4	1.40		24	38.1	1.42						16	37.7	1.45	
29	N.A.			25	N.A.							17	N.A.		
30	N.A.			26	N.A.							18	N.A.		
31	N.A.			27	N.A.							19	N.A.		
32	30.4	1.29		28	30.2	1.30						20	30.3	1.31	
33	35.5	1.94		29	33.4	1.96						21	33.5	1.96	
34	134.7	5.44	dt (15.2)	30	134.6	5.44	dt (15.3)					22	134.7	5.46	dt (15.3)
35	126.9	5.33	dt (15.2)	31	126.9	5.34	dt (15.3)					23	126.7	5.35	dt (15.3)
36	36.7	2.29<br>2.40		32	36.7	2.16<br>2.30						24	36.6	2.33<br>2.41	
37	76.3	5.09		33	76.4	5.09						25	76.6	5.08	
38	43.1	1.70		34	43.0	1.71						26	42.9	1.72	
39	72.4	3.60		35	72.4	3.58						27	72.2	3.62	
40	N.A.			36	N.A.							28	36.1	1.47	
41	N.A.			37	N.A.							29	27.3	1.33	
42	N.A.			38	N.A.							30			
43	26.7	1.41		39	26.9	1.32						31			
44	40.2	1.43		40	40.2	1.42						32	40.2	1.43	

Table S18 continued

**11**, structural assignment Fig. S80				**12**, structural assignment Fig. S82				**13**				**14**, structural assignment Fig. S94			
**ID**	**C**	**H**	**Multi-plicity**	**ID**	**C**	**H**	**Multi-plicity**	**ID**	**C**	**H**	**Multi-plicity**	**ID**	**C**	**H**	**Multi-plicity**
45	68.5	3.70		41	68.5	3.71			Not isolated			33	68.5	3.71	
46	23.5	1.14	d (6.2)	42	23.5	1.14	d (6.3)					34	23.5	1.14	d (6.3)
47	12.7	1.83	s	43	12.7	1.83	s					35	12.5	1.83	
48	11.5	0.93	d (6.9)	44	10.7	0.95	d (6.9)								
				45	12.0	1.64	s								
49	9.9	0.94	d (7.0)	46	9.9	0.94	d (6.9)					36	9.9	0.93	d (6.9)
	unassigned CH_2				unassigned CH_2								unassigned CH_2		
					38.4	1.47							37.3	1.47	
	11	ovlp.			37.9	1.41							37.1	1.52	
					37.5	1.57							37.0	1.40	
					36.0	1.47							30.8	1.28-1.41	
					30.8	1.34							30.1	1.28	
					30.5	1.31							29.8	1.28	
					29.8	1.25							26.9	1.43	
					26.6	1.41							26.3	1.60	
					22.8	1.40							22.7	1.60	
					22.8	1.56							21.9	1.39	

Table S18 continued

**15**				**16**, structural assignment Fig. S101				**17**				**18**, structural assignment Fig. S108			
**ID**	**C**	**H**	**Multi-plicity**	**ID**	**C**	**H**	**Multi-plicity**	**ID**	**C**	**H**	**Multi-plicity**	**ID**	**C**	**H**	**Multi-plicity**
Not detected				1	168.9	-		Not isolated				1	169.0	-	
				2	117.1	5.80	d (15.7)					2	117.3	5.81	d (15.7)
				3	150.8	7.31	d (15.7)								
				4	135.7	-									
				5	139.5	6.05	t (7.5)								
				6	38.2	2.44									
				7	69.0	3.97									
				8	45.6	1.58									
				9	66.6	4.07									
				10	46.2	1.54									
				11	66.9	4.05									
				12	39.3	1.51									
				13	69.6	3.79									
				14	43.2	1.88									
				15	81.0	3.78						3	150.6	7.32	d (15.7)
				16	138.6	-						4	135.7	-	
				17	125.2	5.47	t (7.2)					5	138.5	6.03	t (7.4)
				18	36.7	2.12						6	36.8	2.38	
						2.31								2.56	
				19	72.4	3.60						7	71.4	3.81	
				20	38.0	1.44						8	36.0	1.46	
				21	N.A.							9			
				22	N.A.							10			
				23	72.5	3.47						11			
				24	38.6	1.37						12			
				25	N.A.							13			

Table S18 continued

15				16, structural assignment Fig. S101				17				18, structural assignment Fig. S108			
**ID**	**C**	**H**	**Multi-plicity**	**ID**	**C**	**H**	**Multi-plicity**	**ID**	**C**	**H**	**Multi-plicity**	**ID**	**C**	**H**	**Multi-plicity**
Not detected				26	N.A.			Not isolated				14			
				27	N.A.							15			
				28	30.8	1.27						16	30.6	1.24	
				29	33.7	1.94						17	33.6	1.93	
				30	134.8	5.43	dt (15.3)					18	134.7	5.42	dt (15.2)
				31	126.8	5.33	dt (15.3)					19	127.0	5.31	dt (15.2)
				32	36.9	2.29						20	37.1	2.26	
				33	76.2	5.10						21	76.1	5.12	
				34	43.2	1.70						22	43.5	1.69	
				35	72.4	3.60						23	72.5	3.60	
				36	N.A.							24			
				37	N.A.							25			
				38	N.A.							26			
				39	N.A.							27			
				40	40.1	1.43						28	40.1	1.43	
				41	68.5	3.71						29	68.6	3.71	
				42	23.5	1.14	d (6.2)					30	23.5	1.14	d (6.2)
				43	12.7	1.83	s					31	12.6	1.83	s
				44	10.6	0.98	d (6.9)								
				45	11.5	1.65	s								
				46	9.9	0.94	d (6.9)					32	10.0	0.95	d (6.9)

Table S18 continued

**15**				**16**, structural assignment Fig. S101				**17**				**18**, structural assignment Fig. S108			
**ID**	**C**	**H**	**Multi-plicity**	**ID**	**C**	**H**	**Multi-plicity**	**ID**	**C**	**H**	**Multi-plicity**	**ID**	**C**	**H**	**Multi-plicity**
Not detected				unassigned CH_2				Not isolated				unassigned CH_2			
					38.6	1.33-1.52									
					36.0	1.48									
					30.9	1.20-1.41									
					30.7	1.20-1.41									
					30.4	1.20-1.41									
					37.2	1.42-1.56									
					37.1	1.42-1.56									
					36.9	1.42-1.56									
					23.3	1.63									

Table S18 continued

19				**20**, structural assignment Fig. S105				**21**, structural assignment Fig. S105			
**ID**	**C**	**H**	**Multi-plicity**	**ID**	**C**	**H**	**Multi-plicity**	**ID**	**C**	**H**	**Multi-plicity**
Not detected				1	169.0	-		1	169.0	-	
				2	116.8	5.81	d (15.7)	2	116.8	5.81	d (15.7)
				3	151.1	7.33	d (15.7)	3	151.1	7.33	d (15.7)
				4	135.4	-		4	135.4	-	
				5	140.6	6.08	t (7.4)	5	140.6	6.08	t (7.4)
				6	38.7	2.38		6	38.7	2.38	
				7	68.9	3.91		7	68.9	3.91	
				8	39.6	1.47		8	39.6	1.47	
				9	69.4	3.80		9	70.7	3.92	
				10	43.3	1.88		10	45.9	1.70	
				11	81.0	3.79		11	75.8	4.04	
				12	139.0	-		12	135.2	5.55	dd (7.2, 15.5)
				13	125.0	5.48		13	129.4	5.68	dt (6.7, 15.5)
				14	36.8	2.10		14	41.5	2.23	
						2.30					
				15	72.4	3.60		15	72.2	3.62	
				16				16			
				17				17			
				18				18			
				19	72.4	3.50		19			
				20	38.2	1.37		20			

Table S18 continued

19				**20**, structural assignment Fig. S105				**21**, structural assignment Fig. S105			
**ID**	**C**	**H**	**Multi-plicity**	**ID**	**C**	**H**	**Multi-plicity**	**ID**	**C**	**H**	**Multi-plicity**
	Not detected			21				21			
				22				22			
				23				23			
				24	30.1	1.28		24	30.1	1.28	
				25	33.4	1.94		25	33.4	1.94	
				26	134.5	5.43	dt (15.2)	26	134.5	5.43	dt (15.2)
				27	127.0	5.32	dt (15.2)	27	127.0	5.32	dt (15.2)
				28	36.8	2.28		28	36.8	2.28	
				29	76.3	5.10		29	76.3	5.10	
				30	43.2	1.69		30	43.2	1.69	
				31	72.3	3.59		31	72.3	3.59	
				32				32			
				33				33			
				34				34			
				35	26.8	1.41		35	26.8	1.41	
				36	40.2	1.43		36	40.2	1.43	
				37	68.5	3.71		37	68.5	3.71	
				38	23.5	1.14	d (6.2)	38	23.5	1.14	d (6.2)
				39	12.6	1.82	s	39	12.6	1.82	s
				40	10.7	0.98	d (6.9)	40	11.5	0.93	d (6.9)
				41	11.6	1.64	s				
				42	9.9	0.94	d (6.9)	41	9.9	0.94	d (6.9)

Table S18 continued

**19**				**20**, structural assignment Fig. S105				**21**, structural assignment Fig. S105			
**ID**	**C**	**H**	**Multi-plicity**	**ID**	**C**	**H**	**Multi-plicity**	**ID**	**C**	**H**	**Multi-plicity**
Not detected				unassigned CH_2							
					41	9.9	0.94				
					38.3	1.47					
					38.0	1.39					
					37.7	1.53					
					36.0	1.48					
					30.8	1.29					
					30.4	1.29					
					30.2	1.26					
					29.8	1.25					
					29.6	1.25					
					27.2	1.47					
					26.5	1.40					
					23.0	1.36-1.54					

Table S19 Overview of characterization methods used for the characterization of compounds **1**-**21**.

Compound	MS	MS/MS	1H-NMR	13C-NMR	COSY	HMBC	HSQC
**1**	**X**						
**2**	**X**		**X**	**X**	**X**	**X**	**X**
**3**	**X**		**X**	**X**	**X**	**X**	**X**
**4**	**X**						
**5**	**X**						
**6**	**X**	**X**					
**7**	**X**	**X**					
**8**	**X**	**X**	**X**	**X**	**X**	**X**	**X**
**9**	**X**	**X**	**X**	**X**	**X**	**X**	**X**
**10**	**X**	**X**	**X**	**X**	**X**	**X**	**X**
**11**	**X**	**X**	**X**	**X**	**X**	**X**	**X**
**12**	**X**	**X**	**X**	**X**	**X**	**X**	**X**
**13**	**X**	**X**					
**14**	**X**	**X**	**X**	**X**	**X**	**X**	**X**
**15**	**X**	**X**					
**16**	**X**	**X**	**X**	**X**	**X**	**X**	**X**
**17**	**X**	**X**					
**18**	**X**	**X**	**X**	**X**	**X**	**X**	**X**
**19**	**X**	**X**					
**20**	**X**	**X**	**X**	**X**	**X**	**X**	**X**
**21**	**X**	**X**	**X**	**X**	**X**	**X**	**X**

References and notes

1. D. J. Newman, G. M. Cragg, Natural Products as Sources of New Drugs over the Nearly Four Decades from 01/1981 to 09/2019. *J. Nat. Prod.* **83**, 770–803 (2020). doi:10.1021/acs.jnatprod.9b01285 Medline

2. J. Staunton, K. J. Weissman, Polyketide biosynthesis: A millennium review. *Nat. Prod. Rep.* **18**, 380–416 (2001). doi:10.1039/a909079g Medline

3. T. Robbins, Y.-C. Liu, D. E. Cane, C. Khosla, Structure and mechanism of assembly line polyketide synthases. *Curr. Opin. Struct. Biol.* **41**, 10–18 (2016). doi:10.1016/j.sbi.2016.05.009 Medline

4. M. Grininger, Enzymology of assembly line synthesis by modular polyketide synthases. *Nat. Chem. Biol.* **19**, 401–415 (2023). doi:10.1038/s41589-023-01277-7 Medline

5. S. Donadio, M. J. Staver, J. B. McAlpine, S. J. Swanson, L. Katz, Modular organization of genes required for complex polyketide biosynthesis. *Science* **252**, 675–679 (1991). doi:10.1126/science.2024119 Medline

6. J.-M. Massicard, C. Soligot, K. J. Weissman, C. Jacob, Manipulating polyketide stereochemistry by exchange of polyketide synthase modules. *Chem. Commun.* **56**, 12749–12752 (2020). doi:10.1039/D0CC05068G Medline

7. G. Zhai, Y. Zhu, G. Sun, F. Zhou, Y. Sun, Z. Hong, C. Dong, P. F. Leadlay, K. Hong, Z. Deng, F. Zhou, Y. Sun, Insights into azalomycin F assembly-line contribute to evolution-guided polyketide synthase engineering and identification of intermodular recognition. *Nat. Commun.* **14**, 612 (2023). doi:10.1038/s41467-023-36213-9 Medline

8. M. Klaus, M. Grininger, Engineering strategies for rational polyketide synthase design. *Nat. Prod. Rep.* **35**, 1070–1081 (2018). doi:10.1039/C8NP00030A Medline

9. K. J. Weissman, Genetic engineering of modular PKSs: From combinatorial biosynthesis to synthetic biology. *Nat. Prod. Rep.* **33**, 203–230 (2016). doi:10.1039/C5NP00109A Medline

10. A. Nivina, K. P. Yuet, J. Hsu, C. Khosla, Evolution and Diversity of Assembly-Line Polyketide Synthases. *Chem. Rev.* **119**, 12524–12547 (2019). doi:10.1021/acs.chemrev.9b00525 Medline

11. E. J. N. Helfrich, J. Piel, Biosynthesis of polyketides by trans-AT polyketide synthases. *Nat. Prod. Rep.* **33**, 231–316 (2016). doi:10.1039/C5NP00125K Medline

12. L. Gu, B. Wang, A. Kulkarni, T. W. Geders, R. V. Grindberg, L. Gerwick, K. Håkansson, P. Wipf, J. L. Smith, W. H. Gerwick, D. H. Sherman, Metamorphic enzyme assembly in polyketide diversification. *Nature* **459**, 731–735 (2009). doi:10.1038/nature07870 Medline

13. F. Hemmerling, R. A. Meoded, A. E. Fraley, H. A. Minas, C. L. Dieterich, M. Rust, R. Ueoka, K. Jensen, E. J. N. Helfrich, C. Bergande, M. Biedermann, N. Magnus, B. Piechulla, J. Piel, Modular Halogenation, α-Hydroxylation, and Acylation by a Remarkably Versatile Polyketide Synthase. *Angew. Chem. Int. Ed.* **61**, e202116614 (2022). doi:10.1002/anie.202116614 Medline

14. M. Jenner, S. Frank, A. Kampa, C. Kohlhaas, P. Pöplau, G. S. Briggs, J. Piel, N. J. Oldham, Substrate specificity in ketosynthase domains from trans-AT polyketide synthases. *Angew. Chem. Int. Ed.* **52**, 1143–1147 (2013). doi:10.1002/anie.201207690 Medline

15. T. Bretschneider, J. B. Heim, D. Heine, R. Winkler, B. Busch, B. Kusebauch, T. Stehle, G. Zocher, C. Hertweck, Vinylogous chain branching catalysed by a dedicated polyketide synthase module. *Nature* **502**, 124–128 (2013). doi:10.1038/nature12588 Medline

16. S. Meng, A. D. Steele, W. Yan, G. Pan, E. Kalkreuter, Y.-C. Liu, Z. Xu, B. Shen, Thiocysteine lyases as polyketide synthase domains installing hydropersulfide into natural products and a hydropersulfide methyltransferase. *Nat. Commun.* **12**, 5672 (2021). doi:10.1038/s41467-021-25798-8 Medline

17. R. A. Meoded, R. Ueoka, E. J. N. Helfrich, K. Jensen, N. Magnus, B. Piechulla, J. Piel, A Polyketide Synthase Component for Oxygen Insertion into Polyketide Backbones. *Angew. Chem. Int. Ed.* **57**, 11644–11648 (2018). doi:10.1002/anie.201805363 Medline

18. R. Ueoka, R. A. Meoded, A. Gran-Scheuch, A. Bhushan, M. W. Fraaije, J. Piel, Genome Mining of Oxidation Modules in trans-Acyltransferase Polyketide Synthases Reveals a Culturable Source for Lobatamides. *Angew. Chem. Int. Ed.* **59**, 7761–7765 (2020). doi:10.1002/anie.201916005 Medline

19. A. E. Fraley, C. L. Dieterich, M. F. J. Mabesoone, H. A. Minas, R. A. Meoded, F. Hemmerling, J. Piel, Structure of a Promiscuous Thioesterase Domain Responsible for Branching Acylation in Polyketide Biosynthesis. *Angew. Chem. Int. Ed.* **61**, e202206385 (2022). doi:10.1002/anie.202206385 Medline

20. E. J. N. Helfrich, R. Ueoka, A. Dolev, M. Rust, R. A. Meoded, A. Bhushan, G. Califano, R. Costa, M. Gugger, C. Steinbeck, P. Moreno, J. Piel, Automated structure prediction of trans-acyltransferase polyketide synthase products. *Nat. Chem. Biol.* **15**, 813–821 (2019). doi:10.1038/s41589-019-0313-7 Medline

21. K. M. Fisch, C. Gurgui, N. Heycke, S. A. van der Sar, S. A. Anderson, V. L. Webb, S. Taudien, M. Platzer, B. K. Rubio, S. J. Robinson, P. Crews, J. Piel, Polyketide assembly lines of uncultivated sponge symbionts from structure-based gene targeting. *Nat. Chem. Biol.* **5**, 494–501 (2009). doi:10.1038/nchembio.176 Medline

22. R. Ueoka, A. R. Uria, S. Reiter, T. Mori, P. Karbaum, E. E. Peters, E. J. N. Helfrich, B. I. Morinaka, M. Gugger, H. Takeyama, S. Matsunaga, J. Piel, Metabolic and evolutionary origin of actin-binding polyketides from diverse organisms. *Nat. Chem. Biol.* **11**, 705–712 (2015). doi:10.1038/nchembio.1870 Medline

23. T. Nguyen, K. Ishida, H. Jenke-Kodama, E. Dittmann, C. Gurgui, T. Hochmuth, S. Taudien, M. Platzer, C. Hertweck, J. Piel, Exploiting the mosaic structure of trans-acyltransferase polyketide synthases for natural product discovery and pathway dissection. *Nat. Biotechnol.* **26**, 225–233 (2008). doi:10.1038/nbt1379 Medline

24. Z.-J. Wang, X. Liu, H. Zhou, Y. Liu, Q. Tu, L. Huo, F. Yan, R. Müller, Y. Zhang, X. Xu, Engineered Biosynthesis of Complex Disorazol Polyketides in a Streamlined *Burkholderia thailandensis*. *ACS Synth. Biol.* **12**, 971–977 (2023). doi:10.1021/acssynbio.2c00610 Medline

25. M. Till, P. R. Race, Progress challenges and opportunities for the re-engineering of trans-AT polyketide synthases. *Biotechnol. Lett.* **36**, 877–888 (2014). doi:10.1007/s10529-013-1449-2 Medline

26. E. J. N. Helfrich, R. Ueoka, M. G. Chevrette, F. Hemmerling, X. Lu, S. Leopold-Messer, H. A. Minas, A. Y. Burch, S. E. Lindow, J. Piel, M. H. Medema, Evolution of combinatorial diversity in trans-acyltransferase polyketide synthase assembly lines across bacteria. *Nat. Commun.* **12**, 1422 (2021). doi:10.1038/s41467-021-21163-x Medline

27. Y. Sugimoto, K. Ishida, N. Traitcheva, B. Busch, H.-M. Dahse, C. Hertweck, Freedom and Constraint in Engineered Noncolinear Polyketide Assembly Lines, Freedom and constraint in engineered noncolinear polyketide assembly lines. *Chem. Biol.* **22**, 229–240 (2015). doi:10.1016/j.chembiol.2014.12.014 Medline

28. H. Peng, K. Ishida, Y. Sugimoto, H. Jenke-Kodama, C. Hertweck, Emulating evolutionary processes to morph aureothin-type modular polyketide synthases and associated oxygenases. *Nat. Commun.* **10**, 3918 (2019). doi:10.1038/s41467-019-11896-1 Medline

29. A. M. Albertini, T. Caramori, F. Scoffone, C. Scotti, A. Galizzi, Sequence around the 159 degree region of the Bacillus subtilis genome: The pksX locus spans 33.6 kb. *Microbiology (Reading)* **141**, 299–309 (1995). Medline

30. R. A. Butcher, F. C. Schroeder, M. A. Fischbach, P. D. Straight, R. Kolter, C. T. Walsh, J. Clardy, The identification of bacillaene, the product of the PksX megacomplex in Bacillus subtilis. *Proc. Natl. Acad. Sci. U.S.A.* **104**, 1506–1509 (2007). doi:10.1073/pnas.0610503104 Medline

31. S. W. W. Lockless, R. Ranganathan, Evolutionarily conserved pathways of energetic connectivity in protein families. *Science* **286**, 295–299 (1999). doi:10.1126/science.286.5438.295 Medline

32. O. Rivoire, K. A. Reynolds, R. Ranganathan, Evolution-Based Functional Decomposition of Proteins. *PLOS Comput. Biol.* **12**, e1004817 (2016). doi:10.1371/journal.pcbi.1004817 Medline

33. D. C. Gay, D. T. Wagner, J. L. Meinke, C. E. Zogzas, G. R. Gay, A. T. Keatinge-Clay, The LINKS motif zippers trans-acyltransferase polyketide synthase assembly lines into a biosynthetic megacomplex. *J. Struct. Biol.* **193**, 196–205 (2016). doi:10.1016/j.jsb.2015.12.011 Medline

34. S. Kosol, M. Jenner, J. R. Lewandowski, G. L. Challis, Protein-protein interactions in trans-AT polyketide synthases. *Nat. Prod. Rep.* **35**, 1097–1109 (2018). doi:10.1039/C8NP00066B Medline

35. A. Miyanaga, R. Ouchi, F. Ishikawa, E. Goto, G. Tanabe, F. Kudo, T. Eguchi, Structural Basis of Protein-Protein Interactions between a trans-Acting Acyltransferase and Acyl Carrier Protein in Polyketide Disorazole Biosynthesis. *J. Am. Chem. Soc.* **140**, 7970–7978 (2018). doi:10.1021/jacs.8b04162 Medline

36. Y. U. Tittes, D. A. Herbst, S. F. X. Martin, H. Munoz-Hernandez, R. P. Jakob, T. Maier, The structure of a polyketide synthase bimodule core. *Sci. Adv.* **8**, eabo6918 (2022). doi:10.1126/sciadv.abo6918 Medline

37. K. Blin, S. Shaw, S. A. Kautsar, M. H. Medema, T. Weber, The antiSMASH database version 3: Increased taxonomic coverage and new query features for modular enzymes. *Nucleic Acids Res.* **49** (D1), D639–D643 (2021). doi:10.1093/nar/gkaa978 Medline

38. D. C. Gay, G. Gay, A. J. Axelrod, M. Jenner, C. Kohlhaas, A. Kampa, N. J. Oldham, J. Piel, A. T. Keatinge-Clay, A close look at a ketosynthase from a trans-acyltransferase modular polyketide synthase. *Structure* **22**, 444–451 (2014). doi:10.1016/j.str.2013.12.016 Medline

39. E. M. Musiol, T. Weber, Discrete acyltransferases involved in polyketide biosynthesis. *MedChemComm* **3**, 871 (2012). doi:10.1039/c2md20048a

40. Z. D. Aron, P. D. Fortin, C. T. Calderone, C. T. Walsh, FenF: Servicing the Mycosubtilin synthetase assembly line in trans. *ChemBioChem* **8**, 613–616 (2007). doi:10.1002/cbic.200600575 Medline

41. T. Teşileanu, L. J. Colwell, S. Leibler, Protein sectors: Statistical coupling analysis versus conservation. *PLOS Comput. Biol.* **11**, e1004091 (2015). doi:10.1371/journal.pcbi.1004091 Medline

42. R. N. McLaughlin Jr., F. J. Poelwijk, A. Raman, W. S. Gosal, R. Ranganathan, The spatial architecture of protein function and adaptation. *Nature* **491**, 138–142 (2012). doi:10.1038/nature11500 Medline

43. S. Yuzawa, K. Deng, G. Wang, E. E. K. Baidoo, T. R. Northen, P. D. Adams, L. Katz, J. D. Keasling, Comprehensive in Vitro Analysis of Acyltransferase Domain Exchanges in Modular Polyketide Synthases and Its Application for Short-Chain Ketone Production. *ACS Synth. Biol.* **6**, 139–147 (2017). doi:10.1021/acssynbio.6b00176 Medline

44. E. Englund, M. Schmidt, A. A. Nava, A. Lechner, K. Deng, R. Jocic, Y. Lin, J. Roberts, V. T. Benites, R. Kakumanu, J. W. Gin, Y. Chen, Y. Liu, C. J. Petzold, E. E. K. Baidoo, T. R. Northen, P. D. Adams, L. Katz, S. Yuzawa, J. D. Keasling, Expanding Extender Substrate Selection for Unnatural Polyketide Biosynthesis by Acyltransferase Domain Exchange within a Modular Polyketide Synthase. *J. Am. Chem. Soc.* **145**, 8822–8832 (2023). doi:10.1021/jacs.2c11027 Medline

45. K. A. J. Bozhüyük, A. Linck, A. Tietze, J. Kranz, F. Wesche, S. Nowak, F. Fleischhacker, Y.-N. Shi, P. Grün, H. B. Bode, Modification and de novo design of non-ribosomal peptide synthetases using specific assembly points within condensation domains. *Nat. Chem.* **11**, 653–661 (2019). doi:10.1038/s41557-019-0276-z Medline

46. G.-L. Tang, Y.-Q. Cheng, B. Shen, Leinamycin biosynthesis revealing unprecedented architectural complexity for a hybrid polyketide synthase and nonribosomal peptide synthetase. *Chem. Biol.* **11**, 33–45 (2004). doi:10.1016/j.chembiol.2003.12.014 Medline

47. A. E. Fraley, M. Dell, M. Schmalhofer, R. A. Meoded, C. Bergande, M. Groll, J. Piel, Heterocomplex structure of a polyketide synthase component involved in modular backbone halogenation. *Structure* **31**, 565–572.e4 (2023). doi:10.1016/j.str.2023.02.010 Medline

48. J. Moldenhauer, X.-H. H. Chen, R. Borriss, J. Piel, Biosynthesis of the antibiotic bacillaene, the product of a giant polyketide synthase complex of the trans-AT family. *Angew. Chem. Int. Ed.* **46**, 8195–8197 (2007). doi:10.1002/anie.200703386 Medline

49. C. Kohlhaas, M. Jenner, A. Kampa, G. S. Briggs, J. P. Afonso, J. Piel, N. J. Oldham, Amino acid-accepting ketosynthase domain from a trans-AT polyketide synthase exhibits high selectivity for predicted intermediate. *Chem. Sci.* **4**, 3212 (2013). doi:10.1039/c3sc50540e

50. J. R. Lohman, M. Ma, J. Osipiuk, B. Nocek, Y. Kim, C. Chang, M. Cuff, J. Mack, L. Bigelow, H. Li, M. Endres, G. Babnigg, A. Joachimiak, G. N. Phillips Jr., B. Shen, Structural and evolutionary relationships of "AT-less" type I polyketide synthase ketosynthases. *Proc. Natl. Acad. Sci. U.S.A.* **112**, 12693–12698 (2015). doi:10.1073/pnas.1515460112 Medline

51. R. Ueoka, A. Bhushan, S. I. Probst, W. M. Bray, R. S. Lokey, R. G. Linington, J. Piel, Genome-Based Identification of a Plant-Associated Marine Bacterium as a Rich Natural Product Source. *Angew. Chem. Int. Ed.* **57**, 14519–14523 (2018). doi:10.1002/anie.201805673 Medline

52. M. F. J. Mabesoone, S. Leopold-Messer, H. A. Minas, C. Chepkirui, P. Chawengrum, S. Reiter, R. A. Meoded, S. Wolff, F. Genz, N. Magnus, B. Piechulla, A. S. Walker, J. Piel, Evolution-guided engineering of trans-acyl transferase polyketide synthases, version 1, Zenodo (2023); https://doi.org/10.5281/zenodo.8146703.

53. R. C. Edgar, MUSCLE: A multiple sequence alignment method with reduced time and space complexity. *BMC Bioinformatics* **5**, 113 (2004). doi:10.1186/1471-2105-5-113 Medline

54. J. Jumper, R. Evans, A. Pritzel, T. Green, M. Figurnov, O. Ronneberger, K. Tunyasuvunakool, R. Bates, A. Žídek, A. Potapenko, A. Bridgland, C. Meyer, S. A. A. Kohl, A. J. Ballard, A. Cowie, B. Romera-Paredes, S. Nikolov, R. Jain, J. Adler, T. Back, S. Petersen, D. Reiman, E. Clancy, M. Zielinski, M. Steinegger, M. Pacholska, T. Berghammer, S. Bodenstein, D. Silver, O. Vinyals, A. W. Senior, K. Kavukcuoglu, P. Kohli, D. Hassabis, Highly accurate protein structure prediction with AlphaFold. *Nature* **596**, 583–589 (2021). doi:10.1038/s41586-021-03819-2 Medline

55. M. A. Konkol, K. M. Blair, D. B. Kearns, Plasmid-encoded ComI inhibits competence in the ancestral 3610 strain of Bacillus subtilis. *J. Bacteriol.* **195**, 4085–4093 (2013). doi:10.1128/JB.00696-13 Medline

56. C. Engler, S. Marillonnet, "Generation of Families of Construct Variants Using Golden Gate Shuffling" in *cDNA Libraries* (Chaofu Lu, 2011), pp. 167–181.

57. T. Cermak, E. L. Doyle, M. Christian, L. Wang, Y. Zhang, C. Schmidt, J. A. Baller, N. V. Somia, A. J. Bogdanove, D. F. Voytas, Efficient design and assembly of custom TALEN and other TAL effector-based constructs for DNA targeting. *Nucleic Acids Res.* **39**, e82–e82 (2011). doi:10.1093/nar/gkr218 Medline

58. A. Loeschcke, A. Markert, S. Wilhelm, A. Wirtz, F. Rosenau, K.-E. Jaeger, T. Drepper, TREX: A universal tool for the transfer and expression of biosynthetic pathways in bacteria. *ACS Synth. Biol.* **2**, 22–33 (2013). doi:10.1021/sb3000657 Medline

59. D. Domik, A. Thürmer, T. Weise, W. Brandt, R. Daniel, B. Piechulla, A Terpene Synthase Is Involved in the Synthesis of the Volatile Organic Compound Sodorifen of Serratia plymuthica 4Rx13. *Front. Microbiol.* **7**, 737 (2016). doi:10.3389/fmicb.2016.00737 Medline

60. M. Rust, E. J. N. Helfrich, M. F. Freeman, P. Nanudorn, C. M. Field, C. Rückert, T. Kündig, M. J. Page, V. L. Webb, J. Kalinowski, S. Sunagawa, J. Piel, A multiproducer

microbiome generates chemical diversity in the marine sponge *Mycale hentscheli*. *Proc. Natl. Acad. Sci. U.S.A.* **117**, 9508–9518 (2020). doi:10.1073/pnas.1919245117 Medline

61. H. Wang, Z. Li, R. Jia, J. Yin, A. Li, L. Xia, Y. Yin, R. Müller, J. Fu, A. F. Stewart, Y. Zhang, ExoCET: Exonuclease in vitro assembly combined with RecET recombination for highly efficient direct DNA cloning from complex genomes. *Nucleic Acids Res.* **46**, e28–e28 (2018). doi:10.1093/nar/gkx1249 Medline

62. D. G. Gibson, L. Young, R.-Y. Chuang, J. C. Venter, C. A. Hutchison 3rd, H. O. Smith, Enzymatic assembly of DNA molecules up to several hundred kilobases. *Nat. Methods* **6**, 343–345 (2009). doi:10.1038/nmeth.1318 Medline

63. S. Thoma, M. Schobert, An improved Escherichia coli donor strain for diparental mating. *FEMS Microbiol. Lett.* **294**, 127–132 (2009). doi:10.1111/j.1574-6968.2009.01556.x Medline

64. M. N. Price, P. S. Dehal, A. P. Arkin, FastTree 2—Approximately maximum-likelihood trees for large alignments. *PLOS ONE* **5**, e9490 (2010). doi:10.1371/journal.pone.0009490 Medline

65. G. E. Crooks, G. Hon, J.-M. Chandonia, S. E. Brenner, WebLogo: A sequence logo generator. *Genome Res.* **14**, 1188–1190 (2004). doi:10.1101/gr.849004 Medline

66. F. Sievers, A. Wilm, D. Dineen, T. J. Gibson, K. Karplus, W. Li, R. Lopez, H. McWilliam, M. Remmert, J. Söding, J. D. Thompson, D. G. Higgins, Fast, scalable generation of high-quality protein multiple sequence alignments using Clustal Omega. *Mol. Syst. Biol.* **7**, 539 (2011). doi:10.1038/msb.2011.75 Medline

67. F. Sievers, D. G. Higgins, "The Clustal Omega Multiple Alignment Package" in *Multiple Sequence Alignment*, vol. 2, Methods in Molecular Biology, K. Katoh, Ed. (Humana, 2021), pp. 3–16.

68. B. Wang, Y. Song, M. Luo, Q. Chen, J. Ma, H. Huang, J. Ju, Biosynthesis of 9-methylstreptimidone involves a new decarboxylative step for polyketide terminal diene formation. *Org. Lett.* **15**, 1278–1281 (2013). doi:10.1021/ol400224n Medline

69. R. Sigrist, H. Luhavaya, S. M. K. McKinnie, A. Ferreira da Silva, I. D. Jurberg, B. S. Moore, L. Gonzaga de Oliveira, Nonlinear Biosynthetic Assembly of Alpiniamide by a Hybrid *cis*/*trans*-AT PKS-NRPS. *ACS Chem. Biol.* **15**, 1067–1077 (2020). doi:10.1021/acschembio.0c00081 Medline

70. C. Paulus, Y. Rebets, J. Zapp, C. Rückert, J. Kalinowski, A. Luzhetskyy, New Alpiniamides From *Streptomyces* sp. IB2014/011-12 Assembled by an Unusual Hybrid Non-ribosomal Peptide Synthetase *Trans*-AT Polyketide Synthase Enzyme. *Front. Microbiol.* **9**, 1959 (2018). doi:10.3389/fmicb.2018.01959 Medline

71. S. Alt, B. Wilkinson, Biosynthesis of the Novel Macrolide Antibiotic Anthracimycin. *ACS Chem. Biol.* **10**, 2468–2479 (2015). doi:10.1021/acschembio.5b00525 Medline

72. K. Jungmann, R. Jansen, K. Gerth, V. Huch, D. Krug, W. Fenical, R. Müller, Two of a Kind—The Biosynthetic Pathways of Chlorotonil and Anthracimycin. *ACS Chem. Biol.* **10**, 2480–2490 (2015). doi:10.1021/acschembio.5b00523 Medline

73. T. M. Nye, J. W. Schroeder, D. B. Kearns, L. A. Simmons, Complete Genome Sequence of Undomesticated *Bacillus subtilis* Strain NCIB 3610. *Genome Announc.* **5**, e00364-17 (2017). doi:10.1128/genomeA.00364-17 Medline

74. M. J. Bertin, A. Vulpanovici, E. A. Monroe, A. Korobeynikov, D. H. Sherman, L. Gerwick, W. H. Gerwick, The Phormidolide Biosynthetic Gene Cluster: A trans-AT PKS Pathway Encoding a Toxic Macrocyclic Polyketide. *ChemBioChem* **17**, 164–173 (2016). doi:10.1002/cbic.201500467 Medline

75. A. Nakabachi, R. Ueoka, K. Oshima, R. Teta, A. Mangoni, M. Gurgui, N. J. Oldham, G. van Echten-Deckert, K. Okamura, K. Yamamoto, H. Inoue, M. Ohkuma, Y. Hongoh, S. Y. Miyagishima, M. Hattori, J. Piel, T. Fukatsu, Defensive bacteriome symbiont with a drastically reduced genome. *Curr. Biol.* **23**, 1478–1484 (2013). doi:10.1016/j.cub.2013.06.027 Medline

76. K. P. Yuet, C. W. Liu, S. R. Lynch, J. Kuo, W. Michaels, R. B. Lee, A. E. McShane, B. L. Zhong, C. R. Fischer, C. Khosla, Complete Reconstitution and Deorphanization of the 3 MDa Nocardiosis-Associated Polyketide Synthase. *J. Am. Chem. Soc.* **142**, 5952–5957 (2020). doi:10.1021/jacs.0c00904 Medline

77. M. A. Storey, S. K. Andreassend, J. Bracegirdle, A. Brown, R. A. Keyzers, D. F. Ackerley, P. T. Northcote, J. G. Owen, Metagenomic Exploration of the Marine Sponge *Mycale hentscheli* Uncovers Multiple Polyketide-Producing Bacterial Symbionts. *mBio* **11**, e02997-19 (2020). doi:10.1128/mBio.02997-19 Medline

78. Y. Zou, H. Yin, D. Kong, Z. Deng, S. Lin, A trans-acting ketoreductase in biosynthesis of a symmetric polyketide dimer SIA7248. *ChemBioChem* **14**, 679–683 (2013). doi:10.1002/cbic.201300068 Medline

79. M. Steinmetz, R. Richter, Easy cloning of mini-Tn10 insertions from the Bacillus subtilis chromosome. *J. Bacteriol.* **176**, 1761–1763 (1994). doi:10.1128/jb.176.6.1761-1763.1994 Medline

80. X. Yan, H.-J. Yu, Q. Hong, S.-P. Li, Cre/lox system and PCR-based genome engineering in Bacillus subtilis. *Appl. Environ. Microbiol.* **74**, 5556–5562 (2008). doi:10.1128/AEM.01156-08 Medline

81. J. Moldenhauer, D. C. G. Götz, C. R. Albert, S. K. Bischof, K. Schneider, R. D. Süssmuth, M. Engeser, H. Gross, G. Bringmann, J. Piel, The final steps of bacillaene biosynthesis in Bacillus amyloliquefaciens FZB42: Direct evidence for β,γ dehydration by a trans-acyltransferase polyketide synthase. *Angew. Chem. Int. Ed.* **49**, 1465–1467 (2010). doi:10.1002/anie.200905468 Medline
